# Moralization and extremism robustly amplify myside sharing

**DOI:** 10.1093/pnasnexus/pgad078

**Published:** 2023-04-10

**Authors:** Antoine Marie, Sacha Altay, Brent Strickland

**Affiliations:** Department of Political Science, Aarhus University, Bartholins Allé 7, Aarhus 8000, Denmark; Digital Democracy Lab, Zurich University, Rämistrasse 71, Zürich 8006, Switzerland; Département d’Etudes Cognitives, ENS, EHESS, CNRS, PSL Research University, 24 rue Lhomond, Paris 75230, France; Africa Business School, The School of Collective Intelligence, UM6P, Rabat, Morocco

**Keywords:** extremism, fake news, moralization, political bias, polarization, sharing, social media

## Abstract

We explored whether moralization and attitude extremity may amplify a preference to share politically congruent (“myside”) partisan news and what types of targeted interventions may reduce this tendency. Across 12 online experiments (*N* = 6,989), we examined decisions to share news touching on the divisive issues of gun control, abortion, gender and racial equality, and immigration. Myside sharing was systematically observed and was consistently amplified when participants (i) moralized and (ii) were attitudinally extreme on the issue. The amplification of myside sharing by moralization also frequently occurred above and beyond that of attitude extremity. These effects generalized to both true and fake partisan news. We then examined a number of interventions meant to curb myside sharing by manipulating (i) the audience to which people imagined sharing partisan news (political friends vs. foes), (ii) the anonymity of the account used (anonymous vs. personal), (iii) a message warning against the myside bias, and (iv) a message warning against the reputational costs of sharing “mysided” fake news coupled with an interactive rating task. While some of those manipulations slightly decreased sharing in general and/or the size of myside sharing, the amplification of myside sharing by moral attitudes was consistently robust to these interventions. Our findings regarding the robust exaggeration of selective communication by morality and extremism offer important insights into belief polarization and the spread of partisan and false information online.

**Significance Statement**

Polarization between liberals and conservatives is partly anchored in disagreements about facts. Across 12 experiments (*N* = 6,989), we document one source of this polarization of belief by showing that people's moralization of an issue and attitude extremity exaggerate the selective sharing of partisan news on social media. Those effects were observed on both true and fake news (i.e. fabricated claims). Manipulations of the imagined political composition of the audience and account anonymity, as well as intervention messages highlighting our propensity to process and share partisan information in self-serving ways, had little effect on sharing intentions. By pinning down moralization and extremism as robust amplifiers of partisan communication on politics, our work makes an important contribution to research on the roots of belief polarization and online misinformation spread.

In the United States and a growing number of countries, liberals and conservatives are often incapable of reaching policy agreements on polarized issues, from gun control to economic policy to health care and climate change. While this outcome stems, in part, from divides in fundamental values (1), it also rests on disagreements about *factual claims* around which partisans and policymakers from all sides should, in principle, be able to converge. For instance, US liberals by and large believe that citizens’ right to carry weapons increases crime, whereas many conservatives think this right will drive homicide rates down by allowing people to protect themselves (2). Liberals typically consider human activities to be responsible for global warming and nuclear waste to pose major safety problems, while conservatives are more skeptical of those claims (3–5).

Ideally, citizens and decision-makers would go about attaining knowledge of the facts in an unbiased way and then pragmatically choose optimal policies and politicians on the basis of those facts. In practice, however, the flow of factual information citizens share, access, and evaluate is distorted at nearly all levels of the information processing chain. Differences in geographical residence, class, or historical traditions create constraints on the political leanings of the information people get access to from the onset of their lives (6–8). Later, individuals tend to preferentially befriend, work with, and marry people who share their political worldview (9, 10) and to selectively expose themselves to sources that are supportive of their ideology, both in real life (11, 12) and on social media (13). People are also often excessively skeptical toward ideologically incongruent information, in particular when they are highly morally committed to the issue at stake (14, 15)—whether this tendency stems from rational Bayesian calibration (16) or motivated thinking (7, 17).

In contrast to the above, which focuses on how information is received and searched, our approach differs by focusing on preferences regarding what types of content to communicate (see also Ekstrom and Lai (18) and Shin and Thorson (19) for related work). We ask a novel question by exploring whether people's perception of an issue as being of absolute moral importance and their level of attitude extremity might exaggerate “myside” communicative preferences (20, 21). Our focus on the specific role of moralization and attitude extremity is innovative not only in the context of the study of partisan communicative preferences but also within the myside bias literature which tends to not distinguish between moral and non-moral attitudes (18, 19).

Across the 12 online experiments reported below (*N* = 6,989), we examine sharing intentions on social media of true and “fake” partisan news stories touching on five controversial issues—gun control, abortion rights, sex equality, racial equality, and immigration—on which US liberals and conservatives tend to be polarized (4, 5, 22) and which many moralize highly. Studies exploring intentions to share news are now a well-established approach in political behavior research (see, for instance, Altay et al., Marie and Petersen, Pennycook et al., and Petersen et al. (23–27)). Given that growing shares of people worldwide are using social media to read, discuss, and disseminate partisan news and posts (28), our studies offer a window into the psychological roots of belief polarization on politics and the spread of strongly slanted and false information online.

To foreshadow, Experiments 1–4 establish the basic pattern of results: US respondents show a sharing preference for “mysided” news, which is consistently magnified on issues they consider of absolute moral importance and on which they have extreme attitudes, whether the news items are true or fake. We then look at a range of interventions which could plausibly be expected to curb myside sharing preferences: sharing of partisan news from an anonymous vs. a personal social media account (Experiments 5a and 5b), sharing to a politically like-minded audience vs. foes (Experiments 6a and 6b), and sharing after exposure to messages warning against the myside bias (Experiments 7 and 8) and after warning against the reputational consequences of sharing congruent misinformation coupled with an interactive rating task (Experiments 9 and 10).

## Experiments 1–4: willingness to share true and fake news

The primary question of interest in Experiments 1–4 was whether participants would show a “myside” sharing preference and whether this tendency would be amplified by issue moralization and by attitude extremity. We tested this below across true and fake news items and across various ways of framing our outcome variable in order to assess the robustness of any effect we may find.

### Method

#### Preregistrations

All surveys reported in this paper were implemented in Qualtrics. Power analyses conducted on initial pilot studies suggested required sample sizes of ∼320 to detect a small effect of *d* = 0.2 at 80% power. Experiments 1 and 3 were preregistered at https://aspredicted.org/blind.php?x=9bw9ww and Experiment 4 at https://osf.io/wrd5y. Experiment 2 was not preregistered, but its design and data analysis were identical to those of Experiments 1, 3, and 4. All data and R scripts of the experiments included in this project are available on the Open Science Framework at https://osf.io/5v8fw/.

#### Participants

We recruited 331 participants in Experiment 1, 421 participants in Experiment 2, 318 participants in Experiment 3, and 401 participants in Experiment 4. All respondents were US residents recruited on Amazon's Mechanical Turk (MTurk) in Experiments 1, 2, and 3 and on Prolific in Experiment 4. The sample of Experiment 4 was representative of the US population on age, sex, and ethnicity. The samples of Experiments 1–3 approximated representativeness. No cross-cultural replication of our studies outside the United States were run in this project as the battery of news items was restricted to issues relevant to US politics.

Thirty participants were excluded from Experiment 1 because they stopped during completion or failed the attention check, leaving 301 respondents (*M*_age_ = 34.7, *SD*_age_ = 9.6, 49% women). Thirty-nine participants were removed from Experiment 2 because they failed the attention check, leaving 382 participants (*M*_age_ = 35, *SD*_age_ = 10.7, 39% women). Thirteen participants were excluded from Experiment 3 because they failed the attention check, leaving 305 respondents (*M*_age_ = 39, *SD*_age_ = 12.3, 49% women). Five participants were excluded from Experiment 4 because they failed the attention check, leaving 396 respondents (*M*_age_ = 25, *SD*_age_ = 7, 84% women). Our final sample sizes thus reached our preregistered targets.

#### Designs

In Experiments 1, 2, and 3, participants were randomly exposed to eight true political news items touching on four controversial issues (abortion, gun control, racial equality, and gender equality) and to four true neutral news items. They were asked to report their willingness to share each item. Despite this being a measure of hypothetical sharing decisions, evidence from Mosleh et al. (29) suggests that self-reported willingness to share political news articles in online surveys correlates with actual sharing on social media, and that the perceived interestingness of a piece of news in online experiments predicts its success on actual social media (30).

Experiment 4 adopted the same design as Experiments 1–3, but we relied on eight fake political news items touching on the same four controversial issues, and no neutral news items were included. In all experiments, after exposure to the news, we collected information on participants’ position on each issue (to assess news congruence and attitude extremity) and on whether they moralized the issue.

#### Selection of the news items

**Fig. 1.**
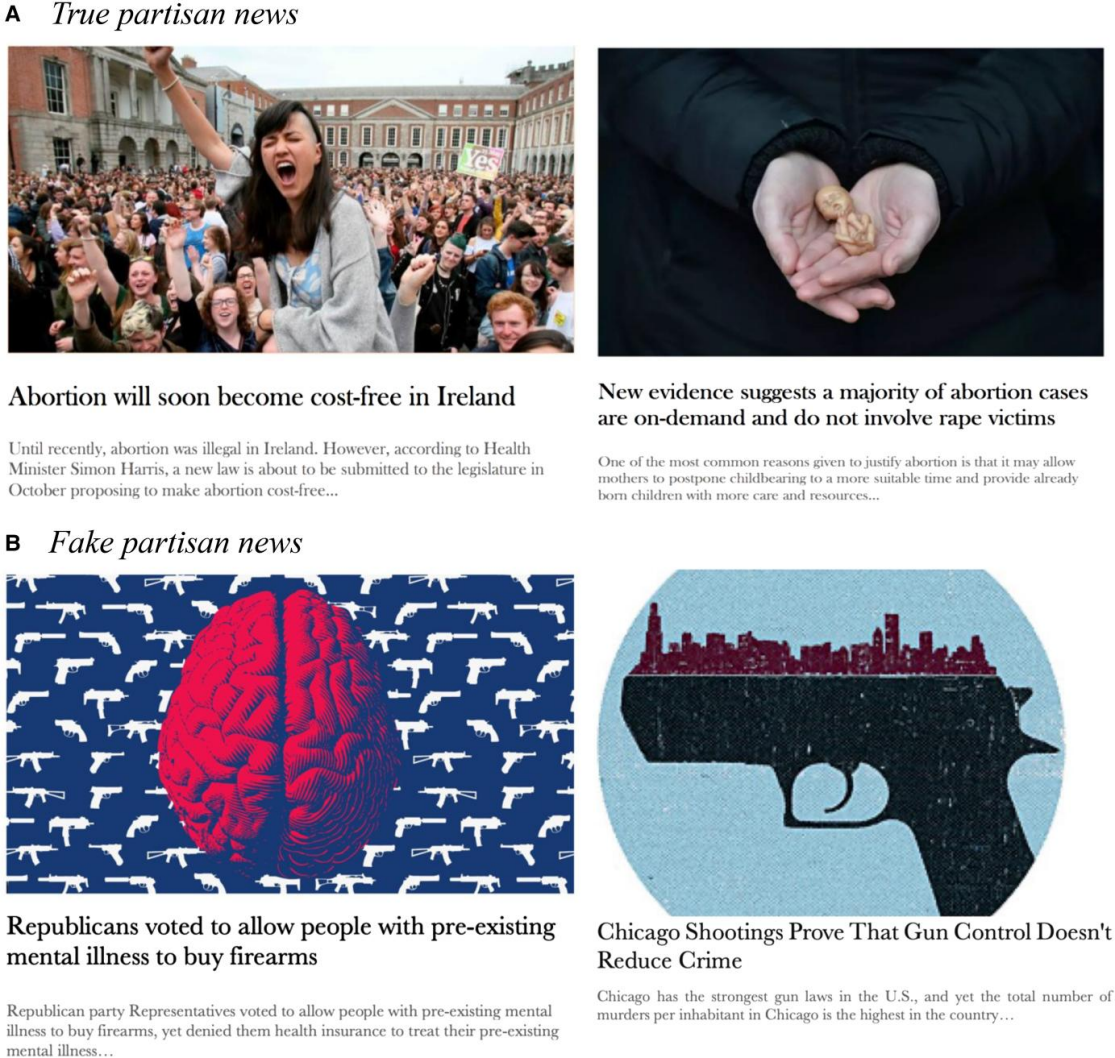
A) Examples of true partisan news items congruent for liberals (left) and conservatives (right) on the issue of abortion. B) Examples of fake partisan news items congruent for liberals (left) and conservatives (right) on the issue of gun control.

The true partisan news items used in Experiments 1–3 were inspired from true press articles found on mainstream news media websites. The fake partisan news items of Experiment 4 were taken from fact checking websites (e.g. Snopes and Politifact). Each news story's title was followed by a short introductory snippet and a picture chosen to illustrate its content (see Fig. 1 and online supplementary material, N–O). We removed information on the items’ sources and gave them all the same press-looking display.

All partisan news items, whether true (Experiments 1–3) or fake (Experiment 4), were organized in pairs pertaining to one controversial issue only: gun control, racial equality, gender equality, and abortion. All partisan headlines reported a societal event or fact as opposed to overtly expressing opinions. One partisan news story on each issue was typically congruent for liberals on the issue (e.g. gun control supporters), and the other was typically congruent for conservatives on the issue (e.g. gun rights supporters). More specifically, a partisan news story was defined as being politically *congruent* to a given political side or group when it reported on an event or societal fact that served its policy agenda, or pointed at a threat members of that political group would typically find credible and relevant.

To be retained in the stimuli database, the partisan news items, whether true or fake, had to clearly be identified by independent MTurk raters as favoring one political side on each issue in two pretest studies. The two pretest studies contained eight true and eight fake items, respectively. All the true and fake news items we pretested were perceived as advancing the political agenda we expected, such that all were retained for the experiments (all *P* < 0.05, one sample *t*-tests, one-tailed; see online supplementary material, O–R). To be accepted as final stimuli, perceived accuracy ratings of the fake news had to fall within “somewhat inaccurate” and “somewhat accurate” (see online supplementary material, R).

The four true neutral news items used in Experiments 1–3 were used as control items. They truthfully relate historical or biological facts that do not advance any political narrative.

Items and their ratings by US MTurkers can be found in the online supplementary material, O–R.

#### Materials and procedure

Experiments 1–3 were run in March–August 2019, Experiment 4 in July 2021. All studies started by requesting informed consent and told participants that they would be exposed to news stories found online and that their sources would not be made available to them (to focus attention on the claims). Participants then saw eight true partisan news and four true neutral items in Experiments 1–3 and eight fake partisan news items in Experiment 4, in a random order. We deemed internal replication on both true and fake news necessary to ensure the robustness of the main sharing patterns. Each headline, news snippet, and the corresponding picture were displayed above the willingness to share question. The question was formulated as “How likely would you be to pass along this news item to friends or people you like?” in Experiments 1–3 and as “How likely would you be to share this news story?” in Experiment 4. It offered four choices in Experiments 1, 2, and 4 [(0) “Very unlikely,” (1) “Unlikely,” (2) “Likely,” and (3) “Very Likely”] and a dichotomous alternative [(0) “Not Share” and (1) “Share”] in Experiment 3. The point of varying the number of choices and the formulation of the question was to assess the sensitivity of sharing patterns to our measurement. Those were qualitatively unaffected by the different operationalizations (see Results below). In contrast with other studies, Experiment 2 also probed participants on their motivations to share each item on three dimensions after collecting intentions to share (perceived accuracy, informativeness, and political usefulness in a random order).

Participants then responded to the questions, presented in a random order: “What is your position on the issue of [racial equality/gender equality/abortion/guns]?”. Their goal was to later determine whether or not each news item aligned with the participant's policy preferences on each issue (i.e. was congruent vs. incongruent, a dichotomous outcome), as well as to quantify the participant's degree of attitude extremity on each issue (a continuous outcome). Response choices ranged from [0] “I don’t care at all” (for gender and racial equality)/“Extremely Pro-life”/“Extremely Pro-gun rights” to [100] “Extremely in favor” (for gender and racial equality)/“Extremely Pro-choice”/“Extremely Pro-gun control.”

We then asked participants whether they moralized the issue—regardless of their policy position. Issue moralization was measured slightly differently across studies. In Experiment 4, it read: “Is one of the following issues of absolute moral importance to you? We mean issues that are connected to your core moral beliefs, convictions, and identity. In other words, the issues that are most likely to trigger strong positive or negative emotions in you. (Several choices possible).” Our intention was to capture issue moralization in a broad sense as encompassing (i) unwillingness to compromise on/prioritization of the issue, (ii) centrality to one's moral identity, and (iii) the activation of strong emotions by the issue, thereby connecting several strands of psychological research on moral attitudes together (e.g. 20, 21, 31–33). Responses to the issue moralization question were collected as a set of binary outcomes by participants ticking one box in front of each issue label (“Gun control vs. Gun rights,” “Racial Equality,” “Gender Equality,” and “Pro-life vs. Pro-choice”). We coded ticking of a box as indicating high issue moralization, and not ticking as meaning low issue moralization.

In the first formulation of the issue moralization item used in Experiments 1–3, however, the question did not include the epithet “moral” (“Is one of the following issues of absolute importance to you?”; see online supplementary material, S). This was because we had reasoned that the controversial issues chosen (gun control, abortion, etc.) would automatically be understood in moral terms. After all, polarized issues tend to be intuitively framed by many partisan minds as the struggle between a positively moralized in-group (viewed as “right” on the issue and virtuous) and a negatively moralized out-group (viewed as “wrong” on the issue and uncooperative if not hostile; 22, 34). We also know that partisan disagreements are partly anchored in differences in sensitivity of the “moral foundations” between liberals and conservatives (1). Moreover, the four issues were selected based on a norming study asking respondents on which topics they were least likely to accept political compromise (see online supplementary material, A), a key consequence of moralization (32). Thus, we initially viewed it as unnecessary to include the word “moral” in the question. Later on, however, we recognized that the “absolute moral importance” formulation could target the issue moralization construct more directly and explicitly, so we adopted it in Experiment 4 and all the studies run chronologically after it.

Experiments 1–4 ended with demographic questions and a general attention check, applied in all studies in this paper (see “videogame” in online supplementary material, T).

#### Analyses

Responses to the attitude questions were originally collected on axes ranging from [0] the most conservative position to [100], the most liberal position on each issue. To facilitate interpretation of the data during our analyses on an intuitive liberal (left)–conservative (right) axis on each issue, scores were reverse coded prior to statistical analyses, to arrive at [0] for the most liberal position and [100] for the most conservative position on each issue. From there, a political news story advancing a conservative narrative on an issue was considered as *congruent* with a participant's attitude on the issue if that attitude was ≥50 and as *incongruent* if the participant's attitude on the issue was strictly <50. The reverse was true for news advancing a typically liberal narrative.

**Fig. 2.**
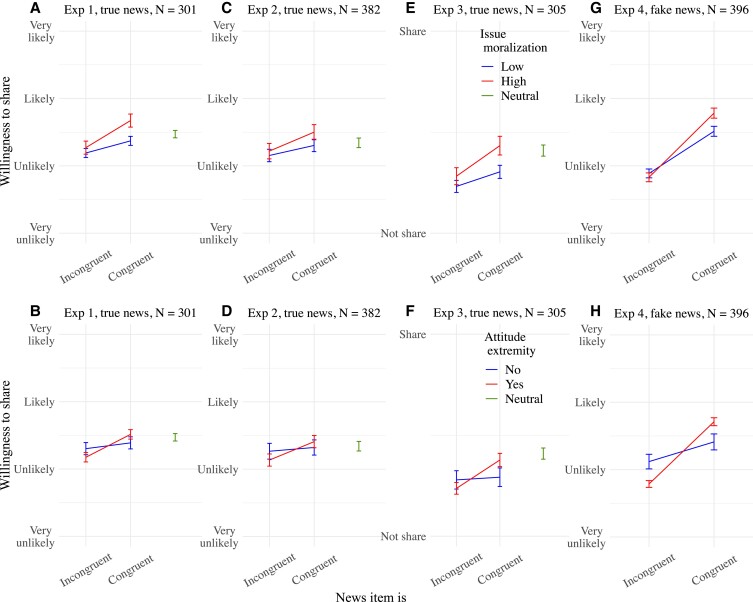
Mean willingness to share news items as a function of news congruence and issue moralization (top) and news congruence and attitude extremity (bottom) in Experiments 1 to 4. A, C, E, and G represent congruence × issue moralization interactions alone. B, D, F, and H represent congruence × attitude extremity interactions alone. Means are surrounded by 95% CIs. The plots of Experiment 4 do not contain the two hostile fake abortion items markedly less subject to myside sharing.

As to a participant's level of *attitude extremity* on each issue, it was defined by taking the absolute distance between their attitude score on the issue and the middle of the scale, 50, and then by rescaling that distance score to a 0–1 continuous range (by dividing scores by 50). The goal of that rescaling to a 0–1 range was to make the attitude extremity measure more comparable to the issue moralization measure. In contrast with issue moralization (dichotomous) and news congruence (dichotomous), attitude extremity as used in the analyses was a continuous measure. (For data visualizations, moreover, a respondent was coded dichotomously as being attitudinally extreme [“Yes”] if her level of attitude extremity was strictly >0.5 and not extreme [“No”] when this was not the case; see Fig. 2.)

All the data analyses in this paper were performed in R (version 3.6.2) using R Studio (Version 1.2.5033). All regression models were mixed-effects models (using the lme4 package; 35). We specified random intercepts for participants and news items and random slopes for the key predictors included in each model (when convergence could not be found, random slopes for predictors were removed until convergence was restored). Main effects are reported from models containing only the main effects and interactions from models containing the main effects and their interactions (see online supplementary material, D–G for regression tables of Experiments 1–4). We report standardized regression coefficients, coefficients’ 95% confidence intervals (CIs) between brackets, and *P-*values.

In all experiments, willingness to share the news stories was first regressed on news congruence, issue moralization, and the congruence × issue moralization interaction; second on news congruence, attitude extremity, and the congruence × attitude extremity interaction; and third on congruence × issue moralization while controlling for congruence × attitude extremity (see online supplementary material, D–G). This was meant to test for the possibility of issue moralization and attitude extremity having different effects on sharing.

By default, and as preregistered, analyses in all studies in this paper were run on the full set of items, whether true or fake. However, exploratory by issue analyses of Experiments 4 and 8 (cf. below) revealed that two specific items, the fake news stories ascribing highly provocative statements to Bernie Sanders (“Bernie Sanders wants to set cut-off date for abortions up to 7 months,” congruent for Republicans) and Mike Pence (“Allowing abortion for rape victims will only incentivize women to report fictitious rapes, Pence said off the record,” congruent for Democrats) on the issue of abortion were uniquely less subject to myside sharing than the other items (see online supplementary material, H, by issue analyses).^1^ We therefore report sharing patterns of the fake news items used in Experiments 4 and 8 first while including those two items and then while excluding them. All other analyses in the paper include all the item participants viewed and rated in each study.

### Results

Table 1 provides summary regression tables from models run in Experiments 1–4 (see also online supplementary material, D–G), and Fig. 2 plots congruence × issue moralization and congruence × attitude extremity interactions from those studies. Main analyses reported in Table 1 and in text below are on individual experiments separately, but additional analyses were also run on Experiments 1–3 pooled to maximize power, as they all contained the same true partisan news.

**Table 1.** Regression tables of willingness to share news as a function of news congruence and issue moralization (left) and news congruence and attitude extremity (right) in Experiments 1 to 4. Betas are standardized regression coefficients, and brackets are 95% confidence intervals. Betas in bold are statistically significant. ns *P* > .05, *P < .05, **P < .01, ***P < .001.

		Exp. 1 (true partisan news)	Exp. 2 (true partisan news)	Exp. 3 (true partisan news)	Exp. 1–3 pooled (true partisan news)	Exp. 4 (fake partisan news)	
		All items				All items	No abortion items
Model with main effects only	Political congruence	***ß* = 0.35** [0.27, 0.42] ***	***ß* = 0.28** [0.20, 0.36] ***	***ß* = 0.28** [0.20, 0.36] ***	***ß* = 0.29** [0.25, 0.33] ***	***ß* = 0.44** [0.35, 0.52] ***	***ß* = 0.60** [0.49, 0.70] ***
	Issue moralization	***ß* = 0.17** [0.10, 0.24] ***	***ß* = 0.14** [0.06, 0.22] ***	***ß* = 0.19** [0.11, 0.27] ***	***ß* = 0.18** [0.13, 0.23] ***	***ß* = 0.14** [0.07, 0.21] ***	***ß* = 0.13** [0.05, 0.21] ***
	Attitude extremity	*ß* = 0.03 [−0.01, 0.07] ns	*ß* = 0.03 [−0.02, 0.08] ns	*ß* = 0.04 [−0.01, 0.08] ns	***ß* = 0.03** [0.00, 0.05] *	***ß* = 0.05** [0.01, 0.09] *	*ß* = 0.03 [−0.01, 0.07] ns
Model with one interaction term	Congruence × issue moralization	***ß* = 0.35** [0.22, 0.48] ***	***ß* = 0.20** [0.05, 0.34] **	***ß* = 0.26** [0.13, 0.40] ***	***ß* = 0.24** [0.16, 0.32] ***	*ß* = 0.08 [−0.04, 0.19] ns	***ß* = 0.30** [0.16, 0.43] ***
	Congruence × attitude extremity	***ß* = 0.15** [0.09, 0.21] ***	***ß* = 0.15** [0.08, 0.21] ***	***ß* = 0.18** [0.11, 0.24] ***	***ß* = 0.13** [0.09, 0.17] ***	***ß* = 0.18** [0.12, 0.24] ***	***ß* = 0.34** [0.27, 0.41] ***
Model with two interactions terms	Congruence × issue moralization	***ß* = 0.28** [0.14, 0.41] ***	*ß* = 0.10 [−0.05, 0.25] ns	***ß* = 0.17** [0.03, 0.31] *	***ß* = 0.16** [0.08, 0.25] ***	*ß* = −0.05 [−0.18, 0.07] ns	*ß* = 0.11 [−0.03, 0.25] ns
	Congruence × attitude extremity	***ß* = 0.11** [0.04, 0.17] **	***ß* = 0.13** [0.06, 0.20] ***	***ß* = 0.15** [0.08, 0.22] ***	***ß* = 0.11** [0.07, 0.14] ***	***ß* = 0.19** [0.12, 0.25] ***	***ß* = 0.32** [0.24, 0.39] ***

In all four experiments, participants were more willing to share politically congruent than incongruent news, both true and fake—i.e. to do “myside” sharing.

There were main effects of issue moralization, such that willingness to share true and fake news was overall greater on issues of absolute moral importance. Small positive main effects of attitude extremity on sharing were also observed when pooling Experiments 1–3 on true news and in Experiment 4 on fake news when including the less shared fake abortion items.

Most importantly, the myside sharing preference of true and fake news was consistently magnified on moralized issues (congruence × issue moralization). Myside sharing of true and fake news also reliably increased with attitude extremity on the issue (congruence × attitude extremity).

We then looked at whether issue moralization amplified myside sharing above and beyond attitude extremity. In Experiments 1 and 3 on true news, that is, in two out of the four studies taken separately, the congruence × issue moralization interaction came out as significant even when controlling for the congruence × attitude extremity interaction. In Experiments 2 (true news) and 4 (fake news) taken separately, however, the congruence × issue moralization interaction did not come out as significant when controlling for the congruence × attitude extremity interaction. Pooling Experiments 1–3 caused the congruence × issue moralization interaction to become significant again while controlling for congruence × attitude extremity. This suggests that issue moralization can amplify myside sharing above and beyond attitude extremity when statistical power is high.

Attitude extremity, but not issue moralization, had the additional effect of making participants even less likely to share incongruent fake news in Experiment 4 when excluding the less shared fake abortion items, but this negative effect did not reach significance when including all the fake news in Experiment 4 nor on true news in Experiments 1–3.

Post hoc analyses run to explore potential effects of partisanship suggested that the size of myside sharing tended to be smaller among Republicans than Democrats, as suggested by negative coefficients of congruence × partisanship interactions, which were marginally significant in Experiment 2 [*ß* = −0.13 (−0.28, 0.02), *P* = 0.08] and Experiment 4 [*ß* = −0.14 (−0.29, 0.01), *P* = 0.07]. Our studies did not power for such effects of partisanship so these are underpowered.

For interested readers, online supplementary material contains various additional analyses: restricted analyses by issue of the pooled data set of Experiments 1–3 on true news (online supplementary material, D–G), restricted analyses by issue of Experiment 4 on fake news (online supplementary material, H), as well as exploratory analyses of the motivations to share in Experiment 2 (online supplementary material, E), not reported in the main text.

### Discussion of Experiments 1–4

Experiments 1–4 repeatedly found that US MTurkers were more likely to share politically congruent than incongruent news stories, whether true or false, touching on four polarizing issues pertaining to the context of US politics (in line with 18, and 19). Furthermore, our results show that this “myside” sharing of true and fake news is consistently amplified when respondents moralize and are attitudinally extreme on the issue. In two out of the four studies, both effects held when controlling for each other, meaning that the effects of moralization could not be reduced to the influence of attitude extremity. Moreover, while moralizing the issue tended to increase sharing of incongruent stories—perhaps with the (unfulfilled) intention to denounce or mock their content—being attitudinally extreme tended to decrease sharing of incongruent stories. This confirms that issue moralization and attitude extremity have distinct psychological effects.

## Experiments 5a and 5b: Sharing from anonymous vs. personal account—true and fake news

Proposals to prevent social media users from creating anonymous accounts are common (e.g. 36). They hypothesize that if users can be easily identified, they will be more cautious regarding the types of divisive and potentially false information they share due to fear of reputational damage. In order to gain information about whether identifiability could alter sharing behavior, Experiments 5a and 5b asked participants to imagine sharing the news from a personal vs. an anonymous account.^2^

### Method

#### Participants and design

We first run a pilot study, Experiment 5a, for which we recruited 325 participants on MTurk. Thirty-eight participants were removed from the data in Experiment 5a because they failed the attention or the English fluency checks, so 287 remained (*M*_age_ = 37, *SD*_age_ = 11, 47% women). The study was run in May 2019 and used the same eight true news as Experiments 1–3 (abortion, gun control, racial equality, and gender equality) and measured willingness to share on a dichotomous scale.

Experiment 5b, the final experiment, used six fake news (gun control, racial equality, and gender equality), a four-point measure of willingness to share, and was run in October 2021. The study was preregistered at https://osf.io/3jgea (see online supplementary material, B). Experiment 5b and its preregistration did not include the hostile fake items touching on abortion found to be less subject to myside sharing in Experiment 4. We reasoned that the best test of the efficacy of a contextual manipulation would be to assess its influence over the items that showed the relevant effects most strongly. A power analysis suggested 800 participants were needed to detect a small effect at 80% power. We recruited 905 US citizens, representative of the national population on age, ethnicity, and sex, on Prolific. However, we excluded 99 participants who failed either the attention check or the manipulation check, such that 806 participants remained in the data set, our target sample size (*M*_age_ = 43.8, *SD*_age_ = 16.4, 53% women).

#### Materials and procedure

The instructions used in Experiment 5a asked participants to imagine that they are sharing news on behalf of an association they are working for and that in order to do so, they are using their own account (making their identity public) vs. an anonymous account. The idea of having participants imagine they are working for an association came from our intention to find a context in which an anonymous account can still have an audience of followers. In order to make the imagined context realistic for participants, the dependent variable was adapted to a willingness to pay for sharing. Additional description of the materials of Experiment 5a can be found in online supplementary material, I.

We then realized that asking participants to imagine working for an association could still carry reputational consequences even in the anonymous account condition (e.g. sharing something that one's employer dislikes may lead one to lose one's job). Experiment 5b thus removed any reference to an association and adopted the following formulation: “How likely would you be to share this news story from an anonymous social media account (no one online can know it's you)? vs. [from your personal social media account (everyone online can know it's you)?]” Experiment 5b also included a manipulation check (see online supplementary material, I).

### Results

As in prior studies, participants reported a higher willingness to share politically congruent news [Experiment 5a, true news: *ß* = 0.41 (0.34, 0.49), *P* < 0.001; Experiment 5b, fake news: *ß* = 0.40 (0.34, 0.46), *P* < 0.001].

Issue moralization was associated with higher overall willingness to share true news [Experiment 5a: *ß* = 0.19 (0.12, 0.26), *P* < 0.001] and fake news [Experiment 5b: *ß* = 0.14 (0.08, 0.20), *P* < 0.001] but no such main effect emerged for attitude extremity. Being high in attitude extremity, however, was associated with a lower willingness to share incongruent true news [Experiment 5a: *ß* = −0.08 (−0.12, −0.03), *P* = 0.002] and incongruent fake news [Experiment 5b: *ß* = −0.09 (−0.13, −0.05), *P* < 0.001].

The myside sharing preference was again magnified both by issue moralization [congruence × issue moralization in Experiment 5a, true news: *ß* = 0.42 (0.30, 0.54), *P* < 0.001; in Experiment 5b, fake news: *ß* = 0.42 (0.33, 0.51), *P* < 0.001] and by attitude extremity on the issue [congruence × attitude extremity in Experiment 5a, true news: *ß* = 0.23 (0.17, 0.29), *P* < 0.001; in Experiment 5b, fake news: *ß* = 0.26 (0.22, 0.31), *P* < 0.001].

When including the two interactions in the model, myside sharing was found to be independently amplified by issue moralization [congruence × issue moralization in Experiment 5a, true news: *ß* = 0.29 (0.16, 0.41), *P* < 0.001; in Experiment 5b, fake news: *ß* = 0.27 (0.18, 0.37), *P* < 0.001] and by attitude extremity [congruence × attitude extremity in Experiment 5a, true news: *ß* = 0.18 (0.11, 0.24), *P* < 0.001; in Experiment 5b, fake news: *ß* = 0.21 (0.16, 0.26), *P* < 0.001].

As regards the manipulation of account type, imagining using a personal account with higher reputational repercussions (vs. an anonymous account) did not overall affect willingness to share in general (no main effect of account type) nor did it affect the size of the myside sharing preference (no congruence × account type interaction), either on true (Experiment 5a) or fake news (Experiment 5b). There were no congruence × moral covariates × account interactions in either study.

We finally looked at possible influences of partisanship using post hoc analyses in Experiment 5b on fake news, with the biggest sample. Note that our studies did not power for partisanship effects so those are likely underpowered. Myside sharing was less pronounced among Republicans than Democrats [congruence × partisanship: *ß* = −0.33 (−0.44, −0.21), *P* < 0.001]. Imagining sharing the news from one's personal account (vs. an anonymous account) did not decrease overall sharing of true or fake political news among either group. However, it did decrease the size of myside sharing of fake news among Republicans [congruence × account on Reps only: *ß* = −0.22 (−0.41, −0.04), *P* = 0.019], but not among Democrats [congruence × account on Dems only: *ß* = 0.03 (−0.10, 0.17)].

**Fig. 3.**
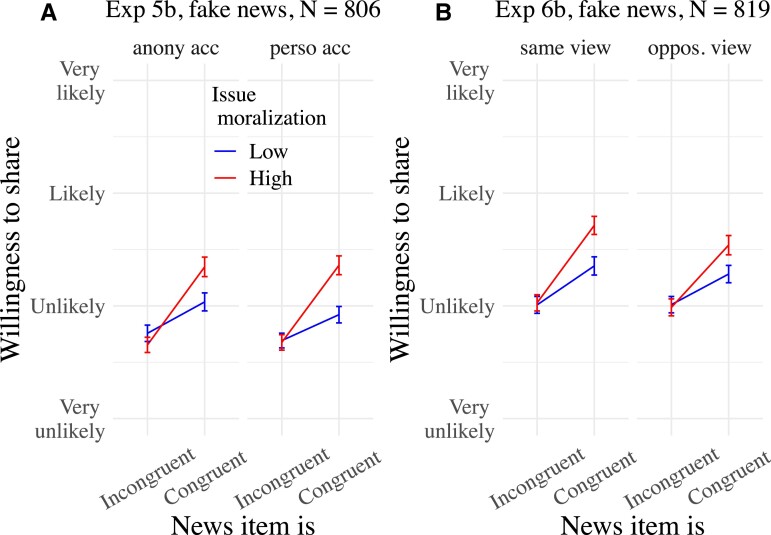
Mean willingness to share news items as a function of news congruence, issue moralization, and experimental condition in Experiments 5b (A) and 6b (B). Experiment 5b manipulated whether respondents imagined sharing the news from an anonymous vs. a personal social media account, and Experiment 6b whether they imagined sharing the news to people who have the same view vs. an opposite view from them on the issue. Only the congruence × issue moralization interactions are represented. Means are surrounded by 95% CIs.

See Fig. 3 and online supplementary material, I, for regression tables.

### Discussion of Experiments 5a and 5b

Experiments 5a and 5b provide yet another demonstration that issue moralization and attitude extremity magnify myside sharing preferences of both true and fake news. Issue moralization also amplified myside sharing above and beyond attitude extremity on both news types. However, myside sharing was reduced by the use of a personal (vs. an anonymous) account only among Republicans, not Democrats. This suggests that the politically popular idea of banning anonymous accounts may not reliably reduce sharing of strongly partisan and potentially false claims.

## Experiments 6a and 6b: sharing to like-minded people vs. foes—true and fake news

The previous experiments put participants in the mindset of sharing news stories to “friends or people [they] like.” People tend to befriend and follow politically like-minded others, so most of those online “friends” could be assumed to agree politically with the participants. In contrast, Experiments 6a and 6b tested whether sharing news to political foes would reduce the size of myside sharing.

### Method

#### Participants and design

We began by running a pilot study in August 2019, Experiment 6a, for which we recruited 290 participants on MTurk. Eleven participants were removed from Experiment 6a because they failed the attention or English fluency checks, leaving 279 respondents in the data set (*M*_age_ = 36, *SD*_age_ = 11, 45% female). We used the same eight true partisan news as in Experiments 1–3 (abortion, gun control, racial equality, and gender equality). Experiment 6a manipulated whether participants were asked to imagine sharing the news to people they agree with vs. disagree with on each issue.

Experiment 6b, the final experiment, was preregistered at https://osf.io/tjeh4 (see online supplementary material, B) and run in October 2021. It asked participants to imagine sharing news to people that have the same vs. an opposite position from them on each issue. Our power analysis suggested 800 participants were needed to detect a small effect of audience composition at 80% power. We recruited a sample of 955 people on Prolific, representative of the US population on age, ethnicity, and sex. 136 participants were removed from the data because they failed the attention or the manipulation checks, leaving 819 participants, our preregistered target sample size (*M*_age_ = 40, *SD*_age_ = 16, 53% female). Experiment 6b used six fake partisan news stories exposed in a random order (gun control, racial equality, and gender equality).

#### Materials and procedure

Experiment 6a employed true news, a dichotomous willingness to share measure, and experimentally manipulated the formulation of the willingness to share question: “Would you share this news story to people [you agree with politically? ] vs. [you disagree with politically?].” We later realized, however, that the concept of someone “disagreeing” with oneself was vague: it could refer to someone with an opposite view from one's own on a polarized issue (e.g. a pro-life person if one is pro-choice), just as it could refer to someone more extreme but on the same side as oneself on the issue (e.g. a die-hard pro-life activist if one is only leaning pro-life).

We solved this issue in Experiment 6b, which employed fake news and a four-point outcome variable, by asking: “How likely would you be to share this news story to people who [have the same position as you on this issue?]” vs. “[have an opposite position from you on this issue?]” Experiment 6b also included a manipulation check (see online supplementary material, J).

### Results

Replicating the myside sharing effect of previous studies, politically congruent news stories were more shared than incongruent ones, whether true [Experiment 6a: *ß* = 0.31 (0.22, 0.39), *P* < 0.001] or fake [Experiment 6b: *ß* = 0.38 (0.32, 0.45), *P* < 0.001].

There also was a positive main effect of issue moralization on true [Experiment 6a: *ß* = 0.15 (0.06, 0.24), *P* < 0.001] and fake news sharing [Experiment 6b: *ß* = 0.18 (0.12, 0.23), *P* < 0.001] as well as of attitude extremity on both news types [Experiment 6a, true news: *ß* = 0.07 (0.02, 0.11), *P* = 0.003; Experiment 6b, fake news: *ß* = 0.05 (0.02, 0.08), *P* = 0.001]. In contrast, being high in attitude extremity was associated with a decreased willingness to share incongruent fake news in Experiment 6b [*ß* = −0.07 (−0.11, −0.04), *P* < 0.001]. This effect was not found on true news in Experiment 6a.

Myside sharing was again magnified both by issue moralization [congruence × issue moralization in Experiment 6a, true news: *ß* = 0.19 (0.05, 0.33), *P* = 0.007; in Experiment 6b, fake news: *ß* = 0.30 (0.21, 0.39), *P* < 0.001] and by attitude extremity [congruence × attitude extremity in Experiment 6a, true news: *ß* = 0.14 (0.07, 0.21), *P* < 0.001; in Experiment 6b, fake news: *ß* = 0.25 (0.20, 0.29), *P* < 0.001].

Moreover, in Experiment 6b, both issue moralization [congruence × issue moralization: *ß* = 0.14 (0.04, 0.24), *P* = 0.004] and attitude extremity [congruence × attitude extremity: *ß* = 0.22 (0.17, 0.27), *P* < 0.001] independently amplified myside sharing of fake news when the interactions controlled for each other. A similar pattern emerged in Experiment 6a on true news, but the congruence × issue moralization interaction did not come out significant [*ß* = 0.11 (−0.04, 0.25), *P* = 0.15] when controlling for congruence × attitude extremity [*ß* = 0.12 (0.05, 0.19), *P* = 0.001].

As regards the effect of our manipulation of the audience, there was no main effect of sharing news to political foes or people with an opposite view on the issue (vs. like-minded people) on either true or fake news in Experiment 6a or 6b. Sharing news to an uncongenial audience, however, had a small reducing effect on the size of people's myside sharing preference (compared to a like-minded audience), both on true news [congruence × audience: Experiment 6a: *ß* = −0.17 (−0.32, −0.02), *P* = 0.03] and fake news [congruence × audience: Experiment 6b: *ß* = −0.11 (−0.21, −0.01), *P* = 0.024]. No congruence × moral covariates × audience interaction was found in either study.

We finally investigated possible influences of partisanship with post hoc analyses in Experiment 6b on partisan fake news, with a larger sample. We did not power for effects of partisanship, so those effects are underpowered. Myside sharing of fake news was again less pronounced among Republicans than Democrats [congruence × partisanship: *ß* = −0.29 (−0.41, −0.17), *P* = 0.001]. Restricted analyses suggested that imagining sharing the news to an uncongenial (vs. like-minded) audience was associated with similar yet nonsignificant decreases in overall sharing of partisan fake news [among Democrats: *ß* = −0.10 (−0.22, 0.02), *P* = 0.1; among Republicans: *ß* = 0.13 (−0.33, 0.07), *P* = 0.2] and with similar nonsignificant reductions in the size of myside sharing of partisan fake news [among Democrats: *ß* = −0.11 (−0.23, 0.00), *P* = 0.059; among Republicans: *ß* = −0.17 (−0.38, 0.05), *P* = 0.127].

See Fig. 3 and online supplementary material, J, for regression tables.

### Discussion of Experiments 6a and 6b

Experiments 6a and 6b confirmed the robustness of our prior findings that US respondents have a myside sharing preference of true and fake news and that it is exaggerated when they moralize and are attitudinally extreme on the issue. Moreover, the amplification effect of issue moralization occurred above and beyond that of attitude extremity. Finally, imagining an audience of foes rather than like-minded people slightly reduced participants’ myside sharing of true and fake news, presumably because this setting made them more aware of the controversial character of politically congruent claims.

## Experiments 7 and 8: intervention message on myside bias—true and fake news

Psychologists have recently explored ways of reducing the spread of misinformation through interventions such as nudges and media literacy tips inviting people to consume information more wisely under uncertainty (e.g. (26) accuracy prompt, (37) digital literacy tips, or (38) inoculation techniques). Experiments 7 and 8 tested whether a targeted and personalized educational message informing participants of their inclination to process political news in ways partial to their political goals and values—a form of undesirable myside bias—would decrease the size of their myside sharing.

### Method

#### Participants and design

A power analysis recommended 800 participants per study to detect a small effect at 80% power. A total of 1,166 participants were recruited on MTurk in February 2020 for Experiment 7, which comprised eight true partisan news items (abortion, gun control, racial equality, and gender equality). 175 participants were removed from the data because they failed either the attention check or the English proficiency check. 889 participants remained in the data set (*M*_age_ = 39, *SD*_age_ = 12, 45% women). Participants in the control condition merely reported their willingness to share each item, whereas in the intervention condition, the headlines were preceded by a message about our inclination to process political information in ways partial to our political agenda (the “myside bias”).

Experiment 8, which recruited 937 participants in December 2019 on MTurk, followed the same design as Experiment 7 (with an intervention message) and used the same eight fake political news items as in Experiment 4 (abortion, gun control, racial equality, and gender equality). 197 participants were removed because they either did not pass the attention check or failed the English proficiency test. 740 participants remained in the data set (*M*_age_ = 37, *SD*_age_ = 12, 41% women).

#### Materials and procedure

The procedure for Experiments 7 and 8 was identical to previous studies except that both experiments contained a control condition with no message and a treatment condition with an intervention message. The message was inspired from scientific research on prior-based and politically motivated thinking and provided a scientific reference (7). It warned participants against a tendency to “favor information that fits one's goals and values, and to disregard information that doesn’t”, in particular on the issues one regards as being “of absolute importance.” In order for the message to embed the labels of those latter issues, participants were asked to report their political attitudes before being allocated to a condition. Below is the message we used (emphasis in bold in questionnaire):

*Please read carefully the following instruction:*

Previous research has shown that the issue(s) you judge as having absolute importance are the ones on which you are *most likely to be politically biased*. In your case, they are:

>>> [Issue(s) rated by respondent as being of “absolute importance” displayed] <<<

Typically, political bias causes one to significantly *favor information that fits one's goals and values, and to disregard information that doesn’t*.

Source: Kahan (7).

In both experiments, intentions to share were collected on a dichotomous scale [(0) “Not share,” (1) “Share”].

### Results

Results from Experiment 7 on true news are from the whole data set. Analyses of myside sharing and its interaction with moral covariates in Experiment 8 on fake news, in contrast, follow the same procedure as in Experiment 4: they are reported first from the whole data set and second from data excluding the two hostile fake abortion items which by issue analyses revealed were less subject to myside sharing (cf. Experiment 4 for details).

Replicating prior findings, participants were more likely to share politically congruent than incongruent news stories, whether true [Experiment 7: *ß* = 0.36 (0.31, 0.41), *P* < 0.001] or fake [Experiment 8: *ß*_all items_ = 0.30 (0.25, 0.35), *P* < 0.001; *ß*_no abortion items_ = 0.37 (0.31, 0.43), *P* < 0.001].

On both true and fake news, there were positive main effects on sharing of issue moralization [Experiment 7: *ß* = 0.25 (0.20, 0.30), *P* < 0.001; Experiment 8: *ß*_all items_ = 0.12 (0.07, 0.18), *P* < 0.001; *ß*_no abortion items_ = 0.16 (0.10, 0.22), *P* < 0.001] and attitude extremity [Experiment 7: *ß* = 0.11 (0.08, 0.13), *P* < 0.001; Experiment 8: *ß*_all items_ = 0.05 (0.02, 0.08), *P* < 0.001; *ß*_no abortion items_ = 0.06 (0.03, 0.09), *P* < 0.001], regardless of news congruence.

Myside sharing was again stronger on highly moralized issues, whether the stories were true [congruence × issue moralization: Experiment 7: *ß* = 0.29 (0.21, 0.37), *P* < 0.001] or fake [congruence × issue moralization: Experiment 8: *ß*_all items_ = 0.21 (0.12, 0.29), *P* < 0.001; *ß*_no abortion items_ = 0.28 (0.18, 0.38), *P* < 0.001]. Attitude extremity also amplified the myside preference, whether the news stories were true [congruence × attitude extremity: Experiment 7: *ß* = 0.20 (0.16, 0.23), *P* < 0.001] or fake [congruence × attitude extremity: Experiment 8: *ß*_all items_ = 0.17 (0.12, 0.21), *P* < 0.001; *ß*_no abortion items_ = 0.24 (0.19, 0.28), *P* < 0.001].

When those interactions were simultaneously included in the model, issue moralization and attitude extremity were found to independently amplify myside sharing in Experiment 7 on true news [congruence × issue moralization: *ß* = 0.16 (0.08, 0.25), *P* < 0.001; congruence × attitude extremity: *ß* = 0.17 (0.13, 0.21), *P* < 0.001]. A similar trend was observed in Experiment 8 when both interactions controlled for each other. When excluding the two fake abortion items, the congruence × issue moralization interaction remained significant [*ß*_all items_ = 0.09 (−0.00, 0.18), *P* = 0.061; *ß*_no abortion items_ = 0.11 (0.01, 0.22), *P* = 0.037] even when controlling for congruence × attitude extremity [*ß*_all items_ = 0.15 (0.11, 0.19), *P* < 0.001; *ß*_no abortion items_ = 0.22 (0.17, 0.27), *P* < 0.001].

As regards the intervention message warning against the myside bias, it marginally decreased overall sharing of true partisan news [main effect of condition: Experiment 7: *ß* = −0.08 (−0.16, 0.00), *P* = 0.063], but not fake partisan news [Experiment 8: *ß* = −0.02 (−0.11, 0.07), *P* = 0.67]. The message also slightly decreased the size of respondents’ myside sharing both when the news stories were true [congruence × condition in Experiment 7: *ß* = −0.08 (−0.17, 0.00), *P* = 0.049] and fake [congruence × condition in Experiment 8: *ß* = −0.09 (−0.18, −0.00), *P* = 0.055], although the latter decrease was only marginally significant. No congruence × moral covariates × condition interaction was found in either study.

We finally investigated possible influences of partisanship with post hoc analyses. Our studies did not power for partisanship effects, so those are underpowered. Consistent with prior findings, myside sharing was smaller among Republicans than Democrats in both studies [congruence × partisanship in Experiment 7 on true news: *ß* = −0.22 (−0.30, −0.14), *P* < 0.001; in Experiment 8 on fake news: *ß* = −0.20 (−0.28, −0.11), *P* < 0.001]. In Experiment 7 on true partisan news, restricted analyses suggested that exposure to the message on myside bias was associated with a small nonsignificant decrease in overall sharing among Republicans [main effect of condition: *ß* = −0.09 (−0.22, 0.03), *P* = 0.14] but not among Democrats [*ß* = −0.02 (−0.13, 0.09), *P* = 0.69]. Still in Experiment 7 using true partisan news, exposure to the message seemed associated with nonsignificant decreases in the size of myside sharing of roughly the same magnitude among the two groups of partisans [congruence × condition among Dems: *ß* = −0.11 (−0.23, 0.01), *P* = 0.07; among Reps: *ß* = −0.07 (−0.18, 0.03), *P* = 0.17]. In Experiment 8 on fake partisan news, restricted analyses suggested null effects of the message on overall sharing [main effect of condition among Dems: *ß* = 0.05 (−0.06, 0.17), *P* = 0.36; among Reps: *ß* = −0.03 (−0.17, 0.11), *P* = 0.66] but slightly bigger nonsignificant decreases in myside sharing among Republicans [congruence × condition: *ß* = −0.12 (−0.25, 0.02), *P* = 0.086] than among Democrats [*ß* = −0.08 (−0.20, 0.04), *P* = 0.19].

**Fig. 4.**
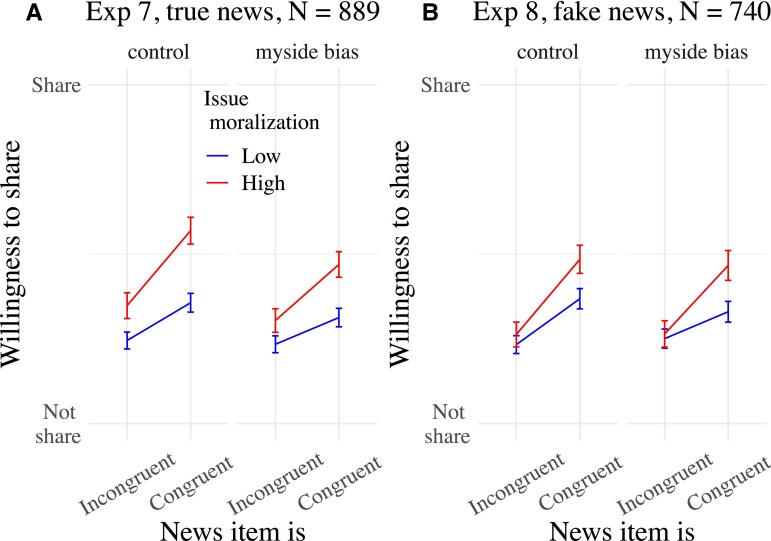
Mean willingness to share news items as a function of news congruence, issue moralization, and experimental condition in Experiments 7 (A) and 8 (B). Both experiments manipulated whether participants were exposed (myside bias) or not (control) to an intervention message on the myside bias. Only the congruence × issue moralization interactions are represented. Means are surrounded by 95% CIs. The plot of Experiment 8 does contain the two hostile fake abortion items markedly less subject to myside sharing in Experiment 4.

See Fig. 4 and online supplementary material, K and L, for regression tables.

### Discussion of Experiments 7 and 8

Issue moralization and attitude extremism again magnified respondents’ tendency to do myside sharing. The amplification of myside sharing by moralization occurred above and beyond that of attitude extremity. As to our intervention message focusing participants’ attention on an undesirable inclination to succumb to myside bias when deciding what to share, it slightly reduced overall sharing of true political news, as well as myside sharing of both true and fake news.

## Experiments 9 and 10: interactive intervention message on myside sharing—true and fake news

Experiments 7 and 8 warned participants against their propensity to *process* political information in ways that are biased toward their values and goals (the myside bias). In contrast, the educational message of Experiments 9 and 10—the latter run on entirely new items—warned against our tendency to *share* misinformation when it fits one's political goals, at a potential cost for one's epistemic reputation (23). Importantly, the interventions also contained an interactive phase whereby respondents had to actively assess whether each item was politically congruent and of absolute moral importance to them prior to reporting their sharing intentions. To our knowledge, this type of interactive design had not been tested in the literature on interventions against misinformation spread. Our expectation was that by focusing attention on the link between properties of the news items and the warning message, myside sharing of political news could be more efficiently reduced.

### Method

#### Participants and design

Experiment 9 was preregistered at https://osf.io/vyu7f (see online supplementary material, B) and run in November 2021. Our power analysis suggested 800 participants were needed to detect a small effect at 80% power. 806 respondents, representative of the US population on age, ethnicity, and sex, were recruited on Prolific, and none were removed from the data (*M*_age_ = 35, *SD*_age_ = 14, 74% women). Experiment 9 randomly exposed participants to the same six fake political news items as Experiments 5b and 6b (gun control, racial equality, and gender equality).

In contrast, and in order to gauge the robustness of our findings, Experiment 10 employed *22 entirely novel items*: 10 fake partisan, 10 true partisan, and 2 true neutral items (see online supplementary material, O–R). Issues covered included abortion, gun control, racial equality, and gender equality as well as a new issue: immigration. Experiment 10 was run in December 2022 and preregistered at https://osf.io/4xwha. A total of 1,063 US respondents were recruited on Prolific for Experiment 10, and 98 were removed from the data because they failed one of the two attention checks, leaving 965 participants (*M*_age_ = 41, *SD*_age_ = 14.5, 49% women).

In the intervention message condition of both studies, respondents read an intervention message about the reputational risks of sharing politically congruent misinformation (“myside” sharing) and then rated each item on dimensions that the message highlighted as potential sources of bias, before reporting their intentions to share. In the control condition, participants read a placebo message and then merely reported their intentions to share without rating the news stories.

#### Materials and procedure

Experiments 9 and 10 were inspired from Experiments 7 and 8 but differed in the following respects. All moral and political attitudes measures were taken before allocation to an experimental condition rather than after viewing the news to avoid post-treatment bias. The control condition presented participants with a placebo text about recent discoveries in archeology on child labor in prehistory (see online supplementary material, K and L). Its length and structure were matched to the intervention message (e.g. it also cited a scientific source).

The intervention message, in contrast, highlighted the following psychological facts: (i) that sharing misinformation can damage one's epistemic reputation and that (ii) one is most at risk of sharing misinformation when it fits one's political goals, in particular on issues (iii) on which one has an extreme position or that (iv) one regards as being of absolute moral importance. The message was inspired from work by Altay, Hacquin, and Mercier (23), by Kahan (7), and by our own findings in this project (not cited). Below is the verbatim of the intervention message (emphasis appeared in bold):

*Please read very carefully the following message. Questions will be asked to you about it*.

Science has shown that most people—both Democrats and Republicans—highly value accuracy and do not intentionally share false information online. This is because *spreading misinformation can damage your reputation* by making you look unreliable, gullible, or foolish.

However, the risk of believing and sharing falsehoods is greatest when the information *fits one's political goals*. When a piece of news tells us “what we want to hear” politically, for instance, we tend to *endorse it too readily—even when it is actually false*.

Importantly, one tends to think least critically on the issues on which one has an *extreme position*, and that one sees as being of *absolute moral importance*, i.e. that touch on one's core convictions and identity.

Source: Altay et al. (23); Kahan (7).

Participants merely reported their intentions to share each story in the control condition: “How likely would you be to share this news story on social media?” [(0) “Very unlikely,” (1) “Unlikely,” (2) “Likely,” and (3) “Very likely”]. In the intervention condition, however, they were first asked to reflect on two properties of each item, previously highlighted by the message, that could potentially favor gullibility and unwarranted sharing: “To what extent does this news story fit your political goals, or tell you ‘what you want to hear?’” [(0) “Not at all,” (1) “Somewhat not,” (2) “Clearly,” and (3) “Very much”] and “To what extent does this news story touch on an issue on which you have an extreme position, or that is of absolute moral importance to you?” [(0) “Very unlikely,” (1) “Unlikely,” (2) “Likely,” and (3) “Very likely”]. Note that our intervention intentionally differed in two respects (message + interactive task) from the control condition, in order to maximize impact. Experiment 10 also contained an attention check to filter out respondents not capable of recollecting the substance of the intervention message. Finally, Experiment 10 (but not Experiment 9) employed an improved measurement of issue moralization, which highlighted trade-off insensitivity, a core component of moralization, more than in previous operationalizations. See online supplementary material, S, for exact phrasing.

### Results

Replicating prior findings, respondents reported greater willingness to share congruent fake news in Experiment 9 [*ß* = 0.47 (0.40, 0.53), *P* < 0.001] and Experiment 10 [*ß* = 0.55 (0.51, 0.60), *P* < 0.001], as well as congruent true news in Experiment 10 [*ß* = 0.48 (0.43, 0.52), *P* < 0.001].

There were also positive main effects of issue moralization on fake news in Experiment 9 [*ß* = 0.17 (0.12, 0.22), *P* < 0.001] and Experiment 10 [*ß* = 0.18 (0.14, 0.21), *P* < 0.001] and on true news in Experiment 10 [*ß* = 0.16 (0.12, 0.20), *P* < 0.001]. Small positive main effects of attitude extremity were also observed on fake news in Experiment 9 [*ß* = 0.04 (0.01, 0.07), *P* = 0.005] and Experiment 10 [*ß* = 0.06 (0.04, 0.08), *P* < 0.001] and on true news in Experiment 10 [*ß* = 0.04 (0.02, 0.06), *P* < 0.001].

In line with previous trials, myside sharing was amplified by issue moralization (congruence × issue moralization). This was the case on fake news in Experiment 9 [*ß* = 0.32 (0.23, 0.41), *P* < 0.001] and Experiment 10 [*ß* = 0.28 (0.22, 0.35), *P* < 0.001], as well as on true news in Experiment 10 [*ß* = 0.25 (0.19, 0.32), *P* < 0.001]. Having an extreme attitude on the issue also exaggerated myside sharing (congruence × attitude extremity) of fake news in Experiment 9 [*ß* = 0.22 (0.18, 0.26), *P* < 0.001] and Experiment 10 [*ß* = 0.24 (0.21, 0.27), *P* < 0.001] as well as of true news in Experiment 10 [*ß* = 0.18 (0.15, 0.21), *P* < 0.001].

When including the two interactions in the model, both issue moralization [congruence × issue moralization in Experiment 9: *ß* = 0.19 (0.10, 0.28), *P* < 0.001; in Experiment 10: *ß* = 0.10 (0.03, 0.17), *P* = 0.004] and attitude extremity [congruence × attitude extremity in Experiment 9: *ß* = 0.18 (0.13, 0.23), *P* < 0.001; in Experiment 10: *ß* = 0.22 (0.18, 0.25), *P* < 0.001] independently contributed to magnify myside sharing of fake news. The same phenomenon was found on true news as well in Experiment 10, with both issue moralization [congruence × issue moralization: *ß* = 0.13 (0.06, 0.20), *P* < 0.001] and attitude extremity [congruence × attitude extremity: *ß* = 0.15 (0.12, 0.19), *P* < 0.001] independently increasing myside sharing.

Attitude extremity had the additional effect of slightly decreasing sharing of incongruent fake news in Experiment 9 [*ß* = −0.06 (−0.09, −0.03), *P* < 0.001] and Experiment 10 [*ß* = −0.06 (−0.08, −0.03), *P* < 0.001], as well as of true news in Experiment 10 [*ß* = −0.04 (−0.07, −0.02), *P* < 0.001]. Issue moralization had no such negative influence on sharing of incongruent news; if anything, its influence was positive, albeit not significant.

As regards the effect of the intervention—combining a message highlighting the reputational hazards of sharing politically congruent falsehoods and an interactive rating task—its effects were to reduce news sharing in general, regardless of its ideological congruence. The intervention reduced sharing of partisan fake news regardless of political congruence [main effect of condition in Experiment 9: *ß* = −0.16 (−0.24, −0.07), *P* < 0.001; in Experiment 10: *ß* = −0.20 (−0.27, −0.12), *P* < 0.001]. It also decreased partisan true news sharing across the board [Experiment 10: *ß* = −0.17 (−0.24, −0.09), *P* < 0.001]. In contrast with the partisan news, the intervention had a much stronger decreasing effect on sharing of the true neutral news employed as control items in Experiment 10 [*ß* = −0.43 (−0.54, −0.33), *P* < 0.001].

The intervention also had a marginally significant decreasing effect on the size of myside sharing of fake news in Experiment 9 [congruence × condition: *ß* = −0.09 (−0.19, 0.00), *P* = 0.053], but myside sharing was no affected in Experiment 10 on either fake [*ß* = −0.01 (−0.10, 0.08)] or true news [*ß* = −0.02 (−0.10, 0.06)]. Let us specify also that in the longer Experiment 10, effects of the experimental intervention on overall sharing and on the size of myside sharing were not influenced by whether analyses were run on the full 22 items set or only the first 8 items viewed by participants (on which treatment effects could have been expected to be stronger). There was no congruence × moral covariates × condition interaction in either study.

Finally, we looked at possible influences of partisanship with post hoc analyses. Note that our studies were not designed to test effects of partisanship, so these tend to be underpowered. In both studies, myside sharing was significantly smaller among Republicans than Democrats [congruence × partisanship in Experiment 9 on fake news: *ß* = −0.33 (−0.45, −0.21), *P* < 0.001; in Experiment 10 on fake news: *ß* = −0.57 (−0.64, −0.50), *P* < 0.001; in Experiment 10 on true news: *ß* = −0.50 (−0.57, −0.43), *P* < 0.001]. In Experiment 9 on fake news, the intervention seemed to markedly reduce overall sharing among Democrats [*ß* = −0.18 (−0.29, −0.08), *P* < 0.001] but not among Republicans [*ß* = −0.02 (−0.22, 0.19), *P* = 0.88]. The intervention, however, seemed to reduce myside sharing of fake news to a slightly greater (but nonsignificant) extent among Republicans [congruence × condition: *ß* = −0.15 (−0.35, 0.05), *P* = 0.14] than among Democrats [congruence × condition: *ß* = −0.08 (−0.20, 0.04), *P* = 0.21]. In Experiment 10, restricted analyses suggested that the intervention reduced overall sharing slightly more among Democrats [true news: *ß* = −0.19 (−0.29, −0.09), *P* < 0.001; fake news: *ß* = −0.25 (−0.35, −0.15), *P* < 0.001] than among Republicans [true news: *ß* = −0.13 (−0.24, −0.02), *P* = 0.025; fake news: *ß* = −0.12 (−0.23, −0.01), *P* = 0.03]. Restricted analyses also suggested nonsignificant decreases in myside sharing of roughly the same size among both groups of partisans [congruence × condition among Dems on true news: *ß* = −0.06 (−0.16, 0.04), *P* = 0.26; on fake news: *ß* = −0.07 (−0.19, 0.06), *P* = 0.28; among Reps on true news: *ß* = 0.03 (−0.08, 0.14), *P* = 0.57; on fake news: *ß* = 0.06 (−0.04, 0.16), *P* = 0.25].

**Fig. 5.**
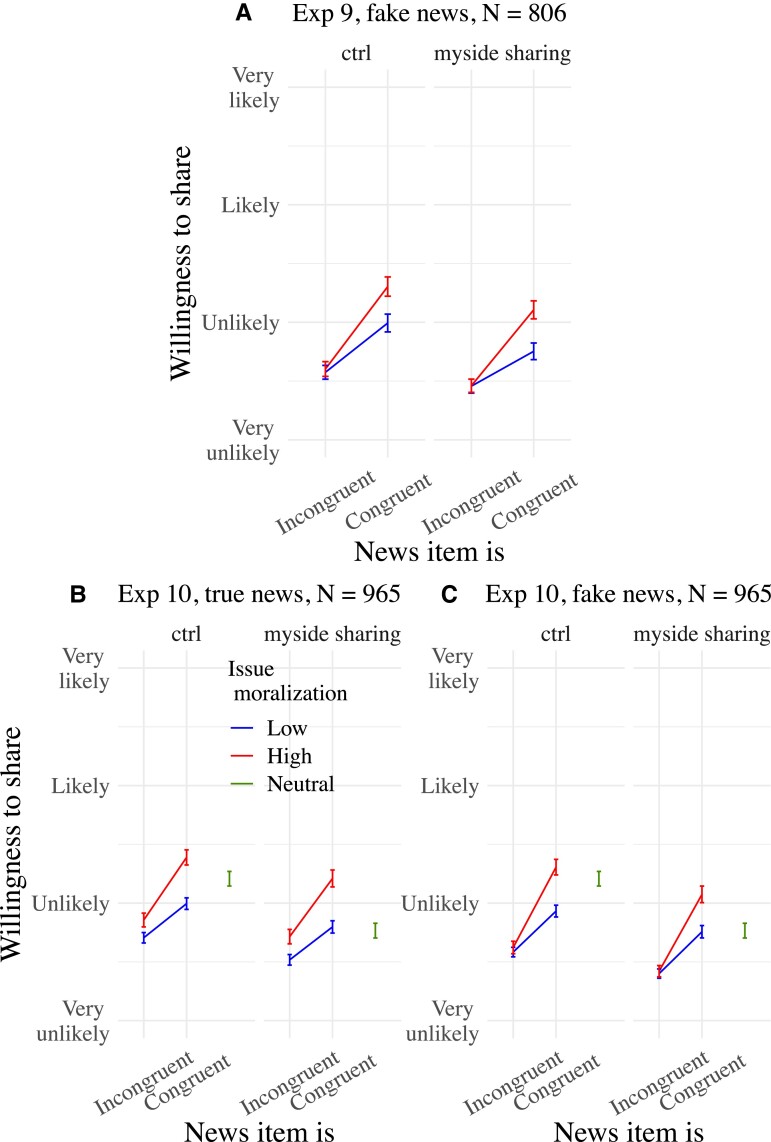
Mean willingness to share news items as a function of news congruence, issue moralization, and experimental condition in Experiments 9 (A) and 10 (B and C). Both experiments manipulated whether participants were exposed (myside sharing) or not (ctrl) to an intervention message on the reputational hazards of sharing congruent falsehoods combined with an interactive rating task. Only the congruence × issue moralization interactions are represented. Means are surrounded by 95% CIs.

See Fig. 5 for visualizations. Online supplementary material, M and N, contains regression tables of the analyses reported above from Experiments 9 and 10. Online supplementary material, N, also contain restricted analyses by issue showing that our basic sharing patterns tended to be observed on all five issues on both the true and fake news employed in Experiment 10.

### Discussion of Experiments 9 and 10

In line with prior findings, participants’ myside sharing was amplified by issue moralization and attitude extremity, and the magnifying effect of moralization was observed even when controlling for the influence of attitude extremity. Let us highlight that those effects were observed on two distinct sets of fake (Experiment 9) and true and fake news stories (Experiment 10). As regards our interactive intervention spotlighting the reputational risks of sharing politically congruent falsehoods, it yielded mixed results. It did slightly reduce overall sharing of both fake and true political news. However, it tended to fail to reduce the size of participants’ myside sharing of partisan news, with the exception of a small, marginally significant effect in Experiment 9. In contrast, the intervention's inhibiting effect on sharing was markedly stronger on true neutral news.

## General discussion

Across 12 experiments (*N* = 6,989), we explored US participants’ intentions to share true and fake partisan news on 5 controversial issues—gun control, abortion, racial equality, sex equality, and immigration—in social media contexts. Our experiments consistently show that people have a strong sharing preference for politically congruent news—Democrats even more so than Republicans. They also demonstrate that this “myside” sharing is magnified when respondents see the issue as being of “absolute moral importance”, and when they have an extreme attitude on the issue. Moreover, issue moralization was found to amplify myside sharing above and beyond attitude extremity in the majority of the studies. Expanding prior research on selective communication, our work provides a clear demonstration that citizens’ myside communicational preference is powerfully amplified by their moral and political ideology (18, 19, 39–43).

By examining this phenomenon across multiple experiments varying numerous parameters, we demonstrated the robustness of myside sharing and of its amplification by participants’ issue moralization and attitude extremity. First, those effects were consistently observed on both true (Experiments 1, 2, 3, 5a, 6a, 7, and 10) and fake (Experiments 4, 5b, 6b, 8, 9, and 10) news stories and across distinct operationalizations of our outcome variable. Moreover, myside sharing and its amplification by issue moralization and attitude extremity were systematically observed despite multiple manipulations of the sharing context. Namely, those effects were observed whether sharing was done from one's personal or an anonymous social media account (Experiments 5a and 5b), whether the audience was made of political friends or foes (Experiments 6a and 6b), and whether participants first saw intervention messages warning against the myside bias (Experiments 7 and 8), or an interactive intervention warning against the reputational costs of sharing mysided falsehoods (Experiments 9 and 10).

At the same time, our studies also make important contributions to the debate about interventions against misinformation in showing that sharing of strongly partisan true and fake news can be slightly moderated by a range of experimental manipulations. Imagining sharing news from a personal (rather than an anonymous) account did reduce fake news sharing among Republicans (but not Democrats; Experiment 5b). Imagining sharing the news to an uncongenial audience rather than like-minded people slightly decreased the size of myside sharing of both true and fake news (Experiments 6a and 6b). The intervention message warning against the myside bias (Experiments 7 and 8) also slightly reduced myside sharing of true and fake partisan news, as well as overall sharing of partisan true news (but unfortunately not of partisan fake news).

As regards the final intervention combining a message warning against the reputational cost of sharing congruent misinformation and an interactive task focusing attention on the news items’ fit to respondents’ political goals (Experiments 9 and 10), it had ambiguous effects. The intervention did reduce overall sharing of partisan true and fake news, which could be regarded as good news from the perspective of belief polarization. On the flip slide, however, the intervention tended to fail at reducing the size of myside sharing of both true and fake partisan news, and it also strongly reduced the sharing of true *neutral* news items, which is rather bad news from the standpoint of accurate information transmission (44). Those mixed findings clearly illustrate that interventions aiming to reduce the spread of politically slanted or problematic content should always include desirable content as well to assess potential negative side effects (45).

What causes selective sharing of strongly partisan news among our US respondents? One likely source is that participants share what they think is *true*, despite having different prior beliefs on many issues—a hypothesis corroborated by exploratory analyses of the motivations for sharing in Experiment 2 (presented in online supplementary material, E). From a Bayesian standpoint, the more a claim from an unknown source fits one's prior beliefs, the more it should be judged credible (16). To the extent, then, that congruent news stories were selected to fit participants’ priors more than incongruent ones, it is (subjectively) rational that they favor sharing congruent news—including when they happen to make entirely fabricated claims.

Regardless of the influence of prior beliefs, passing along claims that comfort politically congruent narratives at a higher rate than cross-cutting claims is also, of course, what the political rationality of coalitional management should incline us to do (46, 47). From a strategic perspective, sharing partisan news that points to a credible threat or outrageous event can help in mobilizing one's group in support of a loved cause or against an enemy, or in signaling political allegiances. News that highlights victories for one's political side can fulfill similar functions. In this respect, issue moralization and attitude extremity may potentially operate as amplifiers of those coalitional instincts—although our studies do not provide a direct demonstration of this (20, 21, 33).

While selective communication of strongly partisan news may be subjectively rational, it can be socially harmful in the aggregate. First, it means that fake, partial, and misleading information may travel far and wide among networks of like-minded peers so long as it surfs on preconceived notions and political interests widely shared within the networks. As long as clicks and shares translate into revenue and attention, news producers and politicians are incentivized to select and format what they say and write so as to court and galvanize their audiences.

If it combines with people's tendency to surround themselves with like-minded others, selective communication of political claims can deepen discrepancies in partisan perceptions of social reality—a fundamental concern that motivated this project. By making voters and politicians on one side routinely appear so ill-informed or extremist to the other side that their ignorance must be attributed to stupidity, ignorance, or hypocrisy, partisan divides in perceptions of facts can in turn fuel affective polarization (22, 48). This can only worsen our already fragile ability to open ourselves to alternative points of view, converge on nuanced accounts of complex issues, and avoid legislative gridlocks.

## Supplementary Material

pgad078_Supplementary_Data

## Data Availability

All data and R scripts are available on OSF at https://osf.io/5v8fw/.

## Footnotes

1 In Experiments 4 and 8, the two fake abortion items were markedly less subject to myside sharing from respondents for whom they are politically congruent, and this myside sharing was not as much amplified by issue moralization and attitude extremity than with the other items in Experiments 4 and 8 (see online supplementary material, L). Abortion as a topic was *not* the problem: by issue analyses of sharing patterns of the true news used in Experiments 1–3 (online supplementary material, G) and Experiment 7 (online supplementary material, K), as well as of a second batch of true and fake news items specifically selected for Experiment 10 (online supplementary material, N), did reveal myside sharing and its amplification by issue moralization and attitude extremity on items touching on abortion. We are not sure why the two Sanders and Pence fake abortion items were less subject to myside sharing than the others in Experiments 4 and 8. Independent raters’ perceptions do not suggest they were perceived as significantly less plausible than the other fake items (see online supplementary material, Q). Their deviation from the general trends may be due to the fact that they are focused on particular politicians and ascribe them such preposterous statements that even supporters of the side they promote found them hostile and were thus more reluctant to share them.

2 We acknowledge that those proposed policies primarily focus on reducing online hostility rather than reducing myside sharing. Nevertheless, we consider it relevant to ask what impact they may have on the sharing of politically slanted but not necessarily hostile news (in particular fake news stories).
