# Correction to: Moralization and extremism robustly amplify myside sharing

**DOI:** 10.1093/pnasnexus/pgad193

**Published:** 2023-06-14

This is a correction to: Antoine Marie and others, Moralization and extremism robustly amplify myside sharing, PNAS Nexus, Volume 2, Issue 4, April 2023, pgad078, https://doi.org/10.1093/pnasnexus/pgad078

In the originally published version of this manuscript, the following sentence in the legend of Figure 2 was incorrect:

Mean willingness to share news items as a function of news congruence and issue moralization (left) and news congruence and attitude extremity (right) in Experiments 1 to 4.

This sentence has been corrected to read as follows:

Mean willingness to share news items as a function of news congruence and issue moralization (top) and news congruence and attitude extremity (bottom) in Experiments 1 to 4.

