# Correction to: Moralization and extremism robustly amplify myside sharing

**DOI:** 10.1093/pnasnexus/pgad271

**Published:** 2023-08-22

This is a correction to: Antoine Marie and others, Moralization and extremism robustly amplify myside sharing, PNAS Nexus, Volume 2, Issue 4, April 2023, pgad078, https://doi.org/10.1093/pnasnexus/pgad078

In the originally published version of this manuscript, the following Ethics Approval Statement was inadvertently omitted:

All participants included in the analyses gave their consent to participate in the surveys. IRB approval number 20152900001072 was given to our studies by the Comité d’éthique de la recherche en santé (CERES) from Université Paris Descartes.

This error has been corrected.

