# Supplementary material for: Moralization and extremism robustly amplify myside sharing
Source: PNAS Nexus. 2023 Apr 10;2(4):pgad078. doi: 10.1093/pnasnexus/pgad078 (PMC10129063; doi:10.1093/pnasnexus/pgad078)
Supplement: pgad078_Supplementary_Data [file pgad078_supplementary_data.zip › PNASNEXUS-PNASNEXUS-2022-00660-s01.pdf]

**A** *True partisan news*

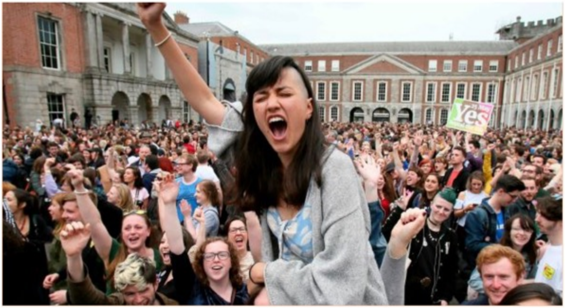


## Abortion will soon become cost-free in Ireland

Until recently, abortion was illegal in Ireland. However, according to Health Minister Simon Harris, a new law is about to be submitted to the legislature in October proposing to make abortion cost-free...

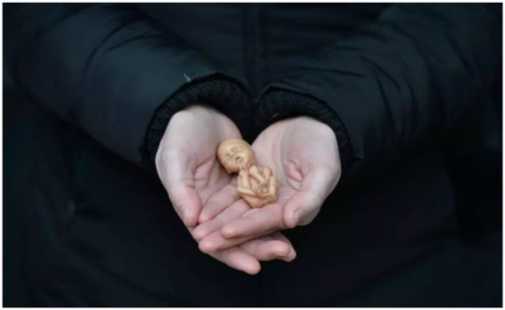

## New evidence suggests a majority of abortion cases are on-demand and do not involve rape victims

One of the most common reasons given to justify abortion is that it may allow mothers to postpone childbearing to a more suitable time and provide already born children with more care and resources...

**B** *Fake partisan news*

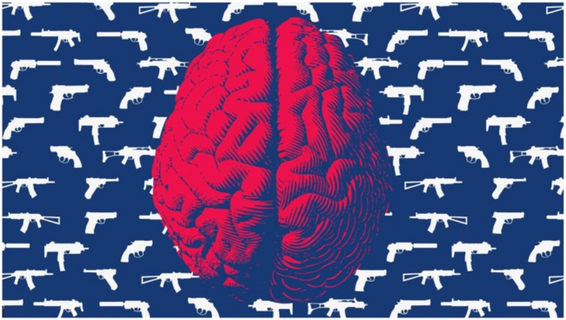

## Republicans voted to allow people with pre-existing mental illness to buy firearms

Republican party Representatives voted to allow people with pre-existing mental illness to buy firearms, yet denied them health insurance to treat their pre-existing mental illness…

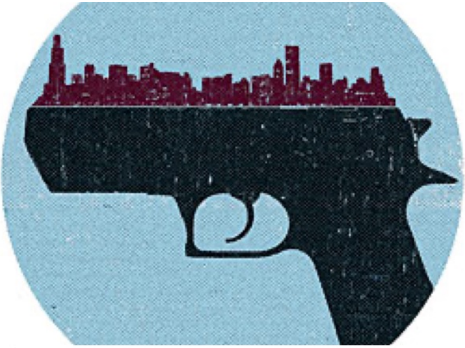

## Chicago Shootings Prove That Gun Control Doesn't Reduce Crime

Chicago has the strongest gun laws in the U.S., and yet the total number of murders per inhabitant in Chicago is the highest in the country…
