# Supplementary material for: Moralization and extremism robustly amplify myside sharing
Source: PNAS Nexus. 2023 Apr 10;2(4):pgad078. doi: 10.1093/pnasnexus/pgad078 (PMC10129063; doi:10.1093/pnasnexus/pgad078)
Supplement: pgad078_Supplementary_Data [file pgad078_supplementary_data.zip › SM_April3.docx]

**Moralization and Extremism Robustly Amplify Myside Sharing**

**Marie, Altay & Strickland 2023**

**Supplementary Materials**

All data and R scripts are available on OSF at: <https://osf.io/5v8fw/>.

Table of Contents

[A. Choice of the polarizing issues 45](#_Toc127004189)

[B. Pre-registrations 45](#_Toc127004190)

[C. Coding of predictors in regression tables 46](#_Toc127004191)

[D. Experiment 1: Willingness to share 4-point, true news 48](#_Toc127004192)

[Congruence x Issue moralization 48](#_Toc127004193)

[Congruence x Attitude extremity 49](#_Toc127004194)

[Congruence x Issue moralization controlling for Congruence x Attitude extremity 50](#_Toc127004195)

[Effects of partisanship 51](#_Toc127004196)

[E. Experiment 2: Willingness to share 4-point, true news 53](#_Toc127004197)

[Congruence x Issue moralization 53](#_Toc127004198)

[Congruence x Attitude extremity 54](#_Toc127004199)

[Congruence x Issue moralization controlling for Congruence x Attitude extremity 55](#_Toc127004200)

[Effects of partisanship 55](#_Toc127004201)

[Exploratory analyses of motivations to share 58](#_Toc127004202)

[Motivations to share: Accuracy 59](#_Toc127004203)

[Motivations to share: Usefulness for one’s political commitments 60](#_Toc127004204)

[Motivations to share: Informativeness 62](#_Toc127004205)

[Regression analysis of willingness to share true news on each motivation to share 64](#_Toc127004206)

[F. Experiment 3: Willingness to share 2-point, true news 70](#_Toc127004207)

[Congruence x Issue moralization 70](#_Toc127004208)

[Congruence x Attitude extremity 71](#_Toc127004209)

[Congruence x Issue moralization controlling for Congruence x Attitude extremity 72](#_Toc127004210)

[Effects of partisanship 73](#_Toc127004211)

[G. Pooled Experiments 1-3, true news 74](#_Toc127004212)

[Congruence x issue moralization, Congruence x attitude extremity, Congruence x issue moralization controlling for Congruence x attitude extremity 74](#_Toc127004213)

[By issue analyses 76](#_Toc127004214)

[H. Experiment 4: Willingness to share 4-point, fake news 81](#_Toc127004215)

[By issue analyses 81](#_Toc127004216)

[Congruence x Issue moralization 86](#_Toc127004217)

[Congruence x Attitude extremity 88](#_Toc127004218)

[Congruence x Issue moralization controlling for Congruence x Attitude extremity 90](#_Toc127004219)

[Effects of partisanship 92](#_Toc127004220)

[I. Experiment 5a & 5b: Sharing from anonymous vs. personal social media account 94](#_Toc127004221)

[Pilot Experiment 5a (true news) 94](#_Toc127004222)

[Materials and procedure 94](#_Toc127004223)

[Congruence x Issue moralization 97](#_Toc127004224)

[Congruence x Attitude extremity 98](#_Toc127004225)

[Congruence x Issue moralization controlling for Congruence x Attitude extremity 99](#_Toc127004226)

[Congruence x Account 100](#_Toc127004227)

[Congruence x Account on Democrats only (restricted analyses) 101](#_Toc127004228)

[Congruence x Account on Republicans only (restricted analyses) 102](#_Toc127004229)

[Congruence x Issue moralization x Account 102](#_Toc127004230)

[Congruence x Attitude extremity x Account 104](#_Toc127004231)

[Final Experiment 5b (fake news) 106](#_Toc127004232)

[Manipulation check 106](#_Toc127004233)

[Congruence x Issue moralization 106](#_Toc127004234)

[Congruence x Attitude extremity 107](#_Toc127004235)

[Congruence x Issue moralization controlling for Congruence x Attitude extremity 108](#_Toc127004236)

[Congruence x Account 109](#_Toc127004237)

[Effects of partisanship 111](#_Toc127004238)

[Congruence x Account on Democrats only (restricted analyses) 113](#_Toc127004239)

[Congruence x Account on Republicans only (restricted analyses) 113](#_Toc127004240)

[Congruence x Issue moralization x Account 114](#_Toc127004241)

[Congruence x Attitude extremity x Account 115](#_Toc127004242)

[J. Experiment 6a & 6b: Sharing to like-minded friends vs. foes 118](#_Toc127004243)

[Pilot Experiment 6a (true news) 118](#_Toc127004244)

[Congruence x Issue moralization 118](#_Toc127004245)

[Congruence x Attitude extremity 119](#_Toc127004246)

[Congruence x Issue moralization controlling for Congruence x Attitude extremity 120](#_Toc127004247)

[Congruence x Audience 121](#_Toc127004248)

[Congruence x Audience on Democrats only (restricted analyses) 122](#_Toc127004249)

[Congruence x Audience on Republicans only (restricted analyses) 122](#_Toc127004250)

[Congruence x Issue moralization x Audience 123](#_Toc127004251)

[Congruence x Attitude extremity x Audience 125](#_Toc127004252)

[Final Experiment 6b (fake news) 127](#_Toc127004253)

[Manipulation check 127](#_Toc127004254)

[Congruence x Issue moralization 127](#_Toc127004255)

[Congruence x Attitude extremity 128](#_Toc127004256)

[Congruence x Issue moralization controlling for Congruence x Attitude extremity 129](#_Toc127004257)

[Congruence x Audience 130](#_Toc127004258)

[Effects of partisanship 132](#_Toc127004259)

[Congruence x Audience on Democrats only (restricted analyses) 133](#_Toc127004260)

[Congruence x Audience on Republicans only (restricted analyses) 134](#_Toc127004261)

[Congruence x Issue moralization x Audience 134](#_Toc127004262)

[Congruence x Attitude extremity x Audience 136](#_Toc127004263)

[K. Experiment 7: Intervention message on the myside bias, true news 138](#_Toc127004264)

[Congruence x Issue moralization 138](#_Toc127004265)

[Congruence x Attitude extremity 139](#_Toc127004266)

[Congruence x Issue moralization controlling for Congruence x Attitude extremity 140](#_Toc127004267)

[By issue analyses 141](#_Toc127004268)

[Congruence x Condition 144](#_Toc127004269)

[Effects of partisanship 145](#_Toc127004270)

[Congruence x Condition on Democrats only (restricted analyses) 146](#_Toc127004271)

[Congruence x Condition on Republicans only (restricted analyses) 146](#_Toc127004272)

[Congruence x Issue moralization x Condition 147](#_Toc127004273)

[Congruence x Attitude extremity x Condition 149](#_Toc127004274)

[L. Experiment 8: Intervention message on the myside bias, fake news 151](#_Toc127004275)

[Congruence x Issue moralization 151](#_Toc127004276)

[Congruence x Attitude extremity 153](#_Toc127004277)

[Congruence x Issue moralization controlling for Congruence x Attitude extremity 155](#_Toc127004278)

[By issue analyses 158](#_Toc127004279)

[Congruence x Condition 162](#_Toc127004280)

[Effects of partisanship 163](#_Toc127004281)

[Congruence x Condition on Democrats only (restricted analyses) 165](#_Toc127004282)

[Congruence x Condition on Republicans only (restricted analyses) 165](#_Toc127004283)

[Congruence x Issue moralization x Condition 166](#_Toc127004284)

[Congruence x Attitude extremity x Condition 168](#_Toc127004285)

[M. Experiment 9: Interactive intervention on the reputational risk of myside sharing, fake news 170](#_Toc127004286)

[Message in the control condition 170](#_Toc127004287)

[Congruence x Issue moralization 170](#_Toc127004288)

[Congruence x Attitude extremity 171](#_Toc127004289)

[Congruence x Issue moralization controlling for Congruence x Attitude extremity 172](#_Toc127004290)

[Congruence x Condition 174](#_Toc127004291)

[Effects of partisanship 175](#_Toc127004292)

[Congruent x Condition on Democrats only (restricted analysis) 177](#_Toc127004293)

[Congruent x Condition on Republicans only (restricted analysis) 178](#_Toc127004294)

[Congruence x Issue moralization x Condition 179](#_Toc127004295)

[Congruence x Attitude extremity x Condition 180](#_Toc127004296)

[N. Experiment 10: Interactive intervention on the reputational risk of myside sharing, true and fake news 182](#_Toc127004297)

[Message in the control condition 182](#_Toc127004298)

[Congruence x Issue moralization, Congruence x Attitude extremity, Congruence x Issue moralization controlling for Congruence x Attitude extremity 182](#_Toc127004299)

[Congruence x Issue moralization, Congruence x Attitude extremity, Congruence x Issue moralization controlling for Congruence x Attitude extremity on 8 first items viewed 185](#_Toc127004300)

[By issue analyses 188](#_Toc127004301)

[Congruence x Condition 202](#_Toc127004302)

[Congruence x Condition on 8 first items viewed only 203](#_Toc127004303)

[Condition on true neutral items 205](#_Toc127004304)

[Effects of partisanship 206](#_Toc127004305)

[Congruence x Condition on Democrats only (restricted analyses) 210](#_Toc127004306)

[Congruence x Condition on Republicans only (restricted analyses) 211](#_Toc127004307)

[Congruence x Issue moralization x Condition 213](#_Toc127004308)

[Congruence x Attitude extremity x Condition 215](#_Toc127004309)

[O. New items selection questions 218](#_Toc127004310)

[Question to assess which side on each issue the news story was congruent to 218](#_Toc127004311)

[Accuracy calibration question used to select the fake news 218](#_Toc127004312)

[P. True partisan news items 219](#_Toc127004313)

[Experiments 1-9 219](#_Toc127004314)

[Raters’ perceptions of true political news items’ slant 228](#_Toc127004315)

[Experiment 10 229](#_Toc127004316)

[Raters’ perceptions of true political news items’ slant 240](#_Toc127004317)

[Q. True neutral news items 245](#_Toc127004318)

[Experiments 1-9 245](#_Toc127004319)

[Experiment 10 249](#_Toc127004320)

[R. Fake partisan news items 251](#_Toc127004321)

[Experiments 1-9 251](#_Toc127004322)

[Raters’ perceptions of fake political news items’ slant 259](#_Toc127004323)

[Experiment 10 264](#_Toc127004324)

[Raters’ perceptions of fake political news items’ slant 274](#_Toc127004325)

[S. Moral and political attitudes questions 275](#_Toc127004326)

[Attitude on each issue (continuous) 275](#_Toc127004327)

[Moral importance of issue (dichotomous) 276](#_Toc127004328)

[T. Attention check ‘videogame’ used in all experiments 277](#_Toc127004329)

# Choice of the polarizing issues

The 4 polarizing issues retained for the partisan news items were chosen based on responses to the questions “*I could never vote for a politician if they supported* …” and “*I could never vote for a politician if they failed to support*…” in a norming study in 2019. These questions enabled us to know which topics U.S. MTurkers judged as being most morally important, and on which they were most likely to be polarized. *Gun Control, Racial Equality, Gender Equality* and *Abortion* were the topics that came up most frequently and which were retained in Experiments 1-9. In Experiment 10, *Immigration* was added to the list of issues.

# Pre-registrations

**Experiment 1 and 3: basic design (true news)**

<https://aspredicted.org/blind.php?x=9bw9ww>.

**Experiment 4: basic design (fake news)**

<https://osf.io/wrd5y>

**Experiment 5b, Sharing from Anonymous vs. Personal account (fake news)**

<https://osf.io/3jgea>

**Experiment 6b, Sharing to Like-Minded Friends vs. Foes (fake news)**

<https://osf.io/tjeh4>

**Experiment 9, Intervention Message on Reputation and Myside sharing (fake news)**

<https://osf.io/vyu7f>

**Experiment 10, Intervention Message on Reputation and Myside sharing (true and fake news)**

<https://osf.io/4xwha>

# Coding of predictors in regression tables

In regression analyses, predictor codes mean the following:

NB: baselines provided in parentheses “( )”.

*Experiments 1-10*

Congruent TRUE = New item is politically congruent (*vs*. incongruent) to the participant’s attitude on the issue (dichotomous)

Congruent NA = New item is true neutral, non-political (*vs*. incongruent; dichotomous)

AbsImpIssue TRUE = The issue covers by the news item is of high (“absolute”) moral importance (*vs*. low importance; dichotomous)

Attit Issue extr: High attitude extremity on the issue (*vs.* low, continuous)

PO_bin, Pol.Orient_bin: Partisanship, i.e., Republican (*vs.* Democrat)

*Experiment 5a and 5b*

Account [Personal] = Sharing from personal account condition (vs. from anonymous account)

*Experiment 6a*

Audience [Disagree]: Condition in which imagined audience is described as disagreeing with the participant (vs. Agree)

*Experiment 6b*

Audience [opposite position]: Condition in which imagined audience is described as having an opposite position on the issue from the participant (vs. same position)

*Experiments 7, 8*

Condition Message = Intervention message on myside bias (vs. control, no message)

*Experiments 9, 10*

Condition Message = Intervention message on the reputational costs of myside sharing of misinformation + interactive rating task (vs. neutral message)

# Experiment 1: Willingness to share 4-point, true news

### Congruence x Issue moralization

| **Experiment 1** | | | | | |
| --- | --- | --- | --- | --- | --- |
|  | **sharing** | | **sharing** | | |
| *Predictors* | *ß* | *p* | *ß* | *p* | *std. p* |
| Congruent [TRUE] | 0.35 (0.27,0.42) | **<0.001** | 0.24 (0.16,0.33) | **<0.001** | **<0.001** |
| Congruent [NA] | 0.36 (0.19,0.54) | **<0.001** | 0.31 (0.12,0.50) | **0.002** | **0.002** |
| AbsImpIssue [TRUE] | 0.17 (0.10,0.24) | **<0.001** | -0.01 (-0.10,0.09) | 0.865 | 0.865 |
| edu | 0.07 (0.00,0.14) | **0.048** | 0.07 (0.00,0.14) | **0.048** | **0.048** |
| sex [female] | 0.11 (-0.03,0.26) | 0.128 | 0.11 (-0.03,0.25) | 0.130 | 0.130 |
| age | -0.14 (-0.21, -0.07) | **<0.001** | -0.14 (-0.21, -0.07) | **<0.001** | **<0.001** |
| Congruent [TRUE] * AbsImpIssue [TRUE] |  |  | 0.35 (0.22,0.48) | **<0.001** | **<0.001** |
| **Random Effects** | | | | | |
| σ^2^ | 0.51 | | 0.50 | | |
| τ_00_ | 0.45 _id_ | | 0.45 _id_ | | |
|  | 0.02 _News_ | | 0.02 _News_ | | |
| τ_11_ | 0.07 _id.CongruentTRUE_ | | 0.07 _id.CongruentTRUE_ | | |
|  | 0.04 _id.CongruentNA_ | | 0.00 _id.CongruentNA_ | | |
|  | 0.02 _id.AbsImpIssueTRUE_ | | 0.02 _id.AbsImpIssueTRUE_ | | |
|  | 0.32 _id.AbsImpIssueNA_ | | 0.19 _id.AbsImpIssueNA_ | | |
| ρ_01_ | -0.34 _id.CongruentTRUE_ | | -0.33 _id.CongruentTRUE_ | | |
|  | 0.10 _id.CongruentNA_ | | -0.92 _id.CongruentNA_ | | |
|  | -0.35 _id.AbsImpIssueTRUE_ | | -0.32 _id.AbsImpIssueTRUE_ | | |
|  | -0.40 _id.AbsImpIssueNA_ | | -0.31 _id.AbsImpIssueNA_ | | |
| ICC | 0.48 | | 0.45 | | |
| Marginal R^2^ / Conditional R^2^ | 0.053 / 0.504 | | 0.061 / 0.486 | | |

### Congruence x Attitude extremity

| **Experiment 1 - true news** | | | | | |
| --- | --- | --- | --- | --- | --- |
|  | **sharing** | | **sharing** | | |
| *Predictors* | *ß* | *p* | *ß* | *p* | *std. p* |
| Congruent [TRUE] | 0.35 (0.28,0.42) | **<0.001** | 0.35 (0.28,0.42) | 0.685 | **<0.001** |
| Attit Issue extr | 0.03 (-0.01,0.07) | 0.095 | -0.04 (-0.09,0.00) | 0.071 | 0.071 |
| edu | 0.10 (0.02,0.17) | **0.017** | 0.09 (0.02,0.17) | **0.019** | **0.019** |
| sex [female] | 0.18 (0.03,0.34) | **0.020** | 0.18 (0.03,0.34) | **0.022** | **0.022** |
| age | -0.17 (-0.24, -0.09) | **<0.001** | -0.17 (-0.25, -0.09) | **<0.001** | **<0.001** |
| Congruent [TRUE] * Attit Issue extr |  |  | 0.15 (0.09,0.21) | **<0.001** | **<0.001** |
| **Random Effects** | | | | | |
| σ^2^ | 0.48 | | 0.48 | | |
| τ_00_ | 0.45 _id_ | | 0.43 _id_ | | |
|  | 0.02 _News_ | | 0.02 _News_ | | |
| τ_11_ | 0.09 _id.CongruentTRUE_ | | 0.08 _id.CongruentTRUE_ | | |
|  | 0.07 _id.Attit_Issue_extr_ | | 0.07 _id.Attit_Issue_extr_ | | |
| ρ_01_ | -0.46 _id.CongruentTRUE_ | | -0.40 _id.CongruentTRUE_ | | |
|  | -0.14 _id.Attit_Issue_extr_ | | -0.13 _id.Attit_Issue_extr_ | | |
| ICC | 0.48 | | 0.48 | | |
| Marginal R^2^ / Conditional R^2^ | 0.071 / 0.517 | | 0.077 / 0.521 | | |

### Congruence x Issue moralization controlling for Congruence x Attitude extremity

| **Experiment 1 - true news** | | | |
| --- | --- | --- | --- |
|  | **sharing** | | |
| *Predictors* | *ß* | *p* | *std. p* |
| Congruent [TRUE] | 0.27 (0.18,0.35) | 0.578 | **<0.001** |
| AbsImpIssue [TRUE] | 0.04 (-0.06,0.14) | 0.473 | 0.473 |
| Attit Issue extr | -0.06 (-0.11, -0.01) | **0.022** | **0.022** |
| edu | 0.08 (0.01,0.16) | **0.032** | **0.032** |
| sex [female] | 0.16 (0.01,0.32) | **0.039** | **0.039** |
| age | -0.16 (-0.23, -0.08) | **<0.001** | **<0.001** |
| Congruent [TRUE] * AbsImpIssue [TRUE] | 0.28 (0.14,0.41) | **<0.001** | **<0.001** |
| Congruent [TRUE] * Attit Issue extr | 0.11 (0.04,0.17) | **0.001** | **0.001** |
| **Random Effects** | | | |
| σ^2^ | 0.47 | | |
| τ_00_ _id_ | 0.46 | | |
| τ_00_ _News_ | 0.03 | | |
| τ_11_ _id.CongruentTRUE_ | 0.08 | | |
| τ_11_ _id.AbsImpIssueTRUE_ | 0.04 | | |
| ρ_01_ _id.CongruentTRUE_ | -0.33 | | |
| ρ_01_ _id.AbsImpIssueTRUE_ | -0.31 | | |
| ICC | 0.49 | | |
| Marginal R^2^ / Conditional R^2^ | 0.084 / 0.533 | | |

### Effects of partisanship

| **Experiment 1 - true news** | | | | | | | | | |
| --- | --- | --- | --- | --- | --- | --- | --- | --- | --- |
|  | **SHARING** | | **SHARING** | | **SHARING** | | **SHARING** | | |
| *Predictors* | *ß* | *p* | *ß* | *p* | *ß* | *p* | *ß* | *p* | *std. p* |
| Congruent [TRUE] | 0.35 (0.27,0.42) | **<0.001** | 0.38 (0.28,0.48) | **<0.001** | 0.24 (0.13,0.36) | **<0.001** | 0.37 (0.27,0.47) | 0.723 | **<0.001** |
| Congruent [NA] | 0.31 (0.13,0.48) | **0.001** | 0.43 (0.24,0.61) | **<0.001** | 0.44 (0.23,0.64) | **<0.001** |  |  |  |
| edu | 0.08 (0.01,0.15) | **0.033** | 0.08 (0.01,0.15) | **0.027** | 0.08 (0.01,0.15) | **0.028** | 0.08 (0.00,0.15) | **0.047** | **0.047** |
| sex [female] | 0.12 (-0.02,0.27) | 0.093 | 0.12 (-0.02,0.26) | 0.105 | 0.11 (-0.03,0.25) | 0.122 | 0.15 (-0.00,0.30) | 0.056 | 0.056 |
| age | -0.15 (-0.22, -0.07) | **<0.001** | -0.16 (-0.23, -0.09) | **<0.001** | -0.15 (-0.22, -0.09) | **<0.001** | -0.18 (-0.25, -0.10) | **<0.001** | **<0.001** |
| Pol Orient bin [Republican] |  |  | 0.38 (0.21,0.55) | **<0.001** | 0.39 (0.21,0.57) | **<0.001** | 0.37 (0.20,0.54) | **0.042** | **<0.001** |
| Congruent [TRUE] * Pol Orient bin [Republican] |  |  | -0.06 (-0.20,0.07) | 0.370 | 0.00 (-0.16,0.16) | 0.978 | -0.04 (-0.18,0.09) | 0.247 | 0.546 |
| Congruent [NA] * Pol Orient bin [Republican] |  |  | -0.27 (-0.42, -0.11) | **0.001** | -0.28 (-0.44, -0.11) | **0.001** |  |  |  |
| AbsImpIssue [TRUE] |  |  |  |  | -0.00 (-0.13,0.13) | 1.000 |  |  |  |
| Congruent [TRUE] * AbsImpIssue [TRUE] |  |  |  |  | 0.43 (0.26,0.60) | **<0.001** |  |  |  |
| AbsImpIssue [TRUE] * Pol Orient bin [Republican] |  |  |  |  | -0.01 (-0.20,0.18) | 0.904 |  |  |  |
| (Congruent [TRUE] * AbsImpIssue [TRUE]) * Pol Orient bin [Republican] |  |  |  |  | -0.19 (-0.45,0.06) | 0.138 |  |  |  |
| Attit Issue extr |  |  |  |  |  |  | -0.06 (-0.12, -0.00) | **0.045** | **0.045** |
| Congruent [TRUE] * Attit Issue extr |  |  |  |  |  |  | 0.19 (0.11,0.27) | **<0.001** | **<0.001** |
| Attit Issue extr * Pol Orient bin [Republican] |  |  |  |  |  |  | 0.05 (-0.05,0.15) | 0.333 | 0.333 |
| (Congruent [TRUE] * Attit Issue extr) * Pol Orient bin [Republican] |  |  |  |  |  |  | -0.10 (-0.23,0.02) | 0.117 | 0.117 |
| **Random Effects** | | | | | | | | | |
| σ^2^ | 0.51 | | 0.51 | | 0.51 | | 0.48 | | |
| τ_00_ | 0.43 _id_ | | 0.38 _id_ | | 0.38 _id_ | | 0.39 _id_ | | |
|  | 0.02 _News_ | | 0.02 _News_ | | 0.02 _News_ | | 0.02 _News_ | | |
| τ_11_ | 0.07 _id.CongruentTRUE_ | | 0.26 _id.Pol.Orient_binRepublican_ | | 0.23 _id.Pol.Orient_binRepublican_ | | 0.42 _id.Pol.Orient_binRepublican_ | | |
|  | 0.20 _id.CongruentNA_ | | 0.07 _id.CongruentTRUE_ | | 0.06 _id.CongruentTRUE_ | | 0.07 _id.CongruentTRUE_ | | |
|  |  | | 0.19 _id.CongruentNA_ | | 0.19 _id.CongruentNA_ | |  | | |
| ρ_01_ | -0.33 _id.CongruentTRUE_ | | -0.33 _id.Pol.Orient_binRepublican_ | | -0.30 _id.Pol.Orient_binRepublican_ | | -0.49 _id.Pol.Orient_binRepublican_ | | |
|  | -0.42 _id.CongruentNA_ | | -0.17 _id.CongruentTRUE_ | | -0.20 _id.CongruentTRUE_ | | -0.16 _id.CongruentTRUE_ | | |
|  |  | | -0.23 _id.CongruentNA_ | | -0.24 _id.CongruentNA_ | |  | | |
| ICC | 0.45 | | 0.44 | | 0.44 | | 0.46 | | |
| Marginal R^2^ / Conditional R^2^ | 0.053 / 0.477 | | 0.078 / 0.481 | | 0.088 / 0.492 | | 0.107 / 0.518 | | |

# Experiment 2: Willingness to share 4-point, true news

### Congruence x Issue moralization

| **Experiment 2 - true news** | | | | |
| --- | --- | --- | --- | --- |
|  | **sharing** | | **sharing** | |
| *Predictors* | *ß* | *p* | *ß* | *p* |
| Congruent [TRUE] | 0.28 (0.20,0.36) | **<0.001** | 0.21 (0.11,0.31) | **<0.001** |
| Congruent [NA] | 0.25 (0.08,0.43) | **0.004** | 0.22 (0.04,0.40) | **0.019** |
| AbsImpIssue [TRUE] | 0.14 (0.06,0.22) | **0.001** | 0.04 (-0.07,0.15) | 0.455 |
| edu | 0.22 (0.14,0.29) | **<0.001** | 0.22 (0.14,0.29) | **<0.001** |
| sex [female] | 0.05 (-0.10,0.20) | 0.488 | 0.05 (-0.10,0.20) | 0.501 |
| age | -0.16 (-0.24, -0.09) | **<0.001** | -0.16 (-0.24, -0.09) | **<0.001** |
| Congruent [TRUE] * AbsImpIssue [TRUE] |  |  | 0.20 (0.05,0.34) | **0.007** |
| **Random Effects** | | | | |
| σ^2^ | 0.47 | | 0.47 | |
| τ_00_ | 0.50 _id_ | | 0.50 _id_ | |
|  | 0.02 _News_ | | 0.02 _News_ | |
| τ_11_ | 0.03 _id.CongruentTRUE_ | | 0.03 _id.CongruentTRUE_ | |
|  | 0.10 _id.CongruentNA_ | | 0.02 _id.CongruentNA_ | |
|  | 0.00 _id.AbsImpIssueTRUE_ | | 0.00 _id.AbsImpIssueTRUE_ | |
|  | 0.06 _id.AbsImpIssueNA_ | | 0.05 _id.AbsImpIssueNA_ | |
| ρ_01_ | -0.34 _id.CongruentTRUE_ | | -0.33 _id.CongruentTRUE_ | |
|  | -0.32 _id.CongruentNA_ | | 0.26 _id.CongruentNA_ | |
|  | -0.59 _id.AbsImpIssueTRUE_ | | -0.56 _id.AbsImpIssueTRUE_ | |
|  | 0.23 _id.AbsImpIssueNA_ | | -0.37 _id.AbsImpIssueNA_ | |
| ICC |  | | 0.52 | |
| Marginal R^2^ / Conditional R^2^ | 0.154 / NA | | 0.083 / 0.562 | |

### Congruence x Attitude extremity

| **Experiment 2 - true news** | | | | | |
| --- | --- | --- | --- | --- | --- |
|  | **sharing** | | **sharing** | | |
| *Predictors* | *ß* | *p* | *ß* | *p* | *std. p* |
| Congruent [TRUE] | 0.29 (0.20,0.37) | **<0.001** | 0.29 (0.21,0.37) | 0.894 | **<0.001** |
| Attit Issue extr | 0.03 (-0.02,0.08) | 0.202 | -0.04 (-0.11,0.02) | 0.135 | 0.158 |
| edu | 0.23 (0.16,0.31) | **<0.001** | 0.23 (0.16,0.31) | **<0.001** | **<0.001** |
| sex [female] | 0.08 (-0.07,0.24) | 0.300 | 0.08 (-0.07,0.24) | 0.272 | 0.304 |
| age | -0.21 (-0.28, -0.13) | **<0.001** | -0.21 (-0.28, -0.13) | **<0.001** | **<0.001** |
| Congruent [TRUE] * Attit Issue extr |  |  | 0.15 (0.08,0.21) | **<0.001** | **<0.001** |
| **Random Effects** | | | | | |
| σ^2^ | 0.45 | | 0.46 | | |
| τ_00_ | 0.46 _id_ | | 0.38 _id_ | | |
|  | 0.02 _News_ | | 0.02 _News_ | | |
| τ_11_ | 0.05 _id.CongruentTRUE_ | | 0.00 _id.CongruentTRUE_ | | |
|  | 0.16 _id.Attit_Issue_extr_ | | 0.09 _id.Attit_Issue_extr_ | | |
| ρ_01_ | -0.62 _id.CongruentTRUE_ | | -1.00 _id.CongruentTRUE_ | | |
|  | -0.19 _id.Attit_Issue_extr_ | | 0.12 _id.Attit_Issue_extr_ | | |
| Marginal R^2^ / Conditional R^2^ | 0.203 / NA | | 0.206 / NA | | |

### Congruence x Issue moralization controlling for Congruence x Attitude extremity

| **Experiment 2 - true news** | | | |
| --- | --- | --- | --- |
|  | **sharing** | | |
| *Predictors* | *ß* | *p* | *std. p* |
| Congruent [TRUE] | 0.26 (0.16,0.35) | 0.904 | **<0.001** |
| AbsImpIssue [TRUE] | 0.11 (-0.02,0.23) | 0.091 | 0.087 |
| Attit Issue extr | -0.07 (-0.14, -0.01) | **0.026** | **0.026** |
| edu | 0.23 (0.16,0.31) | **<0.001** | **<0.001** |
| sex [female] | 0.06 (-0.09,0.22) | 0.430 | 0.431 |
| age | -0.20 (-0.27, -0.12) | **<0.001** | **<0.001** |
| Congruent [TRUE] * AbsImpIssue [TRUE] | 0.10 (-0.05,0.25) | 0.194 | 0.198 |
| Congruent [TRUE] * Attit Issue extr | 0.13 (0.06,0.20) | **0.001** | **<0.001** |
| **Random Effects** | | | |
| σ^2^ | 0.45 | | |
| τ_00_ _id_ | 0.48 | | |
| τ_00_ _News_ | 0.02 | | |
| τ_11_ _id.CongruentTRUE_ | 0.02 | | |
| τ_11_ _id.AbsImpIssueTRUE_ | 0.01 | | |
| ρ_01_ _id.CongruentTRUE_ | -0.26 | | |
| ρ_01_ _id.AbsImpIssueTRUE_ | -0.32 | | |
| Marginal R^2^ / Conditional R^2^ | 0.213 / NA | | |

### Effects of partisanship

| **Experiment 2 - true news** | | | | | | | | | | | | |
| --- | --- | --- | --- | --- | --- | --- | --- | --- | --- | --- | --- | --- |
|  | **SHARING** | | | **SHARING** | | | **SHARING** | | | **SHARING** | | |
| *Predictors* | *ß* | *p* | *std. p* | *ß* | *p* | *std. p* | *ß* | *p* | *std. p* | *ß* | *p* | *std. p* |
| Congruent [TRUE] | 0.28 (0.20,0.36) | **<0.001** | **<0.001** | 0.35 (0.23,0.46) | **<0.001** | **<0.001** | 0.23 (0.09,0.38) | **0.001** | **0.001** | 0.33 (0.22,0.44) | 0.829 | **<0.001** |
| Congruent [NA] | 0.20 (0.03,0.37) | **0.024** | **0.024** | 0.30 (0.12,0.49) | **0.002** | **0.002** | 0.33 (0.12,0.53) | **0.002** | **0.002** |  |  |  |
| edu | 0.22 (0.14,0.29) | **<0.001** | **<0.001** | 0.21 (0.14,0.28) | **<0.001** | **<0.001** | 0.21 (0.13,0.28) | **<0.001** | **<0.001** | 0.22 (0.15,0.30) | **<0.001** | **<0.001** |
| sex [female] | 0.07 (-0.08,0.22) | 0.369 | 0.369 | 0.10 (-0.05,0.26) | 0.187 | 0.187 | 0.09 (-0.06,0.25) | 0.234 | 0.234 | 0.13 (-0.03,0.28) | 0.120 | 0.120 |
| age | -0.16 (-0.24, -0.09) | **<0.001** | **<0.001** | -0.18 (-0.25, -0.10) | **<0.001** | **<0.001** | -0.18 (-0.25, -0.10) | **<0.001** | **<0.001** | -0.21 (-0.29, -0.13) | **<0.001** | **<0.001** |
| Pol Orient bin [Republican] |  |  |  | 0.30 (0.13,0.48) | **0.001** | **0.001** | 0.29 (0.10,0.48) | **0.003** | **0.003** | 0.30 (0.13,0.47) | 0.149 | **0.001** |
| Congruent [TRUE] * Pol Orient bin [Republican] |  |  |  | -0.13 (-0.28,0.02) | 0.082 | 0.082 | -0.04 (-0.23,0.14) | 0.657 | 0.657 | -0.09 (-0.23,0.05) | 0.614 | 0.209 |
| Congruent [NA] * Pol Orient bin [Republican] |  |  |  | -0.22 (-0.36, -0.08) | **0.002** | **0.002** | -0.21 (-0.37, -0.04) | **0.014** | **0.014** |  |  |  |
| AbsImpIssue [TRUE] |  |  |  |  |  |  | 0.04 (-0.10,0.19) | 0.579 | 0.579 |  |  |  |
| Congruent [TRUE] * AbsImpIssue [TRUE] |  |  |  |  |  |  | 0.26 (0.07,0.45) | **0.008** | **0.008** |  |  |  |
| AbsImpIssue [TRUE] * Pol Orient bin [Republican] |  |  |  |  |  |  | 0.06 (-0.16,0.29) | 0.571 | 0.571 |  |  |  |
| (Congruent [TRUE] * AbsImpIssue [TRUE]) * Pol Orient bin [Republican] |  |  |  |  |  |  | -0.18 (-0.47,0.12) | 0.239 | 0.239 |  |  |  |
| Attit Issue extr |  |  |  |  |  |  |  |  |  | -0.06 (-0.14,0.02) | 0.142 | 0.142 |
| Congruent [TRUE] * Attit Issue extr |  |  |  |  |  |  |  |  |  | 0.15 (0.06,0.24) | **0.002** | **0.002** |
| Attit Issue extr * Pol Orient bin [Republican] |  |  |  |  |  |  |  |  |  | 0.04 (-0.08,0.16) | 0.509 | 0.509 |
| (Congruent [TRUE] * Attit Issue extr) * Pol Orient bin [Republican] |  |  |  |  |  |  |  |  |  | -0.01 (-0.14,0.13) | 0.936 | 0.936 |
| **Random Effects** | | | | | | | | | | | | |
| σ^2^ | 0.47 | | | 0.47 | | | 0.47 | | | 0.46 | | |
| τ_00_ | 0.47 _id_ | | | 0.44 _id_ | | | 0.44 _id_ | | | 0.42 _id_ | | |
|  | 0.02 _News_ | | | 0.02 _News_ | | | 0.02 _News_ | | | 0.02 _News_ | | |
| τ_11_ | 0.03 _id.CongruentTRUE_ | | | 0.82 _id.Pol.Orient_binRepublican_ | | | 0.79 _id.Pol.Orient_binRepublican_ | | | 1.42 _id.Pol.Orient_binRepublican_ | | |
|  | 0.03 _id.CongruentNA_ | | | 0.04 _id.CongruentTRUE_ | | | 0.04 _id.CongruentTRUE_ | | | 0.01 _id.CongruentTRUE_ | | |
|  |  | | | 0.03 _id.CongruentNA_ | | | 0.03 _id.CongruentNA_ | | |  | | |
| ρ_01_ | -0.29 _id.CongruentTRUE_ | | | -0.66 _id.Pol.Orient_binRepublican_ | | | -0.65 _id.Pol.Orient_binRepublican_ | | | -0.89 _id.Pol.Orient_binRepublican_ | | |
|  | -0.18 _id.CongruentNA_ | | | -0.04 _id.CongruentTRUE_ | | | -0.05 _id.CongruentTRUE_ | | | 0.60 _id.CongruentTRUE_ | | |
|  |  | | | -0.01 _id.CongruentNA_ | | | -0.02 _id.CongruentNA_ | | |  | | |
| ICC |  | | | 0.50 | | | 0.50 | | | 0.50 | | |
| Marginal R^2^ / Conditional R^2^ | 0.150 / NA | | | 0.095 / 0.546 | | | 0.101 / 0.551 | | | 0.129 / 0.567 | | |

## Exploratory analyses of motivations to share

***Procedure***

Experiment 2 replicated Experiment 1 while additionally testing whether three hypothesized motivations—perceived accuracy, informativeness, and usefulness of a news items for participants’ political commitments—may influence news sharing preferences and correlate with our primary effects of interest (i.e., issue moralization and attitude extremity magnifying myside sharing preferences).

The procedure was identical to Experiment 1’s except that the willingness to share question was followed by three questions on motivations to share, displayed on the same page in a random order. Responses to these additional questions were all collected on 0-100 slider scales with 50 as default choice. The questions asked: “*How accurate do you think this news story is?”* (from [0] “*Not at all accurate”,* to [100] “*Completely accurate*”); *“How informative do you find that news item?*” (from [0] “*I knew that already*” to [100] “*Extremely informative*”); and *“How useful is this piece of news for your personal political commitments?”* (from [0] “*Not at all*” to [100] “*Extremely useful*”). In order to reduce fatigue, participants were randomly allocated to one of two groups which exposed them to only six of the 12 news items.

Experiment 1’s analyses of sharing intentions are reported in the body of the paper in the Results section of Experiments 1-4. Here, were report only the analyses of the reasons to share the news items.

***Results***

Political usefulness, informativeness and accuracy significantly and positively predicted willingness to share a news story regardless of news congruence and moralization of the issue. The strongest predictor of willingness to share was an item’s usefulness for one’s political commitments (ß = 0.29, [0.25,0.33]), *p* < 0.001), followed by its perceived informativeness (ß = 0.24, [0.20, 0.28], *p* < 0.001), and its perceived accuracy (ß = 0.19, [0.16, 0.23], *p* < 0.001). Time spent viewing the story did not predict willingness to share: ß = 0.01, [-0.02, 0.03], *p* = 0.5.

In terms of average scores, perceived accuracy was higher for congruent than incongruent news (ß = 0.31, [0.21,0.41], *p* < 0.001) but the difference was not significantly magnified by issue moralization (ß = 0.13, [-0.04,0.31], *p* = 0.13). Second, the perceived informativeness of congruent news was greater than incongruent news (ß = 0.21, [0.12,0.31], *p* < 0.001), and this difference was amplified by issue moralization (ß = 0.18, [0.02, 0.35], *p* < 0.05). There was also a main effect of issue moralization on perceived informativeness of the news, regardless of news congruence (ß = 0.16, [0.07, 0.25], *p* < 0.001). Third, congruent stories were perceived as more useful to participants’ political commitments than incongruent ones (ß = 0.26, [0.19,0.34], *p* < 0.001), and this difference was magnified by issue moralization (ß = 0.21, [0.07,0.35], *p* < 0.01).

### Motivations to share: Accuracy

| **Experiment 2 - true news** | | | | |
| --- | --- | --- | --- | --- |
|  | **Accuracy** | | **Accuracy** | |
| *Predictors* | *ß* | *p* | *ß* | *p* |
| Congruent [TRUE] | 0.31 (0.21,0.41) | **<0.001** | 0.26 (0.14,0.38) | **<0.001** |
| Congruent [NA] | 0.35 (0.07,0.64) | **0.015** | 0.33 (0.04,0.62) | **0.025** |
| AbsImpIssue [TRUE] | 0.09 (-0.00,0.19) | 0.058 | 0.03 (-0.10,0.16) | 0.678 |
| edu | 0.16 (0.10,0.22) | **<0.001** | 0.16 (0.10,0.22) | **<0.001** |
| sex [female] | -0.02 (-0.14,0.10) | 0.746 | -0.02 (-0.14,0.10) | 0.746 |
| age | -0.09 (-0.15, -0.03) | **0.005** | -0.09 (-0.15, -0.03) | **0.005** |
| Congruent [TRUE] * AbsImpIssue [TRUE] |  |  | 0.13 (-0.04,0.31) | 0.136 |
| **Random Effects** | | | | |
| σ^2^ | 470.80 | | 470.45 | |
| τ_00_ | 160.80 _IPAddress_ | | 160.86 _IPAddress_ | |
|  | 33.73 _News_ | | 34.36 _News_ | |
| ICC | 0.29 | | 0.29 | |
| Marginal R^2^ / Conditional R^2^ | 0.055 / 0.331 | | 0.056 / 0.333 | |

### Motivations to share: Usefulness for one’s political commitments

| **Experiment 2 - true news** | | | | |
| --- | --- | --- | --- | --- |
|  | **Useful commit** | | **Useful commit** | |
| *Predictors* | *ß* | *p* | *ß* | *p* |
| Congruent [TRUE] | 0.26 (0.19,0.34) | **<0.001** | 0.19 (0.10,0.28) | **<0.001** |
| Congruent [NA] | -0.18 (-0.34, -0.02) | **0.028** | -0.22 (-0.39, -0.05) | **0.011** |
| AbsImpIssue [TRUE] | 0.19 (0.11,0.27) | **<0.001** | 0.08 (-0.03,0.19) | 0.142 |
| edu | 0.25 (0.18,0.32) | **<0.001** | 0.25 (0.18,0.32) | **<0.001** |
| sex [female] | 0.01 (-0.13,0.14) | 0.906 | 0.01 (-0.13,0.14) | 0.907 |
| age | -0.19 (-0.26, -0.12) | **<0.001** | -0.19 (-0.26, -0.12) | **<0.001** |
| Congruent [TRUE] * AbsImpIssue [TRUE] |  |  | 0.21 (0.07,0.35) | **0.003** |
| **Random Effects** | | | | |
| σ^2^ | 498.31 | | 496.07 | |
| τ_00_ | 471.72 _IPAddress_ | | 472.11 _IPAddress_ | |
|  | 15.71 _News_ | | 16.92 _News_ | |
| ICC | 0.49 | | 0.50 | |
| Marginal R^2^ / Conditional R^2^ | 0.142 / 0.566 | | 0.144 / 0.569 | |

### Motivations to share: Informativeness

| **Experiment 2 - true news** | | | | |
| --- | --- | --- | --- | --- |
|  | **Informative** | | **Informative** | |
| *Predictors* | *ß* | *p* | *ß* | *p* |
| Congruent [TRUE] | 0.21 (0.12,0.31) | **<0.001** | 0.15 (0.04,0.26) | **0.008** |
| Congruent [NA] | 0.36 (0.14,0.59) | **0.002** | 0.33 (0.09,0.56) | **0.006** |
| AbsImpIssue [TRUE] | 0.16 (0.07,0.25) | **0.001** | 0.07 (-0.06,0.19) | 0.296 |
| edu | 0.16 (0.10,0.23) | **<0.001** | 0.16 (0.10,0.23) | **<0.001** |
| sex [female] | -0.01 (-0.14,0.12) | 0.839 | -0.01 (-0.14,0.12) | 0.838 |
| age | -0.12 (-0.19, -0.05) | **0.001** | -0.12 (-0.19, -0.05) | **0.001** |
| Congruent [TRUE] * AbsImpIssue [TRUE] |  |  | 0.18 (0.02,0.35) | **0.028** |
| **Random Effects** | | | | |
| σ^2^ | 417.17 | | 416.19 | |
| τ_00_ | 212.86 _IPAddress_ | | 213.02 _IPAddress_ | |
|  | 19.97 _News_ | | 21.39 _News_ | |
| ICC | 0.36 | | 0.36 | |
| Marginal R^2^ / Conditional R^2^ | 0.057 / 0.395 | | 0.059 / 0.398 | |

### Regression analysis of willingness to share true news on each motivation to share

Regression analysis, pooling all items:

| **Experiment 2 - true news - pooling all true news** | | |
| --- | --- | --- |
|  | **SHARING** | |
| *Predictors* | *ß* | *p* |
| Accuracy | 0.19 (0.16,0.23) | **<0.001** |
| Useful commit | 0.29 (0.25,0.33) | **<0.001** |
| Informative | 0.24 (0.20,0.28) | **<0.001** |
| Time | 0.01 (-0.02,0.04) | 0.470 |
| edu | 0.07 (0.02,0.12) | **0.008** |
| sex [female] | 0.06 (-0.04,0.16) | 0.248 |
| age | -0.05 (-0.11, -0.00) | **0.041** |
| **Random Effects** | | |
| σ^2^ | 0.34 | |
| τ_00_ _IPAddress_ | 0.19 | |
| τ_00_ _News_ | 0.01 | |
| ICC | 0.37 | |
| Marginal R^2^ / Conditional R^2^ | 0.430 / 0.638 | |

Regression analysis, breaking down by political news type:

| **Experiment 2 - true news - congruent news, high importance issues** | | |
| --- | --- | --- |
|  | **SHARING** | |
| *Predictors* | *ß* | *p* |
| Accuracy | 0.10 (-0.01,0.21) | 0.066 |
| Useful commit | 0.26 (0.14,0.38) | **<0.001** |
| Informative | 0.32 (0.19,0.44) | **<0.001** |
| Time | -0.06 (-0.15,0.02) | 0.132 |
| edu | 0.09 (0.00,0.18) | **0.048** |
| sex [female] | -0.12 (-0.31,0.06) | 0.192 |
| age | -0.08 (-0.18,0.01) | 0.089 |
| **Random Effects** | | |
| σ^2^ | 0.40 | |
| τ_00_ _IPAddress_ | 0.16 | |
| τ_00_ _News_ | 0.01 | |
| ICC | 0.29 | |
| Marginal R^2^ / Conditional R^2^ | 0.427 / 0.596 | |

| **Experiment 2 - true news - congruent news, low importance issues** | | |
| --- | --- | --- |
|  | **SHARING** | |
| *Predictors* | *ß* | *p* |
| Accuracy | 0.15 (0.07,0.23) | **<0.001** |
| Useful commit | 0.45 (0.36,0.54) | **<0.001** |
| Informative | 0.19 (0.10,0.27) | **<0.001** |
| Time | 0.01 (-0.05,0.07) | 0.750 |
| edu | 0.05 (-0.02,0.12) | 0.144 |
| sex [female] | 0.11 (-0.04,0.25) | 0.140 |
| age | -0.03 (-0.10,0.04) | 0.403 |
| **Random Effects** | | |
| σ^2^ | 0.34 | |
| τ_00_ _IPAddress_ | 0.14 | |
| τ_00_ _News_ | 0.00 | |
| ICC | 0.30 | |
| Marginal R^2^ / Conditional R^2^ | 0.521 / 0.664 | |

| **Experiment 2 - true news - incongruent news, high importance issues** | | |
| --- | --- | --- |
|  | **SHARING** | |
| *Predictors* | *ß* | *p* |
| Accuracy | 0.18 (0.07,0.29) | **0.001** |
| Useful commit | 0.33 (0.22,0.44) | **<0.001** |
| Informative | 0.25 (0.14,0.36) | **<0.001** |
| Time | 0.06 (-0.02,0.14) | 0.116 |
| edu | 0.08 (-0.01,0.16) | 0.083 |
| sex [female] | 0.00 (-0.18,0.18) | 0.998 |
| age | -0.04 (-0.13,0.05) | 0.356 |
| **Random Effects** | | |
| σ^2^ | 0.28 | |
| τ_00_ _IPAddress_ | 0.21 | |
| τ_00_ _News_ | 0.03 | |
| ICC | 0.46 | |
| Marginal R^2^ / Conditional R^2^ | 0.486 / 0.720 | |

| **Experiment 2 - true news - incongruent news, low importance issues** | | |
| --- | --- | --- |
|  | **SHARING** | |
| *Predictors* | *ß* | *p* |
| Accuracy | 0.14 (0.06,0.22) | **<0.001** |
| Useful commit | 0.45 (0.36,0.54) | **<0.001** |
| Informative | 0.21 (0.12,0.30) | **<0.001** |
| Time | -0.01 (-0.07,0.05) | 0.811 |
| edu | 0.07 (0.00,0.13) | **0.042** |
| sex [female] | 0.12 (-0.02,0.25) | 0.089 |
| age | -0.07 (-0.14, -0.01) | **0.035** |
| **Random Effects** | | |
| σ^2^ | 0.34 | |
| τ_00_ _IPAddress_ | 0.10 | |
| τ_00_ _News_ | 0.00 | |
| ICC | 0.23 | |
| Marginal R^2^ / Conditional R^2^ | 0.565 / 0.663 | |

# F. Experiment 3: Willingness to share 2-point, true news

### Congruence x Issue moralization

| **Experiment 3 - true news** | | | | | |
| --- | --- | --- | --- | --- | --- |
|  | **sharing** | | **sharing** | | |
| *Predictors* | *ß* | *p* | *ß* | *p* | *std. p* |
| Congruent [TRUE] | 0.28 (0.20,0.36) | **<0.001** | 0.19 (0.10,0.28) | **<0.001** | **<0.001** |
| Congruent [NA] | 0.42 (0.25,0.60) | **<0.001** | 0.38 (0.19,0.57) | **<0.001** | **<0.001** |
| AbsImpIssue [TRUE] | 0.19 (0.11,0.27) | **<0.001** | 0.06 (-0.05,0.16) | 0.293 | 0.293 |
| edu | 0.06 (-0.00,0.12) | 0.058 | 0.06 (-0.00,0.12) | 0.059 | 0.059 |
| sex [female] | -0.08 (-0.20,0.04) | 0.192 | -0.08 (-0.20,0.04) | 0.194 | 0.194 |
| age | -0.08 (-0.14, -0.02) | **0.009** | -0.08 (-0.14, -0.02) | **0.009** | **0.009** |
| Congruent [TRUE] * AbsImpIssue [TRUE] |  |  | 0.26 (0.13,0.40) | **<0.001** | **<0.001** |
| **Random Effects** | | | | | |
| σ^2^ | 0.15 | | 0.15 | | |
| τ_00_ | 0.06 _id_ | | 0.06 _id_ | | |
|  | 0.00 _News_ | | 0.00 _News_ | | |
| τ_11_ | 0.02 _id.CongruentTRUE_ | | 0.02 _id.CongruentTRUE_ | | |
|  | 0.08 _id.CongruentNA_ | | 0.05 _id.CongruentNA_ | | |
|  | 0.02 _id.AbsImpIssueTRUE_ | | 0.02 _id.AbsImpIssueTRUE_ | | |
|  | 0.20 _id.AbsImpIssueNA_ | | 0.16 _id.AbsImpIssueNA_ | | |
| ρ_01_ | 0.03 _id.CongruentTRUE_ | | 0.05 _id.CongruentTRUE_ | | |
|  | -0.35 _id.CongruentNA_ | | -0.10 _id.CongruentNA_ | | |
|  | -0.18 _id.AbsImpIssueTRUE_ | | -0.20 _id.AbsImpIssueTRUE_ | | |
|  | 0.13 _id.AbsImpIssueNA_ | | -0.04 _id.AbsImpIssueNA_ | | |
| ICC | 0.36 | | 0.36 | | |
| Marginal R^2^ / Conditional R^2^ | 0.040 / 0.384 | | 0.043 / 0.392 | | |

### Congruence x Attitude extremity

| **Experiment 3 - true news** | | | | | |
| --- | --- | --- | --- | --- | --- |
|  | **sharing** | | **sharing** | | |
| *Predictors* | *ß* | *p* | *ß* | *p* | *std. p* |
| Congruent [TRUE] | 0.29 (0.21,0.37) | **<0.001** | 0.28 (0.20,0.36) | 0.251 | **<0.001** |
| Attit Issue extr | 0.04 (-0.01,0.08) | 0.114 | -0.05 (-0.11,0.01) | 0.078 | 0.078 |
| edu | 0.06 (-0.01,0.13) | 0.081 | 0.06 (-0.01,0.12) | 0.084 | 0.084 |
| sex [female] | -0.08 (-0.21,0.05) | 0.246 | -0.08 (-0.21,0.06) | 0.259 | 0.259 |
| age | -0.10 (-0.16, -0.03) | **0.004** | -0.10 (-0.16, -0.03) | **0.005** | **0.005** |
| Congruent [TRUE] * Attit Issue extr |  |  | 0.18 (0.11,0.24) | **<0.001** | **<0.001** |
| **Random Effects** | | | | | |
| σ^2^ | 0.13 | | 0.13 | | |
| τ_00_ | 0.11 _id_ | | 0.11 _id_ | | |
|  | 0.01 _News_ | | 0.00 _News_ | | |
| τ_11_ | 0.03 _id.CongruentTRUE_ | | 0.02 _id.CongruentTRUE_ | | |
|  | 0.06 _id.Attit_Issue_extr_ | | 0.06 _id.Attit_Issue_extr_ | | |
| ρ_01_ | -0.39 _id.CongruentTRUE_ | | -0.35 _id.CongruentTRUE_ | | |
|  | -0.75 _id.Attit_Issue_extr_ | | -0.75 _id.Attit_Issue_extr_ | | |
| ICC | 0.37 | | 0.37 | | |
| Marginal R^2^ / Conditional R^2^ | 0.035 / 0.390 | | 0.042 / 0.394 | | |

### Congruence x Issue moralization controlling for Congruence x Attitude extremity

| **Experiment 3 - true news** | | | |
| --- | --- | --- | --- |
|  | **sharing** | | |
| *Predictors* | *ß* | *p* | *std. p* |
| Congruent [TRUE] | 0.23 (0.14,0.32) | 0.290 | **<0.001** |
| AbsImpIssue [TRUE] | 0.12 (0.01,0.23) | **0.034** | **0.034** |
| Attit Issue extr | -0.08 (-0.14, -0.02) | **0.006** | **0.006** |
| edu | 0.06 (-0.01,0.12) | 0.102 | 0.102 |
| sex [female] | -0.08 (-0.21,0.06) | 0.262 | 0.262 |
| age | -0.09 (-0.16, -0.02) | **0.008** | **0.008** |
| Congruent [TRUE] * AbsImpIssue [TRUE] | 0.17 (0.03,0.31) | **0.019** | **0.019** |
| Congruent [TRUE] * Attit Issue extr | 0.15 (0.08,0.22) | **<0.001** | **<0.001** |
| **Random Effects** | | | |
| σ^2^ | 0.12 | | |
| τ_00_ _id_ | 0.11 | | |
| τ_00_ _News_ | 0.01 | | |
| τ_11_ _id.CongruentTRUE_ | 0.03 | | |
| τ_11_ _id.AbsImpIssueTRUE_ | 0.04 | | |
| τ_11_ _id.Attit_Issue_extr_ | 0.05 | | |
| ρ_01_ _id.CongruentTRUE_ | -0.37 | | |
| ρ_01_ _id.AbsImpIssueTRUE_ | -0.09 | | |
| ρ_01_ _id.Attit_Issue_extr_ | -0.74 | | |
| ICC | 0.41 | | |
| Marginal R^2^ / Conditional R^2^ | - 1. 0.441 | | |

### Effects of partisanship

| **Experiment 3 - true news** | | | | | | | | | | |
| --- | --- | --- | --- | --- | --- | --- | --- | --- | --- | --- |
|  | **SHARING** | | **SHARING** | | **SHARING** | | | **SHARING** | | |
| *Predictors* | *ß* | *p* | *ß* | *p* | *ß* | *p* | *std. p* | *ß* | *p* | *std. p* |
| Congruent [TRUE] | 0.28 (0.20,0.36) | **<0.001** | 0.29 (0.18,0.40) | **<0.001** | 0.22 (0.09,0.35) | **0.001** | **0.001** | 0.27 (0.16,0.39) | 0.284 | **<0.001** |
| Congruent [NA] | 0.37 (0.19,0.55) | **<0.001** | 0.44 (0.25,0.64) | **<0.001** | 0.49 (0.28,0.70) | **<0.001** | **<0.001** |  |  |  |
| edu | 0.03 (-0.03,0.09) | 0.313 | 0.01 (-0.05,0.07) | 0.703 | 0.01 (-0.05,0.07) | 0.700 | 0.699 | 0.01 (-0.06,0.07) | 0.796 | 0.796 |
| sex [female] | -0.09 (-0.21,0.03) | 0.143 | -0.05 (-0.17,0.06) | 0.369 | -0.06 (-0.17,0.06) | 0.340 | 0.340 | -0.05 (-0.18,0.08) | 0.468 | 0.468 |
| age | -0.07 (-0.13, -0.01) | **0.021** | -0.07 (-0.13, -0.01) | **0.017** | -0.07 (-0.13, -0.01) | **0.015** | **0.015** | -0.09 (-0.15, -0.02) | **0.007** | **0.007** |
| Pol Orient bin [Republican] |  |  | 0.29 (0.15,0.44) | **<0.001** | 0.33 (0.17,0.49) | **<0.001** | **<0.001** | 0.29 (0.14,0.44) | **0.031** | **<0.001** |
| Congruent [TRUE] * Pol Orient bin [Republican] |  |  | -0.01 (-0.16,0.15) | 0.942 | -0.04 (-0.22,0.15) | 0.686 | 0.686 | 0.04 (-0.12,0.21) | 0.597 | 0.608 |
| Congruent [NA] * Pol Orient bin [Republican] |  |  | -0.16 (-0.32,0.00) | 0.051 | -0.20 (-0.37, -0.02) | **0.028** | **0.028** |  |  |  |
| AbsImpIssue [TRUE] |  |  |  |  | 0.11 (-0.02,0.24) | 0.108 | 0.108 |  |  |  |
| Congruent [TRUE] * AbsImpIssue [TRUE] |  |  |  |  | 0.21 (0.03,0.39) | **0.024** | **0.024** |  |  |  |
| AbsImpIssue [TRUE] * Pol Orient bin [Republican] |  |  |  |  | -0.09 (-0.30,0.12) | 0.394 | 0.394 |  |  |  |
| (Congruent [TRUE] * AbsImpIssue [TRUE]) * Pol Orient bin [Republican] |  |  |  |  | 0.12 (-0.17,0.40) | 0.428 | 0.428 |  |  |  |
| Attit Issue extr |  |  |  |  |  |  |  | -0.03 (-0.10,0.04) | 0.344 | 0.344 |
| Congruent [TRUE] * Attit Issue extr |  |  |  |  |  |  |  | 0.19 (0.09,0.28) | **<0.001** | **<0.001** |
| Attit Issue extr * Pol Orient bin [Republican] |  |  |  |  |  |  |  | 0.00 (-0.11,0.11) | 0.998 | 0.998 |
| (Congruent [TRUE] * Attit Issue extr) * Pol Orient bin [Republican] |  |  |  |  |  |  |  | -0.02 (-0.16,0.12) | 0.767 | 0.767 |
| **Random Effects** | | | | | | | | | | |
| σ^2^ | 0.15 | | 0.15 | | 0.15 | | | 0.14 | | |
| τ_00_ | 0.05 _id_ | | 0.03 _id_ | | 0.03 _id_ | | | 0.04 _id_ | | |
|  | 0.00 _News_ | | 0.00 _News_ | | 0.00 _News_ | | | 0.01 _News_ | | |
| τ_11_ | 0.02 _id.CongruentTRUE_ | | 0.11 _id.Pol.Orient_binRepublican_ | | 0.12 _id.Pol.Orient_binRepublican_ | | | 0.15 _id.Pol.Orient_binRepublican_ | | |
|  | 0.03 _id.CongruentNA_ | | 0.02 _id.CongruentTRUE_ | | 0.02 _id.CongruentTRUE_ | | | 0.02 _id.CongruentTRUE_ | | |
|  |  | | 0.03 _id.CongruentNA_ | | 0.03 _id.CongruentNA_ | | |  | | |
| ρ_01_ | 0.12 _id.CongruentTRUE_ | | -0.69 _id.Pol.Orient_binRepublican_ | | -0.74 _id.Pol.Orient_binRepublican_ | | | -0.83 _id.Pol.Orient_binRepublican_ | | |
|  | -0.14 _id.CongruentNA_ | | 0.15 _id.CongruentTRUE_ | | 0.12 _id.CongruentTRUE_ | | | -0.12 _id.CongruentTRUE_ | | |
|  |  | | -0.10 _id.CongruentNA_ | | -0.15 _id.CongruentNA_ | | |  | | |
| ICC | 0.31 | | 0.30 | | 0.31 | | | 0.32 | | |
| Marginal R^2^ / Conditional R^2^ | 0.033 / 0.333 | | 0.046 / 0.333 | | 0.055 / 0.345 | | | 0.060 / 0.363 | | |

# Pooled Experiments 1-3, true news

### Congruence x issue moralization, Congruence x attitude extremity, Congruence x issue moralization controlling for Congruence x attitude extremity

| **Pooled Experiments 1-3 - true news** | | | | | | | | | | | | |
| --- | --- | --- | --- | --- | --- | --- | --- | --- | --- | --- | --- | --- |
|  | **sharing** | | **sharing** | | **sharing** | | **sharing** | | | **sharing** | | |
| *Predictors* | *ß* | *p* | *ß* | *p* | *ß* | *p* | *ß* | *std. p* | *ß* | | *p* | *std. p* |
| Congruent [TRUE] | 0.29 (0.25,0.33) | **<0.001** | 0.21 (0.16,0.26) | **<0.001** | 0.29 (0.25,0.33) | **<0.001** | 0.29 (0.24,0.33) | **<0.001** | 0.24 (0.19,0.29) | | 0.730 | **<0.001** |
| AbsImpIssue [TRUE] | 0.18 (0.13,0.23) | **<0.001** | 0.06 (0.00,0.12) | **0.047** |  |  |  |  | 0.11 (0.04,0.17) | | **0.001** | **0.001** |
| edu | 0.14 (0.09,0.18) | **<0.001** | 0.14 (0.09,0.18) | **<0.001** | 0.14 (0.10,0.18) | **<0.001** | 0.14 (0.10,0.18) | **<0.001** | 0.14 (0.09,0.18) | | **<0.001** | **<0.001** |
| sex [male] | -0.02 (-0.10,0.07) | 0.673 | -0.02 (-0.10,0.07) | 0.673 | -0.03 (-0.11,0.06) | 0.531 | -0.03 (-0.11,0.06) | 0.531 | -0.02 (-0.10,0.07) | | 0.661 | 0.661 |
| age | -0.16 (-0.21, -0.12) | **<0.001** | -0.16 (-0.21, -0.12) | **<0.001** | -0.17 (-0.21, -0.13) | **<0.001** | -0.17 (-0.21, -0.13) | **<0.001** | -0.16 (-0.21, -0.12) | | **<0.001** | **<0.001** |
| Congruent [TRUE] * AbsImpIssue [TRUE] |  |  | 0.24 (0.16,0.32) | **<0.001** |  |  |  |  | 0.16 (0.08,0.25) | | **<0.001** | **<0.001** |
| Attit Issue extr |  |  |  |  | 0.03 (0.00,0.05) | **0.038** | -0.04 (-0.07, -0.01) | **0.013** | -0.06 (-0.10, -0.03) | | **<0.001** | **<0.001** |
| Congruent [TRUE] * Attit Issue extr |  |  |  |  |  |  | 0.13 (0.09,0.17) | **<0.001** | 0.11 (0.07,0.14) | | **<0.001** | **<0.001** |
| **Random Effects** | | | | | | | | | | | | |
| σ^2^ | 0.14 | | 0.14 | | 0.14 | | 0.14 | | | 0.14 | | |
| τ_00_ | 0.08 _id_ | | 0.08 _id_ | | 0.08 _id_ | | 0.08 _id_ | | | 0.08 _id_ | | |
|  | 0.00 _News_ | | 0.01 _News_ | | 0.01 _News_ | | 0.00 _News_ | | | 0.01 _News_ | | |
| ICC | 0.39 | | 0.39 | | 0.38 | | 0.39 | | | 0.39 | | |
| Marginal R^2^ / Conditional R^2^ | 0.072 / 0.430 | | 0.076 / 0.436 | | 0.065 / 0.425 | | 0.069 / 0.429 | | | 0.078 / 0.437 | | |

### By issue analyses

| **Pooled Experiments 1-3 - true news, abortion issue only** | | | | | | | | | | | |
| --- | --- | --- | --- | --- | --- | --- | --- | --- | --- | --- | --- |
|  | **sharing** | | **sharing** | | **sharing** | | **sharing** | | | **sharing** | |
| *Predictors* | *ß* | *p* | *ß* | *p* | *ß* | *p* | *ß* | *std. p* | *ß* | | *std. p* |
| Congruent [TRUE] | 0.27 (0.21,0.34) | **<0.001** | 0.16 (0.08,0.25) | **<0.001** | 0.27 (0.21,0.34) | **<0.001** | 0.27 (0.21,0.34) | **<0.001** | 0.20 (0.11,0.28) | | **<0.001** |
| AbsImpIssue [TRUE] | 0.03 (-0.09,0.16) | 0.606 | -0.13 (-0.27,0.02) | 0.081 |  |  |  |  | -0.04 (-0.19,0.12) | | 0.628 |
| edu | 0.11 (0.05,0.17) | **<0.001** | 0.11 (0.05,0.17) | **<0.001** | 0.11 (0.05,0.16) | **<0.001** | 0.11 (0.05,0.16) | **<0.001** | 0.11 (0.05,0.16) | | **<0.001** |
| sex [male] | -0.01 (-0.12,0.11) | 0.918 | -0.01 (-0.12,0.11) | 0.918 | -0.02 (-0.14,0.10) | 0.765 | -0.02 (-0.14,0.10) | 0.765 | -0.01 (-0.13,0.11) | | 0.854 |
| age | -0.17 (-0.23, -0.11) | **<0.001** | -0.17 (-0.23, -0.11) | **<0.001** | -0.17 (-0.23, -0.11) | **<0.001** | -0.17 (-0.23, -0.11) | **<0.001** | -0.17 (-0.23, -0.11) | | **<0.001** |
| Congruent [TRUE] * AbsImpIssue [TRUE] |  |  | 0.32 (0.18,0.46) | **<0.001** |  |  |  |  | 0.23 (0.07,0.38) | | **0.004** |
| Attit Issue extr |  |  |  |  | -0.04 (-0.10,0.02) | 0.196 | -0.11 (-0.18, -0.05) | **0.001** | -0.11 (-0.18, -0.03) | | **0.005** |
| Congruent [TRUE] * Attit Issue extr |  |  |  |  |  |  | 0.15 (0.08,0.22) | **<0.001** | 0.11 (0.03,0.18) | | **0.004** |
| **Random Effects** | | | | | | | | | | | |
| σ^2^ | 0.11 | | 0.11 | | 0.11 | | 0.11 | | | 0.11 | |
| τ_00_ | 0.11 _id_ | | 0.11 _id_ | | 0.11 _id_ | | 0.11 _id_ | | | 0.11 _id_ | |
|  | 0.00 _News_ | | 0.00 _News_ | | 0.00 _News_ | | 0.00 _News_ | | | 0.00 _News_ | |
| ICC |  | | 0.50 | |  | |  | | | 0.51 | |
| Marginal R^2^ / Conditional R^2^ | 0.112 / NA | | 0.065 / 0.535 | | 0.114 / NA | | 0.126 / NA | | | 0.070 / 0.540 | |

| **Pooled Experiments 1-3 - true news, Gender equality issue only** | | | | | | | | | | | | |
| --- | --- | --- | --- | --- | --- | --- | --- | --- | --- | --- | --- | --- |
|  | **sharing** | | **sharing** | | **sharing** | | **sharing** | | | **sharing** | | |
| *Predictors* | *ß* | *p* | *ß* | *p* | *ß* | *p* | *ß* | *p* | *std. p* | *ß* | *p* | *std. p* |
| Congruent [TRUE] | 0.23 (0.14,0.32) | **<0.001** | 0.16 (0.06,0.26) | **0.002** | 0.23 (0.14,0.32) | **<0.001** | 0.23 (0.14,0.32) | 0.434 | **<0.001** | 0.18 (0.08,0.28) | 0.476 | **0.001** |
| AbsImpIssue [TRUE] | 0.23 (0.12,0.35) | **<0.001** | 0.08 (-0.06,0.23) | 0.278 |  |  |  |  |  | 0.11 (-0.03,0.26) | 0.133 | 0.133 |
| edu | 0.11 (0.06,0.17) | **<0.001** | 0.11 (0.06,0.17) | **<0.001** | 0.13 (0.07,0.18) | **<0.001** | 0.13 (0.07,0.18) | **<0.001** | **<0.001** | 0.11 (0.06,0.17) | **<0.001** | **<0.001** |
| sex [male] | -0.09 (-0.20,0.02) | 0.106 | -0.09 (-0.20,0.02) | 0.106 | -0.11 (-0.23, -0.00) | **0.042** | -0.11 (-0.23, -0.00) | **0.042** | **0.042** | -0.09 (-0.20,0.02) | 0.106 | 0.106 |
| age | -0.18 (-0.24, -0.13) | **<0.001** | -0.18 (-0.24, -0.13) | **<0.001** | -0.19 (-0.25, -0.14) | **<0.001** | -0.19 (-0.25, -0.14) | **<0.001** | **<0.001** | -0.18 (-0.24, -0.13) | **<0.001** | **<0.001** |
| Congruent [TRUE] * AbsImpIssue [TRUE] |  |  | 0.31 (0.14,0.48) | **<0.001** |  |  |  |  |  | 0.24 (0.06,0.41) | **0.009** | **0.009** |
| Attit Issue extr |  |  |  |  | 0.03 (-0.03,0.08) | 0.296 | -0.04 (-0.11,0.02) | 0.201 | 0.201 | -0.06 (-0.13,0.01) | 0.109 | 0.109 |
| Congruent [TRUE] * Attit Issue extr |  |  |  |  |  |  | 0.15 (0.07,0.22) | **<0.001** | **<0.001** | 0.12 (0.04,0.20) | **0.003** | **0.003** |
| **Random Effects** | | | | | | | | | | | | |
| σ^2^ | 0.15 | | 0.15 | | 0.15 | | 0.15 | | | 0.15 | | |
| τ_00_ | 0.08 _id_ | | 0.08 _id_ | | 0.08 _id_ | | 0.08 _id_ | | | 0.08 _id_ | | |
|  | 0.00 _News_ | | 0.01 _News_ | | 0.00 _News_ | | 0.00 _News_ | | | 0.01 _News_ | | |
| ICC | 0.34 | | 0.36 | | 0.35 | | 0.36 | | | 0.36 | | |
| Marginal R^2^ / Conditional R^2^ | 0.078 / 0.393 | | 0.086 / 0.412 | | 0.067 / 0.393 | | 0.072 / 0.402 | | | 0.088 / 0.415 | | |

| **Pooled Experiments 1-3 - true news, Gun Control issue only** | | | | | | | | | | | | |
| --- | --- | --- | --- | --- | --- | --- | --- | --- | --- | --- | --- | --- |
|  | **sharing** | | **sharing** | | **sharing** | | **sharing** | | | **sharing** | | |
| *Predictors* | *ß* | *p* | *ß* | *p* | *ß* | *p* | *ß* | *p* | *std. p* | *ß* | *p* | *std. p* |
| Congruent [TRUE] | 0.37 (0.30,0.45) | **<0.001** | 0.30 (0.21,0.40) | **<0.001** | 0.37 (0.30,0.45) | **<0.001** | 0.37 (0.30,0.45) | 0.386 | **<0.001** | 0.34 (0.24,0.44) | 0.406 | **<0.001** |
| AbsImpIssue [TRUE] | 0.11 (-0.00,0.23) | 0.057 | 0.02 (-0.12,0.16) | 0.743 |  |  |  |  |  | 0.12 (-0.02,0.27) | 0.099 | 0.099 |
| edu | 0.08 (0.02,0.13) | **0.008** | 0.08 (0.02,0.13) | **0.008** | 0.07 (0.02,0.13) | **0.013** | 0.07 (0.02,0.13) | **0.013** | **0.013** | 0.08 (0.02,0.14) | **0.005** | **0.005** |
| sex [male] | 0.01 (-0.11,0.12) | 0.917 | 0.01 (-0.11,0.12) | 0.917 | 0.00 (-0.11,0.12) | 0.965 | 0.00 (-0.11,0.12) | 0.965 | 0.965 | -0.00 (-0.12,0.11) | 0.939 | 0.939 |
| age | -0.10 (-0.15, -0.04) | **0.001** | -0.10 (-0.15, -0.04) | **0.001** | -0.09 (-0.14, -0.03) | **0.003** | -0.09 (-0.14, -0.03) | **0.003** | **0.003** | -0.09 (-0.15, -0.03) | **0.002** | **0.002** |
| Congruent [TRUE] * AbsImpIssue [TRUE] |  |  | 0.18 (0.03,0.33) | **0.023** |  |  |  |  |  | 0.08 (-0.08,0.25) | 0.305 | 0.305 |
| Attit Issue extr |  |  |  |  | -0.05 (-0.10,0.01) | 0.109 | -0.12 (-0.19, -0.05) | **0.001** | **0.001** | -0.14 (-0.21, -0.07) | **<0.001** | **<0.001** |
| Congruent [TRUE] * Attit Issue extr |  |  |  |  |  |  | 0.14 (0.07,0.22) | **<0.001** | **<0.001** | 0.13 (0.05,0.21) | **0.001** | **0.001** |
| **Random Effects** | | | | | | | | | | | | |
| σ^2^ | 0.14 | | 0.14 | | 0.14 | | 0.14 | | | 0.14 | | |
| τ_00_ | 0.09 _id_ | | 0.09 _id_ | | 0.09 _id_ | | 0.09 _id_ | | | 0.09 _id_ | | |
|  | 0.00 _News_ | | 0.00 _News_ | | 0.00 _News_ | | 0.00 _News_ | | | 0.00 _News_ | | |
| Marginal R^2^ / Conditional R^2^ | 0.080 / NA | | 0.084 / NA | | 0.079 / NA | | 0.088 / NA | | | 0.097 / NA | | |

| **Pooled Experiments 1-3 - true news, Racial equality issue only** | | | | | | | | | | | | |
| --- | --- | --- | --- | --- | --- | --- | --- | --- | --- | --- | --- | --- |
|  | **sharing** | | **sharing** | | **sharing** | | **sharing** | | | **sharing** | | |
| *Predictors* | *ß* | *p* | *ß* | *p* | *ß* | *p* | *ß* | *p* | *std. p* | *ß* | *p* | *std. p* |
| Congruent [TRUE] | 0.28 (0.19,0.37) | **<0.001** | 0.25 (0.15,0.35) | **<0.001** | 0.28 (0.19,0.37) | **<0.001** | 0.28 (0.19,0.37) | 0.226 | **<0.001** | 0.26 (0.16,0.36) | 0.206 | **<0.001** |
| AbsImpIssue [TRUE] | 0.19 (0.08,0.31) | **0.001** | 0.13 (-0.01,0.27) | 0.071 |  |  |  |  |  | 0.18 (0.03,0.33) | **0.017** | **0.017** |
| edu | 0.17 (0.12,0.23) | **<0.001** | 0.17 (0.12,0.23) | **<0.001** | 0.18 (0.12,0.23) | **<0.001** | 0.18 (0.12,0.23) | **<0.001** | **<0.001** | 0.17 (0.12,0.23) | **<0.001** | **<0.001** |
| sex [male] | 0.01 (-0.09,0.12) | 0.798 | 0.01 (-0.09,0.12) | 0.798 | 0.01 (-0.10,0.12) | 0.863 | 0.01 (-0.10,0.12) | 0.863 | 0.863 | 0.01 (-0.10,0.12) | 0.823 | 0.823 |
| age | -0.18 (-0.24, -0.13) | **<0.001** | -0.18 (-0.24, -0.13) | **<0.001** | -0.19 (-0.25, -0.14) | **<0.001** | -0.19 (-0.25, -0.14) | **<0.001** | **<0.001** | -0.18 (-0.23, -0.12) | **<0.001** | **<0.001** |
| Congruent [TRUE] * AbsImpIssue [TRUE] |  |  | 0.13 (-0.03,0.29) | 0.122 |  |  |  |  |  | 0.08 (-0.10,0.25) | 0.389 | 0.389 |
| Attit Issue extr |  |  |  |  | -0.00 (-0.06,0.05) | 0.887 | -0.05 (-0.11,0.02) | 0.175 | 0.175 | -0.07 (-0.14, -0.00) | **0.038** | **0.038** |
| Congruent [TRUE] * Attit Issue extr |  |  |  |  |  |  | 0.08 (0.01,0.16) | **0.027** | **0.027** | 0.07 (-0.01,0.15) | 0.071 | 0.071 |
| **Random Effects** | | | | | | | | | | | | |
| σ^2^ | 0.14 | | 0.14 | | 0.14 | | 0.14 | | | 0.14 | | |
| τ_00_ | 0.08 _id_ | | 0.08 _id_ | | 0.08 _id_ | | 0.08 _id_ | | | 0.08 _id_ | | |
|  | 0.02 _News_ | | 0.02 _News_ | | 0.02 _News_ | | 0.02 _News_ | | | 0.02 _News_ | | |
| ICC | 0.42 | | 0.43 | | 0.43 | | 0.43 | | | 0.43 | | |
| Marginal R^2^ / Conditional R^2^ | 0.088 / 0.473 | | 0.090 / 0.482 | | 0.080 / 0.474 | | 0.082 / 0.477 | | | 0.091 / 0.481 | | |

# H. Experiment 4: Willingness to share 4-point, fake news

### By issue analyses

#### Results reported with vs. without the fake abortion items

As demonstrated in the plot and regression tables below, restricted analyses by issue analyses revealed that in Experiment 4 on fake news, the size of participant’s myside sharing preference was smaller on the two fake abortion-related items ascribing highly provocative statements to Bernie Sanders and Mike Pence than on the four other issues. This myside sharing was also not amplified (but rather diminished) by issue moralization and attitude extremity (cf plots below), deviating from the trends found on the other issues and items, both true and fake, in the studies of this paper (*cf.* restricted analyses by issue below). We are not sure why the two Sanders and Pence fake abortion items were less subject to myside sharing than the others. Independent raters’ perceptions do not suggest they were significantly less plausible than the other fake items (see SOM-Q). We suspect that their deviation from the trends observed with the other items may come from the fact they are focused on particular politicians and ascribe them such preposterous statements that their respective supporters found them hostile, and were thus reluctant to share them. We report sharing patterns of the fake news items used in Experiment 4 first while including those two items, and then while excluding them. Results from the other 10 experiments reported in the paper otherwise systematically include all the items participants viewed and rated.

**Note:**  Abor = Abortion; Race = Race equality; GunCo = Gun control; Gender = Gender equality

| **Experiment 4 - Sharing of congruent items by issue (baseline: abortion)** | | |
| --- | --- | --- |
|  | **sharing** | |
| *Predictors* | *ß* | *p* |
| Issue [Gender] | 0.82 (0.70,0.93) | **<0.001** |
| Issue [GunCo] | 0.40 (0.29,0.52) | **<0.001** |
| Issue [Race] | 0.15 (0.03,0.26) | **0.011** |
| edu | -0.09 (-0.15, -0.02) | **0.010** |
| sex [female] | -0.17 (-0.35,0.01) | 0.065 |
| age | -0.04 (-0.10,0.03) | 0.293 |
| **Random Effects** | | |
| σ^2^ | 0.65 | |
| τ_00_ _id_ | 0.22 | |
| ICC | 0.25 | |
| Marginal R^2^ / Conditional R^2^ | 0.109 / 0.332 | |

| **Experiment 4 - fake news, abortion issue only** | | | | | | | | | | | | |
| --- | --- | --- | --- | --- | --- | --- | --- | --- | --- | --- | --- | --- |
|  | **sharing** | | **sharing** | | **sharing** | | **sharing** | | | **sharing** | | |
| *Predictors* | *ß* | *p* | *ß* | *p* | *ß* | *p* | *ß* | *p* | *std. p* | *ß* | *p* | *std. p* |
| Congruent [TRUE] | 0.08 (-0.04,0.21) | 0.193 | 0.23 (0.02,0.44) | **0.033** | 0.08 (-0.04,0.21) | 0.194 | 0.08 (-0.04,0.20) | **0.007** | 0.191 | 0.15 (-0.08,0.38) | **0.006** | 0.193 |
| AbsImpIssue [TRUE] | 0.22 (0.06,0.38) | **0.008** | 0.33 (0.12,0.54) | **0.002** |  |  |  |  |  | 0.23 (0.00,0.46) | **0.049** | **0.049** |
| edu | -0.11 (-0.19, -0.03) | **0.009** | -0.11 (-0.19, -0.03) | **0.009** | -0.10 (-0.18, -0.02) | **0.017** | -0.10 (-0.18, -0.02) | **0.017** | **0.017** | -0.10 (-0.19, -0.02) | **0.012** | **0.012** |
| sex [female] | -0.23 (-0.45, -0.00) | **0.047** | -0.23 (-0.45, -0.00) | **0.046** | -0.22 (-0.44,0.01) | 0.058 | -0.22 (-0.44,0.01) | 0.058 | 0.058 | -0.24 (-0.46, -0.01) | **0.040** | **0.040** |
| age | -0.03 (-0.11,0.05) | 0.470 | -0.03 (-0.11,0.05) | 0.471 | -0.03 (-0.11,0.05) | 0.490 | -0.03 (-0.11,0.05) | 0.491 | 0.491 | -0.03 (-0.11,0.06) | 0.519 | 0.519 |
| Congruent [TRUE] * AbsImpIssue [TRUE] |  |  | -0.22 (-0.48,0.04) | 0.090 |  |  |  |  |  | -0.10 (-0.39,0.19) | 0.483 | 0.483 |
| Attit Issue extr |  |  |  |  | 0.08 (0.00,0.16) | **0.046** | 0.15 (0.05,0.25) | **0.002** | **0.002** | 0.10 (-0.01,0.22) | 0.064 | 0.064 |
| Congruent [TRUE] * Attit Issue extr |  |  |  |  |  |  | -0.15 (-0.27, -0.03) | **0.018** | **0.018** | -0.13 (-0.26,0.01) | 0.072 | 0.072 |
| **Random Effects** | | | | | | | | | | | | |
| σ^2^ | 0.73 | | 0.73 | | 0.73 | | 0.72 | | | 0.72 | | |
| τ_00_ | 0.19 _id_ | | 0.19 _id_ | | 0.20 _id_ | | 0.20 _id_ | | | 0.20 _id_ | | |
|  | 0.00 _News_ | | 0.00 _News_ | | 0.00 _News_ | | 0.00 _News_ | | | 0.00 _News_ | | |
| ICC | 0.21 | |  | |  | |  | | |  | | |
| Marginal R^2^ / Conditional R^2^ | 0.030 / 0.232 | | 0.042 / NA | | 0.033 / NA | | 0.040 / NA | | | 0.047 / NA | | |

| **Experiment 4 - fake news, Gender equality issue only** | | | | | | | | | | | | |
| --- | --- | --- | --- | --- | --- | --- | --- | --- | --- | --- | --- | --- |
|  | **sharing** | | **sharing** | | **sharing** | | **sharing** | | | **sharing** | | |
| *Predictors* | *ß* | *p* | *ß* | *p* | *ß* | *p* | *ß* | *p* | *std. p* | *ß* | *p* | *std. p* |
| Congruent [TRUE] | 0.85 (0.62,1.07) | **<0.001** | 0.71 (0.54,0.88) | **<0.001** | 0.85 (0.62,1.07) | **<0.001** | 1.03 (0.93,1.14) | **0.014** | **<0.001** | 0.95 (0.75,1.15) | **0.022** | **<0.001** |
| AbsImpIssue [TRUE] | 0.09 (-0.04,0.22) | 0.163 | -0.18 (-0.36, -0.01) | **0.035** |  |  |  |  |  | -0.01 (-0.20,0.17) | 0.871 | 0.871 |
| edu | -0.01 (-0.08,0.06) | 0.766 | -0.01 (-0.08,0.06) | 0.766 | -0.01 (-0.08,0.06) | 0.785 | -0.01 (-0.08,0.06) | 0.785 | 0.785 | -0.01 (-0.08,0.06) | 0.775 | 0.775 |
| sex [female] | -0.07 (-0.26,0.11) | 0.455 | -0.07 (-0.26,0.11) | 0.455 | -0.06 (-0.25,0.13) | 0.522 | -0.06 (-0.25,0.13) | 0.522 | 0.522 | -0.07 (-0.26,0.12) | 0.484 | 0.484 |
| age | -0.01 (-0.07,0.06) | 0.839 | -0.01 (-0.07,0.06) | 0.839 | -0.01 (-0.08,0.06) | 0.804 | -0.01 (-0.08,0.06) | 0.804 | 0.804 | -0.01 (-0.08,0.06) | 0.828 | 0.828 |
| Congruent [TRUE] * AbsImpIssue [TRUE] |  |  | 0.55 (0.33,0.78) | **<0.001** |  |  |  |  |  | 0.22 (-0.01,0.46) | 0.057 | 0.057 |
| Attit Issue extr |  |  |  |  | 0.01 (-0.05,0.08) | 0.719 | -0.22 (-0.30, -0.13) | **<0.001** | **<0.001** | -0.22 (-0.31, -0.13) | **<0.001** | **<0.001** |
| Congruent [TRUE] * Attit Issue extr |  |  |  |  |  |  | 0.46 (0.35,0.56) | **<0.001** | **<0.001** | 0.42 (0.31,0.54) | **<0.001** | **<0.001** |
| **Random Effects** | | | | | | | | | | | | |
| σ^2^ | 0.64 | | 0.61 | | 0.64 | | 0.54 | | | 0.54 | | |
| τ_00_ | 0.08 _id_ | | 0.09 _id_ | | 0.08 _id_ | | 0.13 _id_ | | | 0.13 _id_ | | |
|  | 0.03 _News_ | | 0.00 _News_ | | 0.03 _News_ | | 0.00 _News_ | | | 0.00 _News_ | | |
| ICC | 0.14 | |  | | 0.15 | | 0.19 | | | 0.20 | | |
| Marginal R^2^ / Conditional R^2^ | 0.193 / 0.309 | | 0.318 / NA | | 0.191 / 0.309 | | 0.319 / 0.449 | | | 0.339 / 0.470 | | |

| **Experiment 4 - fake news, Gun Control issue only** | | | | | | | | | | | | |
| --- | --- | --- | --- | --- | --- | --- | --- | --- | --- | --- | --- | --- |
|  | **sharing** | | **sharing** | | **sharing** | | **sharing** | | | **sharing** | | |
| *Predictors* | *ß* | *p* | *ß* | *p* | *ß* | *p* | *ß* | *p* | *std. p* | *ß* | *p* | *std. p* |
| Congruent [TRUE] | 0.64 (0.51,0.78) | **<0.001** | 0.56 (0.41,0.71) | **<0.001** | 0.64 (0.51,0.78) | **<0.001** | 0.67 (0.54,0.80) | 0.747 | **<0.001** | 0.64 (0.49,0.79) | 0.759 | **<0.001** |
| AbsImpIssue [TRUE] | 0.12 (-0.04,0.28) | 0.150 | -0.04 (-0.24,0.17) | 0.739 |  |  |  |  |  | 0.05 (-0.16,0.27) | 0.618 | 0.618 |
| edu | -0.13 (-0.20, -0.05) | **0.001** | -0.13 (-0.20, -0.05) | **0.001** | -0.13 (-0.20, -0.06) | **0.001** | -0.13 (-0.20, -0.06) | **0.001** | **0.001** | -0.13 (-0.20, -0.05) | **0.001** | **0.001** |
| sex [female] | -0.38 (-0.59, -0.18) | **<0.001** | -0.38 (-0.59, -0.18) | **<0.001** | -0.39 (-0.59, -0.19) | **<0.001** | -0.39 (-0.59, -0.19) | **<0.001** | **<0.001** | -0.38 (-0.59, -0.18) | **<0.001** | **<0.001** |
| age | 0.01 (-0.07,0.08) | 0.856 | 0.01 (-0.07,0.08) | 0.856 | 0.01 (-0.06,0.09) | 0.727 | 0.01 (-0.06,0.09) | 0.727 | 0.727 | 0.01 (-0.07,0.08) | 0.830 | 0.830 |
| Congruent [TRUE] * AbsImpIssue [TRUE] |  |  | 0.31 (0.04,0.57) | **0.023** |  |  |  |  |  | 0.11 (-0.16,0.37) | 0.433 | 0.433 |
| Attit Issue extr |  |  |  |  | 0.03 (-0.04,0.10) | 0.428 | -0.13 (-0.22, -0.04) | **0.005** | **0.005** | -0.14 (-0.23, -0.04) | **0.005** | **0.005** |
| Congruent [TRUE] * Attit Issue extr |  |  |  |  |  |  | 0.31 (0.20,0.43) | **<0.001** | **<0.001** | 0.30 (0.18,0.42) | **<0.001** | **<0.001** |
| **Random Effects** | | | | | | | | | | | | |
| σ^2^ | 0.62 | | 0.62 | | 0.62 | | 0.58 | | | 0.58 | | |
| τ_00_ | 0.14 _id_ | | 0.15 _id_ | | 0.14 _id_ | | 0.16 _id_ | | | 0.16 _id_ | | |
|  | 0.01 _News_ | | 0.01 _News_ | | 0.01 _News_ | | 0.01 _News_ | | | 0.01 _News_ | | |
| ICC | 0.20 | | 0.20 | | 0.20 | | 0.23 | | | 0.23 | | |
| Marginal R^2^ / Conditional R^2^ | 0.140 / 0.311 | | 0.143 / 0.319 | | 0.138 / 0.311 | | 0.172 / 0.360 | | | 0.173 / 0.361 | | |

| **Experiment 4 - fake news, Racial equality issue only** | | | | | | | | | | | | |
| --- | --- | --- | --- | --- | --- | --- | --- | --- | --- | --- | --- | --- |
|  | **sharing** | | **sharing** | | **sharing** | | **sharing** | | | **sharing** | | |
| *Predictors* | *ß* | *p* | *ß* | *p* | *ß* | *p* | *ß* | *p* | *std. p* | *ß* | *p* | *std. p* |
| Congruent [TRUE] | 0.18 (-0.09,0.44) | 0.188 | 0.15 (-0.13,0.43) | 0.297 | 0.18 (-0.09,0.44) | 0.188 | 0.31 (0.02,0.60) | 0.535 | **0.035** | 0.31 (-0.01,0.63) | 0.535 | 0.061 |
| AbsImpIssue [TRUE] | 0.22 (0.08,0.36) | **0.002** | 0.19 (-0.00,0.37) | 0.051 |  |  |  |  |  | 0.21 (0.01,0.41) | **0.036** | **0.036** |
| edu | -0.00 (-0.07,0.07) | 0.963 | -0.00 (-0.07,0.07) | 0.963 | -0.00 (-0.08,0.07) | 0.916 | -0.00 (-0.08,0.07) | 0.917 | 0.917 | -0.00 (-0.07,0.07) | 0.970 | 0.970 |
| sex [female] | -0.54 (-0.74, -0.35) | **<0.001** | -0.54 (-0.74, -0.35) | **<0.001** | -0.57 (-0.78, -0.37) | **<0.001** | -0.57 (-0.78, -0.37) | **<0.001** | **<0.001** | -0.55 (-0.75, -0.35) | **<0.001** | **<0.001** |
| age | -0.07 (-0.15, -0.00) | **0.047** | -0.07 (-0.15, -0.00) | **0.047** | -0.07 (-0.15, -0.00) | **0.045** | -0.07 (-0.15, -0.00) | **0.045** | **0.045** | -0.07 (-0.15, -0.00) | **0.046** | **0.046** |
| Congruent [TRUE] * AbsImpIssue [TRUE] |  |  | 0.07 (-0.18,0.32) | 0.579 |  |  |  |  |  | 0.00 (-0.25,0.26) | 0.974 | 0.974 |
| Attit Issue extr |  |  |  |  | 0.05 (-0.02,0.12) | 0.190 | -0.02 (-0.11,0.08) | 0.737 | 0.737 | -0.05 (-0.15,0.05) | 0.295 | 0.295 |
| Congruent [TRUE] * Attit Issue extr |  |  |  |  |  |  | 0.13 (-0.00,0.26) | 0.059 | 0.059 | 0.13 (-0.01,0.26) | 0.072 | 0.072 |
| **Random Effects** | | | | | | | | | | | | |
| σ^2^ | 0.52 | | 0.52 | | 0.52 | | 0.51 | | | 0.52 | | |
| τ_00_ | 0.09 _id_ | | 0.09 _id_ | | 0.10 _id_ | | 0.10 _id_ | | | 0.09 _id_ | | |
|  | 0.15 _News_ | | 0.14 _News_ | | 0.15 _News_ | | 0.09 _News_ | | | 0.09 _News_ | | |
| ICC | 0.31 | | 0.31 | | 0.32 | | 0.27 | | | 0.26 | | |
| Marginal R^2^ / Conditional R^2^ | 0.054 / 0.351 | | 0.056 / 0.344 | | 0.045 / 0.351 | | 0.068 / 0.316 | | | 0.077 / 0.316 | | |

### Congruence x Issue moralization

| **Experiment 4 - Full dataset** | | | | | |
| --- | --- | --- | --- | --- | --- |
|  | **sharing** | | **sharing** | | |
| *Predictors* | *ß* | *p* | *ß* | *p* | *std. p* |
| Congruent [TRUE] | 0.44 (0.35,0.52) | **<0.001** | 0.40 (0.30,0.50) | **<0.001** | **<0.001** |
| AbsImpIssue [TRUE] | 0.14 (0.07,0.21) | **<0.001** | 0.11 (0.02,0.19) | **0.019** | **0.019** |
| edu | -0.05 (-0.11, -0.00) | **0.050** | -0.05 (-0.11, -0.00) | **0.049** | **0.049** |
| sex [female] | -0.32 (-0.47, -0.17) | **<0.001** | -0.32 (-0.47, -0.17) | **<0.001** | **<0.001** |
| age | -0.02 (-0.07,0.03) | 0.466 | -0.02 (-0.07,0.03) | 0.464 | 0.464 |
| Congruent [TRUE] * AbsImpIssue [TRUE] |  |  | 0.08 (-0.04,0.19) | 0.205 | 0.205 |
| **Random Effects** | | | | | |
| σ^2^ | 0.59 | | 0.59 | | |
| τ_00_ | 0.13 _id_ | | 0.13 _id_ | | |
|  | 0.08 _News_ | | 0.07 _News_ | | |
| τ_11_ | 0.02 _id.CongruentTRUE_ | | 0.02 _id.CongruentTRUE_ | | |
|  | 0.00 _id.AbsImpIssueTRUE_ | | 0.00 _id.AbsImpIssueTRUE_ | | |
| ρ_01_ | 1.00 _id.CongruentTRUE_ | | 1.00 _id.CongruentTRUE_ | | |
|  | -1.00 _id.AbsImpIssueTRUE_ | | -1.00 _id.AbsImpIssueTRUE_ | | |
| Marginal R^2^ / Conditional R^2^ | 0.095 / NA | | 0.098 / NA | | |

| **Experiment 4 - No fake abortion items** | | | | |
| --- | --- | --- | --- | --- |
|  | **sharing** | | **sharing** | |
| *Predictors* | *ß* | *p* | *ß* | *p* |
| Congruent [TRUE] | 0.60 (0.49,0.70) | **<0.001** | 0.51 (0.40,0.62) | **<0.001** |
| AbsImpIssue [TRUE] | 0.13 (0.05,0.21) | **0.001** | -0.01 (-0.11,0.09) | 0.891 |
| edu | -0.04 (-0.10,0.01) | 0.128 | -0.04 (-0.10,0.01) | 0.126 |
| sex [female] | -0.34 (-0.49, -0.19) | **<0.001** | -0.34 (-0.49, -0.19) | **<0.001** |
| age | -0.01 (-0.07,0.04) | 0.677 | -0.01 (-0.07,0.04) | 0.670 |
| Congruent [TRUE] * AbsImpIssue [TRUE] |  |  | 0.30 (0.16,0.43) | **<0.001** |
| **Random Effects** | | | | |
| σ^2^ | 0.53 | | 0.53 | |
| τ_00_ | 0.13 _id_ | | 0.13 _id_ | |
|  | 0.07 _News_ | | 0.06 _News_ | |
| τ_11_ | 0.06 _id.CongruentTRUE_ | | 0.06 _id.CongruentTRUE_ | |
|  | 0.00 _id.AbsImpIssueTRUE_ | | 0.00 _id.AbsImpIssueTRUE_ | |
| ρ_01_ | 0.31 _id.CongruentTRUE_ | | 0.25 _id.CongruentTRUE_ | |
|  | -0.59 _id.AbsImpIssueTRUE_ | | -0.75 _id.AbsImpIssueTRUE_ | |
| Marginal R^2^ / Conditional R^2^ | 0.158 / NA | | 0.187 / NA | |

### Congruence x Attitude extremity

| **Experiment 4 - Full dataset** | | | | | |
| --- | --- | --- | --- | --- | --- |
|  | **sharing** | | **sharing** | | |
| *Predictors* | *ß* | *p* | *ß* | *p* | *std. p* |
| Congruent [TRUE] | 0.43 (0.35,0.52) | **<0.001** | 0.51 (0.42,0.60) | 0.610 | **<0.001** |
| Attit Issue extr | 0.05 (0.01,0.09) | **0.010** | -0.03 (-0.08,0.01) | 0.164 | 0.164 |
| edu | -0.06 (-0.11, -0.00) | **0.038** | -0.06 (-0.11, -0.00) | **0.039** | **0.039** |
| sex [female] | -0.31 (-0.46, -0.16) | **<0.001** | -0.31 (-0.46, -0.16) | **<0.001** | **<0.001** |
| age | -0.02 (-0.07,0.04) | 0.562 | -0.02 (-0.07,0.04) | 0.534 | 0.534 |
| Congruent [TRUE] * Attit Issue extr |  |  | 0.18 (0.12,0.24) | **<0.001** | **<0.001** |
| **Random Effects** | | | | | |
| σ^2^ | 0.58 | | 0.57 | | |
| τ_00_ | 0.26 _id_ | | 0.26 _id_ | | |
|  | 0.07 _News_ | | 0.06 _News_ | | |
| τ_11_ | 0.02 _id.CongruentTRUE_ | | 0.02 _id.CongruentTRUE_ | | |
|  | 0.08 _id.Attit_Issue_extr_ | | 0.09 _id.Attit_Issue_extr_ | | |
| ρ_01_ | 0.38 _id.CongruentTRUE_ | | 0.46 _id.CongruentTRUE_ | | |
|  | -0.79 _id.Attit_Issue_extr_ | | -0.78 _id.Attit_Issue_extr_ | | |
| ICC | 0.31 | |  | | |
| Marginal R^2^ / Conditional R^2^ | 0.065 / 0.357 | | 0.125 / NA | | |

| **Experiment 4 - No fake abortion items** | | | | | |
| --- | --- | --- | --- | --- | --- |
|  | **sharing** | | **sharing** | | |
| *Predictors* | *ß* | *p* | *ß* | *p* | *std. p* |
| Congruent [TRUE] | 0.60 (0.49,0.70) | **<0.001** | 0.77 (0.66,0.88) | 0.180 | **<0.001** |
| Attit Issue extr | 0.03 (-0.01,0.07) | 0.197 | -0.13 (-0.19, -0.08) | **<0.001** | **<0.001** |
| edu | -0.05 (-0.10,0.01) | 0.086 | -0.05 (-0.10,0.01) | 0.091 | 0.091 |
| sex [female] | -0.33 (-0.48, -0.18) | **<0.001** | -0.33 (-0.48, -0.18) | **<0.001** | **<0.001** |
| age | -0.01 (-0.06,0.05) | 0.759 | -0.01 (-0.06,0.05) | 0.742 | 0.742 |
| Congruent [TRUE] * Attit Issue extr |  |  | 0.34 (0.27,0.41) | **<0.001** | **<0.001** |
| **Random Effects** | | | | | |
| σ^2^ | 0.53 | | 0.50 | | |
| τ_00_ | 0.31 _id_ | | 0.33 _id_ | | |
|  | 0.07 _News_ | | 0.05 _News_ | | |
| τ_11_ | 0.07 _id.CongruentTRUE_ | | 0.05 _id.CongruentTRUE_ | | |
|  | 0.10 _id.Attit_Issue_extr_ | | 0.13 _id.Attit_Issue_extr_ | | |
| ρ_01_ | -0.17 _id.CongruentTRUE_ | | -0.14 _id.CongruentTRUE_ | | |
|  | -0.88 _id.Attit_Issue_extr_ | | -0.86 _id.Attit_Issue_extr_ | | |
| ICC | 0.33 | |  | | |
| Marginal R^2^ / Conditional R^2^ | 0.110 / 0.400 | | 0.259 / NA | | |

### Congruence x Issue moralization controlling for Congruence x Attitude extremity

| **Experiment 4 - Full dataset** | | | |
| --- | --- | --- | --- |
|  | **sharing** | | |
| *Predictors* | *ß* | *p* | *std. p* |
| Congruent [TRUE] | 0.53 (0.42,0.64) | 0.589 | **<0.001** |
| AbsImpIssue [TRUE] | 0.14 (0.05,0.24) | **0.003** | **0.003** |
| Attit Issue extr | -0.06 (-0.11, -0.01) | **0.017** | **0.017** |
| edu | -0.05 (-0.11, -0.00) | **0.050** | **0.050** |
| sex [female] | -0.32 (-0.47, -0.18) | **<0.001** | **<0.001** |
| age | -0.02 (-0.07,0.03) | 0.466 | 0.466 |
| Congruent [TRUE] * AbsImpIssue [TRUE] | -0.05 (-0.18,0.07) | 0.409 | 0.409 |
| Congruent [TRUE] * Attit Issue extr | 0.19 (0.12,0.25) | **<0.001** | **<0.001** |
| **Random Effects** | | | |
| σ^2^ | 0.58 | | |
| τ_00_ _id_ | 0.13 | | |
| τ_00_ _News_ | 0.07 | | |
| τ_11_ _id.CongruentTRUE_ | 0.02 | | |
| τ_11_ _id.AbsImpIssueTRUE_ | 0.00 | | |
| ρ_01_ _id.CongruentTRUE_ | 1.00 | | |
| ρ_01_ _id.AbsImpIssueTRUE_ | -1.00 | | |
| Marginal R^2^ / Conditional R^2^ | 0.128 / NA | | |

| **Experiment 4 - No fake abortion items** | | | |
| --- | --- | --- | --- |
|  | **sharing** | | |
| *Predictors* | *ß* | *p* | *std. p* |
| Congruent [TRUE] | 0.73 (0.61,0.85) | 0.215 | **<0.001** |
| AbsImpIssue [TRUE] | 0.08 (-0.03,0.18) | 0.150 | 0.150 |
| Attit Issue extr | -0.15 (-0.20, -0.09) | **<0.001** | **<0.001** |
| edu | -0.04 (-0.09,0.01) | 0.140 | 0.140 |
| sex [female] | -0.33 (-0.48, -0.19) | **<0.001** | **<0.001** |
| age | -0.01 (-0.07,0.04) | 0.655 | 0.655 |
| Congruent [TRUE] * AbsImpIssue [TRUE] | 0.11 (-0.03,0.25) | 0.133 | 0.133 |
| Congruent [TRUE] * Attit Issue extr | 0.32 (0.24,0.39) | **<0.001** | **<0.001** |
| **Random Effects** | | | |
| σ^2^ | 0.52 | | |
| τ_00_ _id_ | 0.13 | | |
| τ_00_ _News_ | 0.05 | | |
| τ_11_ _id.CongruentTRUE_ | 0.04 | | |
| τ_11_ _id.AbsImpIssueTRUE_ | 0.00 | | |
| ρ_01_ _id.CongruentTRUE_ | 0.45 | | |
| ρ_01_ _id.AbsImpIssueTRUE_ | -0.70 | | |
| Marginal R^2^ / Conditional R^2^ | 0.263 / NA | | |

### Effects of partisanship

| **No fake abortion items** | | | | | | | | | |
| --- | --- | --- | --- | --- | --- | --- | --- | --- | --- |
|  | **SHARING** | | **SHARING** | | **SHARING** | | **SHARING** | | |
| *Predictors* | *ß* | *p* | *ß* | *p* | *ß* | *p* | *ß* | *p* | *std. p* |
| Congruent [TRUE] | 0.44 (0.35,0.52) | **<0.001** | 0.50 (0.39,0.60) | **<0.001** | 0.46 (0.34,0.58) | **<0.001** | 0.54 (0.43,0.65) | 0.179 | **<0.001** |
| edu | -0.05 (-0.11,0.00) | 0.053 | -0.07 (-0.12, -0.02) | **0.010** | -0.07 (-0.12, -0.02) | **0.010** | -0.07 (-0.12, -0.01) | **0.012** | **0.012** |
| sex [female] | -0.31 (-0.46, -0.16) | **<0.001** | -0.28 (-0.43, -0.13) | **<0.001** | -0.29 (-0.44, -0.14) | **<0.001** | -0.28 (-0.43, -0.13) | **<0.001** | **<0.001** |
| age | -0.02 (-0.08,0.03) | 0.442 | -0.02 (-0.08,0.03) | 0.396 | -0.02 (-0.08,0.03) | 0.405 | -0.02 (-0.08,0.03) | 0.404 | 0.404 |
| Pol Orient bin [Republican] |  |  | 0.13 (-0.02,0.28) | 0.084 | 0.11 (-0.06,0.29) | 0.195 | 0.14 (-0.01,0.29) | 0.647 | 0.075 |
| Congruent [TRUE] * Pol Orient bin [Republican] |  |  | -0.14 (-0.29,0.01) | 0.073 | -0.13 (-0.32,0.06) | 0.189 | -0.06 (-0.22,0.09) | 0.203 | 0.417 |
| AbsImpIssue [TRUE] |  |  |  |  | 0.09 (-0.01,0.19) | 0.066 |  |  |  |
| Congruent [TRUE] * AbsImpIssue [TRUE] |  |  |  |  | 0.07 (-0.07,0.20) | 0.319 |  |  |  |
| AbsImpIssue [TRUE] * Pol Orient bin [Republican] |  |  |  |  | 0.06 (-0.14,0.26) | 0.544 |  |  |  |
| (Congruent [TRUE] * AbsImpIssue [TRUE]) * Pol Orient bin [Republican] |  |  |  |  | 0.00 (-0.27,0.27) | 0.998 |  |  |  |
| Attit Issue extr |  |  |  |  |  |  | -0.03 (-0.09,0.02) | 0.237 | 0.237 |
| Congruent [TRUE] * Attit Issue extr |  |  |  |  |  |  | 0.15 (0.08,0.22) | **<0.001** | **<0.001** |
| Attit Issue extr * Pol Orient bin [Republican] |  |  |  |  |  |  | 0.03 (-0.07,0.13) | 0.593 | 0.593 |
| (Congruent [TRUE] * Attit Issue extr) * Pol Orient bin [Republican] |  |  |  |  |  |  | 0.06 (-0.07,0.19) | 0.369 | 0.369 |
| **Random Effects** | | | | | | | | | |
| σ^2^ | 0.59 | | 0.59 | | 0.58 | | 0.58 | | |
| τ_00_ | 0.13 _id_ | | 0.10 _id_ | | 0.10 _id_ | | 0.11 _id_ | | |
|  | 0.07 _News_ | | 0.07 _News_ | | 0.07 _News_ | | 0.06 _News_ | | |
| τ_11_ | 0.02 _id.CongruentTRUE_ | | 0.35 _id.Pol.Orient_binRepublican_ | | 0.34 _id.Pol.Orient_binRepublican_ | | 0.34 _id.Pol.Orient_binRepublican_ | | |
|  |  | | 0.03 _id.CongruentTRUE_ | | 0.03 _id.CongruentTRUE_ | | 0.02 _id.CongruentTRUE_ | | |
| ρ_01_ | 1.00 _id_ | | -0.60 _id.Pol.Orient_binRepublican_ | | -0.60 _id.Pol.Orient_binRepublican_ | | -0.57 _id.Pol.Orient_binRepublican_ | | |
|  |  | | 1.00 _id.CongruentTRUE_ | | 1.00 _id.CongruentTRUE_ | | 1.00 _id.CongruentTRUE_ | | |
| ICC | 0.30 | | 0.30 | | 0.30 | |  | | |
| Marginal R^2^ / Conditional R^2^ | 0.064 / 0.346 | | 0.072 / 0.353 | | 0.079 / 0.356 | | 0.132 / NA | | |

# I. Experiment 5a & 5b: Sharing from anonymous vs. personal social media account

## Pilot Experiment 5a (true news)

### Materials and procedure

The procedure was identical to Experiment 3 except for the following changes. Our previous designs had implicitly assumed that news sharing was done from a *personal* social media account. Asking participants to imagine they would now be sharing news items anonymously could imply that their account would potentially have no followers, which was unecological (there is *prima facie* no reason to follow an anonymous account online, unless its content is especially interesting). We decided to have the questionnaire begin, in both conditions, with a vignette asking participants to imagine they were working for an association that gives them money to promote any social cause they care about through news sharing. In both the personal account and the anonymous account conditions, sharing was made symbolically costly by telling participants they were given a fictional $100 endowment per news item. They were informed that they could spend whichever proportion of that endowment to promote content online, and keep the rest for themselves. The dependent variable was thus a *willingness to pay for sharing* each headline.

To emphasize anonymity in the anonymous account condition, participants were told they were working for an association called “Public Support”, and shown its (fictitious) Twitter home page at the beginning of the questionnaire.  In contrast, the personal account condition did not mention any association name nor any Twitter account, and the vignettes made it clear that news sharing would be done from participants’ own social media account. Below are the vignettes employed in both conditions:

**Anonymous account condition:**

**Page 1**

*Please read very carefully the following instruction (1/2):*

Imagine you work for an association, called 'Public Support', that gives you a budget for promoting any social cause you care about through sharing news online. The budget the association gives you is limited, but allows you to have a social impact on potentially thousands of users through news sharing. To promote content you care about, you are using the **association's social media account, not your own**. This means that your promotion is done **anonymously** : no one will be able to match the content you publish to your name.


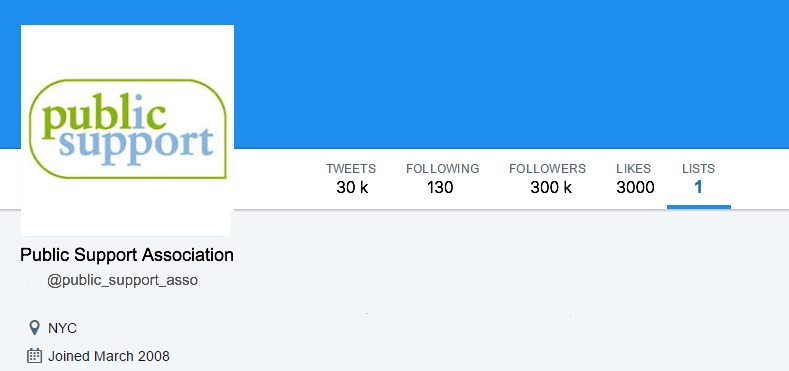


**Page 2**

*Please read very carefully the following instruction (2/2):*

Currently preparing your anonymous promotion, you are about to be presented with a series of news items to potentially share online. For each of them, you are given an endowment of $100, from which you must decide **how much money you are willing to spend in order to share the news (thereby promoting its content)**, or how much to **keep for yourself.**

For instance, if you decide to invest $60 in order to promote a news item on a topic you care about, that means you can keep the remaining $40 for yourself.

Note that the reliability of the news' sources is uncertain. The sources will not be made visible to you.


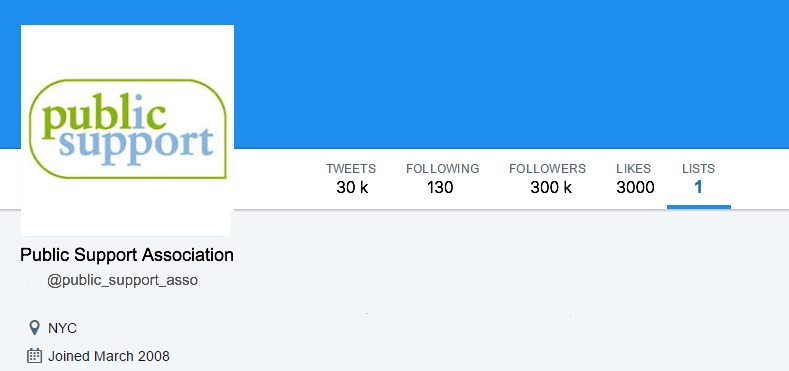


**Personal account condition:**

**Page 1**

*Please read very carefully the following instruction (1/2):*

Imagine you work for an association that gives you a budget for promoting any social cause you care about through sharing news online. The budget the association gives you is **limited**, but allows you to have a social impact on potentially thousands of users through news sharing. To promote content you care about, you are using **your own social media account** (but the money you are investing comes from the association, not your own pocket). This means that all your friends will be able to match the content you publish to your personal identity.

**Page 2**

*Please read very carefully the following instruction (2/2):* Currently preparing your nominative promotion work, you are about to be presented with a series of news items to potentially share online. For each of them, you are given an endowment of $100, from which you must decide how much money you are willing to spend in order to share the news (thereby promoting its content), or how much to keep for yourself. For instance, if you decide to invest $60 in order to promote a news item on a topic you care about, that means you can keep the remaining $40 for yourself. Note that the reliability of the news' sources is uncertain. The sources will not be made visible to you.

In the anonymous account condition, the willingness to pay for sharing question displayed under each item read: “*From a $100 provided by the association, how much would you spend on sharing this news? (from an anonymous social media account, no one will know it's you)*”. In contrast, in the personal account condition, the question read: “*From a $100 provided by the association, how much would you spend on sharing this news item? (from your personal social media account, all your friends will know)*”. Responses were collected using 0-100 slider scales anchored in 0, “*Keep all the money*” and 100, “*Spend all the money*”, with 50 as default slider position.

### Congruence x Issue moralization

| **Pilot Experiment 5a** | | | | | |
| --- | --- | --- | --- | --- | --- |
|  | **W2P** | | **W2P** | | |
| *Predictors* | *ß* | *p* | *ß* | *p* | *std. p* |
| Congruent [TRUE] | 0.41 (0.34,0.49) | **<0.001** | 0.27 (0.19,0.35) | **<0.001** | **<0.001** |
| AbsImpIssue [TRUE] | 0.19 (0.12,0.26) | **<0.001** | -0.02 (-0.11,0.07) | 0.723 | 0.723 |
| Account [Personal account] | -0.04 (-0.20,0.13) | 0.658 | -0.04 (-0.20,0.13) | 0.651 | 0.651 |
| edu | 0.12 (0.04,0.20) | **0.003** | 0.12 (0.04,0.20) | **0.003** | **0.003** |
| sex [female] | -0.11 (-0.28,0.06) | 0.212 | -0.10 (-0.27,0.06) | 0.227 | 0.227 |
| age | 0.02 (-0.07,0.10) | 0.723 | 0.02 (-0.07,0.10) | 0.720 | 0.720 |
| Congruent [TRUE] * AbsImpIssue [TRUE] |  |  | 0.42 (0.30,0.54) | **<0.001** | **<0.001** |
| **Random Effects** | | | | | |
| σ^2^ | 418.69 | | 407.54 | | |
| τ_00_ | 387.01 _id_ | | 395.29 _id_ | | |
|  | 24.74 _News_ | | 29.04 _News_ | | |
| τ_11_ | 115.42 _id.CongruentTRUE_ | | 111.87 _id.CongruentTRUE_ | | |
|  | 28.06 _id.AbsImpIssueTRUE_ | | 34.67 _id.AbsImpIssueTRUE_ | | |
|  | 707.39 _id.AccountPersonal account_ | | 453.02 _id.AccountPersonal account_ | | |
| ρ_01_ | -0.08 _id.CongruentTRUE_ | | -0.11 _id.CongruentTRUE_ | | |
|  | -0.41 _id.AbsImpIssueTRUE_ | | -0.39 _id.AbsImpIssueTRUE_ | | |
|  | -0.57 _id.AccountPersonal account_ | | -0.40 _id.AccountPersonal account_ | | |
| ICC | 0.54 | | 0.55 | | |
| Marginal R^2^ / Conditional R^2^ | 0.068 / 0.570 | | 0.080 / 0.585 | | |

### Congruence x Attitude extremity

| **Pilot Experiment 5a** | | | | | |
| --- | --- | --- | --- | --- | --- |
|  | **W2P** | | **W2P** | | |
| *Predictors* | *ß* | *p* | *ß* | *p* | *std. p* |
| Congruent [TRUE] | 0.42 (0.34,0.49) | **<0.001** | 0.41 (0.34,0.48) | 0.301 | **<0.001** |
| Attit Issue extr | 0.04 (-0.00,0.08) | 0.055 | -0.08 (-0.12, -0.03) | **0.002** | **0.002** |
| Account [Personal account] | -0.03 (-0.20,0.14) | 0.718 | -0.03 (-0.20,0.13) | 0.698 | 0.698 |
| edu | 0.13 (0.04,0.21) | **0.003** | 0.13 (0.04,0.21) | **0.003** | **0.003** |
| sex [female] | -0.10 (-0.27,0.06) | 0.223 | -0.11 (-0.28,0.06) | 0.216 | 0.216 |
| age | 0.02 (-0.07,0.10) | 0.695 | 0.02 (-0.07,0.10) | 0.679 | 0.679 |
| Congruent [TRUE] * Attit Issue extr |  |  | 0.23 (0.17,0.29) | **<0.001** | **<0.001** |
| **Random Effects** | | | | | |
| σ^2^ | 429.13 | | 422.17 | | |
| τ_00_ | 442.76 _id_ | | 435.18 _id_ | | |
|  | 26.00 _News_ | | 24.31 _News_ | | |
| τ_11_ | 110.37 _id.CongruentTRUE_ | | 81.81 _id.CongruentTRUE_ | | |
|  | 29.63 _id.Attit_Issue_extr_ | | 34.37 _id.Attit_Issue_extr_ | | |
|  | 413.81 _id.AccountPersonal account_ | | 1068.78 _id.AccountPersonal account_ | | |
| ρ_01_ | -0.01 _id.CongruentTRUE_ | | 0.06 _id.CongruentTRUE_ | | |
|  | -0.61 _id.Attit_Issue_extr_ | | -0.57 _id.Attit_Issue_extr_ | | |
|  | -0.47 _id.AccountPersonal account_ | | -0.79 _id.AccountPersonal account_ | | |
| ICC | 0.53 | | 0.53 | | |
| Marginal R^2^ / Conditional R^2^ | 0.062 / 0.563 | | 0.075 / 0.570 | | |

### Congruence x Issue moralization controlling for Congruence x Attitude extremity

| **Pilot Experiment 5a** | | | |
| --- | --- | --- | --- |
|  | **W2P** | | |
| *Predictors* | *ß* | *p* | *std. p* |
| Account [Personal account] | -0.04 (-0.20,0.13) | 0.640 | 0.640 |
| Congruent [TRUE] | 0.31 (0.23,0.39) | 0.447 | **<0.001** |
| AbsImpIssue [TRUE] | 0.05 (-0.04,0.15) | 0.292 | 0.292 |
| Attit Issue extr | -0.09 (-0.14, -0.04) | **0.001** | **0.001** |
| edu | 0.12 (0.04,0.20) | **0.003** | **0.003** |
| sex [female] | -0.10 (-0.27,0.06) | 0.222 | 0.223 |
| age | 0.02 (-0.07,0.10) | 0.708 | 0.708 |
| Congruent [TRUE] * AbsImpIssue [TRUE] | 0.29 (0.16,0.41) | **<0.001** | **<0.001** |
| Congruent [TRUE] * Attit Issue extr | 0.18 (0.11,0.24) | **<0.001** | **<0.001** |
| **Random Effects** | | | |
| σ^2^ | 405.73 | | |
| τ_00_ _id_ | 386.50 | | |
| τ_00_ _News_ | 26.18 | | |
| τ_11_ _id.CongruentTRUE_ | 90.19 | | |
| τ_11_ _id.AbsImpIssueTRUE_ | 36.73 | | |
| τ_11_ _id.AccountPersonal account_ | 530.27 | | |
| ρ_01_ _id.CongruentTRUE_ | -0.04 | | |
| ρ_01_ _id.AbsImpIssueTRUE_ | -0.35 | | |
| ρ_01_ _id.AccountPersonal account_ | -0.47 | | |
| ICC | 0.55 | | |
| Marginal R^2^ / Conditional R^2^ | 0.085 / 0.585 | | |

### Congruence x Account

| **Pilot Experiment 5a** | | | |
| --- | --- | --- | --- |
|  | **W2P** | | |
| *Predictors* | *ß* | *p* | *std. p* |
| Congruent [TRUE] | 0.45 (0.35,0.55) | **<0.001** | **<0.001** |
| Account [Personal account] | -0.01 (-0.19,0.16) | 0.896 | 0.896 |
| edu | 0.13 (0.05,0.21) | **0.002** | **0.002** |
| sex [female] | -0.10 (-0.27,0.07) | 0.251 | 0.251 |
| age | 0.02 (-0.07,0.10) | 0.669 | 0.669 |
| Congruent [TRUE] * Account [Personal account] | -0.07 (-0.20,0.07) | 0.321 | 0.321 |
| **Random Effects** | | | |
| σ^2^ | 431.96 | | |
| τ_00_ _id_ | 359.75 | | |
| τ_00_ _News_ | 25.94 | | |
| τ_11_ _id.CongruentTRUE_ | 108.91 | | |
| τ_11_ _id.AccountPersonal account_ | 523.02 | | |
| ρ_01_ _id.CongruentTRUE_ | 0.03 | | |
| ρ_01_ _id.AccountPersonal account_ | -0.45 | | |
| ICC | 0.53 | | |
| Marginal R^2^ / Conditional R^2^ | 0.061 / 0.560 | | |

### Congruence x Account on Democrats only (restricted analyses)

| **Pilot Experiment 5a - Democrats only** | | | | |
| --- | --- | --- | --- | --- |
|  | **W2P** | | **W2P** | |
| *Predictors* | *ß* | *p* | *ß* | *p* |
| Account [Personal account] | -0.03 (-0.23,0.18) | 0.792 | -0.06 (-0.27,0.15) | 0.557 |
| edu | 0.02 (-0.08,0.12) | 0.725 | 0.01 (-0.09,0.12) | 0.773 |
| sex [female] | -0.16 (-0.37,0.05) | 0.130 | -0.17 (-0.38,0.04) | 0.111 |
| age | -0.06 (-0.16,0.05) | 0.273 | -0.06 (-0.16,0.04) | 0.255 |
| Congruent [TRUE] |  |  | 0.50 (0.35,0.66) | **<0.001** |
| Congruent [TRUE] * Account [Personal account] |  |  | -0.09 (-0.28,0.10) | 0.350 |
| **Random Effects** | | | | |
| σ^2^ | 430.55 | | 423.33 | |
| τ_00_ | 253.48 _id_ | | 250.85 _id_ | |
|  | 26.43 _News_ | | 22.95 _News_ | |
| τ_11_ | 185.80 _id.CongruentTRUE_ | | 108.66 _id.CongruentTRUE_ | |
|  | 109.28 _id.AccountPersonal account_ | | 731.73 _id.AccountPersonal account_ | |
| ρ_01_ | 0.21 _id.CongruentTRUE_ | | 0.36 _id.CongruentTRUE_ | |
|  | -0.17 _id.AccountPersonal account_ | | -0.78 _id.AccountPersonal account_ | |
| ICC | 0.42 | | 0.48 | |
| Marginal R^2^ / Conditional R^2^ | 0.013 / 0.426 | | 0.065 / 0.510 | |

### Congruence x Account on Republicans only (restricted analyses)

| **Pilot Experiment 5a - Republicans only** | | | | |
| --- | --- | --- | --- | --- |
|  | **W2P** | | **W2P** | |
| *Predictors* | *ß* | *p* | *ß* | *p* |
| Account [Personal account] | 0.02 (-0.23,0.28) | 0.855 | 0.12 (-0.14,0.38) | 0.366 |
| edu | 0.25 (0.13,0.38) | **<0.001** | 0.25 (0.13,0.38) | **<0.001** |
| sex [female] | 0.02 (-0.24,0.29) | 0.852 | 0.02 (-0.24,0.28) | 0.865 |
| age | 0.06 (-0.07,0.19) | 0.368 | 0.06 (-0.07,0.19) | 0.362 |
| Congruent [TRUE] |  |  | 0.31 (0.19,0.44) | **<0.001** |
| Congruent [TRUE] * Account [Personal account] |  |  | -0.07 (-0.25,0.12) | 0.486 |
| **Random Effects** | | | | |
| σ^2^ | 434.78 | | 434.65 | |
| τ_00_ | 429.13 _id_ | | 416.90 _id_ | |
|  | 47.43 _News_ | | 48.93 _News_ | |
| τ_11_ | 156.33 _id.CongruentTRUE_ | | 74.64 _id.CongruentTRUE_ | |
|  | 450.05 _id.AccountPersonal account_ | | 169.39 _id.AccountPersonal account_ | |
| ρ_01_ | -0.09 _id.CongruentTRUE_ | | 0.04 _id.CongruentTRUE_ | |
|  | -0.45 _id.AccountPersonal account_ | | -0.20 _id.AccountPersonal account_ | |
| ICC | 0.53 | | 0.57 | |
| Marginal R^2^ / Conditional R^2^ | 0.066 / 0.565 | | 0.083 / 0.606 | |

### Congruence x Issue moralization x Account

| **Pilot Experiment 5a** | | |
| --- | --- | --- |
|  | **W2P** | |
| *Predictors* | *ß* | *p* |
| Congruent [TRUE] | 0.29 (0.18,0.40) | **<0.001** |
| AbsImpIssue [TRUE] | -0.02 (-0.14,0.11) | 0.791 |
| Account [Personal account] | -0.01 (-0.20,0.18) | 0.908 |
| edu | 0.12 (0.04,0.20) | **0.003** |
| sex [female] | -0.10 (-0.27,0.07) | 0.230 |
| age | 0.02 (-0.07,0.10) | 0.715 |
| Congruent [TRUE] * AbsImpIssue [TRUE] | 0.46 (0.29,0.63) | **<0.001** |
| Congruent [TRUE] * Account [Personal account] | -0.04 (-0.19,0.12) | 0.641 |
| AbsImpIssue [TRUE] * Account [Personal account] | 0.00 (-0.18,0.18) | 0.990 |
| (Congruent [TRUE] * AbsImpIssue [TRUE]) * Account [Personal account] | -0.08 (-0.31,0.16) | 0.520 |
| **Random Effects** | | |
| σ^2^ | 408.08 | |
| τ_00_ _id_ | 395.48 | |
| τ_00_ _News_ | 28.93 | |
| τ_11_ _id.CongruentTRUE_ | 110.45 | |
| τ_11_ _id.AbsImpIssueTRUE_ | 35.02 | |
| τ_11_ _id.AccountPersonal account_ | 504.08 | |
| ρ_01_ _id.CongruentTRUE_ | -0.11 | |
| ρ_01_ _id.AbsImpIssueTRUE_ | -0.39 | |
| ρ_01_ _id.AccountPersonal account_ | -0.44 | |
| ICC | 0.55 | |
| Marginal R^2^ / Conditional R^2^ | 0.080 / 0.584 | |

### Congruence x Attitude extremity x Account

| **Pilot Experiment 5a** | | | |
| --- | --- | --- | --- |
|  | **W2P** | | |
| *Predictors* | *ß* | *p* | *std. p* |
| Congruent [TRUE] | 0.43 (0.34,0.52) | 0.282 | **<0.001** |
| Attit Issue extr | -0.10 (-0.17, -0.03) | **0.007** | **0.007** |
| Account [Personal account] | -0.02 (-0.19,0.15) | 0.449 | 0.838 |
| edu | 0.13 (0.04,0.21) | **0.003** | **0.003** |
| sex [female] | -0.11 (-0.27,0.06) | 0.222 | 0.222 |
| age | 0.02 (-0.07,0.10) | 0.676 | 0.676 |
| Congruent [TRUE] * Attit Issue extr | 0.26 (0.18,0.35) | **<0.001** | **<0.001** |
| Congruent [TRUE] * Account [Personal account] | -0.04 (-0.17,0.09) | 0.613 | 0.526 |
| Attit Issue extr * Account [Personal account] | 0.04 (-0.05,0.14) | 0.407 | 0.407 |
| (Congruent [TRUE] * Attit Issue extr) * Account [Personal account] | -0.05 (-0.17,0.06) | 0.365 | 0.365 |
| **Random Effects** | | | |
| σ^2^ | 422.49 | | |
| τ_00_ _id_ | 437.13 | | |
| τ_00_ _News_ | 24.08 | | |
| τ_11_ _id.CongruentTRUE_ | 81.51 | | |
| τ_11_ _id.Attit_Issue_extr_ | 35.34 | | |
| τ_11_ _id.AccountPersonal account_ | 1152.90 | | |
| ρ_01_ _id.CongruentTRUE_ | 0.07 | | |
| ρ_01_ _id.Attit_Issue_extr_ | -0.57 | | |
| ρ_01_ _id.AccountPersonal account_ | -0.82 | | |
| ICC | 0.53 | | |
| Marginal R^2^ / Conditional R^2^ | 0.075 / 0.570 | | |

## Final Experiment 5b (fake news)

### Manipulation check

“*In this survey, we asked you to imagine that you were: sharing news from an anonymous account / from your personal account / from your company or organisation’s account*”, choices randomized.

### Congruence x Issue moralization

| **Experiment 5b - fake news** | | | | | |
| --- | --- | --- | --- | --- | --- |
|  | **sharing** | | | **sharing** | |
| *Predictors* | *ß* | *p* | *std. p* | *ß* | *p* |
| Congruent [TRUE] | 0.40 (0.34,0.46) | **<0.001** | **<0.001** | 0.24 (0.18,0.31) | **<0.001** |
| AbsImpIssue [TRUE] | 0.14 (0.08,0.20) | **<0.001** | **<0.001** | -0.05 (-0.12,0.02) | 0.146 |
| Account [personal account] | -0.04 (-0.13,0.05) | 0.413 | 0.413 | -0.04 (-0.13,0.05) | 0.388 |
| edu | -0.04 (-0.09,0.00) | 0.067 | 0.067 | -0.04 (-0.09,0.00) | 0.066 |
| sex [female] | -0.03 (-0.12,0.06) | 0.491 | 0.491 | -0.02 (-0.12,0.07) | 0.609 |
| age | -0.01 (-0.06,0.04) | 0.642 | 0.642 | -0.01 (-0.06,0.03) | 0.618 |
| Congruent [TRUE] * AbsImpIssue [TRUE] |  |  |  | 0.42 (0.33,0.51) | **<0.001** |
| **Random Effects** | | | | | |
| σ^2^ | 0.46 | | | 0.45 | |
| τ_00_ | 0.31 _id_ | | | 0.32 _id_ | |
|  | 0.03 _News_ | | | 0.02 _News_ | |
| τ_11_ | 0.16 _id.CongruentTRUE_ | | | 0.15 _id.CongruentTRUE_ | |
|  | 0.09 _id.AbsImpIssueTRUE_ | | | 0.09 _id.AbsImpIssueTRUE_ | |
|  | 0.79 _id.Accountpersonal account_ | | | 0.96 _id.Accountpersonal account_ | |
| ρ_01_ | 0.20 _id.CongruentTRUE_ | | | 0.21 _id.CongruentTRUE_ | |
|  | -0.30 _id.AbsImpIssueTRUE_ | | | -0.32 _id.AbsImpIssueTRUE_ | |
|  | -0.82 _id.Accountpersonal account_ | | | -0.89 _id.Accountpersonal account_ | |
| ICC | 0.49 | | | 0.49 | |
| Marginal R^2^ / Conditional R^2^ | 0.048 / 0.510 | | | 0.069 / 0.522 | |

### Congruence x Attitude extremity

| **Experiment 5b - fake news** | | | | | | |
| --- | --- | --- | --- | --- | --- | --- |
|  | **sharing** | | | **sharing** | | |
| *Predictors* | *ß* | *p* | *std. p* | *ß* | *p* | *std. p* |
| Congruent [TRUE] | 0.40 (0.34,0.46) | **<0.001** | **<0.001** | 0.46 (0.40,0.52) | **0.010** | **<0.001** |
| Attit Issue extr | 0.03 (0.00,0.06) | **0.037** | **0.037** | -0.09 (-0.13, -0.05) | **<0.001** | **<0.001** |
| Account [personal account] | -0.04 (-0.13,0.05) | 0.426 | 0.426 | -0.04 (-0.13,0.05) | 0.420 | 0.420 |
| edu | -0.04 (-0.09,0.00) | 0.059 | 0.059 | -0.05 (-0.09,0.00) | 0.056 | 0.056 |
| sex [female] | -0.01 (-0.11,0.08) | 0.770 | 0.770 | -0.01 (-0.10,0.09) | 0.890 | 0.890 |
| age | -0.01 (-0.06,0.03) | 0.566 | 0.566 | -0.01 (-0.06,0.04) | 0.656 | 0.656 |
| Congruent [TRUE] * Attit Issue extr |  |  |  | 0.26 (0.22,0.31) | **<0.001** | **<0.001** |
| **Random Effects** | | | | | | |
| σ^2^ | 0.47 | | | 0.46 | | |
| τ_00_ | 0.40 _id_ | | | 0.41 _id_ | | |
|  | 0.02 _News_ | | | 0.02 _News_ | | |
| τ_11_ | 0.15 _id.CongruentTRUE_ | | | 0.13 _id.CongruentTRUE_ | | |
|  | 0.07 _id.Attit_Issue_extr_ | | | 0.09 _id.Attit_Issue_extr_ | | |
|  | 0.07 _id.Accountpersonal account_ | | | 0.08 _id.Accountpersonal account_ | | |
| ρ_01_ | 0.04 _id.CongruentTRUE_ | | | 0.07 _id.CongruentTRUE_ | | |
|  | -0.62 _id.Attit_Issue_extr_ | | | -0.62 _id.Attit_Issue_extr_ | | |
|  | -0.29 _id.Accountpersonal account_ | | | -0.29 _id.Accountpersonal account_ | | |
| ICC | 0.47 | | | 0.47 | | |
| Marginal R^2^ / Conditional R^2^ | 0.044 / 0.496 | | | 0.074 / 0.513 | | |

### Congruence x Issue moralization controlling for Congruence x Attitude extremity

| **Experiment 5b - fake news** | | | |
| --- | --- | --- | --- |
|  | **sharing** | | |
| *Predictors* | *ß* | *p* | *std. p* |
| Account [personal account] | -0.03 (-0.12,0.06) | 0.459 | 0.459 |
| Congruent [TRUE] | 0.35 (0.28,0.42) | **0.012** | **<0.001** |
| AbsImpIssue [TRUE] | -0.00 (-0.08,0.07) | 0.920 | 0.920 |
| Attit Issue extr | -0.09 (-0.13, -0.05) | **<0.001** | **<0.001** |
| edu | -0.04 (-0.09,0.00) | 0.071 | 0.071 |
| sex [female] | -0.02 (-0.11,0.07) | 0.693 | 0.693 |
| age | -0.01 (-0.06,0.03) | 0.628 | 0.627 |
| Congruent [TRUE] * AbsImpIssue [TRUE] | 0.27 (0.18,0.37) | **<0.001** | **<0.001** |
| Congruent [TRUE] * Attit Issue extr | 0.21 (0.16,0.26) | **<0.001** | **<0.001** |
| **Random Effects** | | | |
| σ^2^ | 0.44 | | |
| τ_00_ _id_ | 0.39 | | |
| τ_00_ _News_ | 0.02 | | |
| τ_11_ _id.Accountpersonal account_ | 0.82 | | |
| τ_11_ _id.CongruentTRUE_ | 0.14 | | |
| τ_11_ _id.AbsImpIssueTRUE_ | 0.14 | | |
| τ_11_ _id.Attit_Issue_extr_ | 0.16 | | |
| ρ_01_ _id.Accountpersonal account_ | -0.76 | | |
| ρ_01_ _id.CongruentTRUE_ | 0.00 | | |
| ρ_01_ _id.AbsImpIssueTRUE_ | 0.10 | | |
| ρ_01_ _id.Attit_Issue_extr_ | -0.45 | | |
| ICC | 0.49 | | |
| Marginal R^2^ / Conditional R^2^ | 0.087 / 0.537 | | |

### Congruence x Account

| **Experiment 5b - fake news** | | | |
| --- | --- | --- | --- |
|  | **sharing** | | |
| *Predictors* | *ß* | *p* | *std. p* |
| Congruent [TRUE] | 0.42 (0.34,0.50) | **<0.001** | **<0.001** |
| Account [personal account] | -0.03 (-0.12,0.07) | 0.547 | 0.547 |
| edu | -0.04 (-0.09,0.00) | 0.067 | 0.067 |
| sex [female] | -0.01 (-0.10,0.08) | 0.796 | 0.796 |
| age | -0.01 (-0.06,0.03) | 0.619 | 0.619 |
| Congruent [TRUE] * Account [personal account] | -0.05 (-0.14,0.05) | 0.363 | 0.363 |
| **Random Effects** | | | |
| σ^2^ | 0.48 | | |
| τ_00_ _id_ | 0.28 | | |
| τ_00_ _News_ | 0.02 | | |
| τ_11_ _id.CongruentTRUE_ | 0.15 | | |
| τ_11_ _id.Accountpersonal account_ | 0.43 | | |
| ρ_01_ _id.CongruentTRUE_ | 0.25 | | |
| ρ_01_ _id.Accountpersonal account_ | -0.61 | | |
| ICC | 0.47 | | |
| Marginal R^2^ / Conditional R^2^ | 0.043 / 0.490 | | |

### Effects of partisanship

| **Experiment 5b - fake news** | | | | | | | | | | | | | | | |
| --- | --- | --- | --- | --- | --- | --- | --- | --- | --- | --- | --- | --- | --- | --- | --- |
|  | **sharing** | | **sharing** | | | **sharing** | | **sharing** | | **sharing** | | | **sharing** | | |
| *Predictors* | *ß* | *p* | *ß* | *p* | *std. p* | *ß* | *p* | *ß* | *p* | *ß* | *p* | *std. p* | *ß* | *p* | *std. p* |
| Account [personal account] | -0.06 (-0.15,0.04) | 0.239 | -0.01 (-0.14,0.11) | 0.809 | 0.809 |  |  |  |  | -0.03 (-0.12,0.07) | 0.547 | 0.547 | -0.04 (-0.17,0.10) | 0.605 | 0.605 |
| edu | -0.04 (-0.09,0.01) | 0.087 | -0.04 (-0.09,0.01) | 0.130 | 0.130 | -0.04 (-0.09,0.01) | 0.091 | -0.04 (-0.09,0.01) | 0.126 | -0.04 (-0.09,0.00) | 0.067 | 0.067 | -0.04 (-0.09,0.01) | 0.130 | 0.130 |
| sex [female] | 0.02 (-0.07,0.11) | 0.677 | 0.03 (-0.07,0.12) | 0.553 | 0.553 | 0.02 (-0.07,0.11) | 0.653 | 0.03 (-0.06,0.12) | 0.540 | -0.01 (-0.10,0.08) | 0.796 | 0.796 | 0.03 (-0.07,0.12) | 0.553 | 0.553 |
| age | -0.02 (-0.07,0.02) | 0.345 | -0.03 (-0.08,0.02) | 0.244 | 0.244 | -0.02 (-0.07,0.03) | 0.357 | -0.03 (-0.08,0.02) | 0.247 | -0.01 (-0.06,0.03) | 0.619 | 0.619 | -0.03 (-0.08,0.02) | 0.244 | 0.244 |
| PO bin [Republican] |  |  | 0.16 (-0.01,0.33) | 0.058 | 0.058 |  |  | 0.28 (0.15,0.42) | **<0.001** |  |  |  | 0.26 (0.07,0.44) | **0.006** | **0.006** |
| PO bin [Independent] |  |  | 0.05 (-0.13,0.24) | 0.555 | 0.555 |  |  | 0.19 (0.05,0.32) | **0.007** |  |  |  | 0.20 (0.01,0.40) | **0.043** | **0.043** |
| Account [personal account] * PO bin [Republican] |  |  | -0.09 (-0.33,0.15) | 0.449 | 0.449 |  |  |  |  |  |  |  | 0.04 (-0.22,0.30) | 0.775 | 0.775 |
| Account [personal account] * PO bin [Independent] |  |  | -0.08 (-0.33,0.17) | 0.523 | 0.523 |  |  |  |  |  |  |  | -0.03 (-0.30,0.24) | 0.804 | 0.804 |
| Congruent [TRUE] |  |  |  |  |  | 0.38 (0.33,0.44) | **<0.001** | 0.57 (0.50,0.65) | **<0.001** | 0.42 (0.34,0.50) | **<0.001** | **<0.001** | 0.55 (0.46,0.64) | **<0.001** | **<0.001** |
| Congruent [TRUE] * PO bin [Republican] |  |  |  |  |  |  |  | -0.33 (-0.44, -0.21) | **<0.001** |  |  |  | -0.19 (-0.35, -0.04) | **0.016** | **0.016** |
| Congruent [TRUE] * PO bin [Independent] |  |  |  |  |  |  |  | -0.35 (-0.45, -0.24) | **<0.001** |  |  |  | -0.30 (-0.45, -0.14) | **<0.001** | **<0.001** |
| Congruent [TRUE] * Account [personal account] |  |  |  |  |  |  |  |  |  | -0.05 (-0.14,0.05) | 0.363 | 0.363 | 0.04 (-0.07,0.15) | 0.475 | 0.475 |
| (Congruent [TRUE] * Account [personal account]) * PO bin [Republican] |  |  |  |  |  |  |  |  |  |  |  |  | -0.26 (-0.47, -0.05) | **0.015** | **0.015** |
| (Congruent [TRUE] * Account [personal account]) * PO bin [Independent] |  |  |  |  |  |  |  |  |  |  |  |  | -0.09 (-0.31,0.12) | 0.391 | 0.391 |
| **Random Effects** | | | | | | | | | | | | | | | |
| σ^2^ | 0.55 | | 0.55 | | | 0.53 | | 0.52 | | 0.48 | | | 0.52 | | |
| τ_00_ | 0.37 _id_ | | 0.37 _id_ | | | 0.37 _id_ | | 0.37 _id_ | | 0.28 _id_ | | | 0.37 _id_ | | |
|  | 0.06 _News_ | | 0.06 _News_ | | | 0.02 _News_ | | 0.02 _News_ | | 0.02 _News_ | | | 0.02 _News_ | | |
| τ_11_ | 0.27 _id.PO_binRepublican_ | | 0.14 _id.PO_binRepublican_ | | | 0.11 _id.PO_binRepublican_ | | 0.08 _id.PO_binRepublican_ | | 0.15 _id.CongruentTRUE_ | | | 0.16 _id.PO_binRepublican_ | | |
|  | 0.12 _id.PO_binIndependent_ | | 0.44 _id.PO_binIndependent_ | | | 0.17 _id.PO_binIndependent_ | | 0.20 _id.PO_binIndependent_ | | 0.43 _id.Accountpersonal account_ | | | 0.16 _id.PO_binIndependent_ | | |
|  | 1.01 _id.Accountpersonal account_ | | 0.85 _id.Accountpersonal account_ | | | 1.00 _id.Accountpersonal account_ | | 1.20 _id.Accountpersonal account_ | |  | | | 1.18 _id.Accountpersonal account_ | | |
| ρ_01_ | -0.50 _id.PO_binRepublican_ | | -0.42 _id.PO_binRepublican_ | | | -0.36 _id.PO_binRepublican_ | | -0.38 _id.PO_binRepublican_ | | 0.25 _id.CongruentTRUE_ | | | -0.43 _id.PO_binRepublican_ | | |
|  | -0.28 _id.PO_binIndependent_ | | -0.53 _id.PO_binIndependent_ | | | -0.33 _id.PO_binIndependent_ | | -0.34 _id.PO_binIndependent_ | | -0.61 _id.Accountpersonal account_ | | | -0.31 _id.PO_binIndependent_ | | |
|  | -0.91 _id.Accountpersonal account_ | | -0.85 _id.Accountpersonal account_ | | | -0.90 _id.Accountpersonal account_ | | -0.98 _id.Accountpersonal account_ | |  | | | -0.97 _id.Accountpersonal account_ | | |
| ICC | 0.41 | | 0.43 | | | 0.43 | | 0.43 | | 0.47 | | | 0.42 | | |
| Marginal R^2^ / Conditional R^2^ | 0.003 / 0.410 | | 0.006 / 0.431 | | | 0.039 / 0.453 | | 0.057 / 0.459 | | 0.043 / 0.490 | | | 0.059 / 0.454 | | |

### Congruence x Account on Democrats only (restricted analyses)

| **Experiment 5b - fake news - Democrats only** | | | | |
| --- | --- | --- | --- | --- |
|  | **sharing** | | **sharing** | |
| *Predictors* | *ß* | *p* | *ß* | *p* |
| Account [personal account] | -0.02 (-0.14,0.09) | 0.696 | -0.04 (-0.15,0.08) | 0.540 |
| edu | -0.07 (-0.13, -0.01) | **0.017** | -0.07 (-0.13, -0.01) | **0.014** |
| sex [female] | 0.01 (-0.11,0.12) | 0.920 | -0.00 (-0.12,0.11) | 0.939 |
| age | 0.00 (-0.05,0.06) | 0.884 | 0.00 (-0.05,0.06) | 0.911 |
| Congruent [TRUE] |  |  | 0.35 (0.22,0.48) | **<0.001** |
| Congruent [TRUE] * Account [personal account] |  |  | 0.03 (-0.10,0.17) | 0.614 |
| **Random Effects** | | | | |
| σ^2^ | 0.42 | | 0.42 | |
| τ_00_ | 0.28 _id_ | | 0.28 _id_ | |
|  | 0.12 _News_ | | 0.05 _News_ | |
| τ_11_ | 0.21 _id.CongruentTRUE_ | | 0.21 _id.CongruentTRUE_ | |
|  | 0.60 _id.Accountpersonal account_ | | 0.31 _id.Accountpersonal account_ | |
| ρ_01_ | 0.28 _id.CongruentTRUE_ | | 0.27 _id.CongruentTRUE_ | |
|  | -0.85 _id.Accountpersonal account_ | | -0.68 _id.Accountpersonal account_ | |
| ICC | 0.46 | | 0.52 | |
| Marginal R^2^ / Conditional R^2^ | 0.006 / 0.459 | | 0.040 / 0.535 | |

### Congruence x Account on Republicans only (restricted analyses)

| **Experiment 5b - fake news - Republicans only** | | | | | |
| --- | --- | --- | --- | --- | --- |
|  | **sharing** | | **sharing** | | |
| *Predictors* | *ß* | *p* | *ß* | *p* | *std. p* |
| Account [personal account] | -0.07 (-0.26,0.13) | 0.504 | 0.01 (-0.19,0.21) | 0.922 | 0.922 |
| edu | 0.08 (-0.02,0.17) | 0.124 | 0.08 (-0.02,0.17) | 0.120 | 0.120 |
| sex [female] | -0.05 (-0.25,0.14) | 0.577 | -0.06 (-0.25,0.14) | 0.562 | 0.563 |
| age | -0.06 (-0.16,0.03) | 0.199 | -0.06 (-0.16,0.03) | 0.201 | 0.201 |
| Congruent [TRUE] |  |  | 0.28 (0.14,0.42) | **<0.001** | **<0.001** |
| Congruent [TRUE] * Account [personal account] |  |  | -0.22 (-0.41, -0.04) | **0.019** | **0.019** |
| **Random Effects** | | | | | |
| σ^2^ | 0.61 | | 0.60 | | |
| τ_00_ | 0.22 _id_ | | 0.23 _id_ | | |
|  | 0.07 _News_ | | 0.06 _News_ | | |
| τ_11_ | 0.02 _id.CongruentTRUE_ | | 0.01 _id.CongruentTRUE_ | | |
|  | 0.15 _id.Accountpersonal account_ | | 0.07 _id.Accountpersonal account_ | | |
| ρ_01_ | 1.00 _id.CongruentTRUE_ | | 1.00 _id.CongruentTRUE_ | | |
|  | -0.03 _id.Accountpersonal account_ | | 0.26 _id.Accountpersonal account_ | | |
| ICC | 0.38 | |  | | |
| Marginal R^2^ / Conditional R^2^ | 0.010 / 0.384 | | 0.035 / NA | | |

### Congruence x Issue moralization x Account

| **Experiment 5b - fake news** | | | |
| --- | --- | --- | --- |
|  | **sharing** | | |
| *Predictors* | *ß* | *p* | *std. p* |
| Congruent [TRUE] | 0.27 (0.18,0.37) | **<0.001** | **<0.001** |
| AbsImpIssue [TRUE] | -0.11 (-0.21, -0.01) | **0.025** | **0.025** |
| Account [personal account] | -0.09 (-0.21,0.03) | 0.130 | 0.130 |
| edu | -0.04 (-0.09,0.00) | 0.065 | 0.065 |
| sex [female] | -0.03 (-0.12,0.07) | 0.585 | 0.585 |
| age | -0.01 (-0.06,0.03) | 0.593 | 0.593 |
| Congruent [TRUE] * AbsImpIssue [TRUE] | 0.39 (0.27,0.52) | **<0.001** | **<0.001** |
| Congruent [TRUE] * Account [personal account] | -0.05 (-0.18,0.07) | 0.392 | 0.392 |
| AbsImpIssue [TRUE] * Account [personal account] | 0.12 (-0.02,0.26) | 0.086 | 0.086 |
| (Congruent [TRUE] * AbsImpIssue [TRUE]) * Account [personal account] | 0.04 (-0.13,0.21) | 0.619 | 0.619 |
| **Random Effects** | | | |
| σ^2^ | 0.45 | | |
| τ_00_ _id_ | 0.32 | | |
| τ_00_ _News_ | 0.02 | | |
| τ_11_ _id.CongruentTRUE_ | 0.15 | | |
| τ_11_ _id.AbsImpIssueTRUE_ | 0.09 | | |
| τ_11_ _id.Accountpersonal account_ | 1.03 | | |
| ρ_01_ _id.CongruentTRUE_ | 0.21 | | |
| ρ_01_ _id.AbsImpIssueTRUE_ | -0.31 | | |
| ρ_01_ _id.Accountpersonal account_ | -0.92 | | |
| ICC | 0.49 | | |
| Marginal R^2^ / Conditional R^2^ | 0.071 / 0.523 | | |

### Congruence x Attitude extremity x Account

| **Experiment 5b - fake news** | | | |
| --- | --- | --- | --- |
|  | **sharing** | | |
| *Predictors* | *ß* | *p* | *std. p* |
| Congruent [TRUE] | 0.49 (0.41,0.56) | 0.153 | **<0.001** |
| Attit Issue extr | -0.11 (-0.16, -0.06) | **<0.001** | **<0.001** |
| Account [personal account] | -0.03 (-0.12,0.07) | 0.173 | 0.542 |
| edu | -0.05 (-0.09,0.00) | 0.056 | 0.055 |
| sex [female] | -0.01 (-0.10,0.09) | 0.900 | 0.900 |
| age | -0.01 (-0.06,0.04) | 0.691 | 0.703 |
| Congruent [TRUE] * Attit Issue extr | 0.26 (0.20,0.32) | **<0.001** | **<0.001** |
| Congruent [TRUE] * Account [personal account] | -0.05 (-0.14,0.05) | 0.595 | 0.327 |
| Attit Issue extr * Account [personal account] | 0.05 (-0.02,0.12) | 0.204 | 0.197 |
| (Congruent [TRUE] * Attit Issue extr) * Account [personal account] | 0.01 (-0.08,0.09) | 0.900 | 0.897 |
| **Random Effects** | | | |
| σ^2^ | 0.46 | | |
| τ_00_ _id_ | 0.41 | | |
| τ_00_ _News_ | 0.02 | | |
| τ_11_ _id.CongruentTRUE_ | 0.13 | | |
| τ_11_ _id.Attit_Issue_extr_ | 0.09 | | |
| τ_11_ _id.Accountpersonal account_ | 0.12 | | |
| ρ_01_ _id.CongruentTRUE_ | 0.05 | | |
| ρ_01_ _id.Attit_Issue_extr_ | -0.62 | | |
| ρ_01_ _id.Accountpersonal account_ | -0.33 | | |
| ICC | 0.47 | | |
| Marginal R^2^ / Conditional R^2^ | 0.075 / 0.514 | | |

# J. Experiment 6a & 6b: Sharing to like-minded people vs. foes

## Pilot Experiment 6a (true news)

### Congruence x Issue moralization

| **Pilot Experiment 6a** | | | | | | |
| --- | --- | --- | --- | --- | --- | --- |
|  | **sharing** | | | **sharing** | | |
| *Predictors* | *ß* | *p* | *std. p* | *ß* | *p* | *std. p* |
| Congruent [TRUE] | 0.31 (0.22,0.39) | **<0.001** | **<0.001** | 0.24 (0.15,0.34) | **<0.001** | **<0.001** |
| AbsImpIssue [TRUE] | 0.15 (0.06,0.24) | **0.001** | **0.001** | 0.06 (-0.06,0.17) | 0.318 | 0.318 |
| Audience [Disagree] | 0.06 (-0.08,0.21) | 0.400 | 0.401 | 0.06 (-0.08,0.21) | 0.406 | 0.406 |
| edu | 0.10 (0.03,0.17) | **0.005** | **0.005** | 0.10 (0.03,0.17) | **0.005** | **0.005** |
| sex [female] | -0.07 (-0.22,0.07) | 0.334 | 0.334 | -0.07 (-0.22,0.08) | 0.347 | 0.346 |
| age | -0.04 (-0.12,0.03) | 0.242 | 0.242 | -0.04 (-0.12,0.03) | 0.241 | 0.241 |
| Congruent [TRUE] * AbsImpIssue [TRUE] |  |  |  | 0.19 (0.05,0.33) | **0.007** | **0.007** |
| **Random Effects** | | | | | | |
| σ^2^ | 0.14 | | | 0.14 | | |
| τ_00_ | 0.06 _id_ | | | 0.06 _id_ | | |
|  | 0.00 _News_ | | | 0.00 _News_ | | |
| τ_11_ | 0.03 _id.CongruentTRUE_ | | | 0.03 _id.CongruentTRUE_ | | |
|  | 0.04 _id.AbsImpIssueTRUE_ | | | 0.04 _id.AbsImpIssueTRUE_ | | |
|  | 0.25 _id.ConditionDisagree_ | | | 0.23 _id.ConditionDisagree_ | | |
| ρ_01_ | -0.11 _id.CongruentTRUE_ | | | -0.11 _id.CongruentTRUE_ | | |
|  | -0.21 _id.AbsImpIssueTRUE_ | | | -0.22 _id.AbsImpIssueTRUE_ | | |
|  | -0.90 _id.ConditionDisagree_ | | | -0.85 _id.ConditionDisagree_ | | |
| ICC | 0.40 | | | 0.41 | | |
| Marginal R^2^ / Conditional R^2^ | 0.045 / 0.431 | | | 0.048 / 0.435 | | |

### Congruence x Attitude extremity

| **Pilot Experiment 6a** | | | | | |
| --- | --- | --- | --- | --- | --- |
|  | **sharing** | | **sharing** | | |
| *Predictors* | *ß* | *p* | *ß* | *p* | *std. p* |
| Congruent [TRUE] | 0.31 (0.23,0.40) | **<0.001** | 0.31 (0.23,0.40) | 0.727 | **<0.001** |
| Attit Issue extr | 0.07 (0.02,0.11) | **0.003** | 0.00 (-0.05,0.05) | 0.998 | 0.998 |
| Audience [Disagree] | 0.06 (-0.09,0.21) | 0.416 | 0.06 (-0.09,0.21) | 0.416 | 0.416 |
| edu | 0.10 (0.02,0.17) | **0.010** | 0.10 (0.02,0.17) | **0.010** | **0.010** |
| sex [female] | -0.07 (-0.21,0.08) | 0.381 | -0.06 (-0.21,0.08) | 0.397 | 0.397 |
| age | -0.04 (-0.11,0.03) | 0.257 | -0.04 (-0.11,0.03) | 0.266 | 0.266 |
| Congruent [TRUE] * Attit Issue extr |  |  | 0.14 (0.07,0.21) | **<0.001** | **<0.001** |
| **Random Effects** | | | | | |
| σ^2^ | 0.14 | | 0.14 | | |
| τ_00_ | 0.06 _id_ | | 0.06 _id_ | | |
|  | 0.00 _News_ | | 0.00 _News_ | | |
| τ_11_ | 0.03 _id.CongruentTRUE_ | | 0.02 _id.CongruentTRUE_ | | |
|  | 0.02 _id.Attit_Issue_extr_ | | 0.02 _id.Attit_Issue_extr_ | | |
|  | 0.16 _id.ConditionDisagree_ | | 0.09 _id.ConditionDisagree_ | | |
| ρ_01_ | 0.01 _id.CongruentTRUE_ | | -0.01 _id.CongruentTRUE_ | | |
|  | -0.20 _id.Attit_Issue_extr_ | | -0.24 _id.Attit_Issue_extr_ | | |
|  | -0.76 _id.ConditionDisagree_ | | -0.55 _id.ConditionDisagree_ | | |
| ICC | 0.37 | | 0.37 | | |
| Marginal R^2^ / Conditional R^2^ | 0.042 / 0.399 | | 0.048 / 0.401 | | |

### Congruence x Issue moralization controlling for Congruence x Attitude extremity

| **Pilot Experiment 6a** | | | |
| --- | --- | --- | --- |
|  | **sharing** | | |
| *Predictors* | *ß* | *p* | *std. p* |
| Audience [Disagree] | 0.06 (-0.09,0.20) | 0.430 | 0.430 |
| Congruent [TRUE] | 0.28 (0.18,0.37) | 0.737 | **<0.001** |
| AbsImpIssue [TRUE] | 0.07 (-0.05,0.19) | 0.269 | 0.269 |
| Attit Issue extr | -0.02 (-0.07,0.04) | 0.613 | 0.613 |
| edu | 0.10 (0.02,0.17) | **0.009** | **0.009** |
| sex [female] | -0.08 (-0.22,0.07) | 0.301 | 0.302 |
| age | -0.05 (-0.12,0.03) | 0.212 | 0.212 |
| Congruent [TRUE] * AbsImpIssue [TRUE] | 0.11 (-0.04,0.25) | 0.157 | 0.157 |
| Congruent [TRUE] * Attit Issue extr | 0.12 (0.05,0.19) | **0.001** | **0.001** |
| **Random Effects** | | | |
| σ^2^ | 0.14 | | |
| τ_00_ _id_ | 0.07 | | |
| τ_00_ _News_ | 0.00 | | |
| τ_11_ _id.AbsImpIssueTRUE_ | 0.04 | | |
| τ_11_ _id.CongruentTRUE_ | 0.03 | | |
| τ_11_ _id.Attit_Issue_extr_ | 0.02 | | |
| ρ_01_ _id.AbsImpIssueTRUE_ | -0.16 | | |
| ρ_01_ _id.CongruentTRUE_ | -0.12 | | |
| ρ_01_ _id.Attit_Issue_extr_ | -0.14 | | |
| ICC | 0.41 | | |
| Marginal R^2^ / Conditional R^2^ | 0.052 / 0.438 | | |

### Congruence x Audience

| **Pilot Experiment 6a** | | |
| --- | --- | --- |
|  | **sharing** | |
| *Predictors* | *ß* | *p* |
| Congruent [TRUE] | 0.39 (0.28,0.51) | **<0.001** |
| Audience [Disagree] | 0.13 (-0.03,0.28) | 0.118 |
| edu | 0.10 (0.03,0.17) | **0.007** |
| sex [female] | -0.06 (-0.20,0.09) | 0.453 |
| age | -0.04 (-0.12,0.03) | 0.258 |
| Congruent [TRUE] * Audience [Disagree] | -0.17 (-0.32, -0.02) | **0.030** |
| **Random Effects** | | |
| σ^2^ | 0.15 | |
| τ_00_ _id_ | 0.06 | |
| τ_00_ _News_ | 0.00 | |
| τ_11_ _id.CongruentTRUE_ | 0.03 | |
| τ_11_ _id.ConditionDisagree_ | 0.06 | |
| ρ_01_ _id.CongruentTRUE_ | 0.02 | |
| ρ_01_ _id.ConditionDisagree_ | -0.35 | |
| ICC | 0.37 | |
| Marginal R^2^ / Conditional R^2^ | 0.039 / 0.390 | |

### Congruence x Audience on Democrats only (restricted analyses)

| **Pilot Experiment 6a - true news, Democrats only** | | | | |
| --- | --- | --- | --- | --- |
|  | **sharing** | | **sharing** | |
| *Predictors* | *ß* | *p* | *ß* | *p* |
| Audience [Disagree] | 0.03 (-0.18,0.24) | 0.763 | 0.13 (-0.09,0.36) | 0.247 |
| edu | -0.01 (-0.11,0.10) | 0.915 | -0.01 (-0.11,0.10) | 0.902 |
| sex [female] | -0.05 (-0.26,0.16) | 0.645 | -0.05 (-0.26,0.16) | 0.635 |
| age | -0.02 (-0.12,0.09) | 0.729 | -0.02 (-0.12,0.09) | 0.727 |
| Congruent [TRUE] |  |  | 0.44 (0.27,0.61) | **<0.001** |
| Congruent [TRUE] * Audience [Disagree] |  |  | -0.31 (-0.52, -0.09) | **0.005** |
| **Random Effects** | | | | |
| σ^2^ | 0.13 | | 0.13 | |
| τ_00_ | 0.07 _id_ | | 0.07 _id_ | |
|  | 0.00 _News_ | | 0.00 _News_ | |
| τ_11_ | 0.05 _id.CongruentTRUE_ | | 0.04 _id.CongruentTRUE_ | |
|  | 0.22 _id.AudienceDisagree_ | | 0.27 _id.AudienceDisagree_ | |
| ρ_01_ | -0.11 _id.CongruentTRUE_ | | -0.05 _id.CongruentTRUE_ | |
|  | -0.81 _id.AudienceDisagree_ | | -0.92 _id.AudienceDisagree_ | |
| ICC | 0.39 | | 0.42 | |
| Marginal R^2^ / Conditional R^2^ | 0.001 / 0.393 | | 0.027 / 0.432 | |

### Congruence x Audience on Republicans only (restricted analyses)

| **Pilot Experiment 6a - true news, Republicans only** | | | | |
| --- | --- | --- | --- | --- |
|  | **sharing** | | **sharing** | |
| *Predictors* | *ß* | *p* | *ß* | *p* |
| Audience [Disagree] | 0.06 (-0.15,0.27) | 0.564 | 0.05 (-0.17,0.26) | 0.683 |
| edu | 0.19 (0.09,0.30) | **<0.001** | 0.19 (0.08,0.30) | **<0.001** |
| sex [female] | -0.04 (-0.25,0.17) | 0.707 | -0.04 (-0.25,0.16) | 0.674 |
| age | -0.06 (-0.17,0.05) | 0.282 | -0.06 (-0.16,0.05) | 0.301 |
| Congruent [TRUE] |  |  | 0.26 (0.11,0.42) | **0.001** |
| Congruent [TRUE] * Audience [Disagree] |  |  | 0.02 (-0.19,0.23) | 0.850 |
| **Random Effects** | | | | |
| σ^2^ | 0.16 | | 0.16 | |
| τ_00_ | 0.04 _id_ | | 0.04 _id_ | |
|  | 0.00 _News_ | | 0.01 _News_ | |
| τ_11_ | 0.02 _id.CongruentTRUE_ | | 0.01 _id.CongruentTRUE_ | |
|  | 0.16 _id.AudienceDisagree_ | | 0.03 _id.AudienceDisagree_ | |
| ρ_01_ | 0.26 _id.CongruentTRUE_ | | 1.00 _id.CongruentTRUE_ | |
|  | -0.87 _id.AudienceDisagree_ | | -0.19 _id.AudienceDisagree_ | |
| ICC | 0.25 | | 0.30 | |
| Marginal R^2^ / Conditional R^2^ | 0.054 / 0.290 | | 0.066 / 0.347 | |

### Congruence x Issue moralization x Audience

| **Pilot Experiment 6a** | | | |
| --- | --- | --- | --- |
|  | **sharing** | | |
| *Predictors* | *ß* | *p* | *std. p* |
| Congruent [TRUE] | 0.33 (0.19,0.46) | **<0.001** | **<0.001** |
| AbsImpIssue [TRUE] | 0.12 (-0.04,0.28) | 0.140 | 0.140 |
| Audience [Disagree] | 0.16 (-0.01,0.34) | 0.068 | 0.068 |
| edu | 0.10 (0.03,0.17) | **0.005** | **0.005** |
| sex [female] | -0.07 (-0.22,0.07) | 0.329 | 0.329 |
| age | -0.04 (-0.12,0.03) | 0.246 | 0.246 |
| Congruent [TRUE] * AbsImpIssue [TRUE] | 0.19 (-0.01,0.38) | 0.061 | 0.061 |
| Congruent [TRUE] * Audience [Disagree] | -0.16 (-0.35,0.02) | 0.087 | 0.087 |
| AbsImpIssue [TRUE] * Audience [Disagree] | -0.12 (-0.34,0.11) | 0.302 | 0.302 |
| (Congruent [TRUE] * AbsImpIssue [TRUE]) * Audience [Disagree] | -0.00 (-0.28,0.27) | 0.986 | 0.986 |
| **Random Effects** | | | |
| σ^2^ | 0.14 | | |
| τ_00_ _id_ | 0.06 | | |
| τ_00_ _News_ | 0.00 | | |
| τ_11_ _id.CongruentTRUE_ | 0.03 | | |
| τ_11_ _id.ConditionDisagree_ | 0.24 | | |
| τ_11_ _id.AbsImpIssueTRUE_ | 0.04 | | |
| ρ_01_ _id.CongruentTRUE_ | -0.10 | | |
| ρ_01_ _id.ConditionDisagree_ | -0.90 | | |
| ρ_01_ _id.AbsImpIssueTRUE_ | -0.21 | | |
| ICC | 0.40 | | |
| Marginal R^2^ / Conditional R^2^ | 0.049 / 0.434 | | |

### Congruence x Attitude extremity x Audience

| **Pilot Experiment 6a** | | | |
| --- | --- | --- | --- |
|  | **sharing** | | |
| *Predictors* | *ß* | *p* | *std. p* |
| Congruent [TRUE] | 0.40 (0.29,0.51) | 0.533 | **<0.001** |
| Attit Issue extr | 0.01 (-0.06,0.09) | 0.755 | 0.755 |
| Audience [Disagree] | 0.12 (-0.03,0.28) | 0.198 | 0.123 |
| edu | 0.10 (0.02,0.17) | **0.010** | **0.010** |
| sex [female] | -0.06 (-0.21,0.08) | 0.404 | 0.404 |
| age | -0.04 (-0.11,0.03) | 0.270 | 0.270 |
| Congruent [TRUE] * Attit Issue extr | 0.16 (0.06,0.26) | **0.001** | **0.001** |
| Congruent [TRUE] * Audience [Disagree] | -0.17 (-0.32, -0.02) | 0.596 | **0.026** |
| Attit Issue extr * Audience [Disagree] | -0.03 (-0.13,0.08) | 0.644 | 0.644 |
| (Congruent [TRUE] * Attit Issue extr) * Audience [Disagree] | -0.04 (-0.18,0.10) | 0.562 | 0.562 |
| **Random Effects** | | | |
| σ^2^ | 0.14 | | |
| τ_00_ _id_ | 0.06 | | |
| τ_00_ _News_ | 0.00 | | |
| τ_11_ _id.CongruentTRUE_ | 0.02 | | |
| τ_11_ _id.ConditionDisagree_ | 0.24 | | |
| τ_11_ _id.Attit_Issue_extr_ | 0.02 | | |
| ρ_01_ _id.CongruentTRUE_ | 0.00 | | |
| ρ_01_ _id.ConditionDisagree_ | -0.94 | | |
| ρ_01_ _id.Attit_Issue_extr_ | -0.20 | | |
| ICC | 0.37 | | |
| Marginal R^2^ / Conditional R^2^ | 0.049 / 0.400 | | |

## Final Experiment 6b (fake news)

### Manipulation check

Just to double check, we asked you to imagine sharing the news to people who have “*the same position as you on each issue”/”an opposite position from you on each issue” / “no specific position on each issue*”, choices randomized.

### Congruence x Issue moralization

| **Experiment 6b - fake news** | | | | | |
| --- | --- | --- | --- | --- | --- |
|  | **sharing** | | | **sharing** | |
| *Predictors* | *ß* | *p* | *std. p* | *ß* | *p* |
| Congruent [TRUE] | 0.38 (0.32,0.45) | **<0.001** | **<0.001** | 0.27 (0.20,0.34) | **<0.001** |
| AbsImpIssue [TRUE] | 0.18 (0.12,0.23) | **<0.001** | **<0.001** | 0.03 (-0.04,0.10) | 0.425 |
| Audience [opposite position] | -0.06 (-0.15,0.03) | 0.195 | 0.195 | -0.06 (-0.15,0.03) | 0.189 |
| edu | -0.01 (-0.06,0.04) | 0.670 | 0.670 | -0.01 (-0.06,0.04) | 0.671 |
| sex [female] | 0.05 (-0.05,0.14) | 0.338 | 0.338 | 0.05 (-0.05,0.14) | 0.327 |
| age | -0.07 (-0.11, -0.02) | **0.005** | **0.005** | -0.07 (-0.11, -0.02) | **0.005** |
| Congruent [TRUE] * AbsImpIssue [TRUE] |  |  |  | 0.30 (0.21,0.39) | **<0.001** |
| **Random Effects** | | | | | |
| σ^2^ | 0.52 | | | 0.52 | |
| τ_00_ | 0.38 _id_ | | | 0.37 _id_ | |
|  | 0.03 _News_ | | | 0.03 _News_ | |
| τ_11_ | 0.15 _id.CongruentTRUE_ | | | 0.14 _id.CongruentTRUE_ | |
|  | 0.00 _id.AbsImpIssueTRUE_ | | | 0.00 _id.AbsImpIssueTRUE_ | |
|  | 0.82 _id.Audienceopposite position_ | | | 0.82 _id.Audienceopposite position_ | |
| ρ_01_ | -0.11 _id.CongruentTRUE_ | | | -0.09 _id.CongruentTRUE_ | |
|  | -0.89 _id.AbsImpIssueTRUE_ | | | -0.90 _id.AbsImpIssueTRUE_ | |
|  | -0.74 _id.Audienceopposite position_ | | | -0.74 _id.Audienceopposite position_ | |
| ICC | 0.45 | | | 0.45 | |
| Marginal R^2^ / Conditional R^2^ | 0.053 / 0.483 | | | 0.067 / 0.489 | |

### Congruence x Attitude extremity

| **Experiment 6b - fake news** | | | | | | |
| --- | --- | --- | --- | --- | --- | --- |
|  | **sharing** | | | **sharing** | | |
| *Predictors* | *ß* | *p* | *std. p* | *ß* | *p* | *std. p* |
| Congruent [TRUE] | 0.38 (0.32,0.45) | **<0.001** | **<0.001** | 0.45 (0.39,0.51) | **0.028** | **<0.001** |
| Attit Issue extr | 0.05 (0.02,0.08) | **0.001** | **0.001** | -0.07 (-0.11, -0.04) | **<0.001** | **<0.001** |
| Audience [opposite position] | -0.06 (-0.16,0.03) | 0.172 | 0.172 | -0.06 (-0.15,0.03) | 0.180 | 0.180 |
| edu | -0.01 (-0.06,0.03) | 0.596 | 0.595 | -0.01 (-0.06,0.04) | 0.649 | 0.649 |
| sex [female] | 0.06 (-0.04,0.15) | 0.246 | 0.246 | 0.06 (-0.04,0.15) | 0.230 | 0.230 |
| age | -0.07 (-0.12, -0.03) | **0.002** | **0.002** | -0.07 (-0.12, -0.03) | **0.002** | **0.002** |
| Congruent [TRUE] * Attit Issue extr |  |  |  | 0.25 (0.20,0.29) | **<0.001** | **<0.001** |
| **Random Effects** | | | | | | |
| σ^2^ | 0.53 | | | 0.52 | | |
| τ_00_ | 0.42 _id_ | | | 0.39 _id_ | | |
|  | 0.03 _News_ | | | 0.03 _News_ | | |
| τ_11_ | 0.15 _id.CongruentTRUE_ | | | 0.11 _id.CongruentTRUE_ | | |
|  | 0.01 _id.Attit_Issue_extr_ | | | 0.01 _id.Attit_Issue_extr_ | | |
|  | 0.24 _id.Audienceopposite position_ | | | 0.08 _id.Audienceopposite position_ | | |
| ρ_01_ | -0.16 _id.CongruentTRUE_ | | | -0.03 _id.CongruentTRUE_ | | |
|  | -0.77 _id.Attit_Issue_extr_ | | | -0.79 _id.Attit_Issue_extr_ | | |
|  | -0.34 _id.Audienceopposite position_ | | | -0.15 _id.Audienceopposite position_ | | |
| ICC | 0.45 | | | 0.45 | | |
| Marginal R^2^ / Conditional R^2^ | 0.048 / 0.480 | | | 0.076 / 0.491 | | |

### Congruence x Issue moralization controlling for Congruence x Attitude extremity

| **Experiment 6b - fake news** | | | |
| --- | --- | --- | --- |
|  | **sharing** | | |
| *Predictors* | *ß* | *p* | *std. p* |
| Audience [opposite position] | -0.06 (-0.15,0.03) | 0.190 | 0.190 |
| Congruent [TRUE] | 0.39 (0.32,0.46) | **0.029** | **<0.001** |
| AbsImpIssue [TRUE] | 0.10 (0.02,0.17) | **0.014** | **0.014** |
| Attit Issue extr | -0.09 (-0.13, -0.05) | **<0.001** | **<0.001** |
| edu | -0.01 (-0.06,0.04) | 0.631 | 0.631 |
| sex [female] | 0.05 (-0.04,0.14) | 0.303 | 0.303 |
| age | -0.07 (-0.11, -0.02) | **0.005** | **0.005** |
| Congruent [TRUE] * AbsImpIssue [TRUE] | 0.14 (0.04,0.24) | **0.004** | **0.004** |
| Congruent [TRUE] * Attit Issue extr | 0.22 (0.17,0.27) | **<0.001** | **<0.001** |
| **Random Effects** | | | |
| σ^2^ | 0.51 | | |
| τ_00_ _id_ | 0.38 | | |
| τ_00_ _News_ | 0.03 | | |
| τ_11_ _id.CongruentTRUE_ | 0.11 | | |
| τ_11_ _id.Attit_Issue_extr_ | 0.06 | | |
| τ_11_ _id.AbsImpIssueTRUE_ | 0.04 | | |
| τ_11_ _id.Audienceopposite position_ | 0.43 | | |
| ρ_01_ _id.CongruentTRUE_ | -0.01 | | |
| ρ_01_ _id.Attit_Issue_extr_ | -0.23 | | |
| ρ_01_ _id.AbsImpIssueTRUE_ | -0.05 | | |
| ρ_01_ _id.Audienceopposite position_ | -0.50 | | |
| ICC | 0.46 | | |
| Marginal R^2^ / Conditional R^2^ | 0.085 / 0.504 | | |

### Congruence x Audience

| **Experiment 6b - fake news** | | |
| --- | --- | --- |
|  | **sharing** | |
| *Predictors* | *ß* | *p* |
| Congruent [TRUE] | 0.44 (0.36,0.52) | **<0.001** |
| Audience [opposite position] | -0.02 (-0.12,0.08) | 0.694 |
| edu | -0.01 (-0.06,0.04) | 0.714 |
| sex [female] | 0.06 (-0.03,0.15) | 0.202 |
| age | -0.07 (-0.12, -0.03) | **0.002** |
| Congruent [TRUE] * Audience [opposite position] | -0.11 (-0.21, -0.01) | **0.024** |
| **Random Effects** | | |
| σ^2^ | 0.53 | |
| τ_00_ _id_ | 0.34 | |
| τ_00_ _News_ | 0.03 | |
| τ_11_ _id.CongruentTRUE_ | 0.14 | |
| τ_11_ _id.Audienceopposite position_ | 0.82 | |
| ρ_01_ _id.CongruentTRUE_ | -0.05 | |
| ρ_01_ _id.Audienceopposite position_ | -0.75 | |
| ICC | 0.45 | |
| Marginal R^2^ / Conditional R^2^ | 0.047 / 0.474 | |

### Effects of partisanship

| **Experiment 6b - fake news** | | | | | | | | | | | | | | |
| --- | --- | --- | --- | --- | --- | --- | --- | --- | --- | --- | --- | --- | --- | --- |
|  | **sharing** | | **sharing** | | **sharing** | | **sharing** | | | **sharing** | | **sharing** | | |
| *Predictors* | *ß* | *p* | *ß* | *p* | *ß* | *p* | *ß* | *p* | *std. p* | *ß* | *p* | *ß* | *p* | *std. p* |
| Audience [opposite position] | -0.08 (-0.17,0.02) | 0.107 | -0.12 (-0.24,0.00) | 0.057 |  |  |  |  |  | -0.02 (-0.12,0.08) | 0.694 | -0.06 (-0.20,0.07) | 0.338 | 0.338 |
| edu | -0.01 (-0.06,0.04) | 0.664 | -0.01 (-0.06,0.04) | 0.624 | -0.01 (-0.06,0.04) | 0.680 | -0.01 (-0.06,0.04) | 0.663 | 0.663 | -0.01 (-0.06,0.04) | 0.714 | -0.01 (-0.06,0.04) | 0.624 | 0.624 |
| sex [female] | 0.07 (-0.02,0.17) | 0.113 | 0.08 (-0.02,0.17) | 0.107 | 0.08 (-0.02,0.17) | 0.109 | 0.08 (-0.02,0.17) | 0.110 | 0.110 | 0.06 (-0.03,0.15) | 0.202 | 0.08 (-0.02,0.17) | 0.107 | 0.107 |
| age | -0.08 (-0.13, -0.03) | **0.001** | -0.08 (-0.13, -0.04) | **<0.001** | -0.08 (-0.13, -0.03) | **0.001** | -0.08 (-0.13, -0.04) | **0.001** | **0.001** | -0.07 (-0.12, -0.03) | **0.002** | -0.08 (-0.13, -0.04) | **<0.001** | **<0.001** |
| PO bin [Republican] |  |  | 0.03 (-0.15,0.22) | 0.734 |  |  | 0.17 (0.03,0.31) | **0.015** | **0.015** |  |  | 0.16 (-0.04,0.36) | 0.125 | 0.125 |
| PO bin [Independent] |  |  | -0.11 (-0.28,0.06) | 0.195 |  |  | 0.08 (-0.05,0.21) | 0.206 | 0.206 |  |  | 0.02 (-0.16,0.21) | 0.804 | 0.803 |
| Audience [opposite position] * PO bin [Republican] |  |  | 0.01 (-0.23,0.26) | 0.918 |  |  |  |  |  |  |  | 0.04 (-0.23,0.31) | 0.776 | 0.776 |
| Audience [opposite position] * PO bin [Independent] |  |  | 0.17 (-0.07,0.40) | 0.163 |  |  |  |  |  |  |  | 0.12 (-0.13,0.38) | 0.344 | 0.344 |
| Congruent [TRUE] |  |  |  |  | 0.38 (0.32,0.44) | **<0.001** | 0.52 (0.45,0.60) | **<0.001** | **<0.001** | 0.44 (0.36,0.52) | **<0.001** | 0.57 (0.48,0.66) | **<0.001** | **<0.001** |
| Congruent [TRUE] * PO bin [Republican] |  |  |  |  |  |  | -0.29 (-0.41, -0.17) | **<0.001** | **<0.001** |  |  | -0.25 (-0.42, -0.09) | **0.003** | **0.003** |
| Congruent [TRUE] * PO bin [Independent] |  |  |  |  |  |  | -0.23 (-0.33, -0.12) | **<0.001** | **<0.001** |  |  | -0.27 (-0.42, -0.11) | **0.001** | **0.001** |
| Congruent [TRUE] * Audience [opposite position] |  |  |  |  |  |  |  |  |  | -0.11 (-0.21, -0.01) | **0.024** | -0.10 (-0.21,0.01) | 0.069 | 0.069 |
| (Congruent [TRUE] * Audience [opposite position]) * PO bin [Republican] |  |  |  |  |  |  |  |  |  |  |  | -0.05 (-0.27,0.17) | 0.639 | 0.639 |
| (Congruent [TRUE] * Audience [opposite position]) * PO bin [Independent] |  |  |  |  |  |  |  |  |  |  |  | 0.09 (-0.12,0.29) | 0.423 | 0.423 |
| **Random Effects** | | | | | | | | | | | | | | |
| σ^2^ | 0.60 | | 0.60 | | 0.57 | | 0.57 | | | 0.53 | | 0.57 | | |
| τ_00_ | 0.33 _id_ | | 0.33 _id_ | | 0.34 _id_ | | 0.34 _id_ | | | 0.34 _id_ | | 0.33 _id_ | | |
|  | 0.07 _News_ | | 0.07 _News_ | | 0.03 _News_ | | 0.02 _News_ | | | 0.03 _News_ | | 0.02 _News_ | | |
| τ_11_ | 0.07 _id.PO_binRepublican_ | | 0.21 _id.PO_binRepublican_ | | 0.01 _id.PO_binRepublican_ | | 0.01 _id.PO_binRepublican_ | | | 0.14 _id.CongruentTRUE_ | | 0.25 _id.PO_binRepublican_ | | |
|  | 0.07 _id.PO_binIndependent_ | | 0.03 _id.PO_binIndependent_ | | 0.19 _id.PO_binIndependent_ | | 0.26 _id.PO_binIndependent_ | | | 0.82 _id.Audienceopposite position_ | | 0.21 _id.PO_binIndependent_ | | |
|  | 1.26 _id.Audienceopposite position_ | | 1.00 _id.Audienceopposite position_ | | 0.77 _id.Audienceopposite position_ | | 0.74 _id.Audienceopposite position_ | | |  | | 0.83 _id.Audienceopposite position_ | | |
| ρ_01_ | 0.09 _id.PO_binRepublican_ | | -0.21 _id.PO_binRepublican_ | | 1.00 _id.PO_binRepublican_ | | 1.00 _id.PO_binRepublican_ | | | -0.05 _id.CongruentTRUE_ | | -0.26 _id.PO_binRepublican_ | | |
|  | -0.13 _id.PO_binIndependent_ | | -0.02 _id.PO_binIndependent_ | | -0.33 _id.PO_binIndependent_ | | -0.40 _id.PO_binIndependent_ | | | -0.75 _id.Audienceopposite position_ | | -0.34 _id.PO_binIndependent_ | | |
|  | -0.96 _id.Audienceopposite position_ | | -0.85 _id.Audienceopposite position_ | | -0.74 _id.Audienceopposite position_ | | -0.73 _id.Audienceopposite position_ | | |  | | -0.77 _id.Audienceopposite position_ | | |
| ICC | 0.40 | | 0.42 | | 0.39 | |  | | | 0.45 | | 0.40 | | |
| Marginal R^2^ / Conditional R^2^ | 0.010 / 0.410 | | 0.011 / 0.422 | | 0.047 / 0.416 | | 0.091 / NA | | | 0.047 / 0.474 | | 0.060 / 0.440 | | |

### Congruence x Audience on Democrats only (restricted analyses)

| **Experiment 6b - fake news, Democrats only** | | | | |
| --- | --- | --- | --- | --- |
|  | **sharing** | | **sharing** | |
| *Predictors* | *ß* | *p* | *ß* | *p* |
| Audience [opposite position] | -0.10 (-0.22,0.02) | 0.106 | -0.06 (-0.19,0.06) | 0.343 |
| edu | -0.04 (-0.10,0.02) | 0.171 | -0.04 (-0.10,0.02) | 0.156 |
| sex [female] | 0.14 (0.01,0.26) | **0.029** | 0.13 (0.01,0.25) | **0.034** |
| age | -0.07 (-0.13, -0.01) | **0.029** | -0.07 (-0.13, -0.01) | **0.028** |
| Congruent [TRUE] |  |  | 0.28 (0.16,0.41) | **<0.001** |
| Congruent [TRUE] * Audience [opposite position] |  |  | -0.11 (-0.23,0.00) | 0.059 |
| **Random Effects** | | | | |
| σ^2^ | 0.48 | | 0.48 | |
| τ_00_ | 0.30 _id_ | | 0.30 _id_ | |
|  | 0.14 _News_ | | 0.08 _News_ | |
| τ_11_ | 0.11 _id.CongruentTRUE_ | | 0.11 _id.CongruentTRUE_ | |
|  | 0.55 _id.Audienceopposite position_ | | 0.32 _id.Audienceopposite position_ | |
| ρ_01_ | 0.09 _id.CongruentTRUE_ | | 0.09 _id.CongruentTRUE_ | |
|  | -0.66 _id.Audienceopposite position_ | | -0.49 _id.Audienceopposite position_ | |
| ICC | 0.48 | | 0.50 | |
| Marginal R^2^ / Conditional R^2^ | 0.016 / 0.492 | | 0.030 / 0.510 | |

### Congruence x Audience on Republicans only (restricted analyses)

| **Experiment 6b - fake news, Republicans only** | | | | | |
| --- | --- | --- | --- | --- | --- |
|  | **sharing** | | | **sharing** | |
| *Predictors* | *ß* | *p* | *std. p* | *ß* | *p* |
| Audience [opposite position] | -0.13 (-0.33,0.07) | 0.213 | 0.213 | -0.02 (-0.25,0.21) | 0.860 |
| edu | 0.10 (-0.00,0.21) | 0.051 | 0.051 | 0.11 (0.00,0.21) | **0.049** |
| sex [female] | 0.02 (-0.19,0.22) | 0.868 | 0.868 | 0.02 (-0.19,0.22) | 0.874 |
| age | -0.18 (-0.29, -0.08) | **0.001** | **0.001** | -0.18 (-0.29, -0.08) | **0.001** |
| Congruent [TRUE] |  |  |  | 0.27 (0.11,0.44) | **0.001** |
| Congruent [TRUE] * Audience [opposite position] |  |  |  | -0.17 (-0.38,0.05) | 0.127 |
| **Random Effects** | | | | | |
| σ^2^ | 0.61 | | | 0.61 | |
| τ_00_ | 0.48 _id_ | | | 0.47 _id_ | |
|  | 0.08 _News_ | | | 0.07 _News_ | |
| τ_11_ | 0.15 _id.CongruentTRUE_ | | | 0.11 _id.CongruentTRUE_ | |
|  | 1.33 _id.Audienceopposite position_ | | | 1.43 _id.Audienceopposite position_ | |
| ρ_01_ | -0.40 _id.CongruentTRUE_ | | | -0.38 _id.CongruentTRUE_ | |
|  | -0.95 _id.Audienceopposite position_ | | | -0.99 _id.Audienceopposite position_ | |
| ICC | 0.43 | | | 0.44 | |
| Marginal R^2^ / Conditional R^2^ | 0.037 / 0.448 | | | 0.044 / 0.462 | |

### Congruence x Issue moralization x Audience

| **Experiment 6b - fake news** | | | |
| --- | --- | --- | --- |
|  | **sharing** | | |
| *Predictors* | *ß* | *p* | *std. p* |
| Congruent [TRUE] | 0.31 (0.21,0.40) | **<0.001** | **<0.001** |
| AbsImpIssue [TRUE] | 0.06 (-0.03,0.16) | 0.201 | 0.201 |
| Audience [opposite position] | 0.02 (-0.11,0.14) | 0.803 | 0.803 |
| edu | -0.01 (-0.06,0.04) | 0.668 | 0.668 |
| sex [female] | 0.05 (-0.05,0.14) | 0.330 | 0.330 |
| age | -0.07 (-0.11, -0.02) | **0.005** | **0.005** |
| Congruent [TRUE] * AbsImpIssue [TRUE] | 0.34 (0.21,0.46) | **<0.001** | **<0.001** |
| Congruent [TRUE] * Audience [opposite position] | -0.06 (-0.19,0.06) | 0.330 | 0.330 |
| AbsImpIssue [TRUE] * Audience [opposite position] | -0.07 (-0.20,0.07) | 0.331 | 0.331 |
| (Congruent [TRUE] * AbsImpIssue [TRUE]) * Audience [opposite position] | -0.07 (-0.25,0.10) | 0.397 | 0.397 |
| **Random Effects** | | | |
| σ^2^ | 0.52 | | |
| τ_00_ _id_ | 0.37 | | |
| τ_00_ _News_ | 0.03 | | |
| τ_11_ _id.CongruentTRUE_ | 0.13 | | |
| τ_11_ _id.Audienceopposite position_ | 0.70 | | |
| τ_11_ _id.AbsImpIssueTRUE_ | 0.00 | | |
| ρ_01_ _id.CongruentTRUE_ | -0.08 | | |
| ρ_01_ _id.Audienceopposite position_ | -0.69 | | |
| ρ_01_ _id.AbsImpIssueTRUE_ | -0.92 | | |
| Marginal R^2^ / Conditional R^2^ | 0.118 / NA | | |

### Congruence x Attitude extremity x Audience

| **Experiment 6b - fake news** | | | |
| --- | --- | --- | --- |
|  | **sharing** | | |
| *Predictors* | *ß* | *p* | *std. p* |
| Congruent [TRUE] | 0.50 (0.42,0.57) | 0.358 | **<0.001** |
| Attit Issue extr | -0.05 (-0.10,0.01) | 0.078 | 0.078 |
| Audience [opposite position] | -0.02 (-0.12,0.07) | 0.439 | 0.625 |
| edu | -0.01 (-0.06,0.03) | 0.575 | 0.575 |
| sex [female] | 0.06 (-0.04,0.15) | 0.236 | 0.236 |
| age | -0.07 (-0.12, -0.02) | **0.003** | **0.003** |
| Congruent [TRUE] * Attit Issue extr | 0.24 (0.18,0.31) | **<0.001** | **<0.001** |
| Congruent [TRUE] * Audience [opposite position] | -0.10 (-0.19, -0.00) | 0.398 | **0.043** |
| Attit Issue extr * Audience [opposite position] | -0.04 (-0.11,0.03) | 0.223 | 0.223 |
| (Congruent [TRUE] * Attit Issue extr) * Audience [opposite position] | -0.00 (-0.09,0.09) | 0.997 | 0.997 |
| **Random Effects** | | | |
| σ^2^ | 0.51 | | |
| τ_00_ _id_ | 0.40 | | |
| τ_00_ _News_ | 0.03 | | |
| τ_11_ _id.CongruentTRUE_ | 0.11 | | |
| τ_11_ _id.Attit_Issue_extr_ | 0.09 | | |
| τ_11_ _id.AbsImpIssueTRUE_ | 0.05 | | |
| ρ_01_ _id.CongruentTRUE_ | -0.07 | | |
| ρ_01_ _id.Attit_Issue_extr_ | -0.34 | | |
| ρ_01_ _id.AbsImpIssueTRUE_ | 0.16 | | |
| Marginal R^2^ / Conditional R^2^ | 0.134 / NA | | |

# K. Experiment 7: Intervention message on the myside bias, true news

### Congruence x Issue moralization

| **Experiment 7 - true news** | | | | | | |
| --- | --- | --- | --- | --- | --- | --- |
|  | **sharing** | | | **sharing** | | |
| *Predictors* | *ß* | *p* | *std. p* | *ß* | *p* | *std. p* |
| Congruent [TRUE] | 0.36 (0.31,0.41) | **<0.001** | **<0.001** | 0.27 (0.21,0.32) | **<0.001** | **<0.001** |
| AbsImpIssue [TRUE] | 0.25 (0.20,0.30) | **<0.001** | **<0.001** | 0.10 (0.04,0.17) | **0.001** | **0.001** |
| Condition [message myside bias] | -0.08 (-0.16,0.00) | 0.063 | 0.063 | -0.08 (-0.16,0.00) | 0.064 | 0.064 |
| edu | 0.04 (0.00,0.08) | **0.048** | **0.048** | 0.04 (0.00,0.08) | **0.047** | **0.047** |
| sex [female] | 0.03 (-0.05,0.11) | 0.478 | 0.478 | 0.03 (-0.05,0.12) | 0.453 | 0.453 |
| age | -0.06 (-0.11, -0.02) | **0.002** | **0.002** | -0.06 (-0.11, -0.02) | **0.002** | **0.002** |
| Congruent [TRUE] * AbsImpIssue [TRUE] |  |  |  | 0.29 (0.21,0.37) | **<0.001** | **<0.001** |
| **Random Effects** | | | | | | |
| σ^2^ | 0.13 | | | 0.13 | | |
| τ_00_ | 0.07 _id_ | | | 0.07 _id_ | | |
|  | 0.01 _News_ | | | 0.01 _News_ | | |
| τ_11_ | 0.02 _id.CongruentTRUE_ | | | 0.02 _id.CongruentTRUE_ | | |
|  | 0.02 _id.AbsImpIssueTRUE_ | | | 0.02 _id.AbsImpIssueTRUE_ | | |
|  | 0.24 _id.Conditionmessage myside bias_ | | | 0.24 _id.Conditionmessage myside bias_ | | |
| ρ_01_ | -0.14 _id.CongruentTRUE_ | | | -0.16 _id.CongruentTRUE_ | | |
|  | -0.22 _id.AbsImpIssueTRUE_ | | | -0.22 _id.AbsImpIssueTRUE_ | | |
|  | -0.93 _id.Conditionmessage myside bias_ | | | -0.93 _id.Conditionmessage myside bias_ | | |
| ICC | 0.40 | | | 0.40 | | |
| Marginal R^2^ / Conditional R^2^ | 0.053 / 0.430 | | | 0.059 / 0.439 | | |

### Congruence x Attitude extremity

| **Experiment 7 - true news** | | | | | |
| --- | --- | --- | --- | --- | --- |
|  | **sharing** | | **sharing** | | |
| *Predictors* | *ß* | *p* | *ß* | *p* | *std. p* |
| Congruent [TRUE] | 0.36 (0.31,0.41) | **<0.001** | 0.36 (0.32,0.41) | 0.283 | **<0.001** |
| Attit Issue extr | 0.11 (0.08,0.13) | **<0.001** | 0.01 (-0.02,0.04) | 0.531 | 0.531 |
| Condition [message myside bias] | -0.07 (-0.16,0.01) | 0.086 | -0.07 (-0.15,0.01) | 0.093 | 0.093 |
| edu | 0.03 (-0.01,0.08) | 0.101 | 0.03 (-0.01,0.08) | 0.103 | 0.103 |
| sex [female] | 0.03 (-0.06,0.11) | 0.555 | 0.03 (-0.06,0.11) | 0.551 | 0.551 |
| age | -0.07 (-0.11, -0.03) | **0.002** | -0.07 (-0.11, -0.03) | **0.002** | **0.002** |
| Congruent [TRUE] * Attit Issue extr |  |  | 0.20 (0.16,0.23) | **<0.001** | **<0.001** |
| **Random Effects** | | | | | |
| σ^2^ | 0.13 | | 0.13 | | |
| τ_00_ | 0.09 _id_ | | 0.09 _id_ | | |
|  | 0.01 _News_ | | 0.01 _News_ | | |
| τ_11_ | 0.02 _id.CongruentTRUE_ | | 0.02 _id.CongruentTRUE_ | | |
|  | 0.02 _id.Attit_Issue_extr_ | | 0.02 _id.Attit_Issue_extr_ | | |
|  | 0.14 _id.Conditionmessage myside bias_ | | 0.03 _id.Conditionmessage myside bias_ | | |
| ρ_01_ | -0.25 _id.CongruentTRUE_ | | -0.18 _id.CongruentTRUE_ | | |
|  | -0.48 _id.Attit_Issue_extr_ | | -0.47 _id.Attit_Issue_extr_ | | |
|  | -0.70 _id.Conditionmessage myside bias_ | | -0.51 _id.Conditionmessage myside bias_ | | |
| ICC | 0.39 | | 0.39 | | |
| Marginal R^2^ / Conditional R^2^ | 0.049 / 0.418 | | 0.059 / 0.426 | | |

### Congruence x Issue moralization controlling for Congruence x Attitude extremity

| **Experiment 7 - true news** | | | |
| --- | --- | --- | --- |
|  | **sharing** | | |
| *Predictors* | *ß* | *p* | *std. p* |
| Condition [message myside bias] | -0.08 (-0.16,0.00) | 0.062 | 0.062 |
| Congruent [TRUE] | 0.31 (0.26,0.36) | 0.366 | **<0.001** |
| AbsImpIssue [TRUE] | 0.12 (0.05,0.18) | **<0.001** | **<0.001** |
| Attit Issue extr | -0.02 (-0.05,0.01) | 0.281 | 0.281 |
| edu | 0.04 (-0.00,0.08) | 0.072 | 0.072 |
| sex [female] | 0.02 (-0.07,0.10) | 0.698 | 0.698 |
| age | -0.07 (-0.11, -0.03) | **0.002** | **0.002** |
| Congruent [TRUE] * AbsImpIssue [TRUE] | 0.16 (0.08,0.25) | **<0.001** | **<0.001** |
| Congruent [TRUE] * Attit Issue extr | 0.17 (0.13,0.21) | **<0.001** | **<0.001** |
| **Random Effects** | | | |
| σ^2^ | 0.13 | | |
| τ_00_ _id_ | 0.07 | | |
| τ_00_ _News_ | 0.01 | | |
| τ_11_ _id.CongruentTRUE_ | 0.02 | | |
| ρ_01_ _id_ | -0.12 | | |
| ICC | 0.38 | | |
| Marginal R^2^ / Conditional R^2^ | 0.068 / 0.423 | | |

### By issue analyses

| **Experiment 7 true political news - Abortion issue only** | | | | | | | | | | | | |
| --- | --- | --- | --- | --- | --- | --- | --- | --- | --- | --- | --- | --- |
|  | **sharing** | | **sharing** | | **sharing** | | **sharing** | | | **sharing** | | |
| *Predictors* | *ß* | *p* | *ß* | *p* | *ß* | *p* | *ß* | *p* | *std. p* | *ß* | *p* | *std. p* |
| Congruent [TRUE] | 0.40 (0.33,0.48) | **<0.001** | 0.29 (0.20,0.38) | **<0.001** | 0.40 (0.33,0.48) | **<0.001** | 0.41 (0.33,0.48) | 0.724 | **<0.001** | 0.33 (0.24,0.42) | 0.755 | **<0.001** |
| AbsImpIssue [TRUE] | 0.18 (0.06,0.29) | **0.002** | 0.03 (-0.11,0.16) | 0.701 |  |  |  |  |  | 0.05 (-0.10,0.19) | 0.524 | 0.524 |
| Condition [message myside bias] | -0.09 (-0.20,0.01) | 0.085 | -0.09 (-0.20,0.01) | 0.085 | -0.08 (-0.19,0.02) | 0.126 | -0.08 (-0.19,0.02) | 0.126 | 0.126 | -0.09 (-0.20,0.01) | 0.089 | 0.089 |
| edu | 0.05 (-0.00,0.10) | 0.069 | 0.05 (-0.00,0.10) | 0.069 | 0.05 (-0.01,0.10) | 0.086 | 0.05 (-0.01,0.10) | 0.086 | 0.086 | 0.05 (-0.00,0.10) | 0.074 | 0.074 |
| sex [female] | 0.05 (-0.06,0.16) | 0.379 | 0.05 (-0.06,0.16) | 0.379 | 0.06 (-0.05,0.17) | 0.268 | 0.06 (-0.05,0.17) | 0.268 | 0.268 | 0.04 (-0.07,0.16) | 0.432 | 0.432 |
| age | -0.08 (-0.14, -0.03) | **0.003** | -0.08 (-0.14, -0.03) | **0.003** | -0.08 (-0.14, -0.03) | **0.003** | -0.08 (-0.14, -0.03) | **0.003** | **0.003** | -0.08 (-0.14, -0.03) | **0.003** | **0.003** |
| Congruent [TRUE] * AbsImpIssue [TRUE] |  |  | 0.30 (0.15,0.45) | **<0.001** |  |  |  |  |  | 0.20 (0.05,0.36) | **0.011** | **0.011** |
| Attit Issue extr |  |  |  |  | 0.07 (0.01,0.12) | **0.017** | -0.02 (-0.08,0.05) | 0.567 | 0.567 | -0.02 (-0.09,0.04) | 0.478 | 0.478 |
| Congruent [TRUE] * Attit Issue extr |  |  |  |  |  |  | 0.17 (0.10,0.24) | **<0.001** | **<0.001** | 0.13 (0.06,0.21) | **<0.001** | **<0.001** |
| **Random Effects** | | | | | | | | | | | | |
| σ^2^ | 0.12 | | 0.12 | | 0.12 | | 0.12 | | | 0.12 | | |
| τ_00_ | 0.08 _id_ | | 0.08 _id_ | | 0.08 _id_ | | 0.08 _id_ | | | 0.08 _id_ | | |
|  | 0.00 _News_ | | 0.00 _News_ | | 0.00 _News_ | | 0.00 _News_ | | | 0.00 _News_ | | |
| ICC | 0.38 | | 0.39 | | 0.39 | | 0.40 | | | 0.40 | | |
| Marginal R^2^ / Conditional R^2^ | 0.058 / 0.420 | | 0.061 / 0.429 | | 0.055 / 0.420 | | 0.063 / 0.433 | | | 0.068 / 0.437 | | |

| **Experiment 7 true political news - Gender equality issue only** | | | | | | | | | | | | |
| --- | --- | --- | --- | --- | --- | --- | --- | --- | --- | --- | --- | --- |
|  | **sharing** | | **sharing** | | **sharing** | | **sharing** | | | **sharing** | | |
| *Predictors* | *ß* | *p* | *ß* | *p* | *ß* | *p* | *ß* | *p* | *std. p* | *ß* | *p* | *std. p* |
| Congruent [TRUE] | 0.31 (0.22,0.40) | **<0.001** | 0.26 (0.16,0.36) | **<0.001** | 0.31 (0.22,0.40) | **<0.001** | 0.32 (0.23,0.41) | 0.156 | **<0.001** | 0.30 (0.20,0.40) | 0.187 | **<0.001** |
| AbsImpIssue [TRUE] | 0.42 (0.30,0.53) | **<0.001** | 0.29 (0.15,0.43) | **<0.001** |  |  |  |  |  | 0.34 (0.19,0.49) | **<0.001** | **<0.001** |
| Condition [message myside bias] | -0.05 (-0.15,0.06) | 0.389 | -0.05 (-0.15,0.06) | 0.389 | -0.03 (-0.14,0.08) | 0.604 | -0.03 (-0.14,0.08) | 0.604 | 0.604 | -0.04 (-0.15,0.06) | 0.400 | 0.400 |
| edu | 0.04 (-0.02,0.09) | 0.179 | 0.04 (-0.02,0.09) | 0.179 | 0.04 (-0.01,0.10) | 0.114 | 0.04 (-0.01,0.10) | 0.114 | 0.114 | 0.03 (-0.02,0.09) | 0.218 | 0.218 |
| sex [female] | 0.07 (-0.04,0.18) | 0.217 | 0.07 (-0.04,0.18) | 0.217 | 0.11 (-0.01,0.22) | 0.062 | 0.11 (-0.01,0.22) | 0.062 | 0.062 | 0.06 (-0.05,0.17) | 0.318 | 0.318 |
| age | -0.03 (-0.08,0.02) | 0.272 | -0.03 (-0.08,0.02) | 0.272 | -0.04 (-0.10,0.01) | 0.106 | -0.04 (-0.10,0.01) | 0.106 | 0.106 | -0.03 (-0.08,0.02) | 0.266 | 0.266 |
| Congruent [TRUE] * AbsImpIssue [TRUE] |  |  | 0.25 (0.09,0.42) | **0.003** |  |  |  |  |  | 0.10 (-0.07,0.27) | 0.263 | 0.263 |
| Attit Issue extr |  |  |  |  | 0.10 (0.05,0.16) | **<0.001** | -0.01 (-0.07,0.06) | 0.805 | 0.805 | -0.06 (-0.12,0.01) | 0.101 | 0.101 |
| Congruent [TRUE] * Attit Issue extr |  |  |  |  |  |  | 0.22 (0.15,0.29) | **<0.001** | **<0.001** | 0.20 (0.13,0.28) | **<0.001** | **<0.001** |
| **Random Effects** | | | | | | | | | | | | |
| σ^2^ | 0.14 | | 0.14 | | 0.14 | | 0.14 | | | 0.14 | | |
| τ_00_ | 0.07 _id_ | | 0.07 _id_ | | 0.08 _id_ | | 0.08 _id_ | | | 0.07 _id_ | | |
|  | 0.00 _News_ | | 0.01 _News_ | | 0.00 _News_ | | 0.01 _News_ | | | 0.01 _News_ | | |
| ICC | 0.35 | | 0.36 | | 0.37 | | 0.38 | | | 0.37 | | |
| Marginal R^2^ / Conditional R^2^ | 0.067 / 0.392 | | 0.074 / 0.407 | | 0.041 / 0.392 | | 0.054 / 0.417 | | | 0.083 / 0.421 | | |

| **Experiment 7 true political news - Gun Control issue only** | | | | | | | | | | | | |
| --- | --- | --- | --- | --- | --- | --- | --- | --- | --- | --- | --- | --- |
|  | **sharing** | | **sharing** | | **sharing** | | **sharing** | | | **sharing** | | |
| *Predictors* | *ß* | *p* | *ß* | *p* | *ß* | *p* | *ß* | *p* | *std. p* | *ß* | *p* | *std. p* |
| Congruent [TRUE] | 0.48 (0.40,0.55) | **<0.001** | 0.39 (0.30,0.49) | **<0.001** | 0.48 (0.40,0.55) | **<0.001** | 0.48 (0.40,0.55) | 0.910 | **<0.001** | 0.46 (0.36,0.56) | 0.913 | **<0.001** |
| AbsImpIssue [TRUE] | 0.21 (0.11,0.32) | **<0.001** | 0.11 (-0.02,0.23) | 0.111 |  |  |  |  |  | 0.15 (0.01,0.29) | **0.036** | **0.036** |
| Condition [message myside bias] | -0.15 (-0.26, -0.05) | **0.004** | -0.15 (-0.26, -0.05) | **0.004** | -0.16 (-0.26, -0.05) | **0.003** | -0.16 (-0.26, -0.05) | **0.003** | **0.003** | -0.15 (-0.26, -0.05) | **0.003** | **0.003** |
| edu | 0.03 (-0.03,0.08) | 0.327 | 0.03 (-0.03,0.08) | 0.327 | 0.02 (-0.03,0.07) | 0.468 | 0.02 (-0.03,0.07) | 0.468 | 0.468 | 0.02 (-0.03,0.08) | 0.365 | 0.365 |
| sex [female] | -0.03 (-0.13,0.08) | 0.611 | -0.03 (-0.13,0.08) | 0.611 | -0.05 (-0.15,0.06) | 0.378 | -0.05 (-0.15,0.06) | 0.378 | 0.378 | -0.04 (-0.15,0.07) | 0.471 | 0.471 |
| age | -0.02 (-0.07,0.04) | 0.518 | -0.02 (-0.07,0.04) | 0.518 | -0.02 (-0.07,0.04) | 0.551 | -0.02 (-0.07,0.04) | 0.551 | 0.551 | -0.02 (-0.07,0.03) | 0.447 | 0.447 |
| Congruent [TRUE] * AbsImpIssue [TRUE] |  |  | 0.21 (0.06,0.36) | **0.006** |  |  |  |  |  | 0.03 (-0.13,0.20) | 0.678 | 0.678 |
| Attit Issue extr |  |  |  |  | 0.09 (0.03,0.14) | **0.001** | -0.03 (-0.09,0.04) | 0.416 | 0.416 | -0.06 (-0.12,0.01) | 0.115 | 0.115 |
| Congruent [TRUE] * Attit Issue extr |  |  |  |  |  |  | 0.23 (0.15,0.30) | **<0.001** | **<0.001** | 0.22 (0.14,0.30) | **<0.001** | **<0.001** |
| **Random Effects** | | | | | | | | | | | | |
| σ^2^ | 0.15 | | 0.15 | | 0.15 | | 0.15 | | | 0.15 | | |
| τ_00_ | 0.07 _id_ | | 0.07 _id_ | | 0.07 _id_ | | 0.07 _id_ | | | 0.07 _id_ | | |
|  | 0.00 _News_ | | 0.00 _News_ | | 0.00 _News_ | | 0.00 _News_ | | | 0.00 _News_ | | |
| Marginal R^2^ / Conditional R^2^ | 0.105 / NA | | 0.109 / NA | | 0.101 / NA | | 0.121 / NA | | | 0.128 / NA | | |

| **Experiment 7 true political news - Racial equality issue only** | | | | | | | | | | | | |
| --- | --- | --- | --- | --- | --- | --- | --- | --- | --- | --- | --- | --- |
|  | **sharing** | | **sharing** | | **sharing** | | **sharing** | | | **sharing** | | |
| *Predictors* | *ß* | *p* | *ß* | *p* | *ß* | *p* | *ß* | *p* | *std. p* | *ß* | *p* | *std. p* |
| Congruent [TRUE] | 0.15 (0.05,0.25) | **0.003** | 0.08 (-0.03,0.18) | 0.157 | 0.15 (0.05,0.25) | **0.003** | 0.16 (0.07,0.26) | 0.149 | **0.001** | 0.11 (-0.00,0.22) | 0.210 | 0.052 |
| AbsImpIssue [TRUE] | 0.30 (0.19,0.40) | **<0.001** | 0.14 (0.01,0.28) | **0.039** |  |  |  |  |  | 0.16 (0.02,0.31) | **0.027** | **0.027** |
| Condition [message myside bias] | -0.05 (-0.15,0.06) | 0.357 | -0.05 (-0.15,0.06) | 0.357 | -0.04 (-0.15,0.06) | 0.424 | -0.04 (-0.15,0.06) | 0.424 | 0.424 | -0.05 (-0.15,0.05) | 0.353 | 0.353 |
| edu | 0.05 (0.00,0.11) | **0.040** | 0.05 (0.00,0.11) | **0.040** | 0.06 (0.00,0.11) | **0.041** | 0.06 (0.00,0.11) | **0.041** | **0.041** | 0.05 (0.00,0.11) | **0.045** | **0.045** |
| sex [female] | 0.03 (-0.08,0.14) | 0.574 | 0.03 (-0.08,0.14) | 0.574 | 0.03 (-0.08,0.14) | 0.579 | 0.03 (-0.08,0.14) | 0.579 | 0.579 | 0.03 (-0.08,0.13) | 0.638 | 0.638 |
| age | -0.13 (-0.18, -0.07) | **<0.001** | -0.13 (-0.18, -0.07) | **<0.001** | -0.13 (-0.19, -0.08) | **<0.001** | -0.13 (-0.19, -0.08) | **<0.001** | **<0.001** | -0.13 (-0.18, -0.07) | **<0.001** | **<0.001** |
| Congruent [TRUE] * AbsImpIssue [TRUE] |  |  | 0.31 (0.14,0.47) | **<0.001** |  |  |  |  |  | 0.23 (0.05,0.40) | **0.011** | **0.011** |
| Attit Issue extr |  |  |  |  | 0.07 (0.02,0.13) | **0.006** | 0.00 (-0.06,0.07) | 0.944 | 0.944 | -0.03 (-0.10,0.04) | 0.428 | 0.428 |
| Congruent [TRUE] * Attit Issue extr |  |  |  |  |  |  | 0.14 (0.07,0.22) | **<0.001** | **<0.001** | 0.11 (0.03,0.19) | **0.009** | **0.009** |
| **Random Effects** | | | | | | | | | | | | |
| σ^2^ | 0.14 | | 0.13 | | 0.14 | | 0.13 | | | 0.13 | | |
| τ_00_ | 0.06 _id_ | | 0.06 _id_ | | 0.06 _id_ | | 0.07 _id_ | | | 0.06 _id_ | | |
|  | 0.01 _News_ | | 0.01 _News_ | | 0.01 _News_ | | 0.01 _News_ | | | 0.01 _News_ | | |
| ICC | 0.33 | | 0.35 | | 0.34 | | 0.35 | | | 0.35 | | |
| Marginal R^2^ / Conditional R^2^ | 0.044 / 0.358 | | 0.052 / 0.380 | | 0.030 / 0.358 | | 0.036 / 0.372 | | | 0.055 / 0.385 | | |

### Congruence x Condition

| **Experiment 7 - true news** | | |
| --- | --- | --- |
|  | **sharing** | |
| *Predictors* | *ß* | *p* |
| Congruent [TRUE] | 0.40 (0.34,0.47) | **<0.001** |
| Condition [message myside bias] | -0.04 (-0.13,0.05) | 0.441 |
| edu | 0.04 (-0.00,0.08) | 0.055 |
| sex [female] | 0.05 (-0.04,0.13) | 0.281 |
| age | -0.07 (-0.11, -0.02) | **0.002** |
| Congruent [TRUE] * Condition [message myside bias] | -0.08 (-0.17, -0.00) | **0.049** |
| **Random Effects** | | |
| σ^2^ | 0.14 | |
| τ_00_ _id_ | 0.07 | |
| τ_00_ _News_ | 0.01 | |
| τ_11_ _id.CongruentTRUE_ | 0.02 | |
| τ_11_ _id.Conditionmessage myside bias_ | 0.21 | |
| ρ_01_ _id.CongruentTRUE_ | -0.07 | |
| ρ_01_ _id.Conditionmessage myside bias_ | -0.87 | |
| ICC | 0.37 | |
| Marginal R^2^ / Conditional R^2^ | 0.040 / 0.399 | |

### Effects of partisanship

| **Experiment 7 - true news** | | | | | | | | | | | | | |
| --- | --- | --- | --- | --- | --- | --- | --- | --- | --- | --- | --- | --- | --- |
|  | **sharing** | | **sharing** | | | **sharing** | | **sharing** | | **sharing** | | **sharing** | |
| *Predictors* | *ß* | *p* | *ß* | *p* | *std. p* | *ß* | *p* | *ß* | *p* | *ß* | *p* | *ß* | *p* |
| Condition [message myside bias] | -0.08 (-0.16,0.01) | 0.066 | -0.04 (-0.15,0.06) | 0.420 | 0.420 |  |  |  |  | -0.04 (-0.13,0.05) | 0.441 | 0.01 (-0.11,0.13) | 0.838 |
| edu | 0.04 (0.00,0.09) | **0.035** | 0.05 (0.00,0.09) | **0.032** | **0.032** | 0.05 (0.00,0.09) | **0.031** | 0.05 (0.01,0.09) | **0.028** | 0.04 (-0.00,0.08) | 0.055 | 0.05 (0.00,0.09) | **0.032** |
| sex [female] | 0.06 (-0.03,0.14) | 0.183 | 0.06 (-0.02,0.14) | 0.161 | 0.161 | 0.05 (-0.03,0.14) | 0.218 | 0.06 (-0.03,0.14) | 0.185 | 0.05 (-0.04,0.13) | 0.281 | 0.06 (-0.02,0.14) | 0.161 |
| age | -0.07 (-0.11, -0.02) | **0.002** | -0.06 (-0.11, -0.02) | **0.002** | **0.002** | -0.07 (-0.11, -0.02) | **0.002** | -0.07 (-0.11, -0.02) | **0.002** | -0.07 (-0.11, -0.02) | **0.002** | -0.06 (-0.11, -0.02) | **0.002** |
| Pol Orient bin [Republican] |  |  | 0.15 (0.04,0.27) | **0.011** | **0.011** |  |  | 0.24 (0.14,0.33) | **<0.001** |  |  | 0.28 (0.15,0.41) | **<0.001** |
| Condition [message myside bias] * Pol Orient bin [Republican] |  |  | -0.06 (-0.23,0.10) | 0.463 | 0.463 |  |  |  |  |  |  | -0.08 (-0.27,0.10) | 0.365 |
| Congruent [TRUE] |  |  |  |  |  | 0.36 (0.31,0.40) | **<0.001** | 0.48 (0.42,0.54) | **<0.001** | 0.40 (0.34,0.47) | **<0.001** | 0.54 (0.46,0.62) | **<0.001** |
| Congruent [TRUE] * Pol Orient bin [Republican] |  |  |  |  |  |  |  | -0.22 (-0.30, -0.14) | **<0.001** |  |  | -0.25 (-0.36, -0.14) | **<0.001** |
| Congruent [TRUE] * Condition [message myside bias] |  |  |  |  |  |  |  |  |  | -0.08 (-0.17, -0.00) | **0.049** | -0.11 (-0.21, -0.01) | **0.028** |
| (Congruent [TRUE] * Condition [message myside bias]) * Pol Orient bin [Republican] |  |  |  |  |  |  |  |  |  |  |  | 0.04 (-0.11,0.19) | 0.565 |
| **Random Effects** | | | | | | | | | | | | | |
| σ^2^ | 0.15 | | 0.15 | | | 0.14 | | 0.14 | | 0.14 | | 0.14 | |
| τ_00_ | 0.07 _id_ | | 0.06 _id_ | | | 0.07 _id_ | | 0.06 _id_ | | 0.07 _id_ | | 0.06 _id_ | |
|  | 0.00 _News_ | | 0.00 _News_ | | | 0.01 _News_ | | 0.01 _News_ | | 0.01 _News_ | | 0.01 _News_ | |
| τ_11_ | 0.28 _id.Conditionmessage myside bias_ | | 0.05 _id.Pol.Orient_binRepublican_ | | | 0.28 _id.Conditionmessage myside bias_ | | 0.02 _id.Pol.Orient_binRepublican_ | | 0.02 _id.CongruentTRUE_ | | 0.01 _id.Pol.Orient_binRepublican_ | |
|  |  | | 0.22 _id.Conditionmessage myside bias_ | | |  | | 0.21 _id.Conditionmessage myside bias_ | | 0.21 _id.Conditionmessage myside bias_ | | 0.12 _id.Conditionmessage myside bias_ | |
| ρ_01_ | -1.00 _id_ | | -0.30 _id.Pol.Orient_binRepublican_ | | | -1.00 _id_ | | -0.02 _id.Pol.Orient_binRepublican_ | | -0.07 _id.CongruentTRUE_ | | 0.19 _id.Pol.Orient_binRepublican_ | |
|  |  | | -0.94 _id.Conditionmessage myside bias_ | | |  | | -0.91 _id.Conditionmessage myside bias_ | | -0.87 _id.Conditionmessage myside bias_ | | -0.72 _id.Conditionmessage myside bias_ | |
| ICC |  | | 0.33 | | | 0.36 | | 0.35 | | 0.37 | | 0.35 | |
| Marginal R^2^ / Conditional R^2^ | 0.011 / NA | | 0.011 / 0.334 | | | 0.036 / 0.379 | | 0.046 / 0.383 | | 0.040 / 0.399 | | 0.049 / 0.379 | |

### Congruence x Condition on Democrats only (restricted analyses)

| **Experiment 7 true news, Democrats only** | | | | |
| --- | --- | --- | --- | --- |
|  | **sharing** | | **sharing** | |
| *Predictors* | *ß* | *p* | *ß* | *p* |
| Condition [message myside bias] | -0.02 (-0.13,0.09) | 0.695 | 0.01 (-0.10,0.13) | 0.823 |
| edu | 0.00 (-0.05,0.06) | 0.957 | -0.00 (-0.06,0.05) | 0.984 |
| sex [female] | -0.01 (-0.12,0.10) | 0.822 | -0.02 (-0.12,0.09) | 0.777 |
| age | -0.04 (-0.09,0.02) | 0.171 | -0.04 (-0.09,0.02) | 0.160 |
| Congruent [TRUE] |  |  | 0.41 (0.31,0.52) | **<0.001** |
| Congruent [TRUE] * Condition [message myside bias] |  |  | -0.11 (-0.23,0.01) | 0.071 |
| **Random Effects** | | | | |
| σ^2^ | 0.13 | | 0.13 | |
| τ_00_ | 0.06 _id_ | | 0.06 _id_ | |
|  | 0.01 _News_ | | 0.00 _News_ | |
| τ_11_ | 0.03 _id.CongruentTRUE_ | | 0.03 _id.CongruentTRUE_ | |
|  | 0.18 _id.Conditionmessage myside bias_ | | 0.19 _id.Conditionmessage myside bias_ | |
| ρ_01_ | -0.03 _id.CongruentTRUE_ | | 0.01 _id.CongruentTRUE_ | |
|  | -0.89 _id.Conditionmessage myside bias_ | | -0.91 _id.Conditionmessage myside bias_ | |
| ICC | 0.33 | | 0.36 | |
| Marginal R^2^ / Conditional R^2^ | 0.002 / 0.330 | | 0.034 / 0.383 | |

### Congruence x Condition on Republicans only (restricted analyses)

| **Experiment 7 true news, Republicans only** | | | | |
| --- | --- | --- | --- | --- |
|  | **sharing** | | **sharing** | |
| *Predictors* | *ß* | *p* | *ß* | *p* |
| Condition [message myside bias] | -0.09 (-0.22,0.03) | 0.143 | -0.06 (-0.19,0.07) | 0.365 |
| edu | 0.09 (0.03,0.16) | **0.003** | 0.09 (0.03,0.16) | **0.004** |
| sex [female] | 0.14 (0.00,0.27) | **0.042** | 0.14 (0.00,0.27) | **0.044** |
| age | -0.10 (-0.16, -0.04) | **0.002** | -0.10 (-0.17, -0.04) | **0.002** |
| Congruent [TRUE] |  |  | 0.28 (0.20,0.35) | **<0.001** |
| Congruent [TRUE] * Condition [message myside bias] |  |  | -0.07 (-0.18,0.03) | 0.176 |
| **Random Effects** | | | | |
| σ^2^ | 0.14 | | 0.14 | |
| τ_00_ | 0.08 _id_ | | 0.07 _id_ | |
|  | 0.01 _News_ | | 0.01 _News_ | |
| τ_11_ | 0.02 _id.CongruentTRUE_ | | 0.00 _id.CongruentTRUE_ | |
|  | 0.09 _id.Conditionmessage myside bias_ | | 0.00 _id.Conditionmessage myside bias_ | |
| ρ_01_ | -0.04 _id.CongruentTRUE_ | | 0.32 _id.CongruentTRUE_ | |
|  | -0.55 _id.Conditionmessage myside bias_ | | -0.18 _id.Conditionmessage myside bias_ | |
| ICC | 0.38 | | 0.39 | |
| Marginal R^2^ / Conditional R^2^ | 0.022 / 0.397 | | 0.036 / 0.417 | |

### Congruence x Issue moralization x Condition

| **Experiment 7 - true news** | | |
| --- | --- | --- |
|  | **sharing** | |
| *Predictors* | *ß* | *p* |
| Congruent [TRUE] | 0.31 (0.23,0.38) | **<0.001** |
| AbsImpIssue [TRUE] | 0.11 (0.02,0.20) | **0.018** |
| Condition [message myside bias] | -0.03 (-0.13,0.07) | 0.507 |
| edu | 0.04 (0.00,0.08) | **0.046** |
| sex [female] | 0.03 (-0.05,0.12) | 0.452 |
| age | -0.06 (-0.11, -0.02) | **0.002** |
| Congruent [TRUE] * AbsImpIssue [TRUE] | 0.32 (0.21,0.43) | **<0.001** |
| Congruent [TRUE] * Condition [message myside bias] | -0.08 (-0.18,0.02) | 0.119 |
| AbsImpIssue [TRUE] * Condition [message myside bias] | -0.01 (-0.13,0.11) | 0.892 |
| (Congruent [TRUE] * AbsImpIssue [TRUE]) * Condition [message myside bias] | -0.04 (-0.20,0.11) | 0.586 |
| **Random Effects** | | |
| σ^2^ | 0.13 | |
| τ_00_ _id_ | 0.07 | |
| τ_00_ _News_ | 0.01 | |
| τ_11_ _id.CongruentTRUE_ | 0.02 | |
| τ_11_ _id.Conditionmessage myside bias_ | 0.08 | |
| τ_11_ _id.AbsImpIssueTRUE_ | 0.02 | |
| ρ_01_ _id.CongruentTRUE_ | -0.16 | |
| ρ_01_ _id.Conditionmessage myside bias_ | -0.56 | |
| ρ_01_ _id.AbsImpIssueTRUE_ | -0.23 | |
| ICC | 0.40 | |
| Marginal R^2^ / Conditional R^2^ | 0.060 / 0.439 | |

### Congruence x Attitude extremity x Condition

| **Experiment 7 - true news** | | | |
| --- | --- | --- | --- |
|  | **sharing** | | |
| *Predictors* | *ß* | *p* | *std. p* |
| Congruent [TRUE] | 0.41 (0.35,0.47) | 0.619 | **<0.001** |
| Attit Issue extr | 0.00 (-0.04,0.04) | 0.964 | 0.965 |
| Condition [message myside bias] | -0.03 (-0.12,0.06) | 0.400 | 0.481 |
| edu | 0.03 (-0.01,0.08) | 0.104 | 0.104 |
| sex [female] | 0.03 (-0.06,0.11) | 0.549 | 0.549 |
| age | -0.07 (-0.11, -0.03) | **0.002** | **0.002** |
| Congruent [TRUE] * Attit Issue extr | 0.21 (0.16,0.26) | **<0.001** | **<0.001** |
| Congruent [TRUE] * Condition [message myside bias] | -0.09 (-0.17, -0.01) | 0.686 | **0.031** |
| Attit Issue extr * Condition [message myside bias] | 0.02 (-0.04,0.08) | 0.593 | 0.593 |
| (Congruent [TRUE] * Attit Issue extr) * Condition [message myside bias] | -0.02 (-0.10,0.05) | 0.518 | 0.517 |
| **Random Effects** | | | |
| σ^2^ | 0.13 | | |
| τ_00_ _id_ | 0.09 | | |
| τ_00_ _News_ | 0.01 | | |
| τ_11_ _id.CongruentTRUE_ | 0.02 | | |
| τ_11_ _id.Conditionmessage myside bias_ | 0.03 | | |
| τ_11_ _id.Attit_Issue_extr_ | 0.02 | | |
| ρ_01_ _id.CongruentTRUE_ | -0.18 | | |
| ρ_01_ _id.Conditionmessage myside bias_ | -0.51 | | |
| ρ_01_ _id.Attit_Issue_extr_ | -0.47 | | |
| ICC | 0.39 | | |
| Marginal R^2^ / Conditional R^2^ | 0.060 / 0.427 | | |

# L. Experiment 8: Intervention message on the myside bias, fake news

### Congruence x Issue moralization

| **Experiment 8 - fake news, full dataset** | | | | |
| --- | --- | --- | --- | --- |
|  | **sharing** | | **sharing** | |
| *Predictors* | *ß* | *p* | *ß* | *p* |
| Congruent [TRUE] | 0.30 (0.25,0.35) | **<0.001** | 0.23 (0.18,0.29) | **<0.001** |
| AbsImpIssue [TRUE] | 0.12 (0.07,0.18) | **<0.001** | 0.02 (-0.05,0.09) | 0.530 |
| Condition [message myside bias] | -0.02 (-0.11,0.07) | 0.669 | -0.02 (-0.11,0.07) | 0.664 |
| edu | 0.05 (0.01,0.10) | **0.025** | 0.05 (0.01,0.10) | **0.026** |
| sex [female] | 0.04 (-0.05,0.13) | 0.354 | 0.04 (-0.05,0.14) | 0.352 |
| age | -0.06 (-0.10, -0.01) | **0.012** | -0.06 (-0.10, -0.01) | **0.012** |
| Congruent [TRUE] * AbsImpIssue [TRUE] |  |  | 0.21 (0.12,0.29) | **<0.001** |
| **Random Effects** | | | | |
| σ^2^ | 0.13 | | 0.13 | |
| τ_00_ | 0.07 _id_ | | 0.07 _id_ | |
|  | 0.00 _News_ | | 0.00 _News_ | |
| τ_11_ | 0.02 _id.CongruentTRUE_ | | 0.02 _id.CongruentTRUE_ | |
|  | 0.02 _id.AbsImpIssueTRUE_ | | 0.02 _id.AbsImpIssueTRUE_ | |
|  | 0.12 _id.Conditionmessage myside bias_ | | 0.07 _id.Conditionmessage myside bias_ | |
| ρ_01_ | 0.02 _id.CongruentTRUE_ | | 0.00 _id.CongruentTRUE_ | |
|  | -0.31 _id.AbsImpIssueTRUE_ | | -0.31 _id.AbsImpIssueTRUE_ | |
|  | -0.60 _id.Conditionmessage myside bias_ | | -0.43 _id.Conditionmessage myside bias_ | |
| ICC | 0.37 | | 0.37 | |
| Marginal R^2^ / Conditional R^2^ | 0.032 / 0.393 | | 0.036 / 0.397 | |

| **Experiment 8 - No fake abortion items** | | | | | |
| --- | --- | --- | --- | --- | --- |
|  | **sharing** | | **sharing** | | |
| *Predictors* | *ß* | *p* | *ß* | *p* | *std. p* |
| Congruent [TRUE] | 0.37 (0.31,0.43) | **<0.001** | 0.29 (0.22,0.35) | **<0.001** | **<0.001** |
| AbsImpIssue [TRUE] | 0.16 (0.10,0.22) | **<0.001** | 0.03 (-0.05,0.10) | 0.522 | 0.522 |
| Condition [message myside bias] | 0.00 (-0.09,0.09) | 0.974 | 0.00 (-0.09,0.09) | 0.987 | 0.987 |
| edu | 0.06 (0.01,0.10) | **0.011** | 0.06 (0.01,0.10) | **0.011** | **0.011** |
| sex [female] | 0.03 (-0.06,0.12) | 0.534 | 0.03 (-0.06,0.12) | 0.532 | 0.532 |
| age | -0.05 (-0.09, -0.00) | **0.042** | -0.05 (-0.09, -0.00) | **0.040** | **0.040** |
| Congruent [TRUE] * AbsImpIssue [TRUE] |  |  | 0.28 (0.18,0.38) | **<0.001** | **<0.001** |
| **Random Effects** | | | | | |
| σ^2^ | 0.14 | | 0.14 | | |
| τ_00_ | 0.06 _id_ | | 0.06 _id_ | | |
|  | 0.00 _News_ | | 0.00 _News_ | | |
| τ_11_ | 0.02 _id.CongruentTRUE_ | | 0.02 _id.CongruentTRUE_ | | |
|  | 0.02 _id.AbsImpIssueTRUE_ | | 0.02 _id.AbsImpIssueTRUE_ | | |
|  | 0.23 _id.Conditionmessage myside bias_ | | 0.19 _id.Conditionmessage myside bias_ | | |
| ρ_01_ | 0.10 _id.CongruentTRUE_ | | 0.05 _id.CongruentTRUE_ | | |
|  | -0.33 _id.AbsImpIssueTRUE_ | | -0.34 _id.AbsImpIssueTRUE_ | | |
|  | -0.94 _id.Conditionmessage myside bias_ | | -0.83 _id.Conditionmessage myside bias_ | | |
| ICC | 0.36 | | 0.36 | | |
| Marginal R^2^ / Conditional R^2^ | 0.046 / 0.387 | | 0.054 / 0.396 | | |

### Congruence x Attitude extremity

| **Experiment 8 - fake news, full dataset** | | | | | | |
| --- | --- | --- | --- | --- | --- | --- |
|  | **sharing** | | | **sharing** | | |
| *Predictors* | *ß* | *p* | *std. p* | *ß* | *p* | *std. p* |
| Congruent [TRUE] | 0.30 (0.25,0.35) | **<0.001** | **<0.001** | 0.30 (0.25,0.35) | 0.209 | **<0.001** |
| Attit Issue extr | 0.05 (0.02,0.08) | **<0.001** | **<0.001** | -0.03 (-0.07,0.00) | **0.039** | 0.054 |
| Condition [message myside bias] | -0.02 (-0.11,0.07) | 0.604 | 0.604 | -0.02 (-0.11,0.07) | 0.690 | 0.615 |
| edu | 0.05 (0.01,0.10) | **0.022** | **0.022** | 0.05 (0.01,0.10) | **0.022** | **0.023** |
| sex [female] | 0.05 (-0.04,0.14) | 0.279 | 0.279 | 0.05 (-0.04,0.14) | 0.339 | 0.305 |
| age | -0.06 (-0.11, -0.02) | **0.008** | **0.008** | -0.06 (-0.11, -0.02) | **0.009** | **0.006** |
| Congruent [TRUE] * Attit Issue extr |  |  |  | 0.17 (0.12,0.21) | **<0.001** | **<0.001** |
| **Random Effects** | | | | | | |
| σ^2^ | 0.14 | | | 0.14 | | |
| τ_00_ | 0.07 _id_ | | | 0.05 _id_ | | |
|  | 0.00 _News_ | | | 0.00 _News_ | | |
| τ_11_ | 0.02 _id.CongruentTRUE_ | | | 0.00 _id.CongruentTRUE_ | | |
|  | 0.01 _id.Attit_Issue_extr_ | | | 0.00 _id.Attit_Issue_extr_ | | |
|  | 0.08 _id.Conditionmessage myside bias_ | | | 0.16 _id.Conditionmessage myside bias_ | | |
| ρ_01_ | -0.19 _id.CongruentTRUE_ | | | 1.00 _id.CongruentTRUE_ | | |
|  | -0.37 _id.Attit_Issue_extr_ | | | 0.25 _id.Attit_Issue_extr_ | | |
|  | -0.29 _id.Conditionmessage myside bias_ | | | -0.70 _id.Conditionmessage myside bias_ | | |
| ICC | 0.36 | | |  | | |
| Marginal R^2^ / Conditional R^2^ | 0.031 / 0.380 | | | 0.058 / NA | | |
| **Experiment 8 - fake news, No fake abortion items** | | | | | | |
|  | **sharing** | | | **sharing** | | |
| *Predictors* | *ß* | *p* | *std. p* | *ß* | *p* | *std. p* |
| Congruent [TRUE] | 0.37 (0.31,0.43) | **<0.001** | **<0.001** | 0.38 (0.32,0.43) | **0.031** | **<0.001** |
| Attit Issue extr | 0.06 (0.03,0.09) | **<0.001** | **<0.001** | -0.05 (-0.09, -0.01) | **0.008** | **0.008** |
| Condition [message myside bias] | -0.00 (-0.09,0.09) | 0.961 | 0.961 | -0.00 (-0.09,0.09) | 0.945 | 0.945 |
| edu | 0.06 (0.02,0.11) | **0.009** | **0.009** | 0.06 (0.01,0.11) | **0.010** | **0.010** |
| sex [female] | 0.03 (-0.06,0.13) | 0.487 | 0.487 | 0.03 (-0.06,0.12) | 0.528 | 0.528 |
| age | -0.05 (-0.10, -0.01) | **0.022** | **0.022** | -0.05 (-0.10, -0.01) | **0.020** | **0.020** |
| Congruent [TRUE] * Attit Issue extr |  |  |  | 0.24 (0.19,0.28) | **<0.001** | **<0.001** |
| **Random Effects** | | | | | | |
| σ^2^ | 0.14 | | | 0.14 | | |
| τ_00_ | 0.08 _id_ | | | 0.08 _id_ | | |
|  | 0.00 _News_ | | | 0.00 _News_ | | |
| τ_11_ | 0.02 _id.CongruentTRUE_ | | | 0.01 _id.CongruentTRUE_ | | |
|  | 0.01 _id.Attit_Issue_extr_ | | | 0.02 _id.Attit_Issue_extr_ | | |
|  | 0.06 _id.Conditionmessage myside bias_ | | | 0.06 _id.Conditionmessage myside bias_ | | |
| ρ_01_ | -0.10 _id.CongruentTRUE_ | | | -0.05 _id.CongruentTRUE_ | | |
|  | -0.56 _id.Attit_Issue_extr_ | | | -0.55 _id.Attit_Issue_extr_ | | |
|  | -0.36 _id.Conditionmessage myside bias_ | | | -0.38 _id.Conditionmessage myside bias_ | | |
| ICC | 0.35 | | | 0.35 | | |
| Marginal R^2^ / Conditional R^2^ | 0.044 / 0.376 | | | 0.060 / 0.389 | | |

### Congruence x Issue moralization controlling for Congruence x Attitude extremity

| **Experiment 8 – fake news, full dataset** | | | |
| --- | --- | --- | --- |
|  | **sharing** | | |
| *Predictors* | *ß* | *p* | *std. p* |
| Condition [message myside bias] | -0.02 (-0.11,0.07) | 0.605 | 0.605 |
| Congruent [TRUE] | 0.27 (0.22,0.33) | 0.296 | **<0.001** |
| AbsImpIssue [TRUE] | 0.06 (-0.02,0.13) | 0.138 | 0.138 |
| Attit Issue extr | -0.05 (-0.08, -0.01) | **0.014** | **0.014** |
| edu | 0.05 (0.01,0.09) | **0.027** | **0.027** |
| sex [female] | 0.04 (-0.05,0.13) | 0.387 | 0.387 |
| age | -0.06 (-0.11, -0.02) | **0.007** | **0.007** |
| Congruent [TRUE] * AbsImpIssue [TRUE] | 0.09 (-0.00,0.18) | 0.061 | 0.061 |
| Congruent [TRUE] * Attit Issue extr | 0.15 (0.11,0.19) | **<0.001** | **<0.001** |
| **Random Effects** | | | |
| σ^2^ | 0.13 | | |
| τ_00_ _id_ | 0.07 | | |
| τ_00_ _News_ | 0.00 | | |
| τ_11_ _id.CongruentTRUE_ | 0.02 | | |
| τ_11_ _id.Attit_Issue_extr_ | 0.01 | | |
| τ_11_ _id.AbsImpIssueTRUE_ | 0.02 | | |
| τ_11_ _id.Conditionmessage myside bias_ | 0.10 | | |
| ρ_01_ _id.CongruentTRUE_ | -0.13 | | |
| ρ_01_ _id.Attit_Issue_extr_ | -0.19 | | |
| ρ_01_ _id.AbsImpIssueTRUE_ | -0.28 | | |
| ρ_01_ _id.Conditionmessage myside bias_ | -0.40 | | |
| ICC | 0.38 | | |
| Marginal R^2^ / Conditional R^2^ | 0.041 / 0.402 | | |

| **Experiment 8 - fake news, No fake abortion items** | | | |
| --- | --- | --- | --- |
|  | **sharing** | | |
| *Predictors* | *ß* | *p* | *std. p* |
| Condition [message myside bias] | -0.00 (-0.10,0.09) | 0.924 | 0.924 |
| Congruent [TRUE] | 0.34 (0.28,0.41) | **0.038** | **<0.001** |
| AbsImpIssue [TRUE] | 0.08 (-0.01,0.16) | 0.083 | 0.083 |
| Attit Issue extr | -0.07 (-0.11, -0.03) | **0.001** | **0.001** |
| edu | 0.06 (0.02,0.11) | **0.009** | **0.009** |
| sex [female] | 0.03 (-0.06,0.12) | 0.531 | 0.531 |
| age | -0.05 (-0.10, -0.01) | **0.023** | **0.023** |
| Congruent [TRUE] * AbsImpIssue [TRUE] | 0.11 (0.01,0.22) | **0.037** | **0.038** |
| Congruent [TRUE] * Attit Issue extr | 0.22 (0.17,0.27) | **<0.001** | **<0.001** |
| **Random Effects** | | | |
| σ^2^ | 0.13 | | |
| τ_00_ _id_ | 0.08 | | |
| τ_00_ _News_ | 0.00 | | |
| τ_11_ _id.CongruentTRUE_ | 0.02 | | |
| τ_11_ _id.Attit_Issue_extr_ | 0.02 | | |
| τ_11_ _id.AbsImpIssueTRUE_ | 0.02 | | |
| τ_11_ _id.Conditionmessage myside bias_ | 0.07 | | |
| ρ_01_ _id.CongruentTRUE_ | -0.10 | | |
| ρ_01_ _id.Attit_Issue_extr_ | -0.47 | | |
| ρ_01_ _id.AbsImpIssueTRUE_ | -0.06 | | |
| ρ_01_ _id.Conditionmessage myside bias_ | -0.42 | | |
| ICC | 0.37 | | |
| Marginal R^2^ / Conditional R^2^ | 0.065 / 0.410 | | |

### By issue analyses

| **Experiment 8 fake political news - Abortion issue only** | | | | | | | | | | | | |
| --- | --- | --- | --- | --- | --- | --- | --- | --- | --- | --- | --- | --- |
|  | **sharing** | | **sharing** | | **sharing** | | **sharing** | | | **sharing** | | |
| *Predictors* | *ß* | *p* | *ß* | *p* | *ß* | *p* | *ß* | *p* | *std. p* | *ß* | *p* | *std. p* |
| Congruent [TRUE] | 0.09 (0.01,0.18) | **0.034** | 0.08 (-0.03,0.18) | 0.141 | 0.09 (0.01,0.18) | **0.034** | 0.09 (0.01,0.18) | 0.362 | **0.034** | 0.08 (-0.03,0.18) | 0.363 | 0.164 |
| AbsImpIssue [TRUE] | -0.02 (-0.15,0.10) | 0.720 | -0.04 (-0.19,0.11) | 0.603 |  |  |  |  |  | 0.02 (-0.14,0.18) | 0.794 | 0.794 |
| Condition [message myside bias] | -0.09 (-0.21,0.03) | 0.145 | -0.09 (-0.21,0.03) | 0.145 | -0.09 (-0.21,0.03) | 0.123 | -0.09 (-0.21,0.03) | 0.123 | 0.123 | -0.09 (-0.21,0.03) | 0.123 | 0.123 |
| edu | 0.02 (-0.04,0.08) | 0.466 | 0.02 (-0.04,0.08) | 0.466 | 0.02 (-0.04,0.08) | 0.539 | 0.02 (-0.04,0.08) | 0.539 | 0.539 | 0.02 (-0.04,0.08) | 0.530 | 0.530 |
| sex [female] | 0.09 (-0.03,0.22) | 0.153 | 0.09 (-0.03,0.22) | 0.153 | 0.11 (-0.01,0.24) | 0.071 | 0.11 (-0.01,0.24) | 0.071 | 0.071 | 0.11 (-0.02,0.23) | 0.089 | 0.089 |
| age | -0.10 (-0.16, -0.03) | **0.002** | -0.10 (-0.16, -0.03) | **0.002** | -0.10 (-0.16, -0.04) | **0.002** | -0.10 (-0.16, -0.04) | **0.002** | **0.002** | -0.09 (-0.16, -0.03) | **0.002** | **0.002** |
| Congruent [TRUE] * AbsImpIssue [TRUE] |  |  | 0.03 (-0.14,0.20) | 0.689 |  |  |  |  |  | 0.04 (-0.14,0.22) | 0.656 | 0.656 |
| Attit Issue extr |  |  |  |  | -0.09 (-0.15, -0.03) | **0.003** | -0.09 (-0.16, -0.02) | **0.013** | **0.013** | -0.09 (-0.17, -0.02) | **0.015** | **0.015** |
| Congruent [TRUE] * Attit Issue extr |  |  |  |  |  |  | -0.00 (-0.08,0.08) | 0.960 | 0.960 | -0.01 (-0.09,0.08) | 0.842 | 0.842 |
| **Random Effects** | | | | | | | | | | | | |
| σ^2^ | 0.13 | | 0.13 | | 0.13 | | 0.13 | | | 0.13 | | |
| τ_00_ | 0.07 _id_ | | 0.07 _id_ | | 0.07 _id_ | | 0.07 _id_ | | | 0.07 _id_ | | |
|  | 0.00 _News_ | | 0.00 _News_ | | 0.00 _News_ | | 0.00 _News_ | | | 0.00 _News_ | | |
| ICC | 0.37 | | 0.37 | | 0.37 | | 0.37 | | | 0.37 | | |
| Marginal R^2^ / Conditional R^2^ | 0.014 / 0.383 | | 0.014 / 0.383 | | 0.022 / 0.383 | | 0.022 / 0.383 | | | 0.022 / 0.383 | | |

| **Experiment 8 fake political news - Gender equality issue only** | | | | | | | | | | | | |
| --- | --- | --- | --- | --- | --- | --- | --- | --- | --- | --- | --- | --- |
|  | **sharing** | | **sharing** | | **sharing** | | **sharing** | | | **sharing** | | |
| *Predictors* | *ß* | *p* | *ß* | *p* | *ß* | *p* | *ß* | *p* | *std. p* | *ß* | *p* | *std. p* |
| Congruent [TRUE] | 0.42 (0.33,0.52) | **<0.001** | 0.35 (0.25,0.45) | **<0.001** | 0.42 (0.33,0.52) | **<0.001** | 0.44 (0.35,0.53) | 0.559 | **<0.001** | 0.40 (0.30,0.50) | 0.604 | **<0.001** |
| AbsImpIssue [TRUE] | 0.23 (0.11,0.35) | **<0.001** | 0.07 (-0.08,0.23) | 0.347 |  |  |  |  |  | 0.12 (-0.04,0.28) | 0.131 | 0.131 |
| Condition [message myside bias] | 0.06 (-0.05,0.17) | 0.273 | 0.06 (-0.05,0.17) | 0.273 | 0.05 (-0.06,0.17) | 0.339 | 0.05 (-0.06,0.17) | 0.337 | 0.337 | 0.06 (-0.05,0.17) | 0.289 | 0.289 |
| edu | 0.08 (0.02,0.13) | **0.005** | 0.08 (0.02,0.14) | **0.005** | 0.09 (0.03,0.14) | **0.002** | 0.09 (0.03,0.14) | **0.002** | **0.002** | 0.08 (0.03,0.14) | **0.004** | **0.004** |
| sex [female] | 0.14 (0.03,0.26) | **0.016** | 0.14 (0.03,0.26) | **0.015** | 0.17 (0.05,0.28) | **0.005** | 0.17 (0.05,0.28) | **0.005** | **0.005** | 0.14 (0.03,0.26) | **0.014** | **0.014** |
| age | -0.02 (-0.07,0.04) | 0.578 | -0.02 (-0.07,0.04) | 0.573 | -0.03 (-0.08,0.03) | 0.349 | -0.03 (-0.08,0.03) | 0.346 | 0.346 | -0.02 (-0.07,0.04) | 0.552 | 0.552 |
| Congruent [TRUE] * AbsImpIssue [TRUE] |  |  | 0.31 (0.13,0.49) | **0.001** |  |  |  |  |  | 0.16 (-0.03,0.35) | 0.094 | 0.094 |
| Attit Issue extr |  |  |  |  | 0.07 (0.01,0.12) | **0.021** | -0.05 (-0.12,0.01) | 0.122 | 0.122 | -0.07 (-0.15, -0.00) | **0.048** | **0.048** |
| Congruent [TRUE] * Attit Issue extr |  |  |  |  |  |  | 0.24 (0.16,0.32) | **<0.001** | **<0.001** | 0.22 (0.13,0.31) | **<0.001** | **<0.001** |
| **Random Effects** | | | | | | | | | | | | |
| σ^2^ | 0.16 | | 0.16 | | 0.16 | | 0.15 | | | 0.15 | | |
| τ_00_ | 0.06 _id_ | | 0.06 _id_ | | 0.06 _id_ | | 0.06 _id_ | | | 0.06 _id_ | | |
|  | 0.00 _News_ | | 0.00 _News_ | | 0.00 _News_ | | 0.00 _News_ | | | 0.00 _News_ | | |
| ICC | 0.27 | |  | | 0.28 | | 0.30 | | |  | | |
| Marginal R^2^ / Conditional R^2^ | 0.070 / 0.323 | | 0.107 / NA | | 0.063 / 0.323 | | 0.081 / 0.352 | | | 0.124 / NA | | |

| **Experiment 8 fake political news - Gun Control issue only** | | | | | | | | | | | | |
| --- | --- | --- | --- | --- | --- | --- | --- | --- | --- | --- | --- | --- |
|  | **sharing** | | **sharing** | | **sharing** | | **sharing** | | | **sharing** | | |
| *Predictors* | *ß* | *p* | *ß* | *p* | *ß* | *p* | *ß* | *p* | *std. p* | *ß* | *p* | *std. p* |
| Congruent [TRUE] | 0.46 (0.37,0.55) | **<0.001** | 0.38 (0.27,0.49) | **<0.001** | 0.46 (0.37,0.55) | **<0.001** | 0.47 (0.38,0.55) | 0.095 | **<0.001** | 0.47 (0.36,0.59) | 0.096 | **<0.001** |
| AbsImpIssue [TRUE] | 0.17 (0.05,0.28) | **0.004** | 0.07 (-0.08,0.21) | 0.361 |  |  |  |  |  | 0.16 (0.00,0.31) | **0.043** | **0.043** |
| Condition [message myside bias] | -0.07 (-0.18,0.04) | 0.234 | -0.07 (-0.18,0.04) | 0.233 | -0.07 (-0.18,0.05) | 0.250 | -0.07 (-0.18,0.05) | 0.249 | 0.249 | -0.07 (-0.18,0.05) | 0.242 | 0.242 |
| edu | 0.01 (-0.05,0.06) | 0.777 | 0.01 (-0.05,0.06) | 0.776 | 0.01 (-0.05,0.07) | 0.747 | 0.01 (-0.05,0.07) | 0.745 | 0.745 | 0.01 (-0.05,0.06) | 0.771 | 0.771 |
| sex [female] | -0.02 (-0.13,0.09) | 0.732 | -0.02 (-0.13,0.09) | 0.731 | -0.03 (-0.14,0.09) | 0.648 | -0.03 (-0.14,0.09) | 0.645 | 0.645 | -0.02 (-0.14,0.09) | 0.709 | 0.709 |
| age | -0.04 (-0.10,0.02) | 0.173 | -0.04 (-0.10,0.02) | 0.173 | -0.04 (-0.09,0.02) | 0.213 | -0.04 (-0.09,0.02) | 0.213 | 0.213 | -0.04 (-0.10,0.02) | 0.172 | 0.172 |
| Congruent [TRUE] * AbsImpIssue [TRUE] |  |  | 0.20 (0.03,0.38) | **0.021** |  |  |  |  |  | -0.01 (-0.19,0.17) | 0.917 | 0.917 |
| Attit Issue extr |  |  |  |  | 0.05 (-0.01,0.11) | 0.083 | -0.09 (-0.16, -0.02) | **0.008** | **0.008** | -0.12 (-0.20, -0.05) | **0.001** | **0.001** |
| Congruent [TRUE] * Attit Issue extr |  |  |  |  |  |  | 0.29 (0.21,0.37) | **<0.001** | **<0.001** | 0.29 (0.20,0.38) | **<0.001** | **<0.001** |
| **Random Effects** | | | | | | | | | | | | |
| σ^2^ | 0.16 | | 0.16 | | 0.16 | | 0.15 | | | 0.15 | | |
| τ_00_ | 0.06 _id_ | | 0.06 _id_ | | 0.06 _id_ | | 0.06 _id_ | | | 0.06 _id_ | | |
|  | 0.00 _News_ | | 0.00 _News_ | | 0.00 _News_ | | 0.00 _News_ | | | 0.00 _News_ | | |
| ICC | 0.27 | | 0.27 | | 0.27 | | 0.30 | | | 0.30 | | |
| Marginal R^2^ / Conditional R^2^ | 0.062 / 0.312 | | 0.064 / 0.317 | | 0.057 / 0.312 | | 0.080 / 0.355 | | | 0.085 / 0.355 | | |

| **Experiment 8 fake political news - Racial equality issue only** | | | | | | | | | | | | |
| --- | --- | --- | --- | --- | --- | --- | --- | --- | --- | --- | --- | --- |
|  | **sharing** | | **sharing** | | **sharing** | | **sharing** | | | **sharing** | | |
| *Predictors* | *ß* | *p* | *ß* | *p* | *ß* | *p* | *ß* | *p* | *std. p* | *ß* | *p* | *std. p* |
| Congruent [TRUE] | 0.18 (0.08,0.27) | **<0.001** | 0.11 (0.01,0.21) | **0.028** | 0.18 (0.08,0.27) | **<0.001** | 0.19 (0.09,0.28) | 0.066 | **<0.001** | 0.14 (0.04,0.25) | 0.136 | **0.006** |
| AbsImpIssue [TRUE] | 0.23 (0.11,0.36) | **<0.001** | 0.07 (-0.08,0.22) | 0.335 |  |  |  |  |  | 0.11 (-0.05,0.28) | 0.171 | 0.171 |
| Condition [message myside bias] | 0.00 (-0.12,0.12) | 0.990 | 0.00 (-0.12,0.12) | 0.990 | 0.00 (-0.12,0.12) | 0.966 | 0.00 (-0.12,0.12) | 0.966 | 0.966 | 0.00 (-0.12,0.12) | 0.989 | 0.989 |
| edu | 0.09 (0.03,0.15) | **0.003** | 0.09 (0.03,0.15) | **0.003** | 0.09 (0.03,0.15) | **0.004** | 0.09 (0.03,0.15) | **0.004** | **0.004** | 0.09 (0.03,0.15) | **0.003** | **0.003** |
| sex [female] | -0.03 (-0.15,0.09) | 0.597 | -0.03 (-0.15,0.09) | 0.597 | -0.03 (-0.15,0.09) | 0.616 | -0.03 (-0.15,0.09) | 0.616 | 0.616 | -0.03 (-0.15,0.09) | 0.601 | 0.601 |
| age | -0.08 (-0.14, -0.02) | **0.009** | -0.08 (-0.14, -0.02) | **0.009** | -0.09 (-0.15, -0.03) | **0.003** | -0.09 (-0.15, -0.03) | **0.003** | **0.003** | -0.08 (-0.14, -0.02) | **0.008** | **0.008** |
| Congruent [TRUE] * AbsImpIssue [TRUE] |  |  | 0.32 (0.15,0.48) | **<0.001** |  |  |  |  |  | 0.20 (0.02,0.38) | **0.029** | **0.029** |
| Attit Issue extr |  |  |  |  | 0.06 (0.00,0.12) | **0.036** | -0.02 (-0.09,0.05) | 0.574 | 0.574 | -0.04 (-0.12,0.03) | 0.271 | 0.271 |
| Congruent [TRUE] * Attit Issue extr |  |  |  |  |  |  | 0.17 (0.09,0.25) | **<0.001** | **<0.001** | 0.13 (0.05,0.22) | **0.002** | **0.002** |
| **Random Effects** | | | | | | | | | | | | |
| σ^2^ | 0.11 | | 0.11 | | 0.11 | | 0.11 | | | 0.11 | | |
| τ_00_ | 0.08 _id_ | | 0.08 _id_ | | 0.08 _id_ | | 0.08 _id_ | | | 0.08 _id_ | | |
|  | 0.00 _News_ | | 0.00 _News_ | | 0.00 _News_ | | 0.00 _News_ | | | 0.00 _News_ | | |
| ICC | 0.41 | | 0.41 | | 0.41 | | 0.42 | | | 0.42 | | |
| Marginal R^2^ / Conditional R^2^ | 0.037 / 0.431 | | 0.048 / 0.440 | | 0.028 / 0.431 | | 0.037 / 0.444 | | | 0.050 / 0.447 | | |

### Congruence x Condition

| **Experiment 8** | | |
| --- | --- | --- |
|  | **sharing** | |
| *Predictors* | *ß* | *p* |
| Congruent [TRUE] | 0.34 (0.28,0.41) | **<0.001** |
| Condition [message myside bias] | 0.02 (-0.08,0.12) | 0.679 |
| edu | 0.05 (0.01,0.10) | **0.024** |
| sex [female] | 0.05 (-0.04,0.14) | 0.269 |
| age | -0.06 (-0.10, -0.01) | **0.011** |
| Congruent [TRUE] * Condition [message myside bias] | -0.09 (-0.18,0.00) | 0.055 |
| **Random Effects** | | |
| σ^2^ | 0.14 | |
| τ_00_ _id_ | 0.06 | |
| τ_00_ _News_ | 0.00 | |
| τ_11_ _id.CongruentTRUE_ | 0.02 | |
| τ_11_ _id.Conditionmessage myside bias_ | 0.23 | |
| ρ_01_ _id.CongruentTRUE_ | 0.14 | |
| ρ_01_ _id.Conditionmessage myside bias_ | -0.93 | |
| ICC | 0.35 | |
| Marginal R^2^ / Conditional R^2^ | 0.029 / 0.372 | |

### Effects of partisanship

| **Experiment 8 - fake news** | | | | | | | | | | | | | | | |
| --- | --- | --- | --- | --- | --- | --- | --- | --- | --- | --- | --- | --- | --- | --- | --- |
|  | **sharing** | | **sharing** | | **sharing** | | **sharing** | | | **sharing** | | | **sharing** | | |
| *Predictors* | *ß* | *p* | *ß* | *p* | *ß* | *p* | *ß* | *p* | *std. p* | *ß* | *p* | *ß* | | *p* | *std. p* |
| Condition [message myside bias] | -0.02 (-0.11,0.07) | 0.608 | -0.00 (-0.12,0.11) | 0.950 |  |  |  |  |  | 0.02 (-0.08,0.12) | 0.679 | 0.04 (-0.08,0.17) | | 0.517 | 0.517 |
| edu | 0.05 (0.00,0.09) | **0.032** | 0.04 (-0.00,0.09) | 0.054 | 0.05 (0.00,0.09) | **0.031** | 0.04 (-0.00,0.09) | 0.051 | 0.051 | 0.05 (0.01,0.10) | **0.024** | 0.04 (-0.00,0.09) | | 0.054 | 0.054 |
| sex [female] | 0.06 (-0.04,0.15) | 0.242 | 0.07 (-0.02,0.16) | 0.137 | 0.06 (-0.04,0.15) | 0.241 | 0.07 (-0.02,0.16) | 0.137 | 0.137 | 0.05 (-0.04,0.14) | 0.269 | 0.07 (-0.02,0.16) | | 0.138 | 0.138 |
| age | -0.06 (-0.11, -0.02) | **0.009** | -0.06 (-0.11, -0.02) | **0.006** | -0.06 (-0.11, -0.02) | **0.009** | -0.06 (-0.11, -0.02) | **0.006** | **0.006** | -0.06 (-0.10, -0.01) | **0.011** | -0.06 (-0.11, -0.02) | | **0.006** | **0.006** |
| Pol Orient bin [Republican] |  |  | 0.20 (0.07,0.33) | **0.003** |  |  | 0.28 (0.18,0.39) | **<0.001** | **<0.001** |  |  | 0.29 (0.15,0.43) | | **<0.001** | **<0.001** |
| Condition [message myside bias] * Pol Orient bin [Republican] |  |  | -0.03 (-0.21,0.16) | 0.785 |  |  |  |  |  |  |  | -0.01 (-0.22,0.19) | | 0.901 | 0.901 |
| Congruent [TRUE] |  |  |  |  | 0.30 (0.25,0.35) | **<0.001** | 0.39 (0.33,0.46) | **<0.001** | **<0.001** | 0.34 (0.28,0.41) | **<0.001** | 0.44 (0.36,0.52) | | **<0.001** | **<0.001** |
| Congruent [TRUE] * Pol Orient bin [Republican] |  |  |  |  |  |  | -0.20 (-0.28, -0.11) | **<0.001** | **<0.001** |  |  | -0.19 (-0.31, -0.07) | | **0.002** | **0.002** |
| Congruent [TRUE] * Condition [message myside bias] |  |  |  |  |  |  |  |  |  | -0.09 (-0.18,0.00) | 0.055 | -0.09 (-0.20,0.02) | | 0.099 | 0.099 |
| (Congruent [TRUE] * Condition [message myside bias]) * Pol Orient bin [Republican] |  |  |  |  |  |  |  |  |  |  |  | -0.03 (-0.20,0.14) | | 0.761 | 0.761 |
| **Random Effects** | | | | | | | | | | | | | | | |
| σ^2^ | 0.15 | | 0.15 | | 0.14 | | 0.14 | | | 0.14 | | | 0.14 | | |
| τ_00_ | 0.07 _id_ | | 0.06 _id_ | | 0.07 _id_ | | 0.06 _id_ | | | 0.06 _id_ | | | 0.06 _id_ | | |
|  | 0.00 _News_ | | 0.00 _News_ | | 0.00 _News_ | | 0.00 _News_ | | | 0.00 _News_ | | | 0.00 _News_ | | |
| τ_11_ | 0.22 _id.Conditionmessage myside bias_ | | 0.00 _id.Pol.Orient_binRepublican_ | | 0.00 _id.Conditionmessage myside bias_ | | 0.01 _id.Pol.Orient_binRepublican_ | | | 0.02 _id.CongruentTRUE_ | | | 0.03 _id.Pol.Orient_binRepublican_ | | |
|  |  | | 0.24 _id.Conditionmessage myside bias_ | |  | | 0.19 _id.Conditionmessage myside bias_ | | | 0.23 _id.Conditionmessage myside bias_ | | | 0.22 _id.Conditionmessage myside bias_ | | |
| ρ_01_ | -0.92 _id_ | | 0.13 _id.Pol.Orient_binRepublican_ | | -0.36 _id_ | | -0.08 _id.Pol.Orient_binRepublican_ | | | 0.14 _id.CongruentTRUE_ | | | -0.26 _id.Pol.Orient_binRepublican_ | | |
|  |  | | -0.99 _id.Conditionmessage myside bias_ | |  | | -0.89 _id.Conditionmessage myside bias_ | | | -0.93 _id.Conditionmessage myside bias_ | | | -0.94 _id.Conditionmessage myside bias_ | | |
| ICC | 0.33 | | 0.32 | | 0.34 | | 0.33 | | | 0.35 | | | 0.33 | | |
| Marginal R^2^ / Conditional R^2^ | 0.006 / 0.332 | | 0.014 / 0.332 | | 0.028 / 0.358 | | 0.040 / 0.361 | | | 0.029 / 0.372 | | | 0.041 / 0.356 | | |

### Congruence x Condition on Democrats only (restricted analyses)

| **Experiment 8 fake news, Democrats only** | | | | |
| --- | --- | --- | --- | --- |
|  | **sharing** | | **sharing** | |
| *Predictors* | *ß* | *p* | *ß* | *p* |
| Condition [message myside bias] | 0.05 (-0.06,0.17) | 0.364 | 0.03 (-0.09,0.15) | 0.616 |
| edu | 0.01 (-0.04,0.07) | 0.611 | 0.01 (-0.04,0.07) | 0.642 |
| sex [female] | 0.07 (-0.04,0.19) | 0.210 | 0.07 (-0.04,0.19) | 0.223 |
| age | -0.02 (-0.08,0.03) | 0.427 | -0.02 (-0.08,0.04) | 0.446 |
| Congruent [TRUE] |  |  | 0.34 (0.25,0.44) | **<0.001** |
| Congruent [TRUE] * Condition [message myside bias] |  |  | -0.08 (-0.20,0.04) | 0.198 |
| **Random Effects** | | | | |
| σ^2^ | 0.13 | | 0.12 | |
| τ_00_ | 0.05 _id_ | | 0.05 _id_ | |
|  | 0.01 _News_ | | 0.01 _News_ | |
| τ_11_ | 0.03 _id.CongruentTRUE_ | | 0.02 _id.CongruentTRUE_ | |
|  | 0.20 _id.Conditionmessage myside bias_ | | 0.11 _id.Conditionmessage myside bias_ | |
| ρ_01_ | 0.34 _id.CongruentTRUE_ | | 0.42 _id.CongruentTRUE_ | |
|  | -0.91 _id.Conditionmessage myside bias_ | | -0.63 _id.Conditionmessage myside bias_ | |
| ICC | 0.36 | | 0.38 | |
| Marginal R^2^ / Conditional R^2^ | 0.002 / 0.357 | | 0.026 / 0.393 | |

### Congruence x Condition on Republicans only (restricted analyses)

| **Experiment 8 fake news, Republicans only** | | | | |
| --- | --- | --- | --- | --- |
|  | **sharing** | | **sharing** | |
| *Predictors* | *ß* | *p* | *ß* | *p* |
| Condition [message myside bias] | -0.03 (-0.17,0.11) | 0.665 | 0.01 (-0.14,0.17) | 0.870 |
| edu | 0.09 (0.02,0.16) | **0.013** | 0.09 (0.02,0.16) | **0.013** |
| sex [female] | 0.05 (-0.10,0.19) | 0.514 | 0.05 (-0.10,0.19) | 0.523 |
| age | -0.13 (-0.20, -0.06) | **0.001** | -0.13 (-0.20, -0.06) | **0.001** |
| Congruent [TRUE] |  |  | 0.24 (0.15,0.33) | **<0.001** |
| Congruent [TRUE] * Condition [message myside bias] |  |  | -0.12 (-0.25,0.02) | 0.086 |
| **Random Effects** | | | | |
| σ^2^ | 0.15 | | 0.15 | |
| τ_00_ | 0.07 _id_ | | 0.07 _id_ | |
|  | 0.01 _News_ | | 0.01 _News_ | |
| τ_11_ | 0.01 _id.CongruentTRUE_ | | 0.00 _id.CongruentTRUE_ | |
|  | 0.10 _id.Conditionmessage myside bias_ | | 0.05 _id.Conditionmessage myside bias_ | |
| ρ_01_ | 0.14 _id.CongruentTRUE_ | | 1.00 _id.CongruentTRUE_ | |
|  | -0.62 _id.Conditionmessage myside bias_ | | -0.48 _id.Conditionmessage myside bias_ | |
| ICC | 0.32 | |  | |
| Marginal R^2^ / Conditional R^2^ | 0.026 / 0.338 | | 0.052 / NA | |

### Congruence x Issue moralization x Condition

| **Experiment 8 fake news** | | | |
| --- | --- | --- | --- |
|  | **sharing** | | |
| *Predictors* | *ß* | *p* | *std. p* |
| Congruent [TRUE] | 0.29 (0.21,0.37) | **<0.001** | **<0.001** |
| AbsImpIssue [TRUE] | 0.05 (-0.04,0.15) | 0.248 | 0.248 |
| Condition [message myside bias] | 0.05 (-0.07,0.16) | 0.426 | 0.426 |
| edu | 0.05 (0.01,0.10) | **0.025** | **0.025** |
| sex [female] | 0.04 (-0.05,0.14) | 0.351 | 0.351 |
| age | -0.06 (-0.10, -0.01) | **0.012** | **0.012** |
| Congruent [TRUE] * AbsImpIssue [TRUE] | 0.16 (0.04,0.28) | **0.007** | **0.007** |
| Congruent [TRUE] * Condition [message myside bias] | -0.12 (-0.23, -0.01) | **0.030** | **0.030** |
| AbsImpIssue [TRUE] * Condition [message myside bias] | -0.07 (-0.21,0.07) | 0.313 | 0.313 |
| (Congruent [TRUE] * AbsImpIssue [TRUE]) * Condition [message myside bias] | 0.09 (-0.08,0.27) | 0.282 | 0.282 |
| **Random Effects** | | | |
| σ^2^ | 0.13 | | |
| τ_00_ _id_ | 0.07 | | |
| τ_00_ _News_ | 0.00 | | |
| τ_11_ _id.CongruentTRUE_ | 0.02 | | |
| τ_11_ _id.AbsImpIssueTRUE_ | 0.02 | | |
| τ_11_ _id.Conditionmessage myside bias_ | 0.09 | | |
| ρ_01_ _id.CongruentTRUE_ | 0.01 | | |
| ρ_01_ _id.AbsImpIssueTRUE_ | -0.31 | | |
| ρ_01_ _id.Conditionmessage myside bias_ | -0.52 | | |
| ICC | 0.37 | | |
| Marginal R^2^ / Conditional R^2^ | 0.036 / 0.397 | | |

### Congruence x Attitude extremity x Condition

| **Experiment 8 fake news** | | | |
| --- | --- | --- | --- |
|  | **sharing** | | |
| *Predictors* | *ß* | *p* | *std. p* |
| Congruent [TRUE] | 0.34 (0.27,0.40) | 0.540 | **<0.001** |
| Attit Issue extr | -0.01 (-0.06,0.04) | 0.662 | 0.662 |
| Condition [message myside bias] | 0.02 (-0.08,0.12) | 0.128 | 0.717 |
| edu | 0.05 (0.01,0.10) | **0.025** | **0.025** |
| sex [female] | 0.05 (-0.05,0.14) | 0.328 | 0.328 |
| age | -0.06 (-0.11, -0.02) | **0.008** | **0.008** |
| Congruent [TRUE] * Attit Issue extr | 0.14 (0.08,0.19) | **<0.001** | **<0.001** |
| Congruent [TRUE] * Condition [message myside bias] | -0.09 (-0.17,0.00) | **0.023** | 0.056 |
| Attit Issue extr * Condition [message myside bias] | -0.05 (-0.12,0.01) | 0.108 | 0.108 |
| (Congruent [TRUE] * Attit Issue extr) * Condition [message myside bias] | 0.07 (-0.02,0.15) | 0.110 | 0.110 |
| **Random Effects** | | | |
| σ^2^ | 0.13 | | |
| τ_00_ _id_ | 0.07 | | |
| τ_00_ _News_ | 0.00 | | |
| τ_11_ _id.CongruentTRUE_ | 0.02 | | |
| τ_11_ _id.AbsImpIssueTRUE_ | 0.02 | | |
| τ_11_ _id.Conditionmessage myside bias_ | 0.09 | | |
| ρ_01_ _id.CongruentTRUE_ | 0.05 | | |
| ρ_01_ _id.AbsImpIssueTRUE_ | -0.32 | | |
| ρ_01_ _id.Conditionmessage myside bias_ | -0.51 | | |
| ICC | 0.37 | | |
| Marginal R^2^ / Conditional R^2^ | 0.039 / 0.399 | | |

# M. Experiment 9: Interactive intervention on the reputational risk of myside sharing, fake news

## Message in the control condition

Please read very carefully the following message. Questions will be asked to you about it.

New archeological evidence suggests prehistoric children as young as eight worked as brickmakers and miners. A surge of interest in the archaeology of childhood is revealing details of the skilled and sometimes back-breaking work that youngsters performed hundreds to thousands of years ago.

Their tasks included mining salt and forming bricks. Some children were already learning to create clay vessels by the time they were six years old. Researchers presented several of these findings at a meeting of the European Association of Archaeologists (EAA) in Barcelona, Spain, earlier this month.

Source:

Watson, T. (2018). *Nature.*561, pp. 445-46.

### Congruence x Issue moralization

| **Experiment 9 fake news** | | | | |
| --- | --- | --- | --- | --- |
|  | **sharing** | | **sharing** | |
| *Predictors* | *ß* | *p* | *ß* | *p* |
| Congruent [TRUE] | 0.47 (0.40,0.53) | **<0.001** | 0.36 (0.29,0.43) | **<0.001** |
| AbsImpIssue [TRUE] | 0.17 (0.12,0.22) | **<0.001** | 0.03 (-0.04,0.09) | 0.429 |
| Condition [message myside sharing] | -0.16 (-0.24, -0.07) | **<0.001** | -0.16 (-0.24, -0.07) | **<0.001** |
| edu | -0.10 (-0.14, -0.06) | **<0.001** | -0.10 (-0.14, -0.06) | **<0.001** |
| sex [female] | 0.12 (0.02,0.22) | **0.019** | 0.12 (0.02,0.22) | **0.015** |
| age | -0.05 (-0.09, -0.00) | **0.031** | -0.05 (-0.09, -0.01) | **0.023** |
| Congruent [TRUE] * AbsImpIssue [TRUE] |  |  | 0.32 (0.23,0.41) | **<0.001** |
| **Random Effects** | | | | |
| σ^2^ | 0.39 | | 0.39 | |
| τ_00_ | 0.20 _id_ | | 0.19 _id_ | |
|  | 0.03 _News_ | | 0.03 _News_ | |
| τ_11_ | 0.13 _id.CongruentTRUE_ | | 0.12 _id.CongruentTRUE_ | |
|  | 0.01 _id.AbsImpIssueTRUE_ | | 0.01 _id.AbsImpIssueTRUE_ | |
|  | 0.42 _id.Conditionmessage myside sharing_ | | 0.39 _id.Conditionmessage myside sharing_ | |
| ρ_01_ | 0.38 _id.CongruentTRUE_ | | 0.43 _id.CongruentTRUE_ | |
|  | 0.35 _id.AbsImpIssueTRUE_ | | 0.36 _id.AbsImpIssueTRUE_ | |
|  | -0.73 _id.Conditionmessage myside sharing_ | | -0.71 _id.Conditionmessage myside sharing_ | |
| ICC | 0.47 | | 0.47 | |
| Marginal R^2^ / Conditional R^2^ | 0.087 / 0.515 | | 0.106 / 0.524 | |

### Congruence x Attitude extremity

| **Experiment 9 - fake news** | | | | | | |
| --- | --- | --- | --- | --- | --- | --- |
|  | **sharing** | | | **sharing** | | |
| *Predictors* | *ß* | *p* | *std. p* | *ß* | *p* | *std. p* |
| Congruent [TRUE] | 0.47 (0.41,0.53) | **<0.001** | **<0.001** | 0.54 (0.48,0.61) | 0.936 | **<0.001** |
| Attit Issue extr | 0.04 (0.01,0.07) | **0.005** | **0.005** | -0.06 (-0.09, -0.03) | **0.001** | **0.001** |
| Condition [message myside sharing] | -0.16 (-0.25, -0.08) | **<0.001** | **<0.001** | -0.16 (-0.24, -0.07) | **<0.001** | **<0.001** |
| edu | -0.10 (-0.14, -0.05) | **<0.001** | **<0.001** | -0.10 (-0.14, -0.06) | **<0.001** | **<0.001** |
| sex [female] | 0.13 (0.03,0.23) | **0.010** | **0.010** | 0.14 (0.04,0.24) | **0.008** | **0.008** |
| age | -0.06 (-0.10, -0.01) | **0.009** | **0.009** | -0.06 (-0.10, -0.01) | **0.010** | **0.010** |
| Congruent [TRUE] * Attit Issue extr |  |  |  | 0.22 (0.18,0.26) | **<0.001** | **<0.001** |
| **Random Effects** | | | | | | |
| σ^2^ | 0.40 | | | 0.39 | | |
| τ_00_ | 0.24 _id_ | | | 0.23 _id_ | | |
|  | 0.03 _News_ | | | 0.03 _News_ | | |
| τ_11_ | 0.13 _id.CongruentTRUE_ | | | 0.11 _id.CongruentTRUE_ | | |
|  | 0.02 _id.Attit_Issue_extr_ | | | 0.03 _id.Attit_Issue_extr_ | | |
|  | 0.23 _id.Conditionmessage myside sharing_ | | | 0.14 _id.Conditionmessage myside sharing_ | | |
| ρ_01_ | 0.29 _id.CongruentTRUE_ | | | 0.44 _id.CongruentTRUE_ | | |
|  | -0.33 _id.Attit_Issue_extr_ | | | -0.38 _id.Attit_Issue_extr_ | | |
|  | -0.43 _id.Conditionmessage myside sharing_ | | | -0.32 _id.Conditionmessage myside sharing_ | | |
| ICC | 0.47 | | | 0.46 | | |
| Marginal R^2^ / Conditional R^2^ | 0.083 / 0.512 | | | 0.113 / 0.525 | | |

### Congruence x Issue moralization controlling for Congruence x Attitude extremity

| **Experiment 9 - fake news** | | | |
| --- | --- | --- | --- |
|  | **sharing** | | |
| *Predictors* | *ß* | *p* | *std. p* |
| Condition [message myside sharing] | -0.16 (-0.24, -0.07) | **<0.001** | **<0.001** |
| Congruent [TRUE] | 0.46 (0.39,0.54) | 0.865 | **<0.001** |
| AbsImpIssue [TRUE] | 0.08 (0.01,0.15) | **0.021** | **0.021** |
| Attit Issue extr | -0.08 (-0.12, -0.04) | **<0.001** | **<0.001** |
| edu | -0.10 (-0.14, -0.06) | **<0.001** | **<0.001** |
| sex [female] | 0.13 (0.03,0.23) | **0.013** | **0.013** |
| age | -0.05 (-0.09, -0.01) | **0.027** | **0.027** |
| Congruent [TRUE] * AbsImpIssue [TRUE] | 0.19 (0.10,0.28) | **<0.001** | **<0.001** |
| Congruent [TRUE] * Attit Issue extr | 0.18 (0.13,0.23) | **<0.001** | **<0.001** |
| **Random Effects** | | | |
| σ^2^ | 0.39 | | |
| τ_00_ _id_ | 0.19 | | |
| τ_00_ _News_ | 0.03 | | |
| τ_11_ _id.Conditionmessage myside sharing_ | 0.31 | | |
| τ_11_ _id.CongruentTRUE_ | 0.11 | | |
| τ_11_ _id.AbsImpIssueTRUE_ | 0.01 | | |
| ρ_01_ _id.Conditionmessage myside sharing_ | -0.64 | | |
| ρ_01_ _id.CongruentTRUE_ | 0.56 | | |
| ρ_01_ _id.AbsImpIssueTRUE_ | 0.35 | | |
| ICC | 0.47 | | |
| Marginal R^2^ / Conditional R^2^ | 0.123 / 0.532 | | |

### Congruence x Condition

| **Experiment 9 - fake news** | | |
| --- | --- | --- |
|  | **sharing** | |
| *Predictors* | *ß* | *p* |
| Congruent [TRUE] | 0.52 (0.44,0.60) | **<0.001** |
| Condition [message myside sharing] | -0.14 (-0.23, -0.05) | **0.001** |
| edu | -0.10 (-0.14, -0.05) | **<0.001** |
| sex [female] | 0.14 (0.04,0.24) | **0.007** |
| age | -0.06 (-0.10, -0.01) | **0.010** |
| Congruent [TRUE] * Condition [message myside sharing] | -0.09 (-0.19,0.00) | 0.053 |
| **Random Effects** | | |
| σ^2^ | 0.40 | |
| τ_00_ _id_ | 0.21 | |
| τ_00_ _News_ | 0.03 | |
| τ_11_ _id.CongruentTRUE_ | 0.12 | |
| τ_11_ _id.Conditionmessage myside sharing_ | 0.53 | |
| ρ_01_ _id.CongruentTRUE_ | 0.47 | |
| ρ_01_ _id.Conditionmessage myside sharing_ | -0.85 | |
| ICC | 0.46 | |
| Marginal R^2^ / Conditional R^2^ | 0.084 / 0.509 | |

### Effects of partisanship

| **Experiment 9 - fake news** | | | | | | | | | | | | | |
| --- | --- | --- | --- | --- | --- | --- | --- | --- | --- | --- | --- | --- | --- |
|  | **sharing** | | **sharing** | | **sharing** | | **sharing** | | | **sharing** | | **sharing** | |
| *Predictors* | *ß* | *p* | *ß* | *p* | *ß* | *p* | *ß* | *p* | *std. p* | *ß* | *p* | *ß* | *p* |
| Condition [message myside sharing] | -0.19 (-0.28, -0.10) | **<0.001** | -0.22 (-0.33, -0.10) | **<0.001** |  |  |  |  |  | -0.14 (-0.23, -0.05) | **0.001** | -0.18 (-0.30, -0.06) | **0.004** |
| edu | -0.10 (-0.14, -0.05) | **<0.001** | -0.10 (-0.15, -0.05) | **<0.001** | -0.10 (-0.14, -0.05) | **<0.001** | -0.10 (-0.15, -0.05) | **<0.001** | **<0.001** | -0.10 (-0.14, -0.05) | **<0.001** | -0.10 (-0.15, -0.05) | **<0.001** |
| sex [female] | 0.20 (0.09,0.31) | **<0.001** | 0.17 (0.05,0.29) | **0.005** | 0.20 (0.09,0.31) | **<0.001** | 0.16 (0.04,0.28) | **0.007** | **0.007** | 0.14 (0.04,0.24) | **0.007** | 0.17 (0.05,0.29) | **0.005** |
| age | -0.06 (-0.11, -0.02) | **0.005** | -0.08 (-0.13, -0.03) | **0.001** | -0.06 (-0.11, -0.01) | **0.010** | -0.08 (-0.13, -0.03) | **0.002** | **0.002** | -0.06 (-0.10, -0.01) | **0.010** | -0.08 (-0.13, -0.03) | **0.001** |
| PO bin [Republican] |  |  | -0.02 (-0.18,0.15) | 0.840 |  |  | 0.23 (0.09,0.36) | **0.001** | **0.001** |  |  | 0.13 (-0.06,0.31) | 0.174 |
| Condition [message myside sharing] * PO bin [Republican] |  |  | 0.12 (-0.12,0.36) | 0.331 |  |  |  |  |  |  |  | 0.16 (-0.10,0.43) | 0.216 |
| Congruent [TRUE] |  |  |  |  | 0.46 (0.40,0.52) | **<0.001** | 0.64 (0.55,0.72) | **<0.001** | **<0.001** | 0.52 (0.44,0.60) | **<0.001** | 0.67 (0.57,0.77) | **<0.001** |
| Congruent [TRUE] * PO bin [Republican] |  |  |  |  |  |  | -0.33 (-0.45, -0.21) | **<0.001** | **<0.001** |  |  | -0.29 (-0.45, -0.13) | **<0.001** |
| Congruent [TRUE] * Condition [message myside sharing] |  |  |  |  |  |  |  |  |  | -0.09 (-0.19,0.00) | 0.053 | -0.07 (-0.17,0.03) | 0.173 |
| (Congruent [TRUE] * Condition [message myside sharing]) * PO bin [Republican] |  |  |  |  |  |  |  |  |  |  |  | -0.09 (-0.31,0.12) | 0.394 |
| **Random Effects** | | | | | | | | | | | | | |
| σ^2^ | 0.46 | | 0.48 | | 0.44 | | 0.44 | | | 0.40 | | 0.44 | |
| τ_00_ | 0.31 _id_ | | 0.31 _id_ | | 0.32 _id_ | | 0.33 _id_ | | | 0.21 _id_ | | 0.32 _id_ | |
|  | 0.08 _News_ | | 0.09 _News_ | | 0.03 _News_ | | 0.03 _News_ | | | 0.03 _News_ | | 0.03 _News_ | |
| τ_11_ | 0.56 _id.Conditionmessage myside sharing_ | | 0.12 _id.PO_binRepublican_ | | 0.02 _id.Conditionmessage myside sharing_ | | 0.20 _id.PO_binRepublican_ | | | 0.12 _id.CongruentTRUE_ | | 0.23 _id.PO_binRepublican_ | |
|  |  | | 0.44 _id.Conditionmessage myside sharing_ | |  | | 0.58 _id.Conditionmessage myside sharing_ | | | 0.53 _id.Conditionmessage myside sharing_ | | 0.51 _id.Conditionmessage myside sharing_ | |
| ρ_01_ | -0.75 _id_ | | -0.40 _id.PO_binRepublican_ | | -0.55 _id_ | | -0.48 _id.PO_binRepublican_ | | | 0.47 _id.CongruentTRUE_ | | -0.49 _id.PO_binRepublican_ | |
|  |  | | -0.72 _id.Conditionmessage myside sharing_ | |  | | -0.78 _id.Conditionmessage myside sharing_ | | | -0.85 _id.Conditionmessage myside sharing_ | | -0.76 _id.Conditionmessage myside sharing_ | |
| ICC | 0.43 | | 0.43 | | 0.45 | | 0.44 | | | 0.46 | | 0.41 | |
| Marginal R^2^ / Conditional R^2^ | 0.031 / 0.451 | | 0.033 / 0.448 | | 0.073 / 0.487 | | 0.103 / 0.497 | | | 0.084 / 0.509 | | 0.117 / 0.475 | |

### Congruent x Condition on Democrats only (restricted analysis)

| **Experiment 9 - fake news - Democrats only** | | | | |
| --- | --- | --- | --- | --- |
|  | **sharing** | | **sharing** | |
| *Predictors* | *ß* | *p* | *ß* | *p* |
| Condition [message myside sharing] | -0.18 (-0.29, -0.08) | **<0.001** | -0.18 (-0.28, -0.08) | **0.001** |
| edu | -0.07 (-0.12, -0.02) | **0.009** | -0.07 (-0.12, -0.02) | **0.008** |
| sex [female] | 0.12 (-0.00,0.25) | 0.052 | 0.11 (-0.01,0.23) | 0.078 |
| age | -0.07 (-0.12, -0.02) | **0.004** | -0.07 (-0.12, -0.02) | **0.004** |
| Congruent [TRUE] |  |  | 0.35 (0.22,0.49) | **<0.001** |
| Congruent [TRUE] * Condition [message myside sharing] |  |  | -0.08 (-0.20,0.04) | 0.211 |
| **Random Effects** | | | | |
| σ^2^ | 0.35 | | 0.35 | |
| τ_00_ | 0.21 _id_ | | 0.21 _id_ | |
|  | 0.14 _News_ | | 0.07 _News_ | |
| τ_11_ | 0.18 _id.CongruentTRUE_ | | 0.17 _id.CongruentTRUE_ | |
|  | 0.44 _id.Conditionmessage myside sharing_ | | 0.38 _id.Conditionmessage myside sharing_ | |
| ρ_01_ | 0.39 _id.CongruentTRUE_ | | 0.42 _id.CongruentTRUE_ | |
|  | -0.88 _id.Conditionmessage myside sharing_ | | -0.84 _id.Conditionmessage myside sharing_ | |
| ICC | 0.46 | | 0.53 | |
| Marginal R^2^ / Conditional R^2^ | 0.027 / 0.477 | | 0.052 / 0.559 | |

### Congruent x Condition on Republicans only (restricted analysis)

| **Experiment 9 - fake news - Republicans only** | | | | |
| --- | --- | --- | --- | --- |
|  | **sharing** | | **sharing** | |
| *Predictors* | *ß* | *p* | *ß* | *p* |
| Condition [message myside sharing] | -0.02 (-0.22,0.19) | 0.883 | -0.01 (-0.22,0.21) | 0.960 |
| edu | -0.16 (-0.26, -0.06) | **0.002** | -0.16 (-0.26, -0.06) | **0.002** |
| sex [female] | 0.15 (-0.08,0.37) | 0.193 | 0.14 (-0.08,0.37) | 0.207 |
| age | -0.08 (-0.18,0.02) | 0.113 | -0.08 (-0.18,0.02) | 0.117 |
| Congruent [TRUE] |  |  | 0.33 (0.19,0.48) | **<0.001** |
| Congruent [TRUE] * Condition [message myside sharing] |  |  | -0.15 (-0.35,0.05) | 0.147 |
| **Random Effects** | | | | |
| σ^2^ | 0.54 | | 0.53 | |
| τ_00_ | 0.18 _id_ | | 0.19 _id_ | |
|  | 0.07 _News_ | | 0.06 _News_ | |
| τ_11_ | 0.05 _id.CongruentTRUE_ | | 0.02 _id.CongruentTRUE_ | |
|  | 0.57 _id.Conditionmessage myside sharing_ | | 0.51 _id.Conditionmessage myside sharing_ | |
| ρ_01_ | 0.77 _id.CongruentTRUE_ | | 1.00 _id.CongruentTRUE_ | |
|  | -0.68 _id.Conditionmessage myside sharing_ | | -0.63 _id.Conditionmessage myside sharing_ | |
| ICC | 0.37 | | 0.40 | |
| Marginal R^2^ / Conditional R^2^ | 0.044 / 0.398 | | 0.063 / 0.435 | |

### Congruence x Issue moralization x Condition

| **Experiment 9 - fake news** | | | |
| --- | --- | --- | --- |
|  | **sharing** | | |
| *Predictors* | *ß* | *p* | *std. p* |
| Congruent [TRUE] | 0.42 (0.32,0.51) | **<0.001** | **<0.001** |
| AbsImpIssue [TRUE] | 0.08 (-0.01,0.17) | 0.095 | 0.095 |
| Condition [message myside sharing] | -0.09 (-0.20,0.01) | 0.082 | 0.082 |
| edu | -0.10 (-0.14, -0.06) | **<0.001** | **<0.001** |
| sex [female] | 0.12 (0.02,0.22) | **0.015** | **0.015** |
| age | -0.05 (-0.09, -0.01) | **0.021** | **0.021** |
| Congruent [TRUE] * AbsImpIssue [TRUE] | 0.29 (0.17,0.41) | **<0.001** | **<0.001** |
| Congruent [TRUE] * Condition [message myside sharing] | -0.12 (-0.24,0.00) | 0.056 | 0.056 |
| AbsImpIssue [TRUE] * Condition [message myside sharing] | -0.10 (-0.23,0.02) | 0.116 | 0.116 |
| (Congruent [TRUE] * AbsImpIssue [TRUE]) * Condition [message myside sharing] | 0.06 (-0.11,0.23) | 0.475 | 0.475 |
| **Random Effects** | | | |
| σ^2^ | 0.39 | | |
| τ_00_ _id_ | 0.20 | | |
| τ_00_ _News_ | 0.03 | | |
| τ_11_ _id.CongruentTRUE_ | 0.12 | | |
| τ_11_ _id.Conditionmessage myside sharing_ | 0.41 | | |
| τ_11_ _id.AbsImpIssueTRUE_ | 0.01 | | |
| ρ_01_ _id.CongruentTRUE_ | 0.43 | | |
| ρ_01_ _id.Conditionmessage myside sharing_ | -0.72 | | |
| ρ_01_ _id.AbsImpIssueTRUE_ | 0.38 | | |
| ICC | 0.47 | | |
| Marginal R^2^ / Conditional R^2^ | 0.108 / 0.525 | | |

### Congruence x Attitude extremity x Condition

| **Experiment 9 - fake news** | | | |
| --- | --- | --- | --- |
|  | **sharing** | | |
| *Predictors* | *ß* | *p* | *std. p* |
| Congruent [TRUE] | 0.59 (0.51,0.67) | 0.496 | **<0.001** |
| Attit Issue extr | -0.04 (-0.09,0.00) | 0.064 | 0.064 |
| Condition [message myside sharing] | -0.14 (-0.23, -0.06) | 0.569 | **0.001** |
| edu | -0.10 (-0.14, -0.06) | **<0.001** | **<0.001** |
| sex [female] | 0.14 (0.04,0.24) | **0.008** | **0.008** |
| age | -0.06 (-0.10, -0.01) | **0.009** | **0.009** |
| Congruent [TRUE] * Attit Issue extr | 0.21 (0.15,0.27) | **<0.001** | **<0.001** |
| Congruent [TRUE] * Condition [message myside sharing] | -0.09 (-0.19, -0.00) | 0.284 | **0.046** |
| Attit Issue extr * Condition [message myside sharing] | -0.04 (-0.10,0.03) | 0.280 | 0.280 |
| (Congruent [TRUE] * Attit Issue extr) * Condition [message myside sharing] | 0.01 (-0.07,0.09) | 0.781 | 0.781 |
| **Random Effects** | | | |
| σ^2^ | 0.39 | | |
| τ_00_ _id_ | 0.24 | | |
| τ_00_ _News_ | 0.03 | | |
| τ_11_ _id.CongruentTRUE_ | 0.10 | | |
| τ_11_ _id.Conditionmessage myside sharing_ | 0.13 | | |
| τ_11_ _id.Attit_Issue_extr_ | 0.03 | | |
| ρ_01_ _id.CongruentTRUE_ | 0.44 | | |
| ρ_01_ _id.Conditionmessage myside sharing_ | -0.32 | | |
| ρ_01_ _id.Attit_Issue_extr_ | -0.39 | | |
| ICC | 0.46 | | |
| Marginal R^2^ / Conditional R^2^ | 0.116 / 0.527 | | |

# N. Experiment 10: Interactive intervention on the reputational risk of myside sharing, true and fake news

## Message in the control condition

Please read very carefully the following message. Questions will be asked to you about it.

New archeological evidence suggests prehistoric children as young as eight worked as brickmakers and miners. A surge of interest in the archaeology of childhood is revealing details of the skilled and sometimes back-breaking work that youngsters performed hundreds to thousands of years ago.

Their tasks included mining salt and forming bricks. Some children were already learning to create clay vessels by the time they were six years old. Researchers presented several of these findings at a meeting of the European Association of Archaeologists (EAA) in Barcelona, Spain, earlier this month.

Source:

Watson, T. (2018). *Nature.*561, pp. 445-46.

### Congruence x Issue moralization, Congruence x Attitude extremity, Congruence x Issue moralization controlling for Congruence x Attitude extremity

| **Experiment 10 true political news** | | | | | | | | | | | | |
| --- | --- | --- | --- | --- | --- | --- | --- | --- | --- | --- | --- | --- |
|  | **SHARING** | | **SHARING** | | **SHARING** | | **SHARING** | | | **SHARING** | | |
| *Predictors* | *ß* | *p* | *ß* | *p* | *ß* | *p* | *ß* | *p* | *std. p* | *ß* | *p* | *std. p* |
| Congruent [TRUE] | 0.48 (0.43,0.52) | **<0.001** | 0.38 (0.33,0.43) | **<0.001** | 0.48 (0.43,0.52) | **<0.001** | 0.49 (0.44,0.53) | 0.094 | **<0.001** | 0.44 (0.39,0.49) | **0.046** | **<0.001** |
| AbsImpIssue [TRUE] | 0.16 (0.12,0.20) | **<0.001** | 0.04 (-0.01,0.09) | 0.103 |  |  |  |  |  | 0.09 (0.04,0.14) | **0.001** | **0.001** |
| Condition [message myside sharing] | -0.17 (-0.24, -0.09) | **<0.001** | -0.17 (-0.24, -0.09) | **<0.001** | -0.17 (-0.24, -0.09) | **<0.001** | -0.17 (-0.24, -0.09) | **<0.001** | **<0.001** | -0.17 (-0.24, -0.09) | **<0.001** | **<0.001** |
| edu | -0.07 (-0.10, -0.03) | **0.001** | -0.07 (-0.10, -0.03) | **0.001** | -0.07 (-0.10, -0.03) | **0.001** | -0.06 (-0.10, -0.03) | **0.001** | **0.001** | -0.07 (-0.10, -0.03) | **0.001** | **0.001** |
| sex [female] | -0.14 (-0.21, -0.06) | **<0.001** | -0.13 (-0.21, -0.05) | **0.001** | -0.13 (-0.21, -0.06) | **0.001** | -0.12 (-0.20, -0.05) | **0.001** | **0.001** | -0.13 (-0.20, -0.05) | **0.001** | **0.001** |
| age | -0.05 (-0.08, -0.01) | **0.019** | -0.05 (-0.09, -0.01) | **0.015** | -0.05 (-0.09, -0.01) | **0.013** | -0.05 (-0.09, -0.01) | **0.012** | **0.012** | -0.05 (-0.08, -0.01) | **0.016** | **0.016** |
| Congruent [TRUE] * AbsImpIssue [TRUE] |  |  | 0.25 (0.19,0.32) | **<0.001** |  |  |  |  |  | 0.13 (0.06,0.20) | **<0.001** | **<0.001** |
| Attit Issue extr |  |  |  |  | 0.04 (0.02,0.06) | **<0.001** | -0.04 (-0.07, -0.02) | **<0.001** | **<0.001** | -0.06 (-0.09, -0.04) | **<0.001** | **<0.001** |
| Congruent [TRUE] * Attit Issue extr |  |  |  |  |  |  | 0.18 (0.15,0.21) | **<0.001** | **<0.001** | 0.15 (0.12,0.19) | **<0.001** | **<0.001** |
| **Random Effects** | | | | | | | | | | | | |
| σ^2^ | 0.50 | | 0.50 | | 0.50 | | 0.50 | | | 0.49 | | |
| τ_00_ | 0.26 _id_ | | 0.26 _id_ | | 0.26 _id_ | | 0.26 _id_ | | | 0.26 _id_ | | |
|  | 0.04 _News_ | | 0.04 _News_ | | 0.04 _News_ | | 0.04 _News_ | | | 0.04 _News_ | | |
| τ_11_ | 0.20 _id.CongruentTRUE_ | | 0.19 _id.CongruentTRUE_ | | 0.20 _id.CongruentTRUE_ | | 0.17 _id.CongruentTRUE_ | | | 0.17 _id.CongruentTRUE_ | | |
| ρ_01_ | 0.17 _id_ | | 0.19 _id_ | | 0.18 _id_ | | 0.25 _id_ | | | 0.24 _id_ | | |
| ICC | 0.47 | | 0.47 | | 0.47 | | 0.47 | | | 0.47 | | |
| Marginal R^2^ / Conditional R^2^ | 0.080 / 0.511 | | 0.085 / 0.514 | | 0.075 / 0.513 | | 0.085 / 0.516 | | | 0.091 / 0.515 | | |

| **Experiment 10 fake political news** | | | | | | | | | | | | |
| --- | --- | --- | --- | --- | --- | --- | --- | --- | --- | --- | --- | --- |
|  | **SHARING** | | **SHARING** | | **SHARING** | | **SHARING** | | | **SHARING** | | |
| *Predictors* | *ß* | *p* | *ß* | *p* | *ß* | *p* | *ß* | *p* | *std. p* | *ß* | *p* | *std. p* |
| Congruent [TRUE] | 0.55 (0.51,0.60) | **<0.001** | 0.45 (0.39,0.50) | **<0.001** | 0.56 (0.51,0.60) | **<0.001** | 0.57 (0.52,0.61) | 0.830 | **<0.001** | 0.53 (0.47,0.58) | 0.642 | **<0.001** |
| AbsImpIssue [TRUE] | 0.18 (0.14,0.21) | **<0.001** | 0.04 (-0.01,0.08) | 0.103 |  |  |  |  |  | 0.10 (0.05,0.16) | **<0.001** | **<0.001** |
| Condition [message myside sharing] | -0.20 (-0.27, -0.12) | **<0.001** | -0.20 (-0.27, -0.12) | **<0.001** | -0.19 (-0.27, -0.12) | **<0.001** | -0.19 (-0.27, -0.12) | **<0.001** | **<0.001** | -0.20 (-0.27, -0.12) | **<0.001** | **<0.001** |
| edu | -0.08 (-0.11, -0.04) | **<0.001** | -0.08 (-0.11, -0.04) | **<0.001** | -0.08 (-0.11, -0.04) | **<0.001** | -0.07 (-0.11, -0.04) | **<0.001** | **<0.001** | -0.08 (-0.11, -0.04) | **<0.001** | **<0.001** |
| sex [female] | -0.07 (-0.15,0.00) | 0.062 | -0.07 (-0.14,0.01) | 0.081 | -0.07 (-0.15,0.00) | 0.063 | -0.06 (-0.14,0.01) | 0.110 | 0.110 | -0.06 (-0.14,0.01) | 0.102 | 0.102 |
| age | -0.03 (-0.06,0.01) | 0.165 | -0.03 (-0.07,0.01) | 0.141 | -0.03 (-0.07,0.01) | 0.117 | -0.03 (-0.07,0.01) | 0.124 | 0.124 | -0.03 (-0.06,0.01) | 0.159 | 0.159 |
| Congruent [TRUE] * AbsImpIssue [TRUE] |  |  | 0.28 (0.22,0.35) | **<0.001** |  |  |  |  |  | 0.10 (0.03,0.17) | **0.004** | **0.004** |
| Attit Issue extr |  |  |  |  | 0.06 (0.04,0.08) | **<0.001** | -0.06 (-0.08, -0.03) | **<0.001** | **<0.001** | -0.08 (-0.10, -0.05) | **<0.001** | **<0.001** |
| Congruent [TRUE] * Attit Issue extr |  |  |  |  |  |  | 0.24 (0.21,0.27) | **<0.001** | **<0.001** | 0.22 (0.18,0.25) | **<0.001** | **<0.001** |
| **Random Effects** | | | | | | | | | | | | |
| σ^2^ | 0.45 | | 0.45 | | 0.45 | | 0.45 | | | 0.44 | | |
| τ_00_ | 0.24 _id_ | | 0.24 _id_ | | 0.24 _id_ | | 0.23 _id_ | | | 0.23 _id_ | | |
|  | 0.02 _News_ | | 0.02 _News_ | | 0.02 _News_ | | 0.02 _News_ | | | 0.02 _News_ | | |
| τ_11_ | 0.26 _id.CongruentTRUE_ | | 0.24 _id.CongruentTRUE_ | | 0.26 _id.CongruentTRUE_ | | 0.22 _id.CongruentTRUE_ | | | 0.22 _id.CongruentTRUE_ | | |
| ρ_01_ | 0.05 _id_ | | 0.07 _id_ | | 0.05 _id_ | | 0.14 _id_ | | | 0.13 _id_ | | |
| ICC | 0.47 | | 0.47 | | 0.47 | | 0.47 | | | 0.47 | | |
| Marginal R^2^ / Conditional R^2^ | 0.100 / 0.523 | | 0.107 / 0.525 | | 0.096 / 0.522 | | 0.113 / 0.529 | | | 0.119 / 0.531 | | |

### Congruence x Issue moralization, Congruence x Attitude extremity, Congruence x Issue moralization controlling for Congruence x Attitude extremity on 8 first items viewed

| **Experiment 10 true political news - 8 first items only** | | | | | | | | | | | | |
| --- | --- | --- | --- | --- | --- | --- | --- | --- | --- | --- | --- | --- |
|  | **SHARING** | | **SHARING** | | **SHARING** | | **SHARING** | | | **SHARING** | | |
| *Predictors* | *ß* | *p* | *ß* | *p* | *ß* | *p* | *ß* | *p* | *std. p* | *ß* | *p* | *std. p* |
| Congruent [TRUE] | 0.49 (0.43,0.56) | **<0.001** | 0.42 (0.35,0.50) | **<0.001** | 0.50 (0.44,0.56) | **<0.001** | 0.51 (0.45,0.57) | 0.104 | **<0.001** | 0.48 (0.40,0.56) | 0.101 | **<0.001** |
| AbsImpIssue [TRUE] | 0.20 (0.14,0.27) | **<0.001** | 0.12 (0.04,0.20) | **0.003** |  |  |  |  |  | 0.16 (0.08,0.25) | **<0.001** | **<0.001** |
| Condition [message myside sharing] | -0.18 (-0.27, -0.09) | **<0.001** | -0.18 (-0.27, -0.09) | **<0.001** | -0.18 (-0.27, -0.09) | **<0.001** | -0.18 (-0.27, -0.09) | **<0.001** | **<0.001** | -0.18 (-0.27, -0.09) | **<0.001** | **<0.001** |
| edu | -0.07 (-0.11, -0.02) | **0.004** | -0.06 (-0.11, -0.02) | **0.004** | -0.06 (-0.11, -0.02) | **0.005** | -0.06 (-0.11, -0.02) | **0.006** | **0.006** | -0.06 (-0.11, -0.02) | **0.004** | **0.004** |
| sex [female] | -0.06 (-0.15,0.02) | 0.155 | -0.06 (-0.15,0.03) | 0.177 | -0.06 (-0.15,0.03) | 0.185 | -0.06 (-0.15,0.03) | 0.216 | 0.216 | -0.06 (-0.15,0.03) | 0.168 | 0.168 |
| age | -0.06 (-0.11, -0.02) | **0.007** | -0.06 (-0.11, -0.02) | **0.006** | -0.07 (-0.11, -0.02) | **0.004** | -0.06 (-0.11, -0.02) | **0.005** | **0.005** | -0.06 (-0.10, -0.02) | **0.008** | **0.008** |
| Congruent [TRUE] * AbsImpIssue [TRUE] |  |  | 0.19 (0.08,0.30) | **0.001** |  |  |  |  |  | 0.05 (-0.07,0.18) | 0.373 | 0.373 |
| Attit Issue extr |  |  |  |  | 0.06 (0.03,0.09) | **<0.001** | -0.01 (-0.05,0.03) | 0.495 | 0.495 | -0.05 (-0.09, -0.01) | **0.026** | **0.026** |
| Congruent [TRUE] * Attit Issue extr |  |  |  |  |  |  | 0.17 (0.12,0.22) | **<0.001** | **<0.001** | 0.16 (0.10,0.22) | **<0.001** | **<0.001** |
| **Random Effects** | | | | | | | | | | | | |
| σ^2^ | 0.47 | | 0.47 | | 0.47 | | 0.47 | | | 0.47 | | |
| τ_00_ | 0.25 _id_ | | 0.25 _id_ | | 0.25 _id_ | | 0.25 _id_ | | | 0.24 _id_ | | |
|  | 0.04 _News_ | | 0.04 _News_ | | 0.05 _News_ | | 0.05 _News_ | | | 0.04 _News_ | | |
| τ_11_ | 0.11 _id.CongruentTRUE_ | | 0.10 _id.CongruentTRUE_ | | 0.13 _id.CongruentTRUE_ | | 0.10 _id.CongruentTRUE_ | | | 0.08 _id.CongruentTRUE_ | | |
| ρ_01_ | 0.41 _id_ | | 0.44 _id_ | | 0.37 _id_ | | 0.51 _id_ | | | 0.55 _id_ | | |
| ICC | 0.47 | | 0.46 | | 0.48 | | 0.47 | | | 0.46 | | |
| Marginal R^2^ / Conditional R^2^ | 0.088 / 0.513 | | 0.092 / 0.514 | | 0.082 / 0.519 | | 0.092 / 0.520 | | | 0.099 / 0.515 | | |

| **Experiment 10 fake political news - 8 first items only** | | | | | | | | | | | | |
| --- | --- | --- | --- | --- | --- | --- | --- | --- | --- | --- | --- | --- |
|  | **SHARING** | | **SHARING** | | **SHARING** | | **SHARING** | | | **SHARING** | | |
| *Predictors* | *ß* | *p* | *ß* | *p* | *ß* | *p* | *ß* | *p* | *std. p* | *ß* | *p* | *std. p* |
| Congruent [TRUE] | 0.54 (0.47,0.60) | **<0.001** | 0.45 (0.37,0.52) | **<0.001** | 0.54 (0.47,0.61) | **<0.001** | 0.55 (0.49,0.62) | 0.743 | **<0.001** | 0.53 (0.45,0.61) | 0.757 | **<0.001** |
| AbsImpIssue [TRUE] | 0.19 (0.13,0.25) | **<0.001** | 0.08 (0.00,0.16) | **0.038** |  |  |  |  |  | 0.16 (0.07,0.24) | **<0.001** | **<0.001** |
| Condition [message myside sharing] | -0.19 (-0.27, -0.10) | **<0.001** | -0.19 (-0.27, -0.10) | **<0.001** | -0.19 (-0.27, -0.10) | **<0.001** | -0.18 (-0.27, -0.10) | **<0.001** | **<0.001** | -0.19 (-0.27, -0.10) | **<0.001** | **<0.001** |
| edu | -0.09 (-0.13, -0.05) | **<0.001** | -0.09 (-0.13, -0.05) | **<0.001** | -0.09 (-0.13, -0.05) | **<0.001** | -0.09 (-0.13, -0.05) | **<0.001** | **<0.001** | -0.09 (-0.13, -0.05) | **<0.001** | **<0.001** |
| sex [female] | -0.09 (-0.17, -0.00) | **0.050** | -0.08 (-0.17,0.00) | 0.053 | -0.08 (-0.17,0.00) | 0.057 | -0.08 (-0.17,0.01) | 0.066 | 0.066 | -0.08 (-0.17,0.00) | 0.056 | 0.056 |
| age | -0.02 (-0.06,0.02) | 0.410 | -0.02 (-0.06,0.02) | 0.395 | -0.02 (-0.06,0.02) | 0.327 | -0.02 (-0.06,0.02) | 0.364 | 0.364 | -0.02 (-0.06,0.03) | 0.443 | 0.443 |
| Congruent [TRUE] * AbsImpIssue [TRUE] |  |  | 0.24 (0.13,0.35) | **<0.001** |  |  |  |  |  | 0.05 (-0.07,0.17) | 0.383 | 0.383 |
| Attit Issue extr |  |  |  |  | 0.05 (0.02,0.08) | **0.003** | -0.05 (-0.09, -0.02) | **0.006** | **0.006** | -0.09 (-0.13, -0.05) | **<0.001** | **<0.001** |
| Congruent [TRUE] * Attit Issue extr |  |  |  |  |  |  | 0.23 (0.17,0.28) | **<0.001** | **<0.001** | 0.22 (0.16,0.28) | **<0.001** | **<0.001** |
| **Random Effects** | | | | | | | | | | | | |
| σ^2^ | 0.43 | | 0.43 | | 0.43 | | 0.42 | | | 0.42 | | |
| τ_00_ | 0.20 _id_ | | 0.20 _id_ | | 0.21 _id_ | | 0.20 _id_ | | | 0.20 _id_ | | |
|  | 0.02 _News_ | | 0.02 _News_ | | 0.02 _News_ | | 0.02 _News_ | | | 0.02 _News_ | | |
| τ_11_ | 0.26 _id.CongruentTRUE_ | | 0.25 _id.CongruentTRUE_ | | 0.27 _id.CongruentTRUE_ | | 0.24 _id.CongruentTRUE_ | | | 0.24 _id.CongruentTRUE_ | | |
| ρ_01_ | 0.15 _id_ | | 0.17 _id_ | | 0.15 _id_ | | 0.22 _id_ | | | 0.22 _id_ | | |
| ICC | 0.48 | | 0.48 | | 0.48 | | 0.48 | | | 0.47 | | |
| Marginal R^2^ / Conditional R^2^ | 0.098 / 0.529 | | 0.104 / 0.530 | | 0.092 / 0.528 | | 0.109 / 0.534 | | | 0.116 / 0.536 | | |

### By issue analyses

| **Experiment 10 true political news - Abortion issue only** | | | | | | | | | | | | |
| --- | --- | --- | --- | --- | --- | --- | --- | --- | --- | --- | --- | --- |
|  | **sharing** | | **sharing** | | **sharing** | | **sharing** | | | **sharing** | | |
| *Predictors* | *ß* | *p* | *ß* | *p* | *ß* | *p* | *ß* | *p* | *std. p* | *ß* | *p* | *std. p* |
| Congruent [TRUE] | 0.26 (0.19,0.33) | **<0.001** | 0.21 (0.10,0.32) | **<0.001** | 0.26 (0.19,0.32) | **<0.001** | 0.26 (0.19,0.33) | 0.718 | **<0.001** | 0.27 (0.15,0.39) | 0.734 | **<0.001** |
| AbsImpIssue [TRUE] | 0.39 (0.28,0.50) | **<0.001** | 0.35 (0.22,0.48) | **<0.001** |  |  |  |  |  | 0.31 (0.16,0.46) | **<0.001** | **<0.001** |
| Condition [message myside sharing] | -0.07 (-0.18,0.03) | 0.178 | -0.07 (-0.18,0.03) | 0.178 | -0.07 (-0.17,0.04) | 0.225 | -0.07 (-0.17,0.04) | 0.225 | 0.225 | -0.07 (-0.17,0.03) | 0.190 | 0.190 |
| edu | -0.04 (-0.10,0.01) | 0.094 | -0.04 (-0.10,0.01) | 0.094 | -0.04 (-0.10,0.01) | 0.101 | -0.04 (-0.10,0.01) | 0.101 | 0.101 | -0.05 (-0.10,0.01) | 0.085 | 0.085 |
| sex [female] | -0.10 (-0.21,0.01) | 0.065 | -0.10 (-0.21,0.01) | 0.065 | -0.07 (-0.18,0.03) | 0.182 | -0.07 (-0.18,0.03) | 0.182 | 0.182 | -0.11 (-0.22, -0.00) | **0.044** | **0.044** |
| age | -0.03 (-0.08,0.03) | 0.341 | -0.03 (-0.08,0.03) | 0.341 | -0.03 (-0.08,0.02) | 0.264 | -0.03 (-0.08,0.02) | 0.264 | 0.264 | -0.02 (-0.07,0.03) | 0.414 | 0.414 |
| Congruent [TRUE] * AbsImpIssue [TRUE] |  |  | 0.08 (-0.05,0.21) | 0.241 |  |  |  |  |  | -0.01 (-0.17,0.14) | 0.891 | 0.891 |
| Attit Issue extr |  |  |  |  | 0.15 (0.10,0.21) | **<0.001** | 0.12 (0.05,0.18) | **<0.001** | **<0.001** | 0.04 (-0.03,0.11) | 0.282 | 0.282 |
| Congruent [TRUE] * Attit Issue extr |  |  |  |  |  |  | 0.08 (0.01,0.14) | **0.017** | **0.017** | 0.08 (0.01,0.16) | **0.035** | **0.035** |
| **Random Effects** | | | | | | | | | | | | |
| σ^2^ | 0.57 | | 0.57 | | 0.57 | | 0.57 | | | 0.57 | | |
| τ_00_ | 0.50 _id_ | | 0.50 _id_ | | 0.51 _id_ | | 0.51 _id_ | | | 0.49 _id_ | | |
|  | 0.10 _News_ | | 0.10 _News_ | | 0.10 _News_ | | 0.10 _News_ | | | 0.10 _News_ | | |
| ICC | 0.51 | | 0.51 | | 0.51 | | 0.52 | | | 0.51 | | |
| Marginal R^2^ / Conditional R^2^ | 0.053 / 0.534 | | 0.053 / 0.535 | | 0.042 / 0.5  35 | | 0.044 / 0.538 | | | 0.059 / 0.537 | | |

| **Experiment 10 fake political news - Abortion issue only** | | | | | | | | | | | | | | |
| --- | --- | --- | --- | --- | --- | --- | --- | --- | --- | --- | --- | --- | --- | --- |
|  | **sharing** | | | **sharing** | | | **sharing** | | **sharing** | | | **sharing** | | |
| *Predictors* | *ß* | *p* | *std. p* | *ß* | *p* | *std. p* | *ß* | *p* | *ß* | *p* | *std. p* | *ß* | *p* | *std. p* |
| Congruent [TRUE] | 0.42 (0.35,0.50) | **<0.001** | **<0.001** | 0.26 (0.14,0.38) | **<0.001** | **<0.001** | 0.42 (0.35,0.50) | **<0.001** | 0.43 (0.35,0.50) | 0.649 | **<0.001** | 0.36 (0.23,0.49) | 0.704 | **<0.001** |
| AbsImpIssue [TRUE] | 0.18 (0.07,0.28) | **0.001** | **0.001** | 0.05 (-0.08,0.18) | 0.472 | 0.472 |  |  |  |  |  | 0.08 (-0.07,0.24) | 0.281 | 0.281 |
| Condition [message myside sharing] | -0.13 (-0.23, -0.03) | **0.011** | **0.011** | -0.13 (-0.23, -0.03) | **0.011** | **0.011** | -0.13 (-0.23, -0.03) | **0.015** | -0.13 (-0.23, -0.03) | **0.015** | **0.015** | -0.13 (-0.23, -0.03) | **0.012** | **0.012** |
| edu | -0.08 (-0.13, -0.03) | **0.001** | **0.001** | -0.08 (-0.13, -0.03) | **0.001** | **0.001** | -0.08 (-0.13, -0.03) | **0.001** | -0.08 (-0.13, -0.03) | **0.001** | **0.001** | -0.08 (-0.14, -0.03) | **0.001** | **0.001** |
| sex [female] | -0.06 (-0.16,0.05) | 0.266 | 0.266 | -0.06 (-0.16,0.05) | 0.266 | 0.266 | -0.05 (-0.15,0.05) | 0.354 | -0.05 (-0.15,0.05) | 0.354 | 0.354 | -0.06 (-0.17,0.04) | 0.235 | 0.235 |
| age | -0.07 (-0.13, -0.02) | **0.005** | **0.005** | -0.07 (-0.13, -0.02) | **0.005** | **0.005** | -0.08 (-0.13, -0.02) | **0.004** | -0.08 (-0.13, -0.02) | **0.004** | **0.004** | -0.07 (-0.12, -0.02) | **0.006** | **0.006** |
| Congruent [TRUE] * AbsImpIssue [TRUE] |  |  |  | 0.26 (0.11,0.40) | **<0.001** | **<0.001** |  |  |  |  |  | 0.10 (-0.06,0.27) | 0.226 | 0.226 |
| Attit Issue extr |  |  |  |  |  |  | 0.07 (0.02,0.12) | **0.007** | -0.01 (-0.07,0.05) | 0.723 | 0.723 | -0.03 (-0.10,0.04) | 0.400 | 0.400 |
| Congruent [TRUE] * Attit Issue extr |  |  |  |  |  |  |  |  | 0.16 (0.10,0.23) | **<0.001** | **<0.001** | 0.14 (0.06,0.22) | **0.001** | **0.001** |
| **Random Effects** | | | | | | | | | | | | | | |
| σ^2^ | 0.56 | | | 0.55 | | | 0.56 | | 0.55 | | | 0.55 | | |
| τ_00_ | 0.31 _id_ | | | 0.32 _id_ | | | 0.31 _id_ | | 0.32 _id_ | | | 0.32 _id_ | | |
|  | 0.01 _News_ | | | 0.01 _News_ | | | 0.01 _News_ | | 0.01 _News_ | | | 0.01 _News_ | | |
| ICC | 0.36 | | | 0.37 | | | 0.37 | | 0.38 | | | 0.37 | | |
| Marginal R^2^ / Conditional R^2^ | 0.071 / 0.409 | | | 0.074 / 0.416 | | | 0.068 / 0.409 | | 0.075 / 0.423 | | | 0.079 / 0.424 | | |

| **Experiment 10 true political news - Gun control issue only** | | | | | | | | | | | | |
| --- | --- | --- | --- | --- | --- | --- | --- | --- | --- | --- | --- | --- |
|  | **sharing** | | **sharing** | | **sharing** | | **sharing** | | | **sharing** | | |
| *Predictors* | *ß* | *p* | *ß* | *p* | *ß* | *p* | *ß* | *p* | *std. p* | *ß* | *p* | *std. p* |
| Congruent [TRUE] | 0.60 (0.53,0.67) | **<0.001** | 0.45 (0.36,0.53) | **<0.001** | 0.60 (0.53,0.67) | **<0.001** | 0.61 (0.54,0.68) | 0.418 | **<0.001** | 0.54 (0.45,0.63) | 0.563 | **<0.001** |
| AbsImpIssue [TRUE] | 0.10 (-0.00,0.20) | 0.055 | -0.11 (-0.23,0.01) | 0.085 |  |  |  |  |  | -0.03 (-0.16,0.11) | 0.710 | 0.710 |
| Condition [message myside sharing] | -0.17 (-0.27, -0.08) | **<0.001** | -0.17 (-0.27, -0.08) | **<0.001** | -0.17 (-0.27, -0.08) | **<0.001** | -0.17 (-0.27, -0.08) | **<0.001** | **<0.001** | -0.18 (-0.27, -0.08) | **<0.001** | **<0.001** |
| edu | -0.10 (-0.14, -0.05) | **<0.001** | -0.10 (-0.14, -0.05) | **<0.001** | -0.10 (-0.15, -0.05) | **<0.001** | -0.10 (-0.15, -0.05) | **<0.001** | **<0.001** | -0.10 (-0.15, -0.05) | **<0.001** | **<0.001** |
| sex [female] | -0.12 (-0.21, -0.02) | **0.018** | -0.12 (-0.21, -0.02) | **0.018** | -0.13 (-0.22, -0.03) | **0.011** | -0.13 (-0.22, -0.03) | **0.011** | **0.011** | -0.12 (-0.22, -0.02) | **0.015** | **0.015** |
| age | -0.04 (-0.09,0.01) | 0.093 | -0.04 (-0.09,0.01) | 0.093 | -0.04 (-0.09,0.00) | 0.073 | -0.04 (-0.09,0.00) | 0.073 | 0.073 | -0.04 (-0.09,0.00) | 0.076 | 0.076 |
| Congruent [TRUE] * AbsImpIssue [TRUE] |  |  | 0.41 (0.27,0.55) | **<0.001** |  |  |  |  |  | 0.19 (0.03,0.34) | **0.017** | **0.017** |
| Attit Issue extr |  |  |  |  | 0.05 (0.00,0.10) | **0.044** | -0.10 (-0.16, -0.04) | **0.001** | **0.001** | -0.09 (-0.16, -0.03) | **0.006** | **0.006** |
| Congruent [TRUE] * Attit Issue extr |  |  |  |  |  |  | 0.29 (0.23,0.36) | **<0.001** | **<0.001** | 0.25 (0.18,0.33) | **<0.001** | **<0.001** |
| **Random Effects** | | | | | | | | | | | | |
| σ^2^ | 0.61 | | 0.59 | | 0.61 | | 0.57 | | | 0.57 | | |
| τ_00_ | 0.29 _id_ | | 0.30 _id_ | | 0.29 _id_ | | 0.31 _id_ | | | 0.31 _id_ | | |
|  | 0.01 _News_ | | 0.01 _News_ | | 0.01 _News_ | | 0.01 _News_ | | | 0.01 _News_ | | |
| ICC | 0.33 | | 0.34 | | 0.33 | | 0.36 | | | 0.36 | | |
| Marginal R^2^ / Conditional R^2^ | 0.116 / 0.406 | | 0.126 / 0.426 | | 0.116 / 0.407 | | 0.139 / 0.448 | | | 0.141 / 0.450 | | |

| **Experiment 10 fake political news - Gun control issue only** | | | | | | | | | | | | |
| --- | --- | --- | --- | --- | --- | --- | --- | --- | --- | --- | --- | --- |
|  | **sharing** | | **sharing** | | **sharing** | | **sharing** | | | **sharing** | | |
| *Predictors* | *ß* | *p* | *ß* | *p* | *ß* | *p* | *ß* | *p* | *std. p* | *ß* | *p* | *std. p* |
| Congruent [TRUE] | 0.70 (0.62,0.77) | **<0.001** | 0.52 (0.43,0.62) | **<0.001** | 0.70 (0.62,0.77) | **<0.001** | 0.70 (0.63,0.77) | 0.163 | **<0.001** | 0.64 (0.55,0.73) | 0.234 | **<0.001** |
| AbsImpIssue [TRUE] | 0.15 (0.05,0.24) | **0.003** | -0.08 (-0.20,0.04) | 0.205 |  |  |  |  |  | 0.05 (-0.09,0.18) | 0.495 | 0.495 |
| Condition [message myside sharing] | -0.15 (-0.24, -0.06) | **0.002** | -0.15 (-0.24, -0.06) | **0.002** | -0.15 (-0.24, -0.06) | **0.002** | -0.15 (-0.24, -0.06) | **0.002** | **0.002** | -0.15 (-0.24, -0.06) | **0.002** | **0.002** |
| edu | -0.09 (-0.14, -0.05) | **<0.001** | -0.09 (-0.14, -0.05) | **<0.001** | -0.09 (-0.14, -0.05) | **<0.001** | -0.09 (-0.14, -0.05) | **<0.001** | **<0.001** | -0.09 (-0.14, -0.05) | **<0.001** | **<0.001** |
| sex [female] | -0.13 (-0.22, -0.04) | **0.006** | -0.13 (-0.22, -0.04) | **0.006** | -0.14 (-0.23, -0.05) | **0.003** | -0.14 (-0.23, -0.05) | **0.003** | **0.003** | -0.13 (-0.23, -0.04) | **0.005** | **0.005** |
| age | -0.00 (-0.05,0.05) | 0.953 | -0.00 (-0.05,0.05) | 0.953 | -0.00 (-0.05,0.04) | 0.915 | -0.00 (-0.05,0.04) | 0.915 | 0.915 | -0.00 (-0.05,0.04) | 0.904 | 0.904 |
| Congruent [TRUE] * AbsImpIssue [TRUE] |  |  | 0.45 (0.30,0.60) | **<0.001** |  |  |  |  |  | 0.17 (0.01,0.32) | **0.037** | **0.037** |
| Attit Issue extr |  |  |  |  | 0.05 (0.00,0.10) | **0.039** | -0.13 (-0.19, -0.07) | **<0.001** | **<0.001** | -0.14 (-0.20, -0.08) | **<0.001** | **<0.001** |
| Congruent [TRUE] * Attit Issue extr |  |  |  |  |  |  | 0.36 (0.29,0.43) | **<0.001** | **<0.001** | 0.32 (0.25,0.40) | **<0.001** | **<0.001** |
| **Random Effects** | | | | | | | | | | | | |
| σ^2^ | 0.65 | | 0.63 | | 0.65 | | 0.59 | | | 0.58 | | |
| τ_00_ | 0.22 _id_ | | 0.23 _id_ | | 0.22 _id_ | | 0.25 _id_ | | | 0.25 _id_ | | |
|  | 0.01 _News_ | | 0.01 _News_ | | 0.01 _News_ | | 0.01 _News_ | | | 0.01 _News_ | | |
| ICC | 0.26 | | 0.27 | | 0.26 | | 0.31 | | | 0.31 | | |
| Marginal R^2^ / Conditional R^2^ | 0.143 / 0.363 | | 0.155 / 0.386 | | 0.140 / 0.363 | | 0.173 / 0.430 | | | 0.177 / 0.431 | | |

| **Experiment 10 true political news - Racial equality issue only** | | | | | | | | | | | | |
| --- | --- | --- | --- | --- | --- | --- | --- | --- | --- | --- | --- | --- |
|  | **sharing** | | **sharing** | | **sharing** | | **sharing** | | | **sharing** | | |
| *Predictors* | *ß* | *p* | *ß* | *p* | *ß* | *p* | *ß* | *p* | *std. p* | *ß* | *p* | *std. p* |
| Congruent [TRUE] | 0.49 (0.40,0.59) | **<0.001** | 0.35 (0.25,0.46) | **<0.001** | 0.49 (0.40,0.59) | **<0.001** | 0.54 (0.45,0.64) | **0.032** | **<0.001** | 0.43 (0.32,0.54) | 0.085 | **<0.001** |
| AbsImpIssue [TRUE] | 0.35 (0.25,0.44) | **<0.001** | 0.09 (-0.04,0.21) | 0.162 |  |  |  |  |  | 0.15 (0.02,0.28) | **0.026** | **0.026** |
| Condition [message myside sharing] | -0.23 (-0.32, -0.14) | **<0.001** | -0.23 (-0.32, -0.14) | **<0.001** | -0.24 (-0.33, -0.14) | **<0.001** | -0.24 (-0.33, -0.14) | **<0.001** | **<0.001** | -0.23 (-0.32, -0.14) | **<0.001** | **<0.001** |
| edu | -0.07 (-0.12, -0.02) | **0.003** | -0.07 (-0.12, -0.02) | **0.003** | -0.07 (-0.12, -0.03) | **0.003** | -0.07 (-0.12, -0.03) | **0.003** | **0.003** | -0.07 (-0.12, -0.02) | **0.003** | **0.003** |
| sex [female] | -0.13 (-0.23, -0.04) | **0.006** | -0.13 (-0.23, -0.04) | **0.006** | -0.15 (-0.25, -0.05) | **0.002** | -0.15 (-0.25, -0.05) | **0.002** | **0.002** | -0.14 (-0.23, -0.04) | **0.004** | **0.004** |
| age | -0.06 (-0.11, -0.01) | **0.010** | -0.06 (-0.11, -0.01) | **0.010** | -0.08 (-0.13, -0.03) | **0.001** | -0.08 (-0.13, -0.03) | **0.001** | **0.001** | -0.06 (-0.11, -0.01) | **0.010** | **0.010** |
| Congruent [TRUE] * AbsImpIssue [TRUE] |  |  | 0.52 (0.36,0.68) | **<0.001** |  |  |  |  |  | 0.36 (0.19,0.53) | **<0.001** | **<0.001** |
| Attit Issue extr |  |  |  |  | 0.08 (0.04,0.13) | **0.001** | -0.05 (-0.11,0.01) | 0.083 | 0.083 | -0.09 (-0.15, -0.02) | **0.007** | **0.007** |
| Congruent [TRUE] * Attit Issue extr |  |  |  |  |  |  | 0.28 (0.20,0.35) | **<0.001** | **<0.001** | 0.22 (0.14,0.30) | **<0.001** | **<0.001** |
| **Random Effects** | | | | | | | | | | | | |
| σ^2^ | 0.70 | | 0.68 | | 0.70 | | 0.67 | | | 0.66 | | |
| τ_00_ | 0.18 _id_ | | 0.19 _id_ | | 0.20 _id_ | | 0.22 _id_ | | | 0.20 _id_ | | |
|  | 0.01 _News_ | | 0.04 _News_ | | 0.01 _News_ | | 0.02 _News_ | | | 0.04 _News_ | | |
| ICC | 0.21 | | 0.25 | | 0.23 | | 0.27 | | | 0.27 | | |
| Marginal R^2^ / Conditional R^2^ | 0.121 / 0.306 | | 0.152 / 0.366 | | 0.099 / 0.307 | | 0.126 / 0.358 | | | 0.164 / 0.390 | | |

| **Experiment 10 fake political news - Racial equality issue only** | | | | | | | | | | | | |
| --- | --- | --- | --- | --- | --- | --- | --- | --- | --- | --- | --- | --- |
|  | **sharing** | | **sharing** | | **sharing** | | **sharing** | | | **sharing** | | |
| *Predictors* | *ß* | *p* | *ß* | *p* | *ß* | *p* | *ß* | *p* | *std. p* | *ß* | *p* | *std. p* |
| Congruent [TRUE] | 0.54 (0.46,0.63) | **<0.001** | 0.36 (0.27,0.44) | **<0.001** | 0.54 (0.46,0.63) | **<0.001** | 0.61 (0.54,0.67) | **0.004** | **<0.001** | 0.44 (0.35,0.53) | **0.012** | **<0.001** |
| AbsImpIssue [TRUE] | 0.26 (0.16,0.36) | **<0.001** | -0.02 (-0.14,0.09) | 0.680 |  |  |  |  |  | 0.03 (-0.10,0.16) | 0.617 | 0.617 |
| Condition [message myside sharing] | -0.25 (-0.35, -0.16) | **<0.001** | -0.25 (-0.35, -0.16) | **<0.001** | -0.25 (-0.35, -0.16) | **<0.001** | -0.25 (-0.35, -0.16) | **<0.001** | **<0.001** | -0.25 (-0.35, -0.16) | **<0.001** | **<0.001** |
| edu | -0.06 (-0.10, -0.01) | **0.025** | -0.06 (-0.10, -0.01) | **0.025** | -0.06 (-0.11, -0.01) | **0.021** | -0.06 (-0.11, -0.01) | **0.021** | **0.021** | -0.06 (-0.10, -0.01) | **0.023** | **0.023** |
| sex [female] | -0.06 (-0.15,0.04) | 0.260 | -0.06 (-0.15,0.04) | 0.260 | -0.08 (-0.18,0.02) | 0.126 | -0.08 (-0.18,0.02) | 0.126 | 0.126 | -0.07 (-0.16,0.03) | 0.183 | 0.183 |
| age | -0.04 (-0.09,0.01) | 0.114 | -0.04 (-0.09,0.01) | 0.114 | -0.05 (-0.10, -0.01) | **0.029** | -0.05 (-0.10, -0.01) | **0.029** | **0.029** | -0.04 (-0.09,0.01) | 0.107 | 0.107 |
| Congruent [TRUE] * AbsImpIssue [TRUE] |  |  | 0.57 (0.43,0.70) | **<0.001** |  |  |  |  |  | 0.39 (0.24,0.53) | **<0.001** | **<0.001** |
| Attit Issue extr |  |  |  |  | 0.08 (0.04,0.13) | **0.001** | -0.07 (-0.13, -0.01) | **0.015** | **0.015** | -0.08 (-0.14, -0.02) | **0.013** | **0.013** |
| Congruent [TRUE] * Attit Issue extr |  |  |  |  |  |  | 0.31 (0.25,0.38) | **<0.001** | **<0.001** | 0.24 (0.17,0.31) | **<0.001** | **<0.001** |
| **Random Effects** | | | | | | | | | | | | |
| σ^2^ | 0.49 | | 0.46 | | 0.49 | | 0.46 | | | 0.44 | | |
| τ_00_ | 0.24 _id_ | | 0.25 _id_ | | 0.25 _id_ | | 0.27 _id_ | | | 0.26 _id_ | | |
|  | 0.00 _News_ | | 0.00 _News_ | | 0.00 _News_ | | 0.00 _News_ | | | 0.00 _News_ | | |
| ICC | 0.33 | |  | | 0.34 | |  | | | 0.37 | | |
| Marginal R^2^ / Conditional R^2^ | 0.117 / 0.409 | | 0.218 / NA | | 0.108 / 0.409 | | 0.216 / NA | | | 0.169 / 0.479 | | |

| **Experiment 10 true political news - Gender equality issue only** | | | | | | | | | | | | | | | | | |
| --- | --- | --- | --- | --- | --- | --- | --- | --- | --- | --- | --- | --- | --- | --- | --- | --- | --- |
|  | **sharing** | | | **sharing** | | | **sharing** | | | | **sharing** | | | | **sharing** | | |
| *Predictors* | *ß* | *p* | *std. p* | *ß* | *p* | *std. p* | *ß* | *p* | *std. p* | *ß* | | *p* | *std. p* | *ß* | | *p* | *std. p* |
| Congruent [TRUE] | 0.25 (0.17,0.33) | **<0.001** | **<0.001** | 0.14 (0.04,0.23) | **<0.001** | **0.005** | 0.35 (0.27,0.44) | **<0.001** | **<0.001** | 0.38 (0.29,0.46) | | 0.196 | **<0.001** | 0.18 (0.08,0.27) | | 0.427 | **<0.001** |
| AbsImpIssue [TRUE] | 0.21 (0.11,0.31) | **<0.001** | **<0.001** | 0.05 (-0.08,0.18) | 0.533 | 0.467 |  |  |  |  | |  |  | 0.12 (-0.01,0.26) | | 0.617 | 0.078 |
| Condition [message myside sharing] | -0.25 (-0.34, -0.15) | **<0.001** | **<0.001** | -0.25 (-0.34, -0.15) | **<0.001** | **<0.001** | -0.25 (-0.35, -0.15) | **<0.001** | **<0.001** | -0.25 (-0.35, -0.15) | | **<0.001** | **<0.001** | -0.25 (-0.34, -0.15) | | **<0.001** | **<0.001** |
| edu | -0.04 (-0.08,0.01) | 0.172 | 0.144 | -0.04 (-0.08,0.01) | 0.172 | 0.146 | -0.03 (-0.08,0.02) | 0.260 | 0.260 | -0.03 (-0.08,0.02) | | 0.260 | 0.260 | -0.04 (-0.08,0.01) | | 0.166 | 0.143 |
| sex [female] | -0.14 (-0.24, -0.05) | **0.006** | **0.003** | -0.14 (-0.24, -0.05) | **0.006** | **0.004** | -0.12 (-0.22, -0.02) | **0.024** | **0.024** | -0.12 (-0.22, -0.02) | | **0.024** | **0.024** | -0.14 (-0.23, -0.04) | | **0.008** | **0.005** |
| age | -0.08 (-0.12, -0.03) | **0.003** | **0.002** | -0.08 (-0.12, -0.03) | **0.003** | **0.002** | -0.09 (-0.14, -0.04) | **<0.001** | **<0.001** | -0.09 (-0.14, -0.04) | | **<0.001** | **<0.001** | -0.08 (-0.12, -0.03) | | **0.003** | **0.002** |
| Congruent [TRUE] * AbsImpIssue [TRUE] |  |  |  | 0.32 (0.16,0.48) | **<0.001** | **<0.001** |  |  |  |  | |  |  | 0.21 (0.04,0.38) | | **<0.001** | **0.014** |
| Attit Issue extr |  |  |  |  |  |  | 0.01 (-0.05,0.06) | 0.821 | 0.821 | -0.10 (-0.16, -0.03) | | **0.003** | **0.003** | -0.10 (-0.16, -0.03) | | **0.002** | **0.003** |
| Congruent [TRUE] * Attit Issue extr |  |  |  |  |  |  |  |  |  | 0.20 (0.13,0.27) | | **<0.001** | **<0.001** | 0.13 (0.05,0.21) | | **<0.001** | **0.001** |
| **Random Effects** | | | | | | | | | | | | | | | | | |
| σ^2^ | 0.51 | | | 0.49 | | | 0.51 | | | | 0.49 | | | | 0.48 | | |
| τ_00_ | 0.25 _id_ | | | 0.26 _id_ | | | 0.26 _id_ | | | | 0.26 _id_ | | | | 0.26 _id_ | | |
|  | 0.02 _News_ | | | 0.04 _News_ | | | 0.01 _News_ | | | | 0.01 _News_ | | | | 0.04 _News_ | | |
| ICC | 0.34 | | | 0.38 | | | 0.35 | | | | 0.36 | | | | 0.38 | | |
| Marginal R^2^ / Conditional R^2^ | 0.069 / 0.387 | | | 0.090 / 0.432 | | | 0.060 / 0.387 | | | | 0.073 / 0.408 | | | | 0.096 / 0.442 | | |

| **Experiment 10 fake political news - Gender equality issue only** | | | | | | | | | | | | |
| --- | --- | --- | --- | --- | --- | --- | --- | --- | --- | --- | --- | --- |
|  | **sharing** | | **sharing** | | **sharing** | | **sharing** | | | **sharing** | | |
| *Predictors* | *ß* | *p* | *ß* | *p* | *ß* | *p* | *ß* | *p* | *std. p* | *ß* | *p* | *std. p* |
| Congruent [TRUE] | 0.36 (0.29,0.44) | **<0.001** | 0.30 (0.20,0.39) | **<0.001** | 0.36 (0.29,0.44) | **<0.001** | 0.40 (0.31,0.48) | 0.200 | **<0.001** | 0.35 (0.26,0.45) | 0.284 | **<0.001** |
| AbsImpIssue [TRUE] | 0.36 (0.25,0.46) | **<0.001** | 0.20 (0.07,0.33) | **0.003** |  |  |  |  |  | 0.25 (0.12,0.39) | **<0.001** | **<0.001** |
| Condition [message myside sharing] | -0.25 (-0.35, -0.16) | **<0.001** | -0.25 (-0.35, -0.16) | **<0.001** | -0.25 (-0.35, -0.15) | **<0.001** | -0.25 (-0.35, -0.15) | **<0.001** | **<0.001** | -0.25 (-0.35, -0.16) | **<0.001** | **<0.001** |
| edu | -0.09 (-0.14, -0.04) | **<0.001** | -0.09 (-0.14, -0.04) | **<0.001** | -0.08 (-0.13, -0.03) | **0.001** | -0.08 (-0.13, -0.03) | **0.001** | **0.001** | -0.09 (-0.14, -0.04) | **<0.001** | **<0.001** |
| sex [female] | 0.06 (-0.04,0.16) | 0.245 | 0.06 (-0.04,0.16) | 0.245 | 0.08 (-0.02,0.18) | 0.099 | 0.08 (-0.02,0.18) | 0.099 | 0.099 | 0.06 (-0.04,0.15) | 0.264 | 0.264 |
| age | -0.08 (-0.13, -0.03) | **0.002** | -0.08 (-0.13, -0.03) | **0.002** | -0.10 (-0.15, -0.05) | **<0.001** | -0.10 (-0.15, -0.05) | **<0.001** | **<0.001** | -0.08 (-0.13, -0.03) | **0.002** | **0.002** |
| Congruent [TRUE] * AbsImpIssue [TRUE] |  |  | 0.31 (0.16,0.47) | **<0.001** |  |  |  |  |  | 0.18 (0.01,0.34) | **0.036** | **0.036** |
| Attit Issue extr |  |  |  |  | 0.07 (0.03,0.12) | **0.003** | -0.03 (-0.09,0.03) | 0.330 | 0.330 | -0.08 (-0.14, -0.01) | **0.022** | **0.022** |
| Congruent [TRUE] * Attit Issue extr |  |  |  |  |  |  | 0.21 (0.14,0.28) | **<0.001** | **<0.001** | 0.19 (0.11,0.26) | **<0.001** | **<0.001** |
| **Random Effects** | | | | | | | | | | | | |
| σ^2^ | 0.57 | | 0.56 | | 0.57 | | 0.55 | | | 0.55 | | |
| τ_00_ | 0.23 _id_ | | 0.24 _id_ | | 0.25 _id_ | | 0.26 _id_ | | | 0.24 _id_ | | |
|  | 0.00 _News_ | | 0.00 _News_ | | 0.00 _News_ | | 0.00 _News_ | | | 0.01 _News_ | | |
| ICC | 0.29 | | 0.30 | | 0.31 | | 0.33 | | | 0.31 | | |
| Marginal R^2^ / Conditional R^2^ | 0.100 / 0.363 | | 0.113 / 0.382 | | 0.077 / 0.363 | | 0.094 / 0.390 | | | 0.122 / 0.398 | | |

| **Experiment 10 true political news - Immigration issue only** | | | | | | | | | | | | |
| --- | --- | --- | --- | --- | --- | --- | --- | --- | --- | --- | --- | --- |
|  | **sharing** | | **sharing** | | **sharing** | | **sharing** | | | **sharing** | | |
| *Predictors* | *ß* | *p* | *ß* | *p* | *ß* | *p* | *ß* | *p* | *std. p* | *ß* | *p* | *std. p* |
| Congruent [TRUE] | 0.54 (0.46,0.61) | **<0.001** | 0.42 (0.34,0.51) | **<0.001** | 0.54 (0.46,0.61) | **<0.001** | 0.54 (0.46,0.61) | 0.565 | **<0.001** | 0.47 (0.39,0.56) | 0.571 | **<0.001** |
| AbsImpIssue [TRUE] | 0.26 (0.14,0.38) | **<0.001** | 0.02 (-0.13,0.16) | 0.832 |  |  |  |  |  | 0.08 (-0.08,0.24) | 0.314 | 0.314 |
| Condition [message myside sharing] | -0.17 (-0.27, -0.07) | **0.001** | -0.17 (-0.27, -0.07) | **0.001** | -0.16 (-0.26, -0.06) | **0.001** | -0.16 (-0.26, -0.06) | **0.001** | **0.001** | -0.17 (-0.26, -0.07) | **0.001** | **0.001** |
| edu | -0.07 (-0.11, -0.02) | **0.009** | -0.07 (-0.11, -0.02) | **0.009** | -0.06 (-0.11, -0.01) | **0.016** | -0.06 (-0.11, -0.01) | **0.016** | **0.016** | -0.06 (-0.11, -0.02) | **0.009** | **0.009** |
| sex [female] | -0.20 (-0.30, -0.11) | **<0.001** | -0.20 (-0.30, -0.11) | **<0.001** | -0.20 (-0.30, -0.11) | **<0.001** | -0.20 (-0.30, -0.11) | **<0.001** | **<0.001** | -0.20 (-0.30, -0.11) | **<0.001** | **<0.001** |
| age | -0.04 (-0.09,0.01) | 0.101 | -0.04 (-0.09,0.01) | 0.101 | -0.03 (-0.08,0.02) | 0.248 | -0.03 (-0.08,0.02) | 0.248 | 0.248 | -0.04 (-0.09,0.01) | 0.126 | 0.126 |
| Congruent [TRUE] * AbsImpIssue [TRUE] |  |  | 0.48 (0.30,0.66) | **<0.001** |  |  |  |  |  | 0.28 (0.09,0.46) | **0.003** | **0.003** |
| Attit Issue extr |  |  |  |  | 0.08 (0.03,0.13) | **0.002** | -0.06 (-0.12, -0.01) | **0.033** | **0.033** | -0.08 (-0.14, -0.01) | **0.016** | **0.016** |
| Congruent [TRUE] * Attit Issue extr |  |  |  |  |  |  | 0.29 (0.22,0.36) | **<0.001** | **<0.001** | 0.25 (0.17,0.32) | **<0.001** | **<0.001** |
| **Random Effects** | | | | | | | | | | | | |
| σ^2^ | 0.59 | | 0.57 | | 0.59 | | 0.55 | | | 0.55 | | |
| τ_00_ | 0.23 _id_ | | 0.24 _id_ | | 0.23 _id_ | | 0.25 _id_ | | | 0.25 _id_ | | |
|  | 0.00 _News_ | | 0.00 _News_ | | 0.00 _News_ | | 0.00 _News_ | | | 0.00 _News_ | | |
| ICC | 0.28 | | 0.29 | | 0.29 | | 0.31 | | | 0.31 | | |
| Marginal R^2^ / Conditional R^2^ | 0.104 / 0.356 | | 0.111 / 0.371 | | 0.099 / 0.357 | | 0.119 / 0.396 | | | 0.127 / 0.399 | | |

| **Experiment 10 fake political news - Immigration issue only** | | | | | | | | | | | | |
| --- | --- | --- | --- | --- | --- | --- | --- | --- | --- | --- | --- | --- |
|  | **sharing** | | **sharing** | | **sharing** | | **sharing** | | | **sharing** | | |
| *Predictors* | *ß* | *p* | *ß* | *p* | *ß* | *p* | *ß* | *p* | *std. p* | *ß* | *p* | *std. p* |
| Congruent [TRUE] | 0.55 (0.47,0.63) | **<0.001** | 0.49 (0.40,0.58) | **<0.001** | 0.55 (0.47,0.63) | **<0.001** | 0.55 (0.47,0.63) | 0.907 | **<0.001** | 0.55 (0.46,0.64) | 0.896 | **<0.001** |
| AbsImpIssue [TRUE] | 0.18 (0.06,0.29) | **0.003** | 0.04 (-0.11,0.19) | 0.588 |  |  |  |  |  | 0.18 (0.02,0.34) | **0.025** | **0.025** |
| Condition [message myside sharing] | -0.20 (-0.30, -0.11) | **<0.001** | -0.20 (-0.30, -0.11) | **<0.001** | -0.20 (-0.29, -0.10) | **<0.001** | -0.20 (-0.29, -0.10) | **<0.001** | **<0.001** | -0.20 (-0.30, -0.11) | **<0.001** | **<0.001** |
| edu | -0.08 (-0.13, -0.03) | **0.001** | -0.08 (-0.13, -0.03) | **0.001** | -0.07 (-0.12, -0.03) | **0.002** | -0.07 (-0.12, -0.03) | **0.002** | **0.002** | -0.08 (-0.13, -0.03) | **0.001** | **0.001** |
| sex [female] | -0.15 (-0.24, -0.05) | **0.003** | -0.15 (-0.24, -0.05) | **0.003** | -0.15 (-0.24, -0.05) | **0.003** | -0.15 (-0.24, -0.05) | **0.003** | **0.003** | -0.15 (-0.24, -0.05) | **0.003** | **0.003** |
| age | -0.02 (-0.06,0.03) | 0.490 | -0.02 (-0.06,0.03) | 0.490 | -0.01 (-0.06,0.04) | 0.697 | -0.01 (-0.06,0.04) | 0.697 | 0.697 | -0.02 (-0.07,0.03) | 0.482 | 0.482 |
| Congruent [TRUE] * AbsImpIssue [TRUE] |  |  | 0.27 (0.08,0.46) | **0.005** |  |  |  |  |  | 0.00 (-0.19,0.20) | 0.970 | 0.970 |
| Attit Issue extr |  |  |  |  | 0.02 (-0.03,0.07) | 0.400 | -0.14 (-0.20, -0.08) | **<0.001** | **<0.001** | -0.17 (-0.23, -0.10) | **<0.001** | **<0.001** |
| Congruent [TRUE] * Attit Issue extr |  |  |  |  |  |  | 0.32 (0.25,0.40) | **<0.001** | **<0.001** | 0.32 (0.24,0.40) | **<0.001** | **<0.001** |
| **Random Effects** | | | | | | | | | | | | |
| σ^2^ | 0.66 | | 0.66 | | 0.66 | | 0.61 | | | 0.61 | | |
| τ_00_ | 0.18 _id_ | | 0.18 _id_ | | 0.18 _id_ | | 0.21 _id_ | | | 0.20 _id_ | | |
|  | 0.02 _News_ | | 0.02 _News_ | | 0.02 _News_ | | 0.02 _News_ | | | 0.02 _News_ | | |
| ICC | 0.24 | | 0.24 | | 0.24 | | 0.27 | | | 0.27 | | |
| Marginal R^2^ / Conditional R^2^ | 0.098 / 0.311 | | 0.100 / 0.312 | | 0.094 / 0.311 | | 0.119 / 0.360 | | | 0.123 / 0.360 | | |

### Congruence x Condition

| **Experiment 10 true political news** | | |
| --- | --- | --- |
|  | **sharing** | |
| *Predictors* | *ß* | *p* |
| Congruent [TRUE] | 0.49 (0.43,0.54) | **<0.001** |
| Condition [message myside sharing] | -0.16 (-0.24, -0.09) | **<0.001** |
| edu | -0.06 (-0.10, -0.03) | **0.001** |
| sex [female] | -0.13 (-0.20, -0.05) | **0.001** |
| age | -0.05 (-0.09, -0.01) | **0.012** |
| Congruent [TRUE] * Condition [message myside sharing] | -0.02 (-0.10,0.06) | 0.625 |
| **Random Effects** | | |
| σ^2^ | 0.50 | |
| τ_00_ _id_ | 0.26 | |
| τ_00_ _News_ | 0.04 | |
| τ_11_ _id.CongruentTRUE_ | 0.20 | |
| ρ_01_ _id_ | 0.21 | |
| ICC | 0.47 | |
| Marginal R^2^ / Conditional R^2^ | 0.074 / 0.513 | |

| **Experiment 10 fake political news** | | |
| --- | --- | --- |
|  | **sharing** | |
| *Predictors* | *ß* | *p* |
| Congruent [TRUE] | 0.56 (0.50,0.62) | **<0.001** |
| Condition [message myside sharing] | -0.19 (-0.27, -0.12) | **<0.001** |
| edu | -0.07 (-0.11, -0.04) | **<0.001** |
| sex [female] | -0.06 (-0.14,0.01) | 0.101 |
| age | -0.03 (-0.07,0.01) | 0.114 |
| Congruent [TRUE] * Condition [message myside sharing] | -0.01 (-0.10,0.08) | 0.820 |
| **Random Effects** | | |
| σ^2^ | 0.45 | |
| τ_00_ _id_ | 0.24 | |
| τ_00_ _News_ | 0.02 | |
| τ_11_ _id.CongruentTRUE_ | 0.26 | |
| ρ_01_ _id_ | 0.08 | |
| ICC | 0.47 | |
| Marginal R^2^ / Conditional R^2^ | 0.093 / 0.519 | |

### Congruence x Condition on 8 first items viewed only

| **Experiment 10 true political news - 8 first items only** | | |
| --- | --- | --- |
|  | **SHARING** | |
| *Predictors* | *ß* | *p* |
| Congruent [TRUE] | 0.51 (0.43,0.59) | **<0.001** |
| Condition [message myside sharing] | -0.18 (-0.27, -0.08) | **<0.001** |
| edu | -0.06 (-0.11, -0.02) | **0.005** |
| sex [female] | -0.05 (-0.14,0.04) | 0.279 |
| age | -0.07 (-0.11, -0.02) | **0.003** |
| Congruent [TRUE] * Condition [message myside sharing] | -0.02 (-0.14,0.09) | 0.715 |
| **Random Effects** | | |
| σ^2^ | 0.47 | |
| τ_00_ _id_ | 0.25 | |
| τ_00_ _News_ | 0.05 | |
| τ_11_ _id.CongruentTRUE_ | 0.13 | |
| ρ_01_ _id_ | 0.43 | |
| ICC | 0.48 | |
| Marginal R^2^ / Conditional R^2^ | 0.079 / 0.519 | |

| **Experiment 10 fake political news - 8 first items only** | | |
| --- | --- | --- |
|  | **SHARING** | |
| *Predictors* | *ß* | *p* |
| Congruent [TRUE] | 0.55 (0.47,0.64) | **<0.001** |
| Condition [message myside sharing] | -0.18 (-0.27, -0.09) | **<0.001** |
| edu | -0.09 (-0.13, -0.05) | **<0.001** |
| sex [female] | -0.08 (-0.16,0.01) | 0.083 |
| age | -0.02 (-0.06,0.02) | 0.338 |
| Congruent [TRUE] * Condition [message myside sharing] | -0.03 (-0.15,0.09) | 0.634 |
| **Random Effects** | | |
| σ^2^ | 0.43 | |
| τ_00_ _id_ | 0.20 | |
| τ_00_ _News_ | 0.02 | |
| τ_11_ _id.CongruentTRUE_ | 0.27 | |
| ρ_01_ _id_ | 0.19 | |
| ICC | 0.48 | |
| Marginal R^2^ / Conditional R^2^ | 0.090 / 0.525 | |

### Condition on true neutral items

| **Experiment 10 true neutral news** | | |
| --- | --- | --- |
|  | **sharing** | |
| *Predictors* | *ß* | *p* |
| Condition [message myside sharing] | -0.43 (-0.54, -0.33) | **<0.001** |
| edu | -0.07 (-0.13, -0.02) | **0.006** |
| sex [female] | -0.23 (-0.33, -0.12) | **<0.001** |
| age | -0.05 (-0.10,0.00) | 0.069 |
| **Random Effects** | | |
| σ^2^ | 0.48 | |
| τ_00_ _id_ | 0.47 | |
| τ_00_ _News_ | 0.04 | |
| ICC | 0.51 | |
| Marginal R^2^ / Conditional R^2^ | 0.068 / 0.547 | |

### Effects of partisanship

| **Experiment 10 - true political news** | | | | | | | | | | | | | | |
| --- | --- | --- | --- | --- | --- | --- | --- | --- | --- | --- | --- | --- | --- | --- |
|  | **sharing** | | **sharing** | | | **sharing** | | **sharing** | | | **sharing** | | **sharing** | |
| *Predictors* | *ß* | *p* | *ß* | *p* | *std. p* | *ß* | *p* | *ß* | *p* | *std. p* | *ß* | *p* | *ß* | *p* |
| Condition [message myside sharing] | -0.17 (-0.25, -0.09) | **<0.001** | -0.22 (-0.34, -0.11) | **<0.001** | **<0.001** |  |  |  |  |  | -0.16 (-0.24, -0.09) | **<0.001** | -0.19 (-0.31, -0.06) | **0.003** |
| edu | -0.06 (-0.10, -0.02) | **0.004** | -0.07 (-0.11, -0.03) | **0.001** | **0.001** | -0.06 (-0.10, -0.02) | **0.003** | -0.07 (-0.11, -0.03) | **0.001** | **0.001** | -0.06 (-0.10, -0.03) | **0.001** | -0.07 (-0.11, -0.03) | **0.001** |
| sex [female] | -0.12 (-0.20, -0.03) | **0.006** | -0.13 (-0.21, -0.05) | **0.002** | **0.002** | -0.11 (-0.19, -0.03) | **0.008** | -0.13 (-0.21, -0.05) | **0.002** | **0.002** | -0.13 (-0.21, -0.05) | **0.001** | -0.13 (-0.21, -0.05) | **0.002** |
| age | -0.06 (-0.10, -0.02) | **0.006** | -0.03 (-0.07,0.01) | 0.143 | 0.143 | -0.06 (-0.10, -0.02) | **0.004** | -0.03 (-0.07,0.01) | 0.122 | 0.122 | -0.05 (-0.09, -0.01) | **0.012** | -0.03 (-0.07,0.01) | 0.143 |
| PO bin [Republican] |  |  | -0.33 (-0.44, -0.21) | **<0.001** | **<0.001** |  |  | -0.03 (-0.12,0.06) | 0.504 | 0.504 |  |  | -0.05 (-0.17,0.07) | 0.390 |
| PO bin [Independent] |  |  | -0.06 (-0.63,0.51) | 0.831 | 0.831 |  |  | -0.38 (-0.76,0.00) | 0.052 | 0.052 |  |  | 0.20 (-0.42,0.81) | 0.531 |
| Condition [message myside sharing] * PO bin [Republican] |  |  | 0.11 (-0.05,0.27) | 0.179 | 0.179 |  |  |  |  |  |  |  | 0.06 (-0.11,0.23) | 0.506 |
| Condition [message myside sharing] * PO bin [Independent] |  |  | -0.61 (-1.28,0.07) | 0.080 | 0.081 |  |  |  |  |  |  |  | -0.58 (-1.33,0.16) | 0.126 |
| Congruent [TRUE] |  |  |  |  |  | 0.45 (0.42,0.49) | **<0.001** | 0.75 (0.70,0.81) | **<0.001** | **<0.001** | 0.49 (0.43,0.54) | **<0.001** | 0.79 (0.72,0.85) | **<0.001** |
| Congruent [TRUE] * PO bin [Republican] |  |  |  |  |  |  |  | -0.50 (-0.57, -0.43) | **<0.001** | **<0.001** |  |  | -0.55 (-0.64, -0.46) | **<0.001** |
| Congruent [TRUE] * PO bin [Independent] |  |  |  |  |  |  |  | -0.55 (-0.86, -0.24) | **0.001** | **0.001** |  |  | -0.52 (-0.98, -0.05) | **0.029** |
| Congruent [TRUE] * Condition [message myside sharing] |  |  |  |  |  |  |  |  |  |  | -0.02 (-0.10,0.06) | 0.625 | -0.07 (-0.16,0.01) | 0.085 |
| (Congruent [TRUE] * Condition [message myside sharing]) * PO bin [Republican] |  |  |  |  |  |  |  |  |  |  |  |  | 0.10 (-0.02,0.22) | 0.091 |
| (Congruent [TRUE] * Condition [message myside sharing]) * PO bin [Independent] |  |  |  |  |  |  |  |  |  |  |  |  | -0.04 (-0.67,0.58) | 0.888 |
| **Random Effects** | | | | | | | | | | | | | | |
| σ^2^ | 0.60 | | 0.60 | | | 0.56 | | 0.54 | | | 0.50 | | 0.54 | |
| τ_00_ | 0.38 _id_ | | 0.36 _id_ | | | 0.39 _id_ | | 0.38 _id_ | | | 0.30 _id_ | | 0.37 _id_ | |
|  | 0.03 _News_ | | 0.03 _News_ | | | 0.04 _News_ | | 0.05 _News_ | | | 0.04 _News_ | | 0.05 _News_ | |
| τ_11_ | 0.67 _id.Conditionmessage myside sharing_ | | 0.85 _id.PO_binRepublican_ | | | 0.84 _id.Conditionmessage myside sharing_ | | 0.88 _id.PO_binRepublican_ | | | 0.20 _id.CongruentTRUE_ | | 0.66 _id.PO_binRepublican_ | |
|  |  | | 0.59 _id.PO_binIndependent_ | | |  | | 0.61 _id.PO_binIndependent_ | | | 0.79 _id.Conditionmessage myside sharing_ | | 0.33 _id.PO_binIndependent_ | |
|  |  | | 0.06 _id.Conditionmessage myside sharing_ | | |  | | 0.26 _id.Conditionmessage myside sharing_ | | |  | | 0.10 _id.Conditionmessage myside sharing_ | |
| ρ_01_ | -0.73 _id_ | | -0.78 _id.PO_binRepublican_ | | | -0.79 _id_ | | -0.78 _id.PO_binRepublican_ | | | 0.14 _id.CongruentTRUE_ | | -0.69 _id.PO_binRepublican_ | |
|  |  | | -0.74 _id.PO_binIndependent_ | | |  | | -0.30 _id.PO_binIndependent_ | | | -0.90 _id.Conditionmessage myside sharing_ | | -0.61 _id.PO_binIndependent_ | |
|  |  | | -0.25 _id.Conditionmessage myside sharing_ | | |  | | -0.44 _id.Conditionmessage myside sharing_ | | |  | | -0.30 _id.Conditionmessage myside sharing_ | |
| ICC | 0.39 | | 0.37 | | | 0.43 | | 0.44 | | | 0.47 | | 0.42 | |
| Marginal R^2^ / Conditional R^2^ | 0.019 / 0.397 | | 0.040 / 0.398 | | | 0.060 / 0.467 | | 0.104 / 0.496 | | | 0.074 / 0.513 | | 0.114 / 0.484 | |

| **Experiment 10 - fake political news** | | | | | | | | | | | | | | | |
| --- | --- | --- | --- | --- | --- | --- | --- | --- | --- | --- | --- | --- | --- | --- | --- |
|  | **sharing** | | **sharing** | | | **sharing** | | **sharing** | | | **sharing** | | **sharing** | | |
| *Predictors* | *ß* | *p* | *ß* | *p* | *std. p* | *ß* | *p* | *ß* | *p* | *std. p* | *ß* | *p* | *ß* | *p* | *std. p* |
| Condition [message myside sharing] | -0.20 (-0.27, -0.12) | **<0.001** | -0.28 (-0.39, -0.17) | **<0.001** | **<0.001** |  |  |  |  |  | -0.19 (-0.27, -0.12) | **<0.001** | -0.24 (-0.36, -0.12) | **<0.001** | **<0.001** |
| edu | -0.08 (-0.12, -0.04) | **<0.001** | -0.08 (-0.12, -0.04) | **<0.001** | **<0.001** | -0.08 (-0.12, -0.04) | **<0.001** | -0.08 (-0.12, -0.04) | **<0.001** | **<0.001** | -0.07 (-0.11, -0.04) | **<0.001** | -0.08 (-0.12, -0.04) | **<0.001** | **<0.001** |
| sex [female] | -0.06 (-0.13,0.02) | 0.176 | -0.06 (-0.14,0.02) | 0.163 | 0.163 | -0.05 (-0.13,0.03) | 0.216 | -0.05 (-0.14,0.03) | 0.182 | 0.182 | -0.07 (-0.14,0.01) | 0.076 | -0.06 (-0.14,0.02) | 0.163 | 0.163 |
| age | -0.05 (-0.09, -0.01) | **0.015** | -0.04 (-0.08, -0.00) | **0.039** | **0.039** | -0.05 (-0.09, -0.01) | **0.011** | -0.05 (-0.09, -0.00) | **0.032** | **0.032** | -0.03 (-0.07,0.01) | 0.117 | -0.04 (-0.08, -0.00) | **0.039** | **0.039** |
| PO bin [Republican] |  |  | -0.18 (-0.30, -0.07) | **0.002** | **0.002** |  |  | 0.20 (0.11,0.29) | **<0.001** | **<0.001** |  |  | 0.13 (0.01,0.26) | **0.031** | **0.031** |
| PO bin [Independent] |  |  | 0.16 (-0.60,0.91) | 0.681 | 0.681 |  |  | -0.25 (-0.60,0.11) | 0.168 | 0.168 |  |  | 0.36 (-0.43,1.15) | 0.370 | 0.370 |
| Condition [message myside sharing] * PO bin [Republican] |  |  | 0.19 (0.03,0.35) | **0.022** | **0.022** |  |  |  |  |  |  |  | 0.11 (-0.06,0.28) | 0.198 | 0.198 |
| Condition [message myside sharing] * PO bin [Independent] |  |  | -0.64 (-1.46,0.19) | 0.131 | 0.130 |  |  |  |  |  |  |  | -0.50 (-1.38,0.38) | 0.270 | 0.269 |
| Congruent [TRUE] |  |  |  |  |  | 0.53 (0.50,0.56) | **<0.001** | 0.87 (0.82,0.93) | **<0.001** | **<0.001** | 0.56 (0.50,0.62) | **<0.001** | 0.91 (0.85,0.98) | **<0.001** | **<0.001** |
| Congruent [TRUE] * PO bin [Republican] |  |  |  |  |  |  |  | -0.57 (-0.64, -0.50) | **<0.001** | **<0.001** |  |  | -0.64 (-0.73, -0.55) | **<0.001** | **<0.001** |
| Congruent [TRUE] * PO bin [Independent] |  |  |  |  |  |  |  | -0.57 (-0.88, -0.26) | **<0.001** | **<0.001** |  |  | -0.41 (-0.87,0.06) | 0.087 | 0.087 |
| Congruent [TRUE] * Condition [message myside sharing] |  |  |  |  |  |  |  |  |  |  | -0.01 (-0.10,0.08) | 0.821 | -0.08 (-0.17, -0.00) | **0.048** | **0.048** |
| (Congruent [TRUE] * Condition [message myside sharing]) * PO bin [Republican] |  |  |  |  |  |  |  |  |  |  |  |  | 0.15 (0.03,0.27) | **0.015** | **0.015** |
| (Congruent [TRUE] * Condition [message myside sharing]) * PO bin [Independent] |  |  |  |  |  |  |  |  |  |  |  |  | -0.28 (-0.90,0.35) | 0.381 | 0.381 |
| **Random Effects** | | | | | | | | | | | | | | | |
| σ^2^ | 0.58 | | 0.58 | | | 0.52 | | 0.51 | | | 0.45 | | 0.51 | | |
| τ_00_ | 0.35 _id_ | | 0.34 _id_ | | | 0.36 _id_ | | 0.37 _id_ | | | 0.29 _id_ | | 0.35 _id_ | | |
|  | 0.02 _News_ | | 0.02 _News_ | | | 0.01 _News_ | | 0.03 _News_ | | | 0.02 _News_ | | 0.03 _News_ | | |
| τ_11_ | 1.08 _id.Conditionmessage myside sharing_ | | 0.00 _id.PO_binRepublican_ | | | 0.27 _id.Conditionmessage myside sharing_ | | 0.01 _id.PO_binRepublican_ | | | 0.26 _id.CongruentTRUE_ | | 0.24 _id.PO_binRepublican_ | | |
|  |  | | 0.17 _id.PO_binIndependent_ | | |  | | 0.25 _id.PO_binIndependent_ | | | 0.81 _id.Conditionmessage myside sharing_ | | 0.20 _id.PO_binIndependent_ | | |
|  |  | | 0.81 _id.Conditionmessage myside sharing_ | | |  | | 1.15 _id.Conditionmessage myside sharing_ | | |  | | 0.50 _id.Conditionmessage myside sharing_ | | |
| ρ_01_ | -0.95 _id_ | | -0.12 _id.PO_binRepublican_ | | | -0.57 _id_ | | -0.26 _id.PO_binRepublican_ | | | 0.01 _id.CongruentTRUE_ | | -0.43 _id.PO_binRepublican_ | | |
|  |  | | -0.06 _id.PO_binIndependent_ | | |  | | 0.65 _id.PO_binIndependent_ | | | -0.96 _id.Conditionmessage myside sharing_ | | -0.11 _id.PO_binIndependent_ | | |
|  |  | | -0.85 _id.Conditionmessage myside sharing_ | | |  | | -0.95 _id.Conditionmessage myside sharing_ | | |  | | -0.70 _id.Conditionmessage myside sharing_ | | |
| ICC | 0.36 | | 0.35 | | | 0.41 | | 0.43 | | | 0.47 | | 0.40 | | |
| Marginal R^2^ / Conditional R^2^ | 0.020 / 0.369 | | 0.026 / 0.370 | | | 0.077 / 0.459 | | 0.111 / 0.496 | | | 0.092 / 0.519 | | 0.128 / 0.476 | | |

### Congruence x Condition on Democrats only (restricted analyses)

| **Experiment 10 true political news, Democrats only** | | | | |
| --- | --- | --- | --- | --- |
|  | **sharing** | | **sharing** | |
| *Predictors* | *ß* | *p* | *ß* | *p* |
| Condition [message myside sharing] | -0.19 (-0.29, -0.09) | **<0.001** | -0.19 (-0.29, -0.09) | **<0.001** |
| edu | -0.03 (-0.08,0.02) | 0.240 | -0.03 (-0.08,0.02) | 0.204 |
| sex [female] | -0.12 (-0.22, -0.02) | **0.019** | -0.13 (-0.23, -0.03) | **0.012** |
| age | -0.08 (-0.13, -0.03) | **0.002** | -0.08 (-0.13, -0.03) | **0.002** |
| Congruent [TRUE] |  |  | 0.36 (0.26,0.46) | **<0.001** |
| Congruent [TRUE] * Condition [message myside sharing] |  |  | -0.06 (-0.16,0.04) | 0.260 |
| **Random Effects** | | | | |
| σ^2^ | 0.48 | | 0.47 | |
| τ_00_ | 0.24 _id_ | | 0.24 _id_ | |
|  | 0.13 _News_ | | 0.09 _News_ | |
| τ_11_ | 0.16 _id.CongruentTRUE_ | | 0.16 _id.CongruentTRUE_ | |
| ρ_01_ | 0.48 _id_ | | 0.46 _id_ | |
| ICC | 0.44 | | 0.51 | |
| Marginal R^2^ / Conditional R^2^ | 0.027 / 0.454 | | 0.054 / 0.541 | |

| **Experiment 10 fake political news, Democrats only** | | | | |
| --- | --- | --- | --- | --- |
|  | **sharing** | | **sharing** | |
| *Predictors* | *ß* | *p* | *ß* | *p* |
| Condition [message myside sharing] | -0.25 (-0.35, -0.15) | **<0.001** | -0.24 (-0.34, -0.14) | **<0.001** |
| edu | -0.05 (-0.10, -0.00) | **0.047** | -0.05 (-0.10, -0.00) | **0.039** |
| sex [female] | -0.09 (-0.19,0.01) | 0.063 | -0.10 (-0.20, -0.00) | **0.045** |
| age | -0.07 (-0.12, -0.02) | **0.005** | -0.07 (-0.12, -0.02) | **0.005** |
| Congruent [TRUE] |  |  | 0.41 (0.30,0.52) | **<0.001** |
| Congruent [TRUE] * Condition [message myside sharing] |  |  | -0.07 (-0.19,0.06) | 0.285 |
| **Random Effects** | | | | |
| σ^2^ | 0.42 | | 0.42 | |
| τ_00_ | 0.21 _id_ | | 0.21 _id_ | |
|  | 0.09 _News_ | | 0.04 _News_ | |
| τ_11_ | 0.29 _id.CongruentTRUE_ | | 0.27 _id.CongruentTRUE_ | |
| ρ_01_ | 0.17 _id_ | | 0.16 _id_ | |
| ICC | 0.42 | | 0.50 | |
| Marginal R^2^ / Conditional R^2^ | 0.036 / 0.439 | | 0.072 / 0.539 | |

### Congruence x Condition on Republicans only (restricted analyses)

| **Experiment 10 true political news, Republicans only** | | | | |
| --- | --- | --- | --- | --- |
|  | **sharing** | | **sharing** | |
| *Predictors* | *ß* | *p* | *ß* | *p* |
| Condition [message myside sharing] | -0.13 (-0.24, -0.02) | **0.025** | -0.14 (-0.25, -0.02) | **0.020** |
| edu | -0.09 (-0.14, -0.03) | **0.002** | -0.09 (-0.14, -0.03) | **0.002** |
| sex [female] | -0.13 (-0.24, -0.02) | **0.025** | -0.13 (-0.24, -0.02) | **0.024** |
| age | -0.00 (-0.06,0.05) | 0.888 | -0.00 (-0.06,0.05) | 0.881 |
| Congruent [TRUE] |  |  | 0.24 (0.16,0.31) | **<0.001** |
| Congruent [TRUE] * Condition [message myside sharing] |  |  | 0.03 (-0.08,0.14) | 0.573 |
| **Random Effects** | | | | |
| σ^2^ | 0.49 | | 0.49 | |
| τ_00_ | 0.26 _id_ | | 0.26 _id_ | |
|  | 0.06 _News_ | | 0.06 _News_ | |
| τ_11_ | 0.18 _id.CongruentTRUE_ | | 0.13 _id.CongruentTRUE_ | |
| ρ_01_ | 0.07 _id_ | | 0.14 _id_ | |
| ICC | 0.40 | | 0.46 | |
| Marginal R^2^ / Conditional R^2^ | 0.017 / 0.408 | | 0.030 / 0.473 | |

| **Experiment 10 fake political news, Republicans only** | | | | |
| --- | --- | --- | --- | --- |
|  | **sharing** | | **sharing** | |
| *Predictors* | *ß* | *p* | *ß* | *p* |
| Condition [message myside sharing] | -0.12 (-0.23, -0.01) | **0.034** | -0.12 (-0.23, -0.01) | **0.027** |
| edu | -0.08 (-0.14, -0.03) | **0.003** | -0.08 (-0.14, -0.03) | **0.003** |
| sex [female] | 0.01 (-0.09,0.12) | 0.794 | 0.01 (-0.10,0.12) | 0.806 |
| age | -0.00 (-0.06,0.05) | 0.930 | -0.00 (-0.06,0.05) | 0.919 |
| Congruent [TRUE] |  |  | 0.24 (0.17,0.31) | **<0.001** |
| Congruent [TRUE] * Condition [message myside sharing] |  |  | 0.06 (-0.04,0.16) | 0.258 |
| **Random Effects** | | | | |
| σ^2^ | 0.44 | | 0.44 | |
| τ_00_ | 0.23 _id_ | | 0.23 _id_ | |
|  | 0.09 _News_ | | 0.09 _News_ | |
| τ_11_ | 0.17 _id.CongruentTRUE_ | | 0.11 _id.CongruentTRUE_ | |
| ρ_01_ | 0.31 _id_ | | 0.41 _id_ | |
| ICC | 0.42 | | 0.50 | |
| Marginal R^2^ / Conditional R^2^ | 0.012 / 0.429 | | 0.027 / 0.511 | |

### Congruence x Issue moralization x Condition

| **Experiment 10 true political news** | | |
| --- | --- | --- |
|  | **sharing** | |
| *Predictors* | *ß* | *p* |
| Congruent [TRUE] | 0.39 (0.32,0.45) | **<0.001** |
| AbsImpIssue [TRUE] | -0.01 (-0.07,0.05) | 0.754 |
| Condition [message myside sharing] | -0.21 (-0.29, -0.12) | **<0.001** |
| edu | -0.07 (-0.10, -0.03) | **0.001** |
| sex [female] | -0.13 (-0.21, -0.05) | **0.001** |
| age | -0.05 (-0.08, -0.01) | **0.016** |
| Congruent [TRUE] * AbsImpIssue [TRUE] | 0.26 (0.17,0.35) | **<0.001** |
| Congruent [TRUE] * Condition [message myside sharing] | -0.01 (-0.11,0.08) | 0.775 |
| AbsImpIssue [TRUE] * Condition [message myside sharing] | 0.11 (0.02,0.20) | **0.020** |
| (Congruent [TRUE] * AbsImpIssue [TRUE]) * Condition [message myside sharing] | -0.02 (-0.14,0.11) | 0.809 |
| **Random Effects** | | |
| σ^2^ | 0.49 | |
| τ_00_ _id_ | 0.26 | |
| τ_00_ _News_ | 0.04 | |
| τ_11_ _id.CongruentTRUE_ | 0.19 | |
| ρ_01_ _id_ | 0.19 | |
| ICC | 0.47 | |
| Marginal R^2^ / Conditional R^2^ | 0.086 / 0.515 | |

| **Experiment 10 fake political news** | | |
| --- | --- | --- |
|  | **sharing** | |
| *Predictors* | *ß* | *p* |
| Congruent [TRUE] | 0.45 (0.38,0.52) | **<0.001** |
| AbsImpIssue [TRUE] | 0.04 (-0.02,0.10) | 0.191 |
| Condition [message myside sharing] | -0.19 (-0.28, -0.11) | **<0.001** |
| edu | -0.08 (-0.11, -0.04) | **<0.001** |
| sex [female] | -0.07 (-0.14,0.01) | 0.081 |
| age | -0.03 (-0.07,0.01) | 0.141 |
| Congruent [TRUE] * AbsImpIssue [TRUE] | 0.27 (0.19,0.36) | **<0.001** |
| Congruent [TRUE] * Condition [message myside sharing] | -0.02 (-0.12,0.08) | 0.744 |
| AbsImpIssue [TRUE] * Condition [message myside sharing] | -0.01 (-0.09,0.08) | 0.898 |
| (Congruent [TRUE] * AbsImpIssue [TRUE]) * Condition [message myside sharing] | 0.02 (-0.10,0.14) | 0.745 |
| **Random Effects** | | |
| σ^2^ | 0.45 | |
| τ_00_ _id_ | 0.24 | |
| τ_00_ _News_ | 0.02 | |
| τ_11_ _id.CongruentTRUE_ | 0.24 | |
| ρ_01_ _id_ | 0.07 | |
| ICC | 0.47 | |
| Marginal R^2^ / Conditional R^2^ | 0.107 / 0.525 | |

### Congruence x Attitude extremity x Condition

| **Experiment 10 true political news** | | |
| --- | --- | --- |
|  | **sharing** | |
| *Predictors* | *ß* | *p* |
| Congruent [TRUE] | 0.39 (0.32,0.45) | **<0.001** |
| AbsImpIssue [TRUE] | -0.01 (-0.07,0.05) | 0.754 |
| Condition [message myside sharing] | -0.21 (-0.29, -0.12) | **<0.001** |
| edu | -0.07 (-0.10, -0.03) | **0.001** |
| sex [female] | -0.13 (-0.21, -0.05) | **0.001** |
| age | -0.05 (-0.08, -0.01) | **0.016** |
| Congruent [TRUE] * AbsImpIssue [TRUE] | 0.26 (0.17,0.35) | **<0.001** |
| Congruent [TRUE] * Condition [message myside sharing] | -0.01 (-0.11,0.08) | 0.775 |
| AbsImpIssue [TRUE] * Condition [message myside sharing] | 0.11 (0.02,0.20) | **0.020** |
| (Congruent [TRUE] * AbsImpIssue [TRUE]) * Condition [message myside sharing] | -0.02 (-0.14,0.11) | 0.809 |
| **Random Effects** | | |
| σ^2^ | 0.49 | |
| τ_00_ _id_ | 0.26 | |
| τ_00_ _News_ | 0.04 | |
| τ_11_ _id.CongruentTRUE_ | 0.19 | |
| ρ_01_ _id_ | 0.19 | |
| ICC | 0.47 | |
| Marginal R^2^ / Conditional R^2^ | 0.086 / 0.515 | |

| **Experiment 10 fake political news** | | |
| --- | --- | --- |
|  | **sharing** | |
| *Predictors* | *ß* | *p* |
| Congruent [TRUE] | 0.45 (0.38,0.52) | **<0.001** |
| AbsImpIssue [TRUE] | 0.04 (-0.02,0.10) | 0.191 |
| Condition [message myside sharing] | -0.19 (-0.28, -0.11) | **<0.001** |
| edu | -0.08 (-0.11, -0.04) | **<0.001** |
| sex [female] | -0.07 (-0.14,0.01) | 0.081 |
| age | -0.03 (-0.07,0.01) | 0.141 |
| Congruent [TRUE] * AbsImpIssue [TRUE] | 0.27 (0.19,0.36) | **<0.001** |
| Congruent [TRUE] * Condition [message myside sharing] | -0.02 (-0.12,0.08) | 0.744 |
| AbsImpIssue [TRUE] * Condition [message myside sharing] | -0.01 (-0.09,0.08) | 0.898 |
| (Congruent [TRUE] * AbsImpIssue [TRUE]) * Condition [message myside sharing] | 0.02 (-0.10,0.14) | 0.745 |
| **Random Effects** | | |
| σ^2^ | 0.45 | |
| τ_00_ _id_ | 0.24 | |
| τ_00_ _News_ | 0.02 | |
| τ_11_ _id.CongruentTRUE_ | 0.24 | |
| ρ_01_ _id_ | 0.07 | |
| ICC | 0.47 | |
| Marginal R^2^ / Conditional R^2^ | 0.107 / 0.525 | |

# O. New items selection questions

NB: These items were the same whether the items were true or fake.

## Question to assess which side on each issue the news story was congruent to

Assuming that this news story is true, is it more favorable to Democrats or Republicans?

|  | Democrats | Republicans |
| --- | --- | --- |

|  | 0 | 10 | 20 | 30 | 40 | 50 | 60 | 70 | 80 | 90 | 100 |
| --- | --- | --- | --- | --- | --- | --- | --- | --- | --- | --- | --- |

| () | **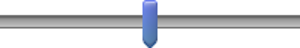** |
| --- | --- |

## Accuracy calibration question used to select the fake news

Perceived accuracy had to be comprised between 3, “Somewhat inaccurate” and 5, “Somewhat accurate” on a 1-7 Likert scale.

How accurate do you think this news story is?

|  | Completely inaccurate (1) | Pretty inaccurate (2) | Somewhat inaccurate (3) | Undecided (4) | Somewhat accurate (5) | Pretty accurate (6) | Completely accurate (7) |
| --- | --- | --- | --- | --- | --- | --- | --- |
|  | o | o | o | o | o | o | o |

# P. True partisan news items

Above each headline is provided its label in the R scripts. In the label “GunCo.D”, “GunCo” specifies the issue, and “D” that it was congruent for liberals-Democrats on the issue (in contrast, “R” means that an item was congruent for conservatives-Republicans on the issue).

### Experiments 1-9

**Gun Control**

Congruent for Liberals (GunCo.D):


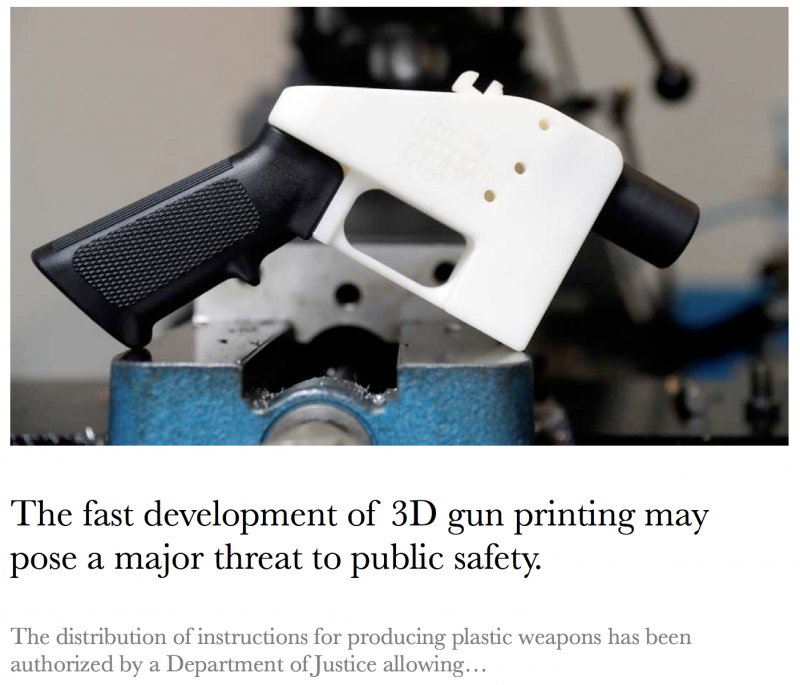


Inspired from: <https://www.rand.org/blog/articles/2018/05/four-ways-3d-printing-may-threaten-security.html>

Congruent for Conservatives (GunCo.R):


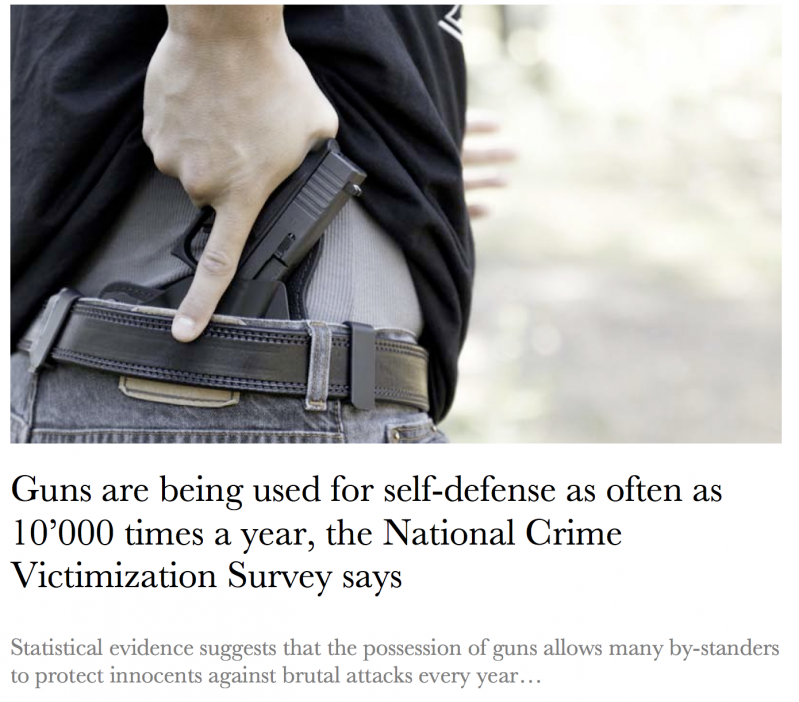


Inspired from: https://www.npr.org/2018/04/13/602143823/how-often-do-people-use-guns-in-self-defense?t=1614276238706

**Racial Equality**

Congruent for Liberals (Race.D) :


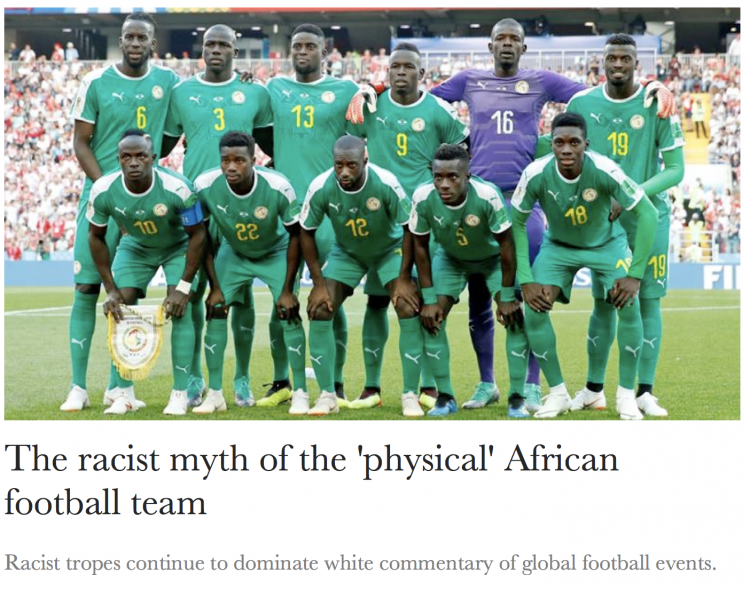


Source: https://www.aljazeera.com/opinions/2018/6/27/the-racist-myth-of-the-physical-african-football-team

Congruent for Conservatives (Race.R):


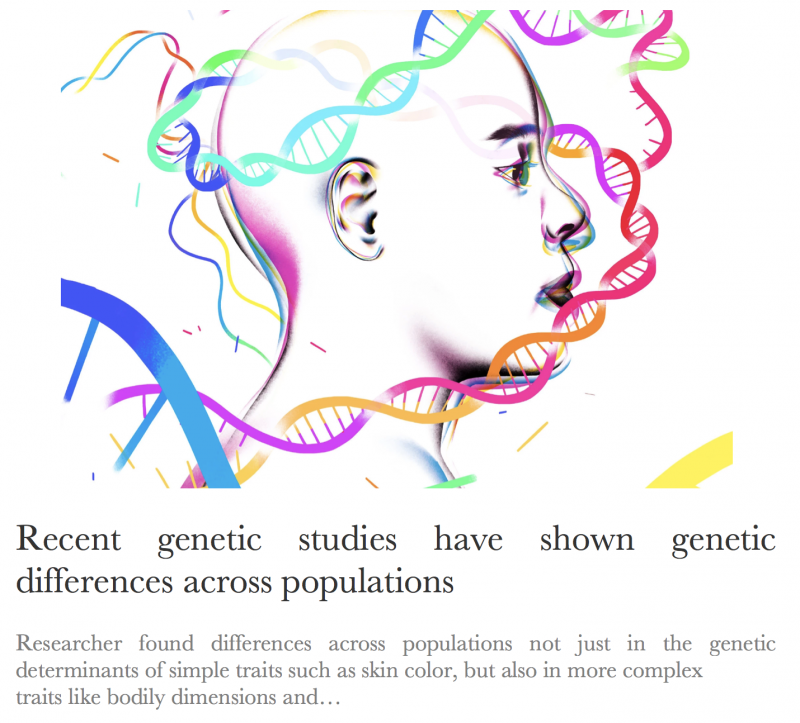


Inspired from: <https://bmcgenomics.biomedcentral.com/articles/10.1186/s12864-015-2328-0>

**Gender Equality**

Congruent for Liberals (Gender.D)


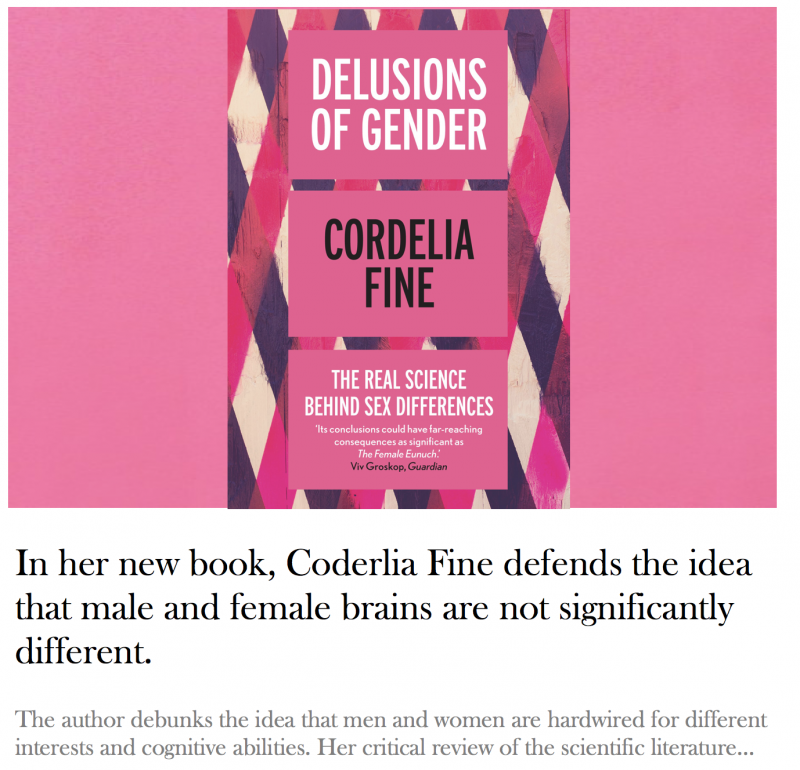


Inspired from: <https://wwnorton.com/books/Delusions-of-Gender>

Congruent for Conservatives (Gender.R)


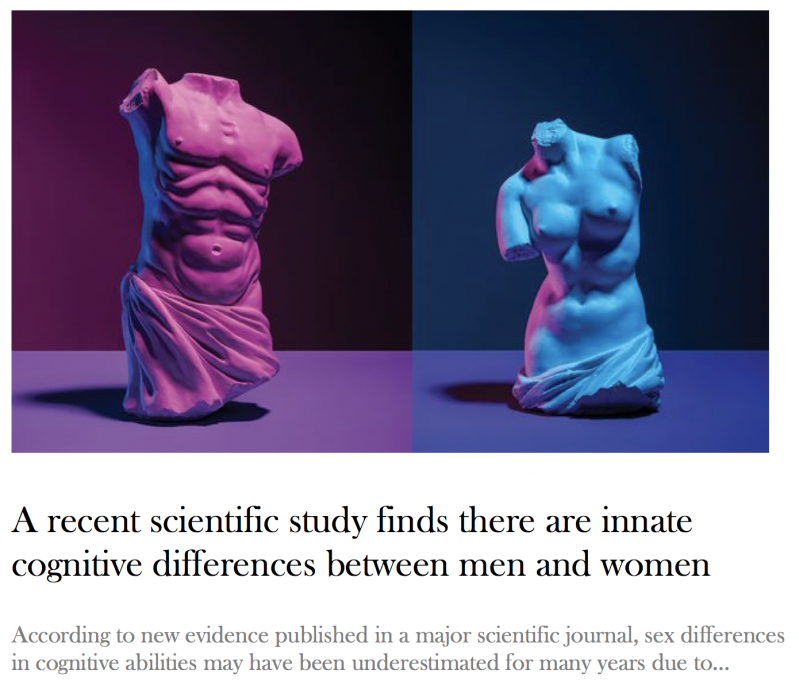


Inspired from: <https://ifstudies.org/blog/straight-talk-about-sex-differences-in-occupational-choices-and-work-family-tradeoffs>

**Abortion**

Congruent for Liberals (Abor.D)


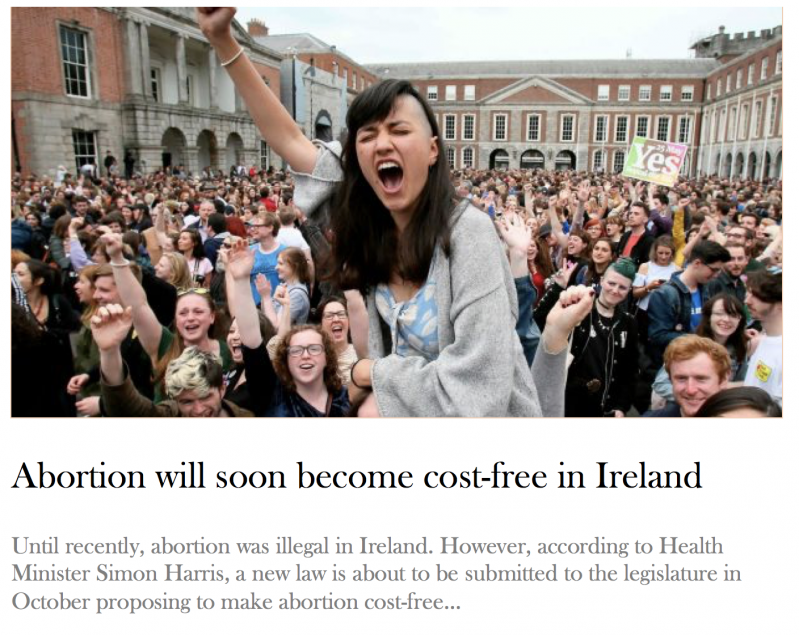


Inspired from: https://eu.usatoday.com/story/news/nation-now/2018/09/20/ireland-abortions-cost-free-women-official-says/1376884002/

Congruent for Conservatives (Abor.R)


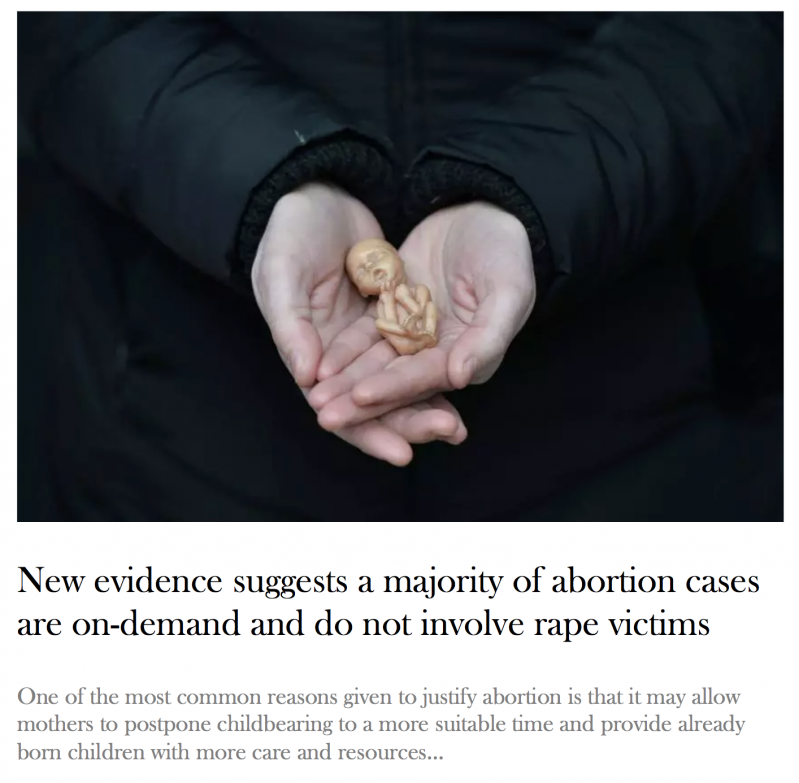


Source: https://www.scmp.com/comment/letters/article/2152591/most-abortion-cases-are-demand-and-do-not-involve-rape-victims

### Raters’ perceptions of true political news items’ slant

The following graphs display perceptions of true news items’ political slant according to MTurk raters:

Means and 95 CI. All one sample t-tests (one-tailed) yield p values < 0.05.

**
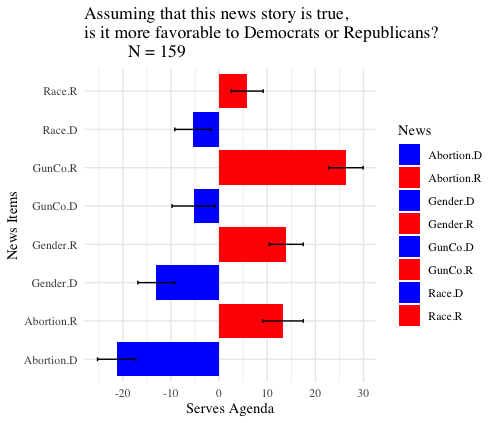
**

Smaller deviations from 0 are for Agenda_Race.D: t(158) = -2.84, p = 0.002 and Agenda_GunCo.D: t(158) = -2.34, p = 0.01 (one tailed tests)

### Experiment 10

Abortion

Congruent to Democrats: t_abor_d_1


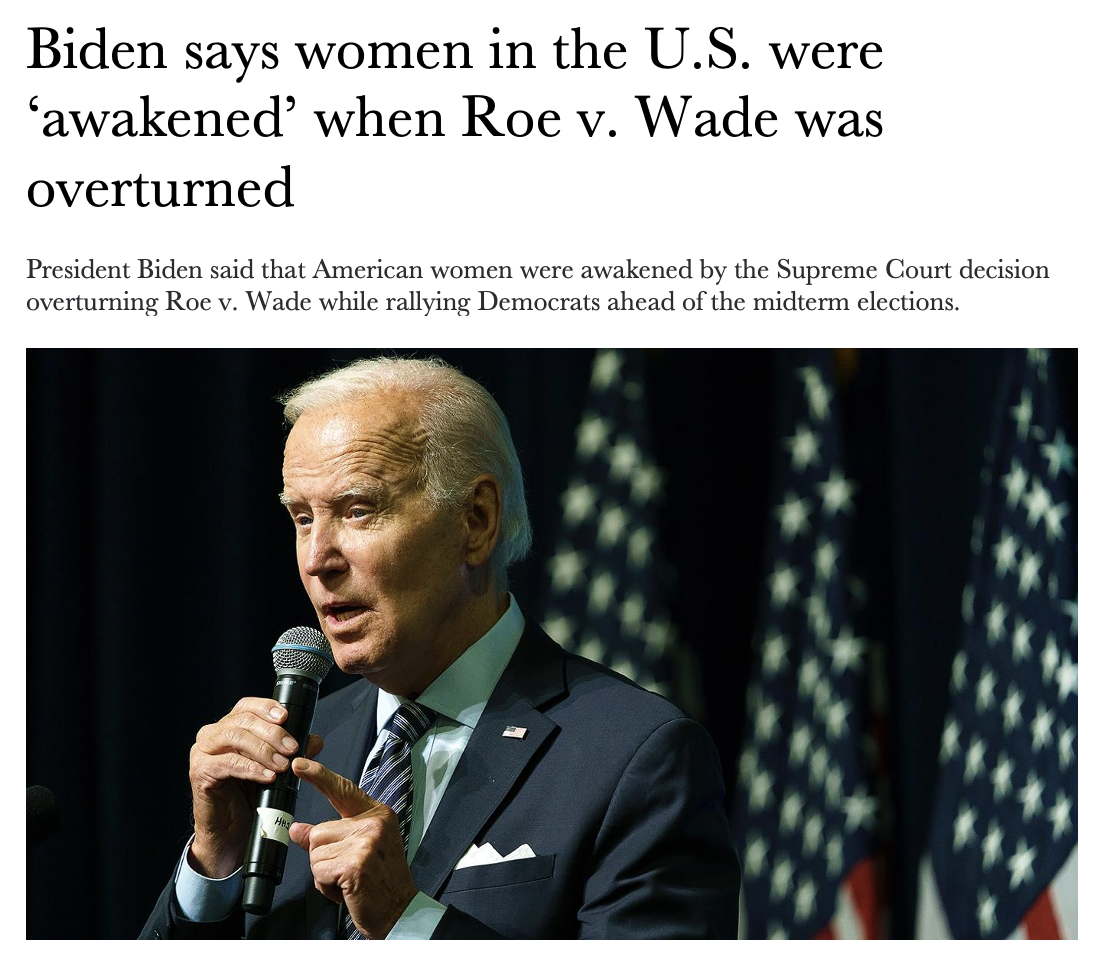


Congruent to Republicans:

t_abor_r_1


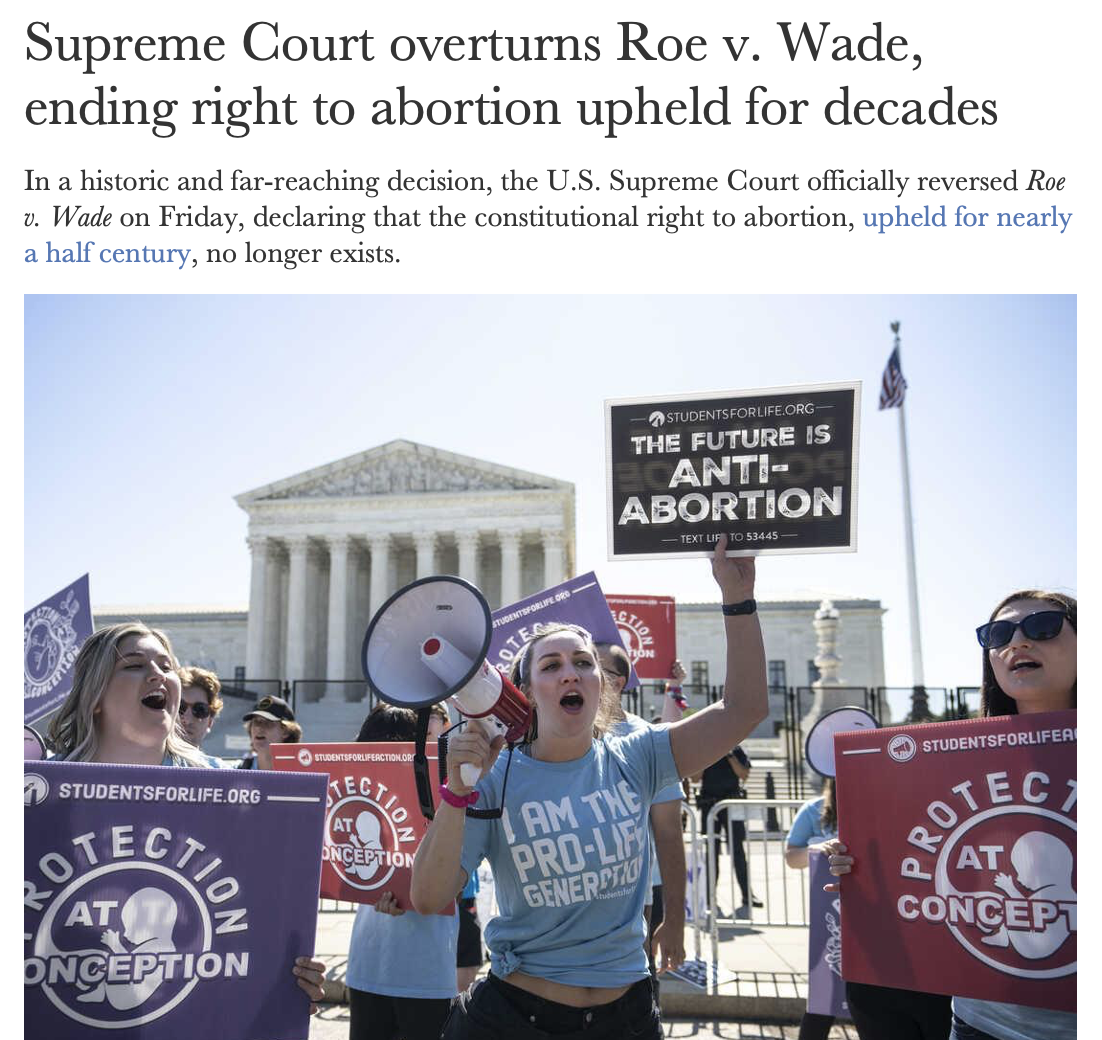


Congruent to Democrats:

t_gender_d_2


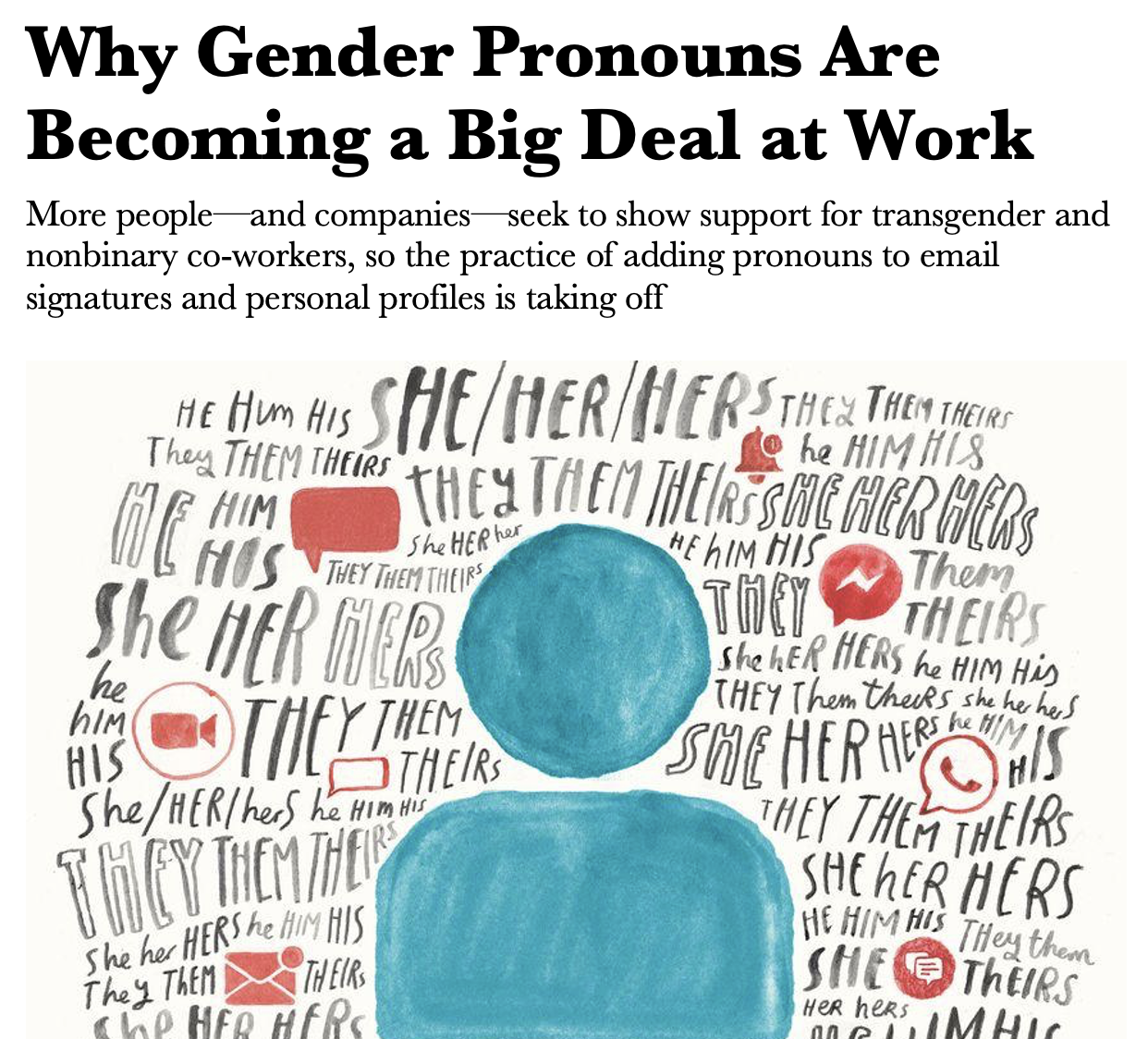


Congruent to Republicans:

t_gender_r_1


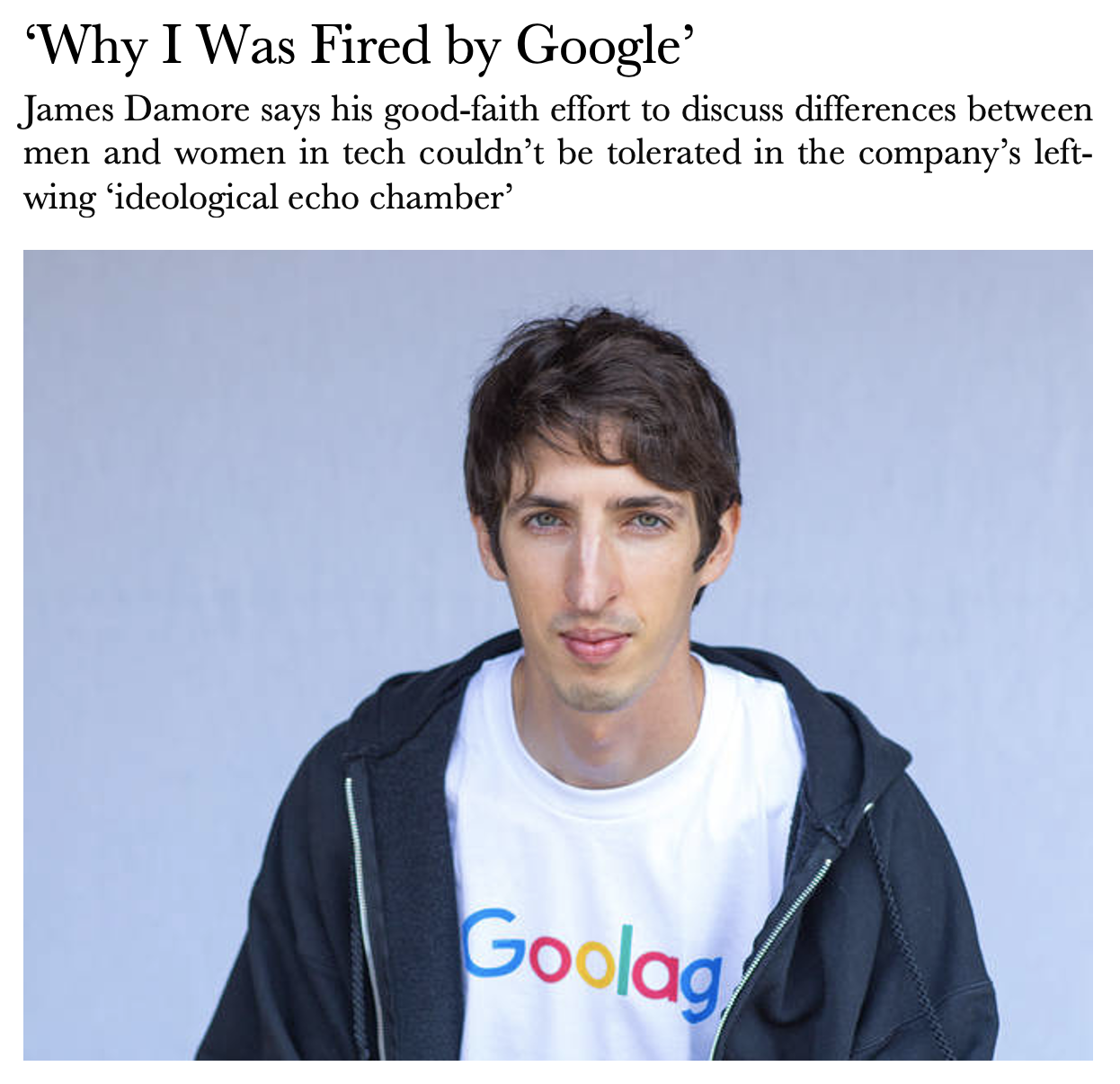


**Gun control**

Congruent to Democrats:

t_gender_d_2


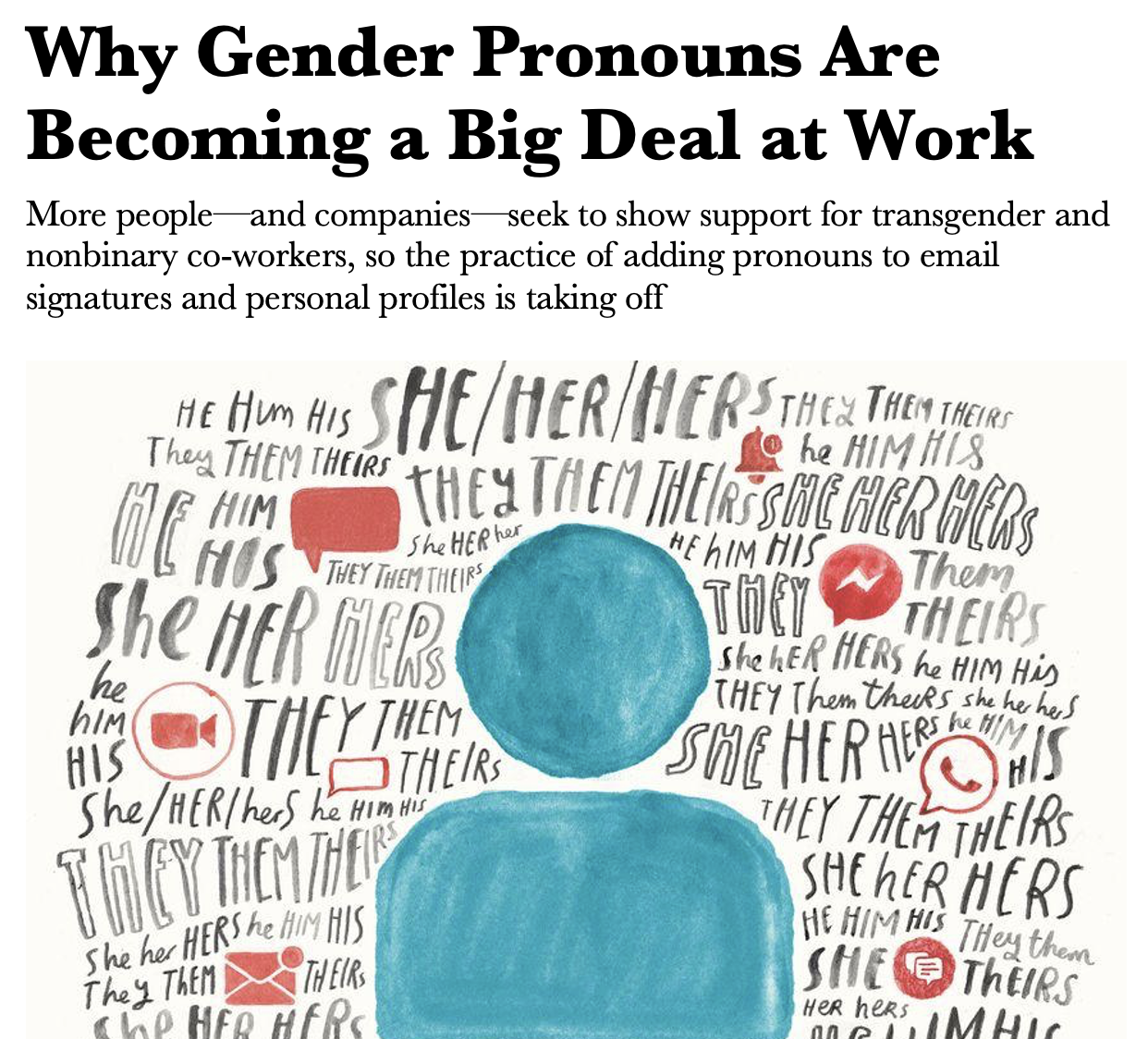


Congruent to Republicans:

t_guns_r_1


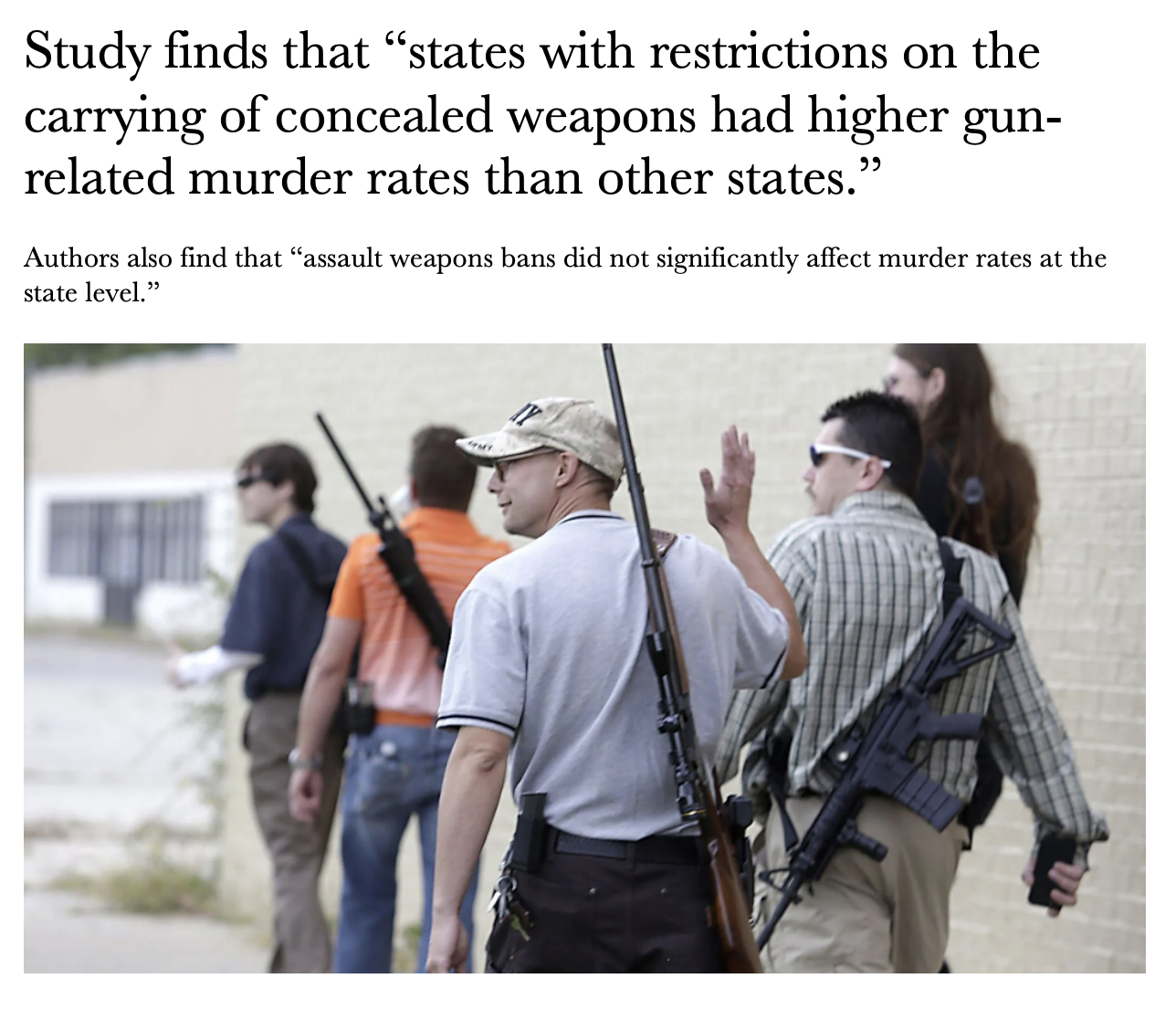


Congruent to Democrats: t_guns_d_4


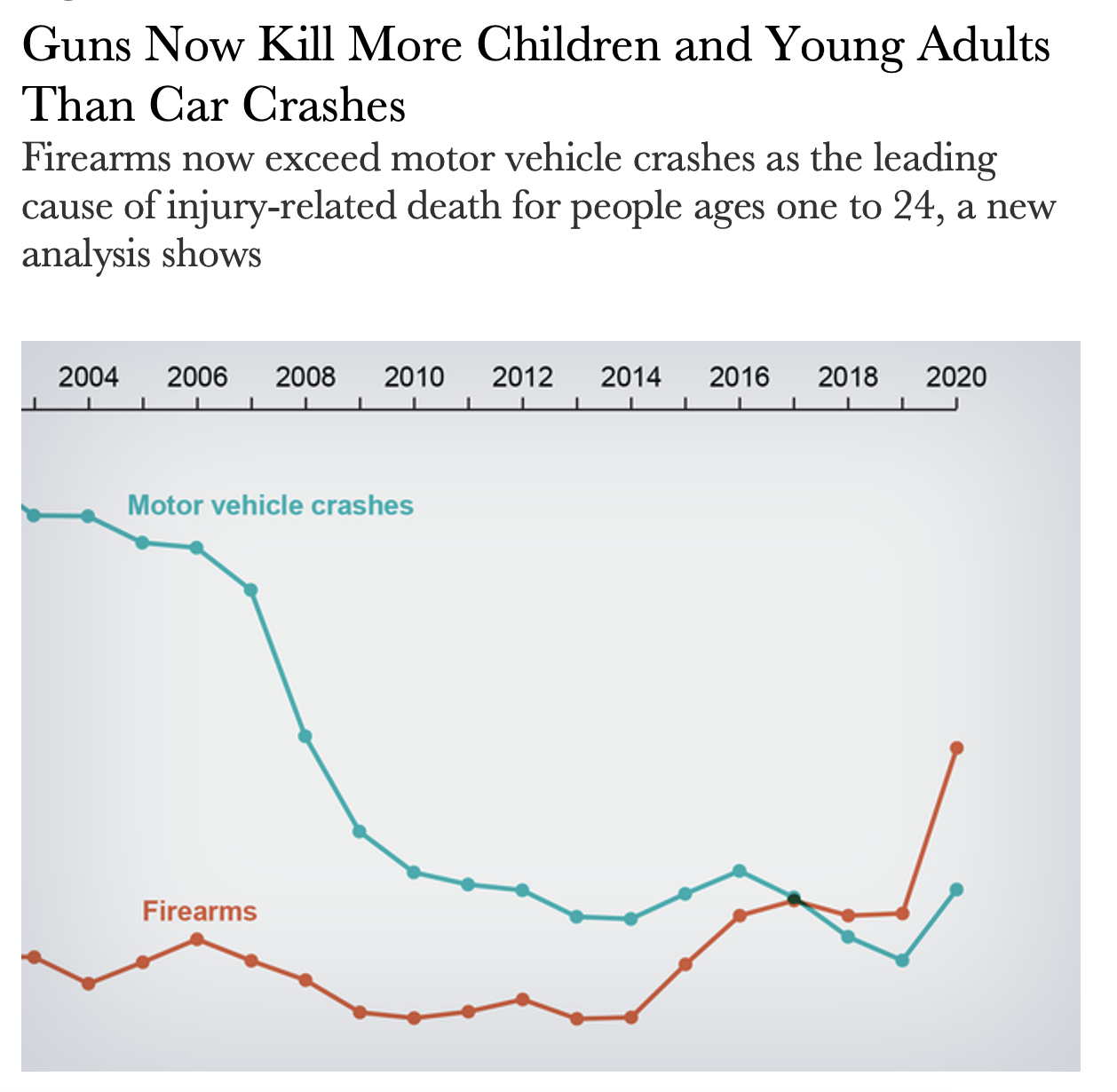


**Immigration**

Congruent to Democrats: **t_immi_d_2**

**
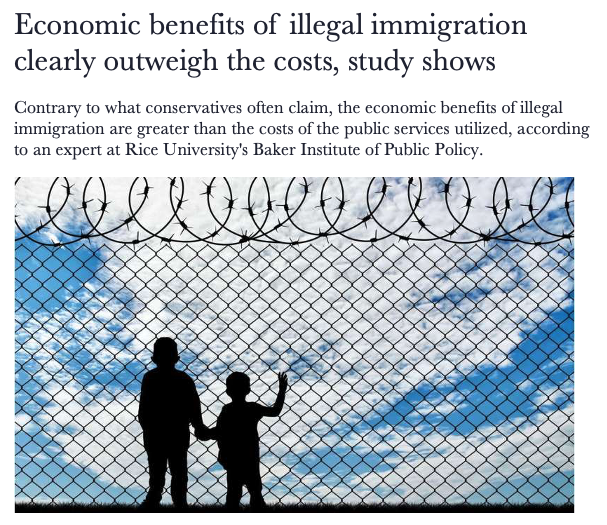
**

Congruent to Republicans: t_immi_r_1


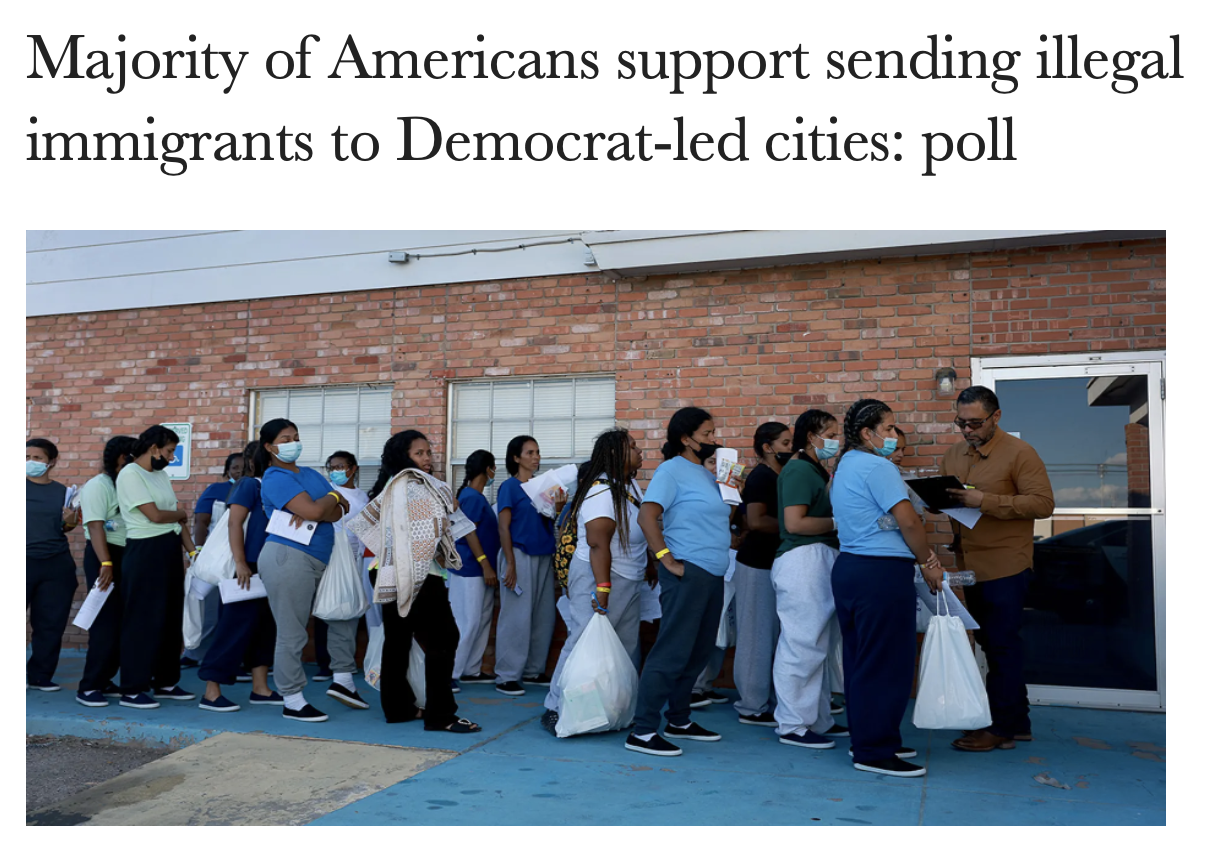


**Race**

Congruent to Democrats: **t_race_d_1**

**
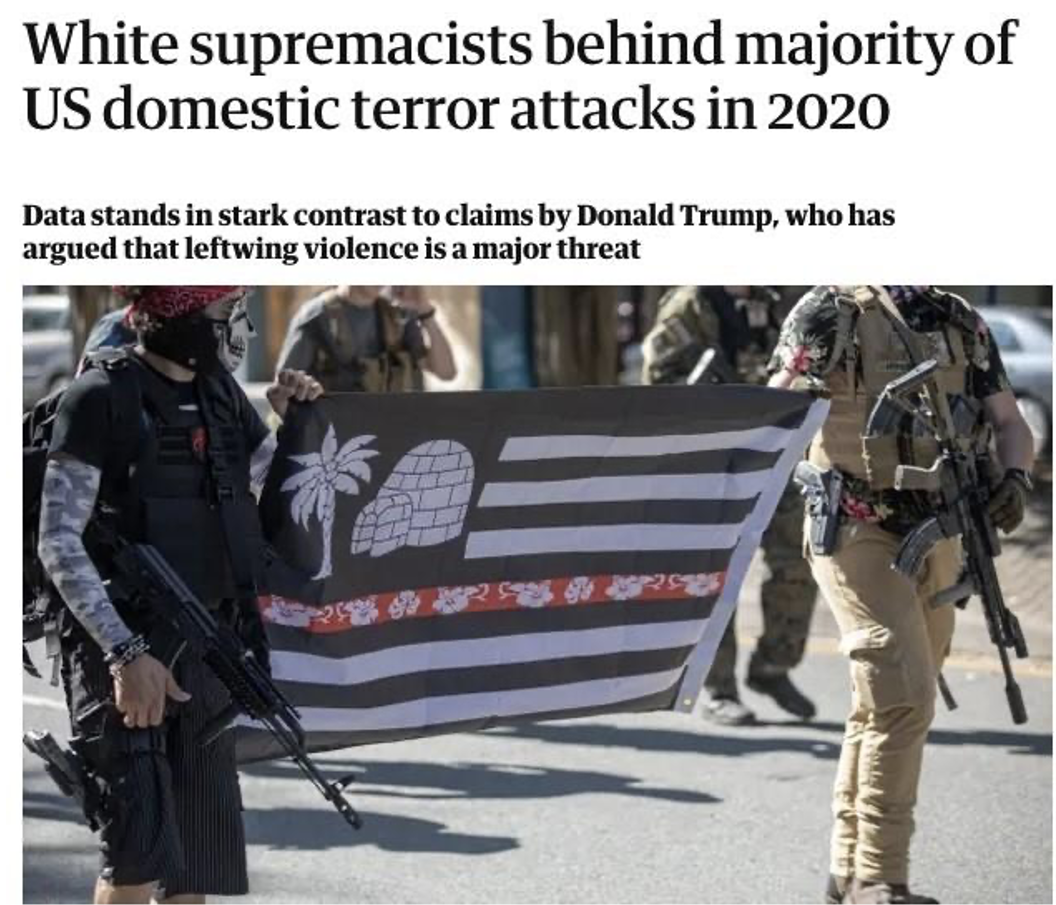
**

Congruent to Republicans:

**t_race_r_1**

**
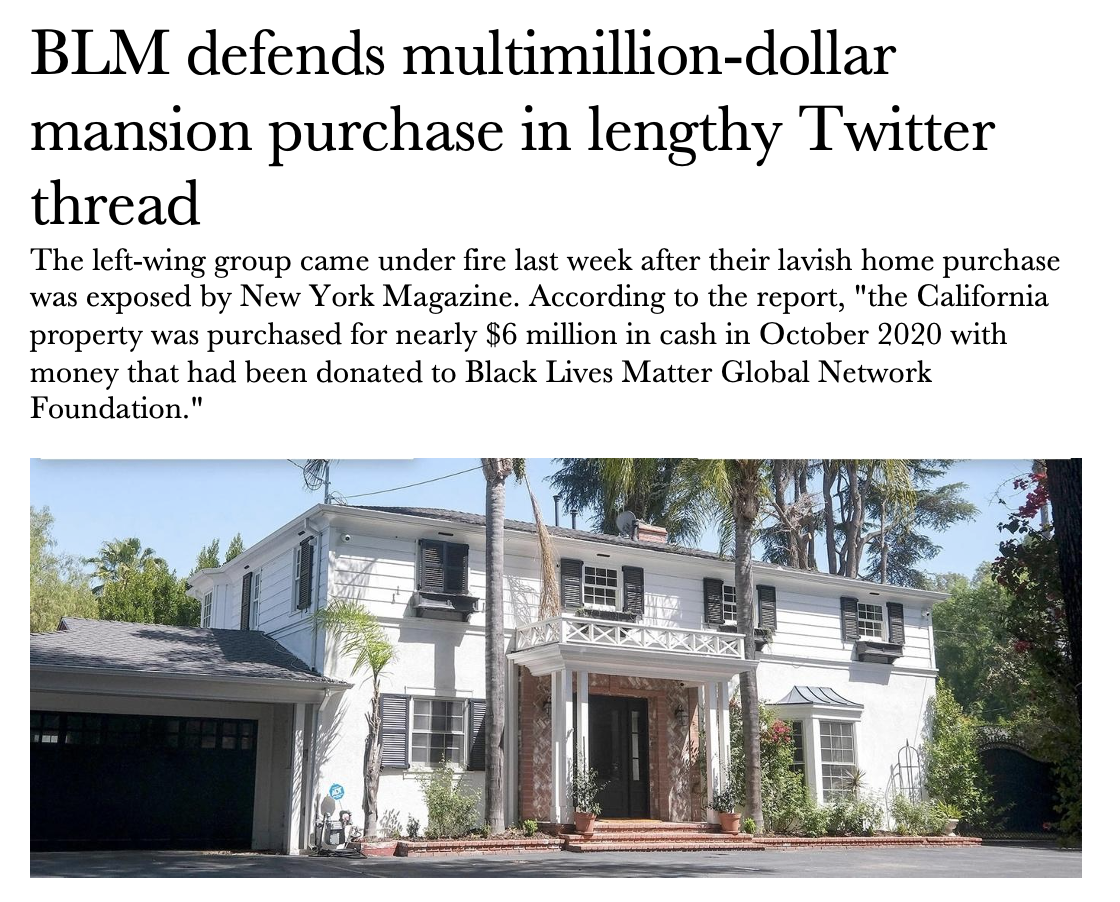
**

### Raters’ perceptions of true political news items’ slant

The following plot shows perceptions of fake news items’ political slant according to U.S. Prolific raters. Means and 95 CI.

NB : Among the true neutral news, only t_neut_smart and t_neut_greece were used in Experiment 10.

# Q. True neutral news items

### Experiments 1-9

Neutral.1:


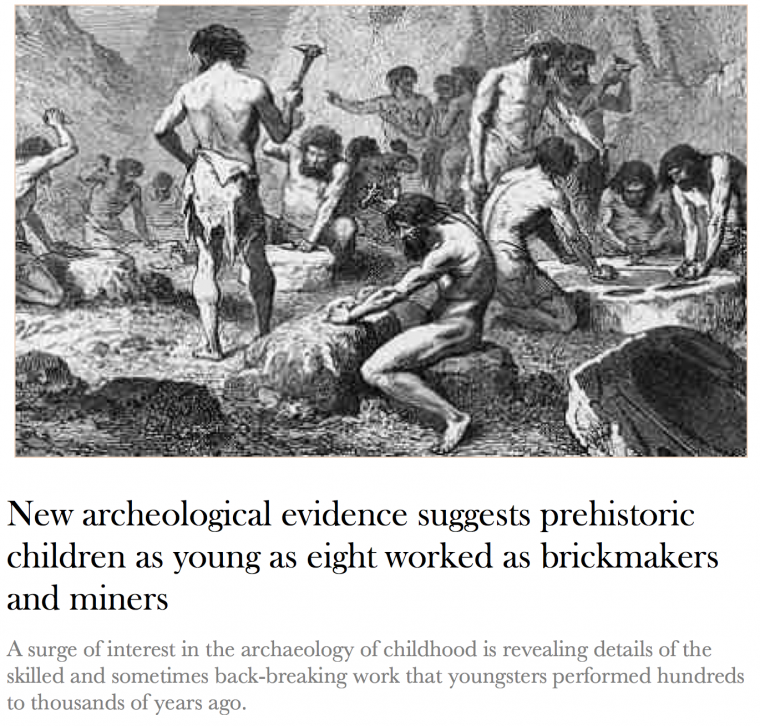


Source: <https://www.nature.com/articles/d41586-018-06747-w>

Neutral.2:


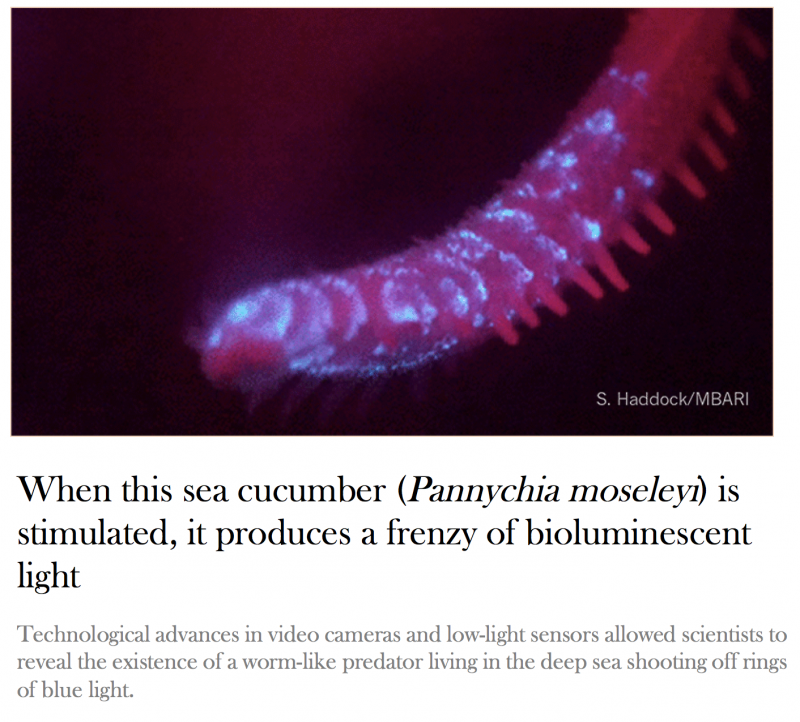


Inspired from: <https://www.nature.com/articles/d41586-018-06660-2?WT.feed_name=subjects_zoology>

Neutral.3:


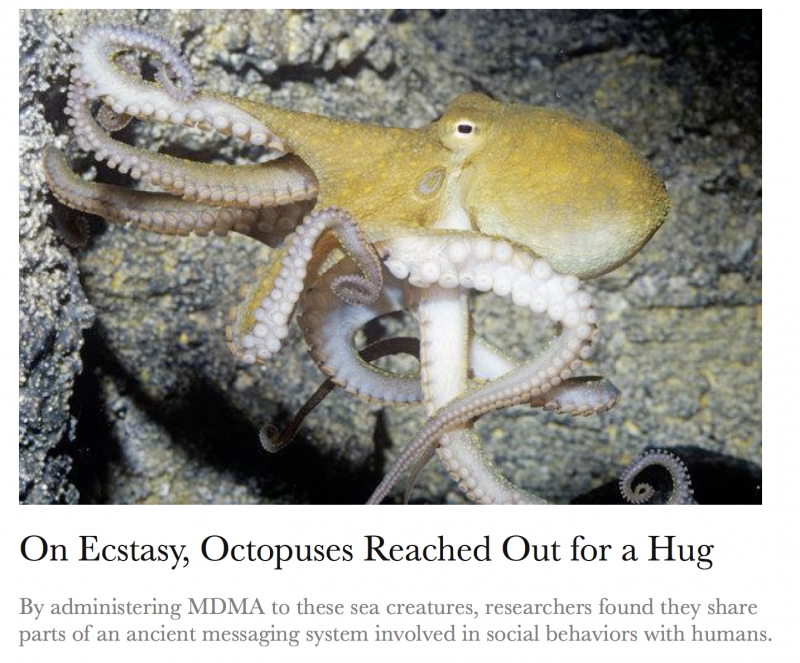


Source: <https://www.nytimes.com/2018/09/20/science/octopus-ecstasy-mdma.html>

Neutral.4:


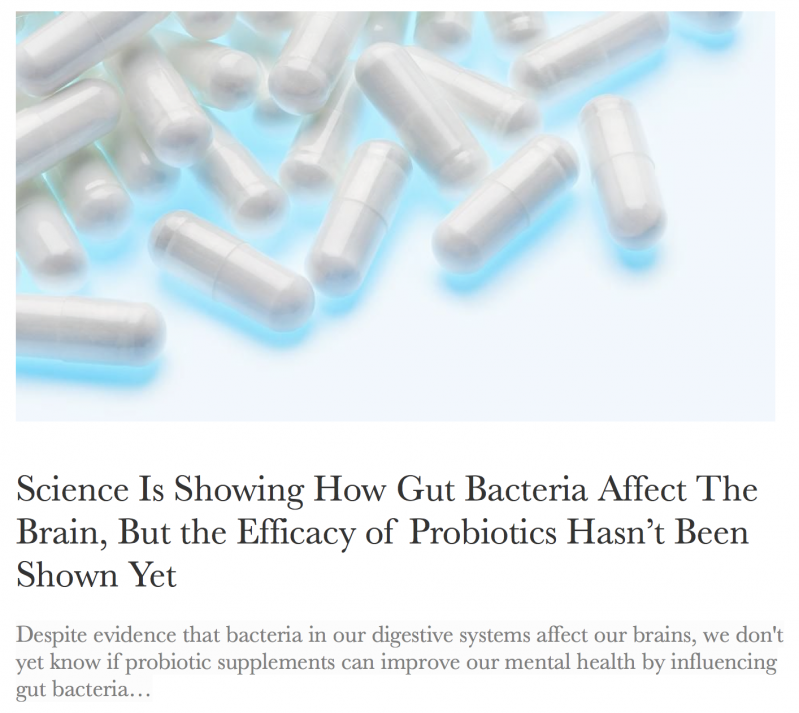


Source: https://www.forbes.com/sites/daviddisalvo/2017/08/27/science-is-showing-how-gut-bacteria-affect-the-brain-but-dont-bother-taking-probiotics-yet/?sh=2778912735a5

### Experiment 10

t_neut_smart


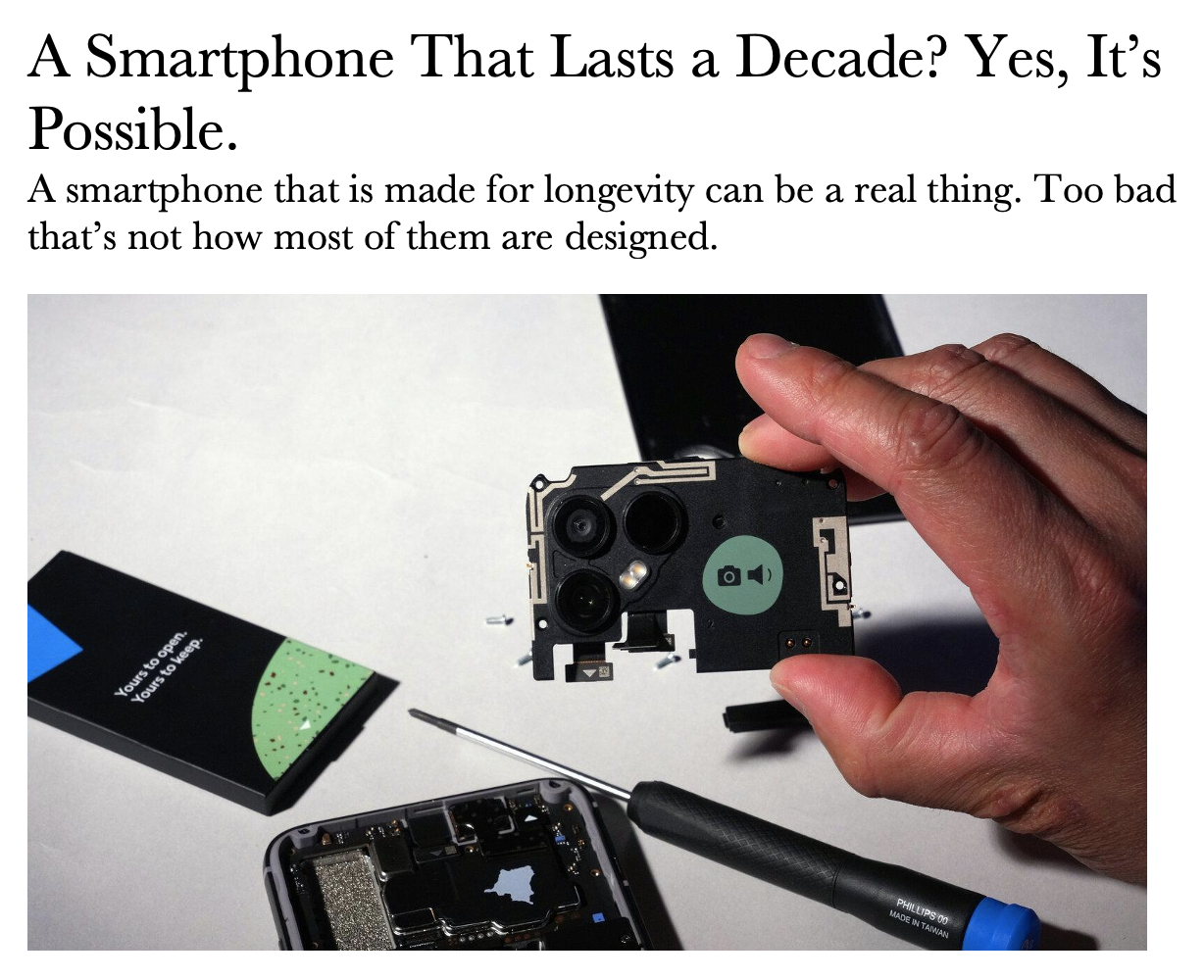


t_neut_greece

**
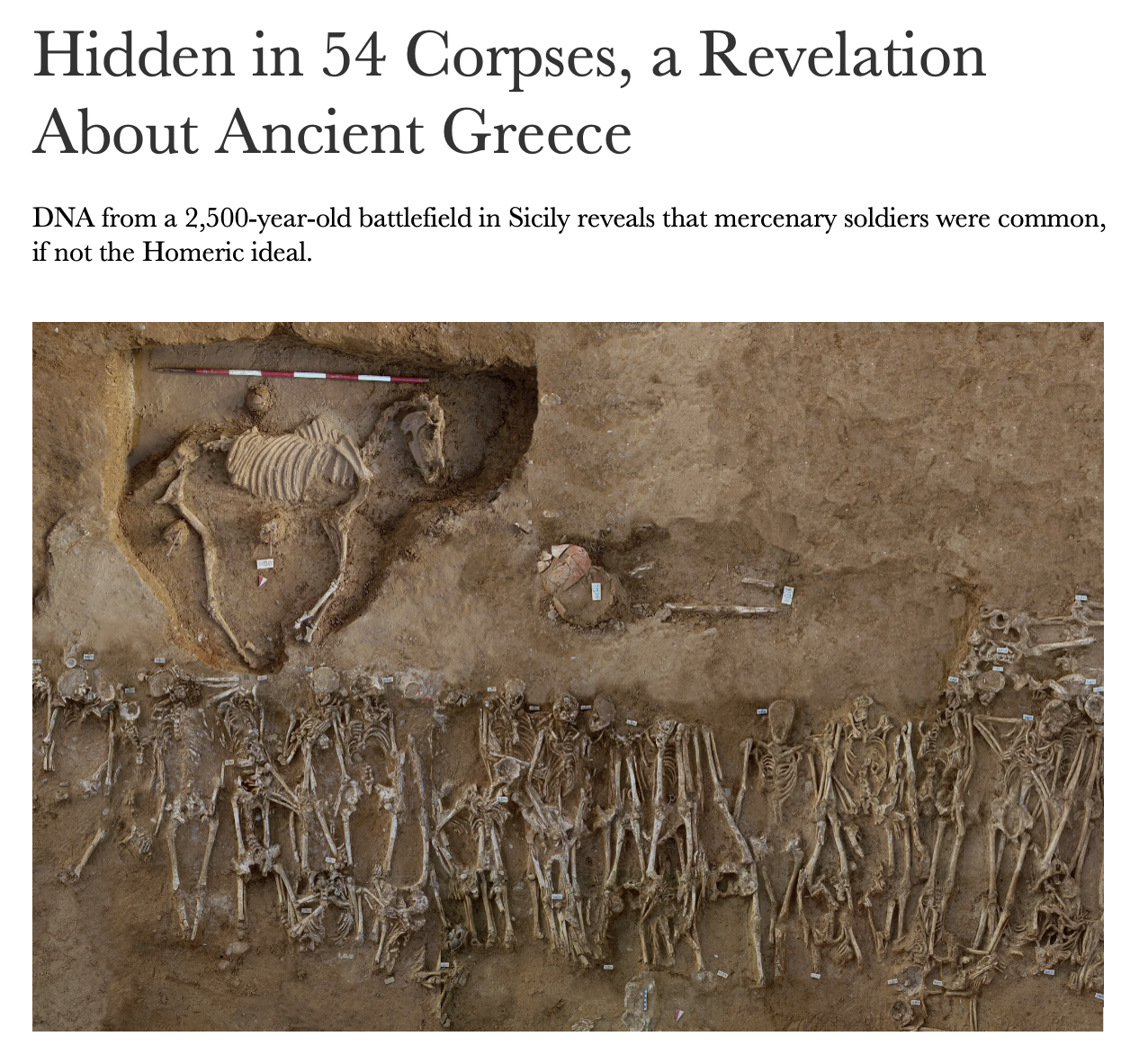
**

# R. Fake partisan news items

### Experiments 1-9

**Gun control**

Congruent for Liberals (GunCo D_fake/ GunCo D_Mental):


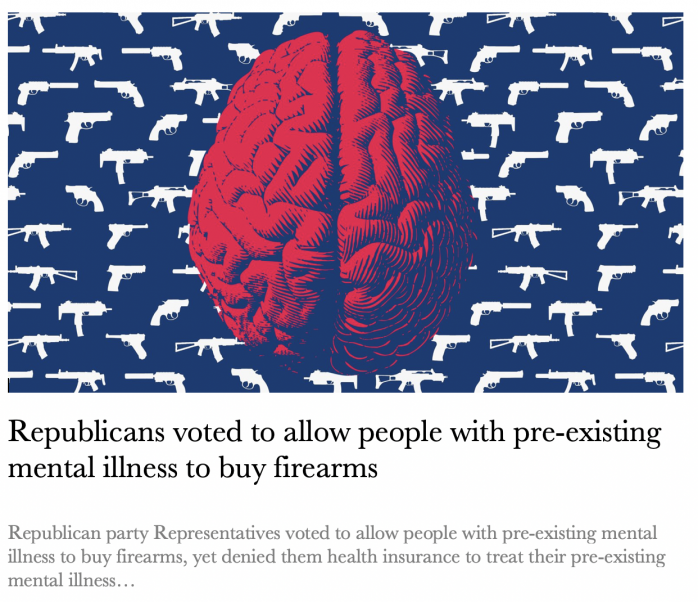


Inspired from: https://www.politifact.com/factchecks/2019/apr/22/eric-swalwell/did-republicans-rush-repeal-gun-control-law-mental/

Congruent for Conservatives (GunCo.R_fake/ GunCo.R_Chicago):


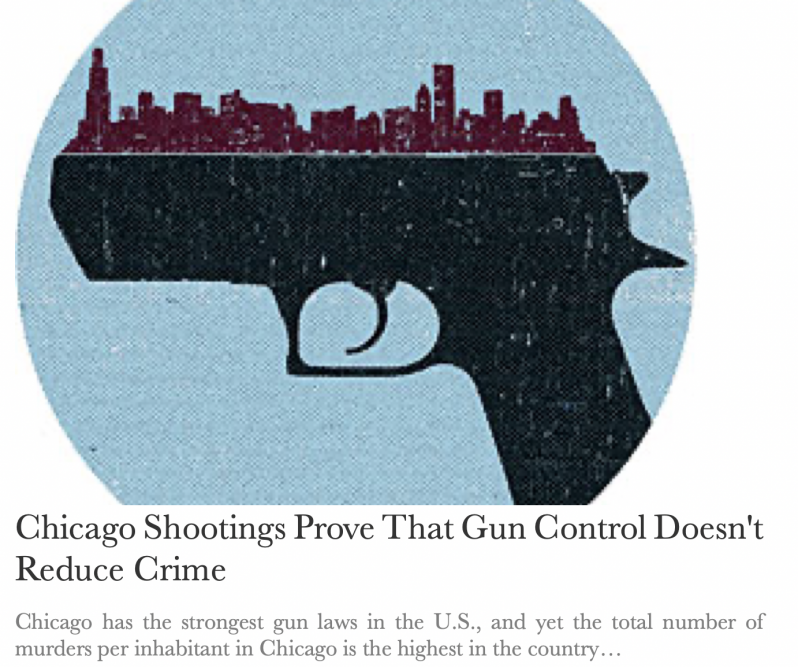


Inspired from: <https://www.npr.org/2017/10/05/555580598/fact-check-is-chicago-proof-that-gun-laws-don-t-work?t=1614759843909>

<https://www.politifact.com/factchecks/2017/oct/03/sarah-huckabee-sanders/chicago-toughest-gun-control-claim-shot-full-holes/>

**Racial equality**

Congruent for Liberals (Race.D_FreeTuition):


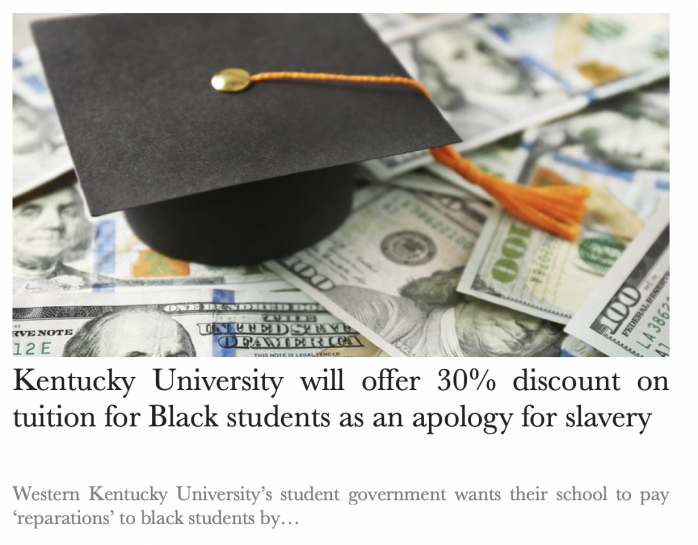


Inspired from: <https://www.brookings.edu/blog/brown-center-chalkboard/2019/06/27/to-boost-black-students-we-should-give-free-college-to-students-who-dont-need-it/>

<https://www.vox.com/identities/2017/8/3/16084202/minority-students-free-college-myth-trump-affirmative-action>

Congruent for Conservatives (Race.R_Mohammed):


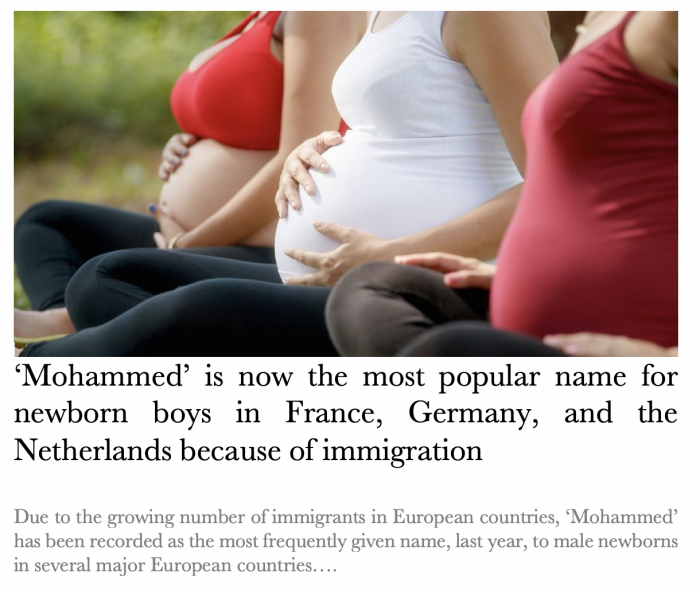


Inspired from: <https://ec.europa.eu/home-affairs/sites/homeaffairs/files/what-we-do/policies/european-agenda-migration/20190306_managing-migration-factsheet-debunking-myths-about-migration_en.pdf>

**Gender equality**

Congruent for Liberals (Gender.D_PayGap):


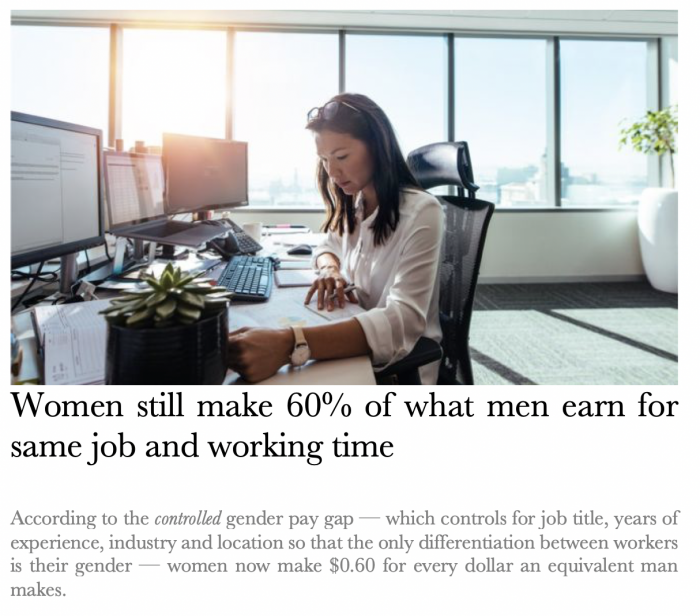


Inspired from: <https://www.politifact.com/article/2019/dec/23/key-facts-about-gender-pay-gap-explained/>

Congruent for Conservatives (Gender.R_Hormone):


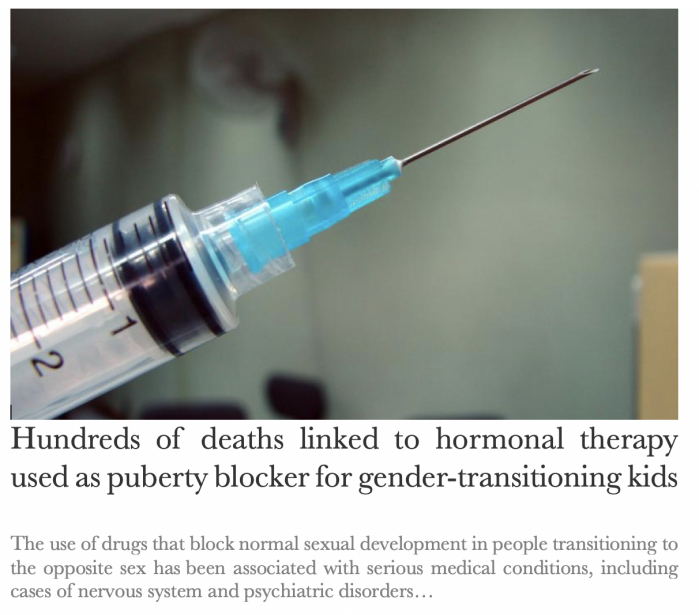


Inspired from: <https://www.lifesitenews.com/news/thousands-of-deaths-linked-to-puberty-blockers-for-gender-confused-kids>

<https://www.nbcnews.com/feature/nbc-out/viral-fake-news-story-linked-trans-healthcare-thousands-deaths-n1059831>

**Abortion**

Congruent for Liberals (Abor.D_Pence):


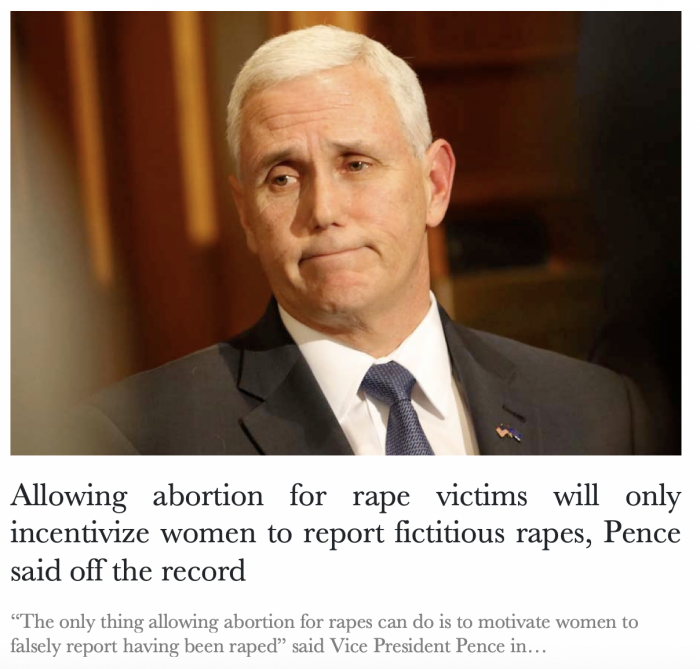


Inspired from: <http://web.archive.org/web/20170205025214/http://politicot.com/mike-pence-allowing-rape-victims-abortions-will-lead-women-trying-get-raped/>

<https://www.factcheck.org/2017/09/fake-mike-pence-abortion-quote/>

Congruent for Conservatives (Abor.R_Bernie):


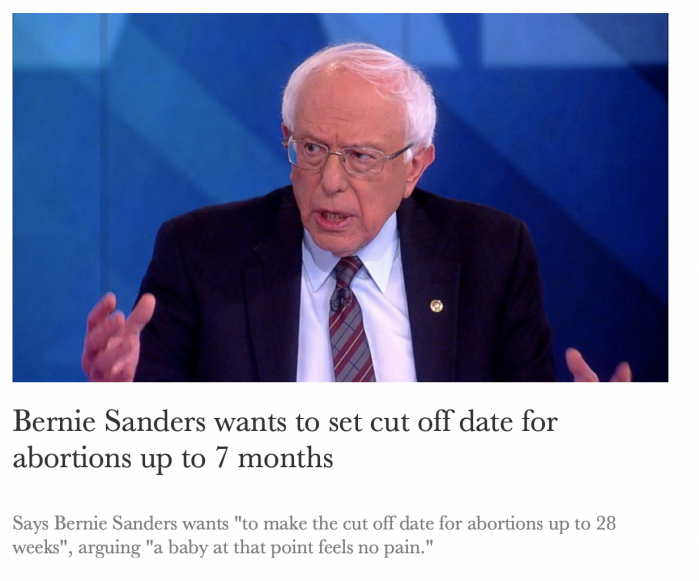


Inspired from: <https://www.politifact.com/factchecks/2020/oct/09/mike-pence/fact-checking-pences-claim-democrats-and-abortion-/>

### Raters’ perceptions of fake political news items’ slant

The following plot shows perceptions of fake news items’ political slant according to MTurk raters:

Means and 95 CI. All one sample t-tests (one-tailed) yield p values < 0.05.


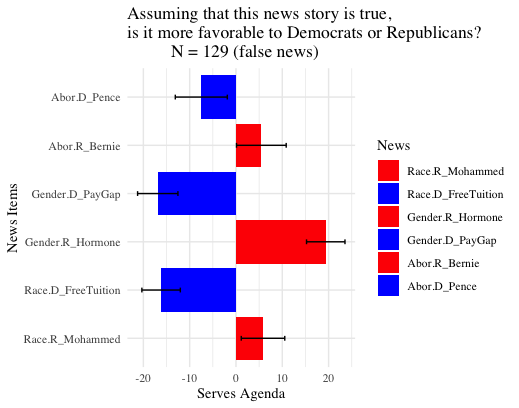


Smallest deviation from 0 is for Abor.R_Bernie: t(128) = 2.02, p = 0.022 (one tailed test)


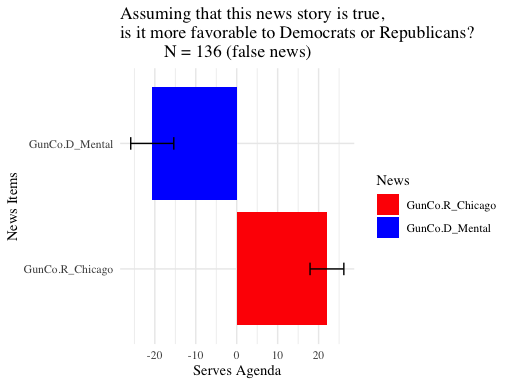


Perceptions of fake news items’ accuracy according to people identifying as Democrat-Liberal (< 50) and as Republican-conservative (>= 50):


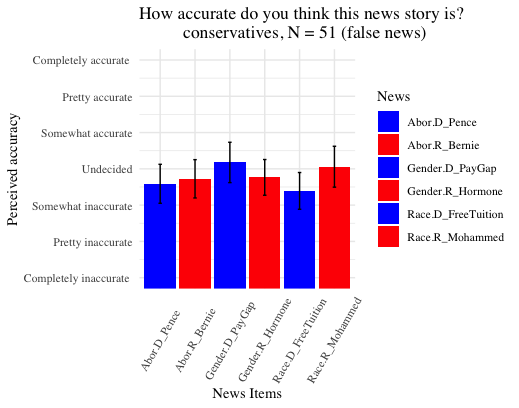

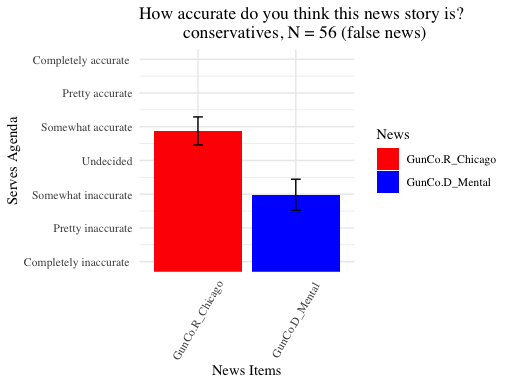


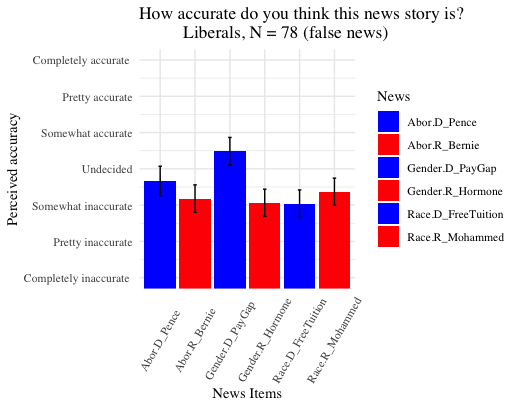


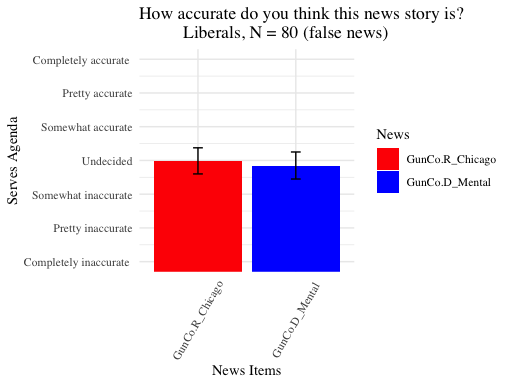


### Experiment 10

**Abortion**

f_abor_d_2


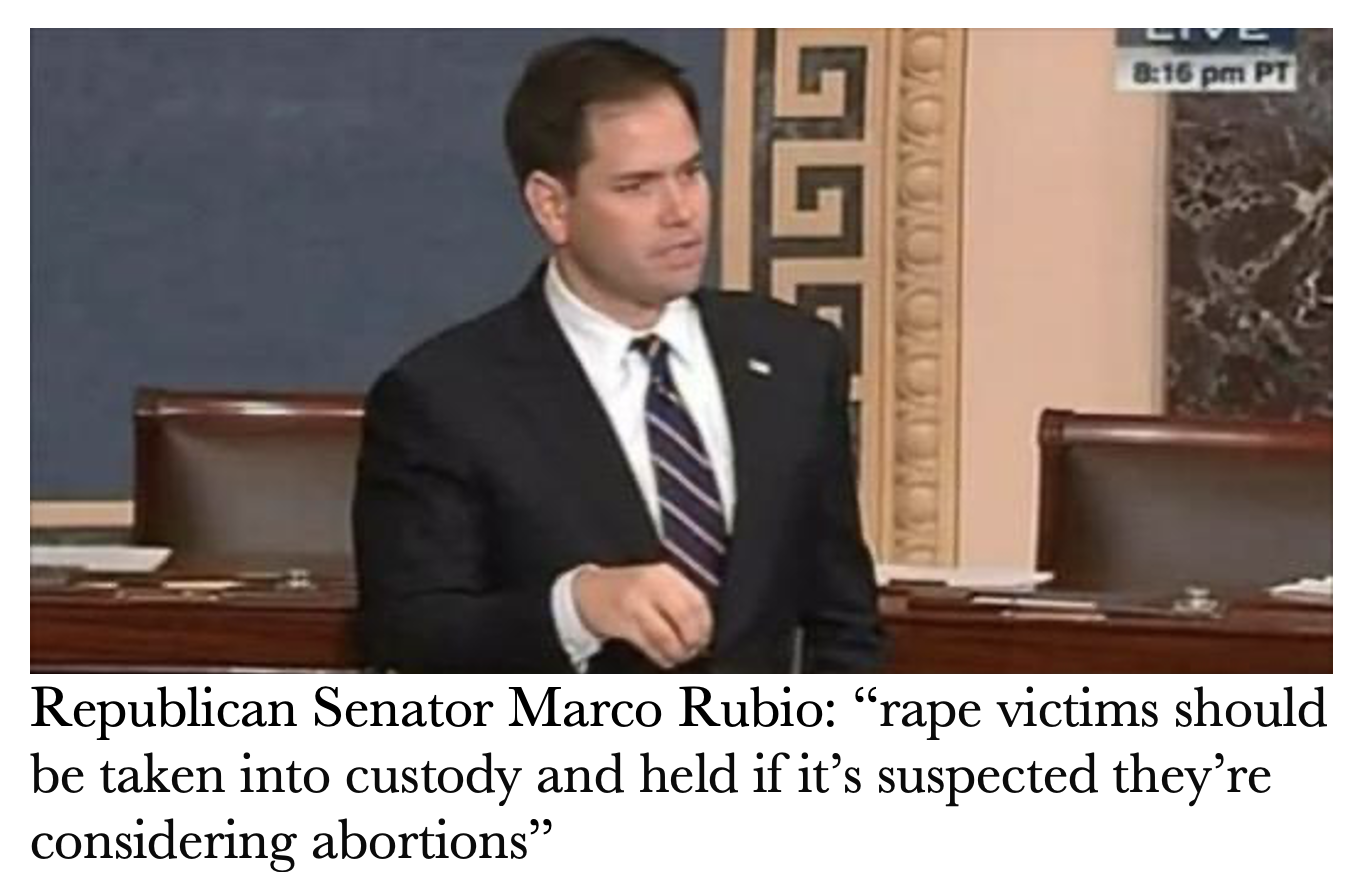


f_abor_r_1


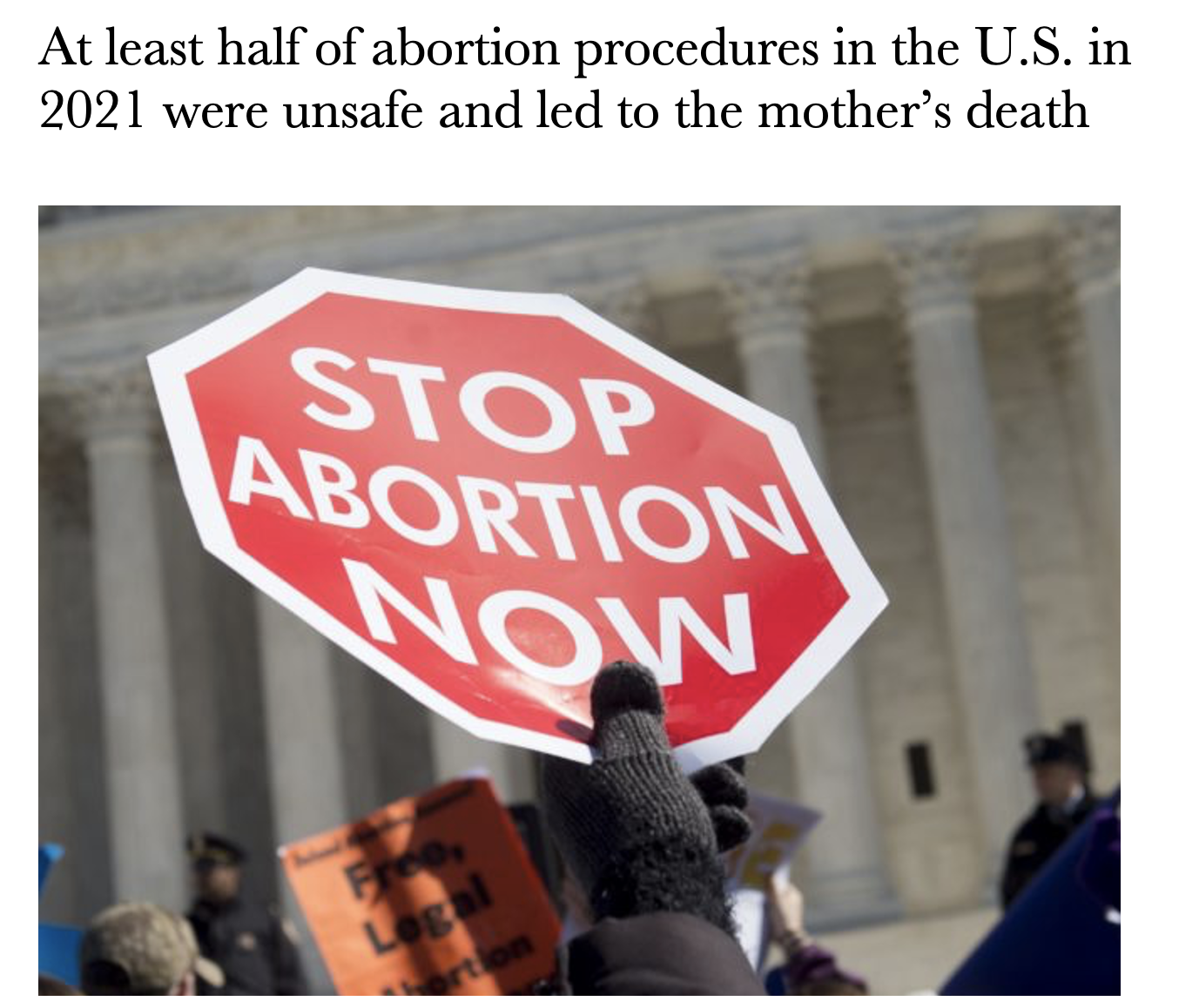


Race equality

f_race_d_1


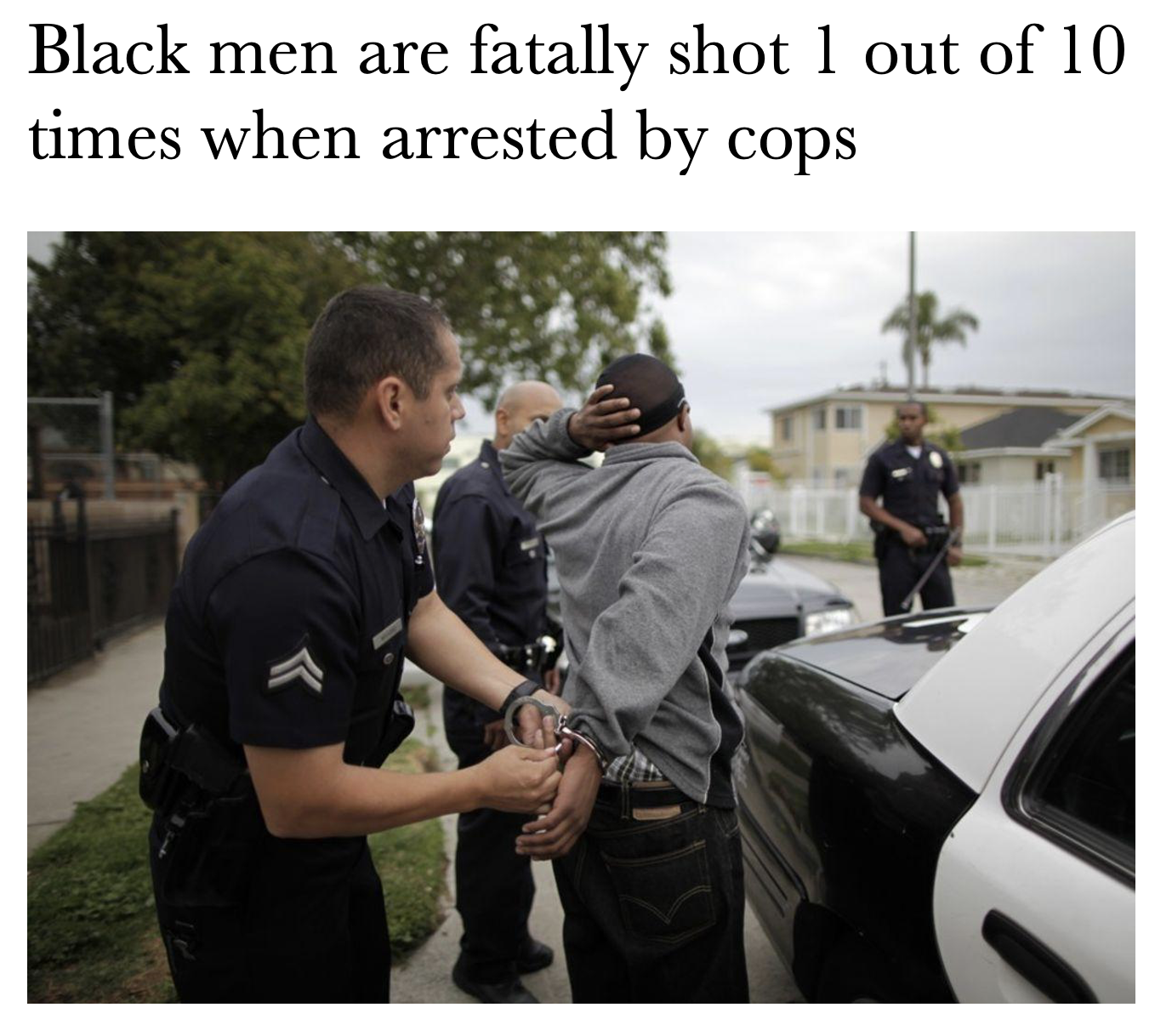


f_race_r_7


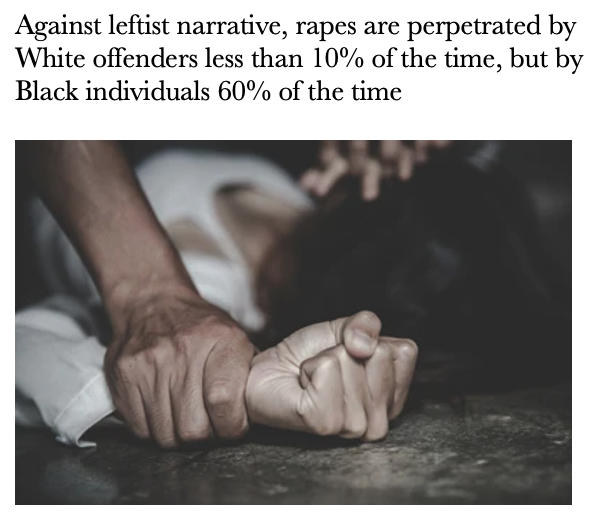


Gender equality

f_gender_d_4


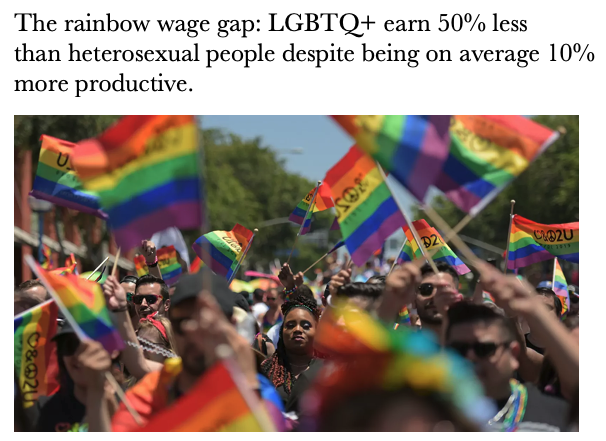


f_gender_r_2


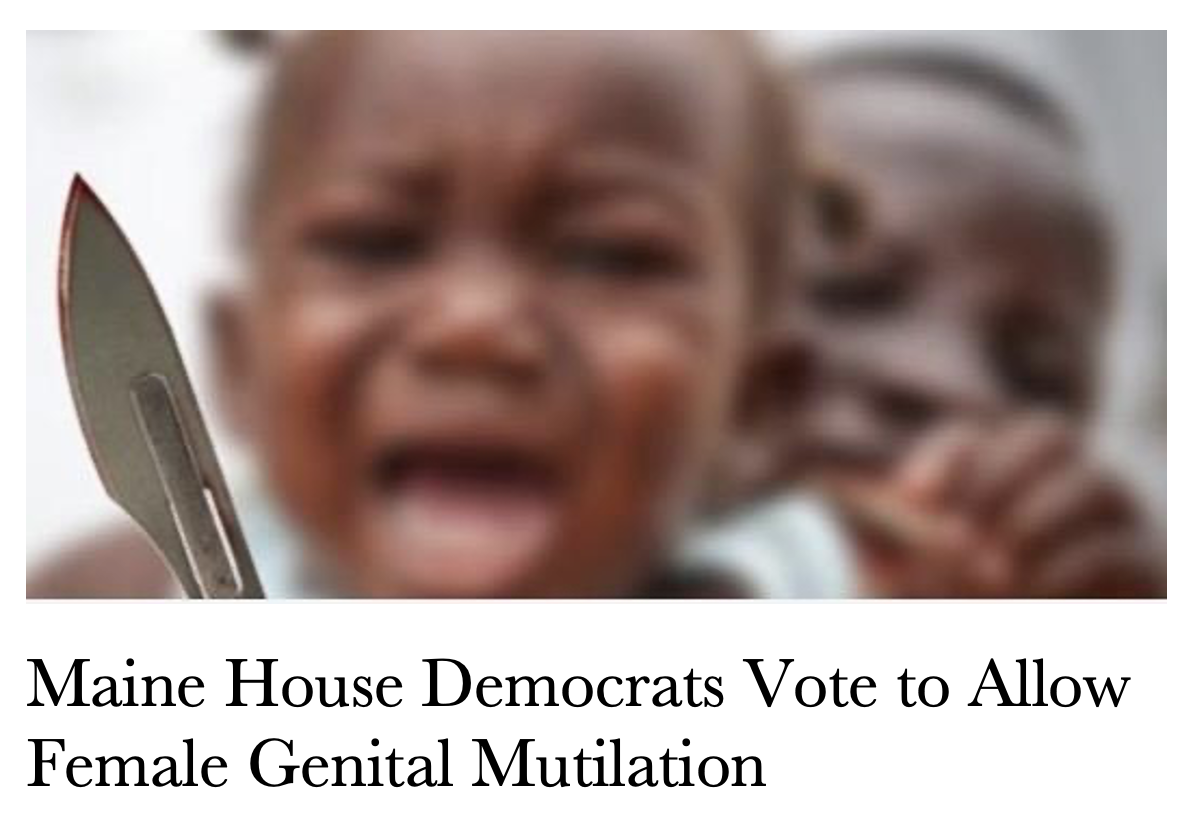


Gun control

f_guns_d_1


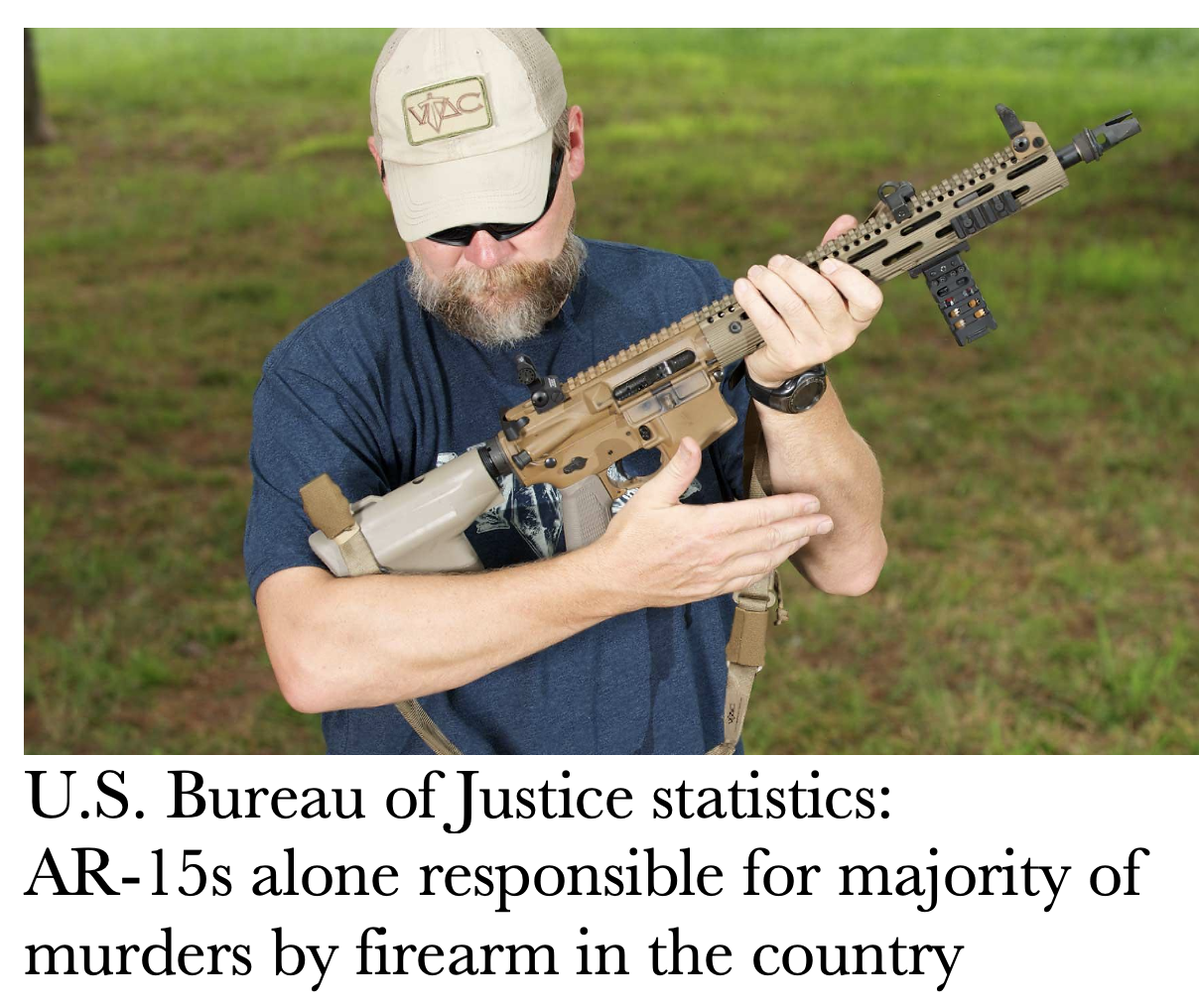


f_guns_r_1


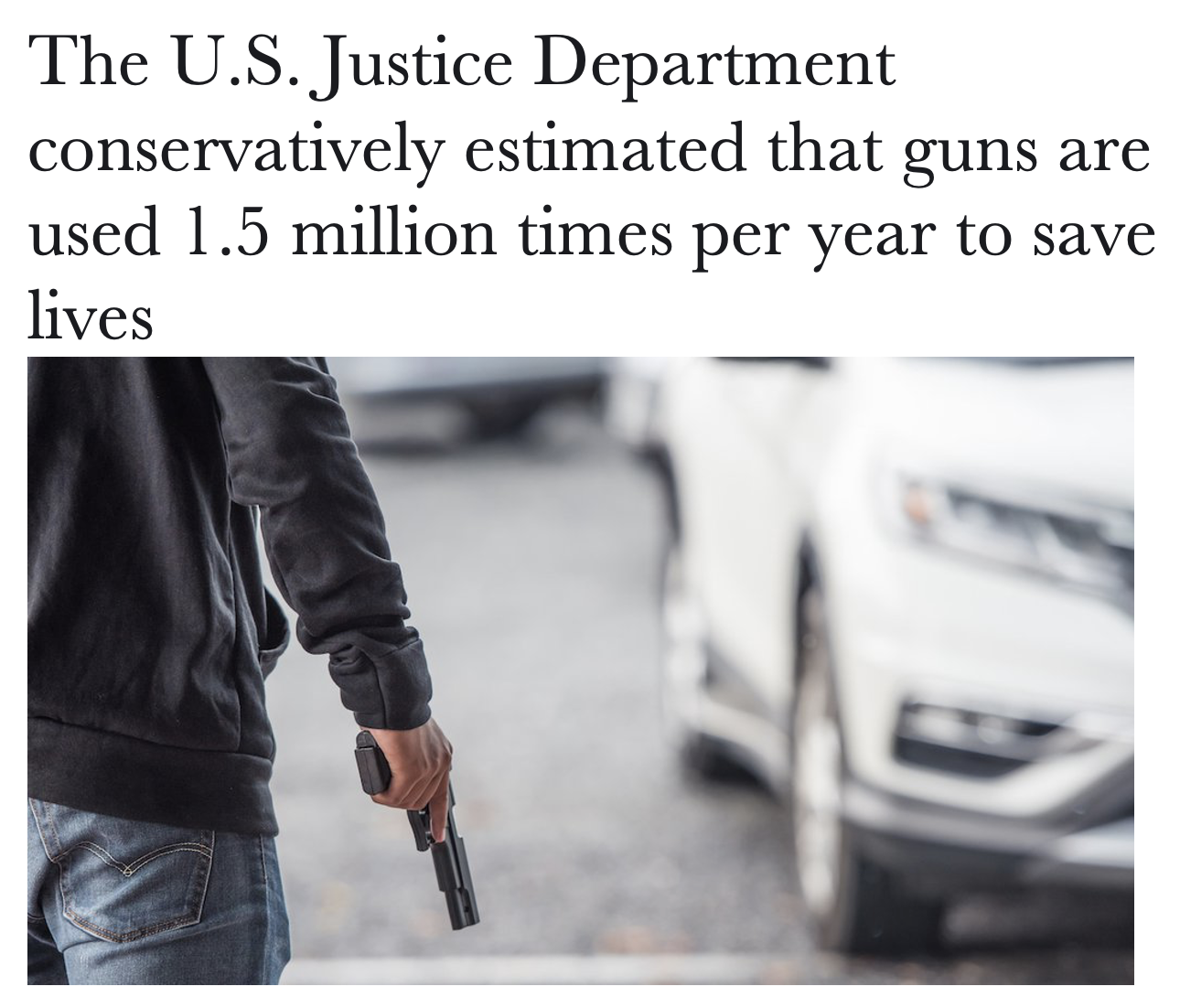


Immigration

f_immi_d_4


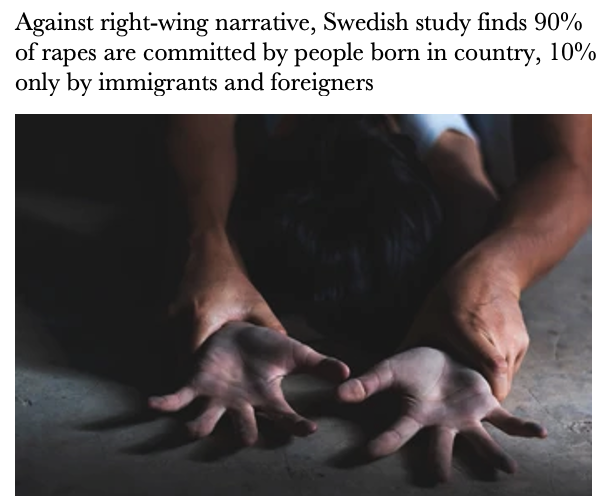


f_immi_r_1


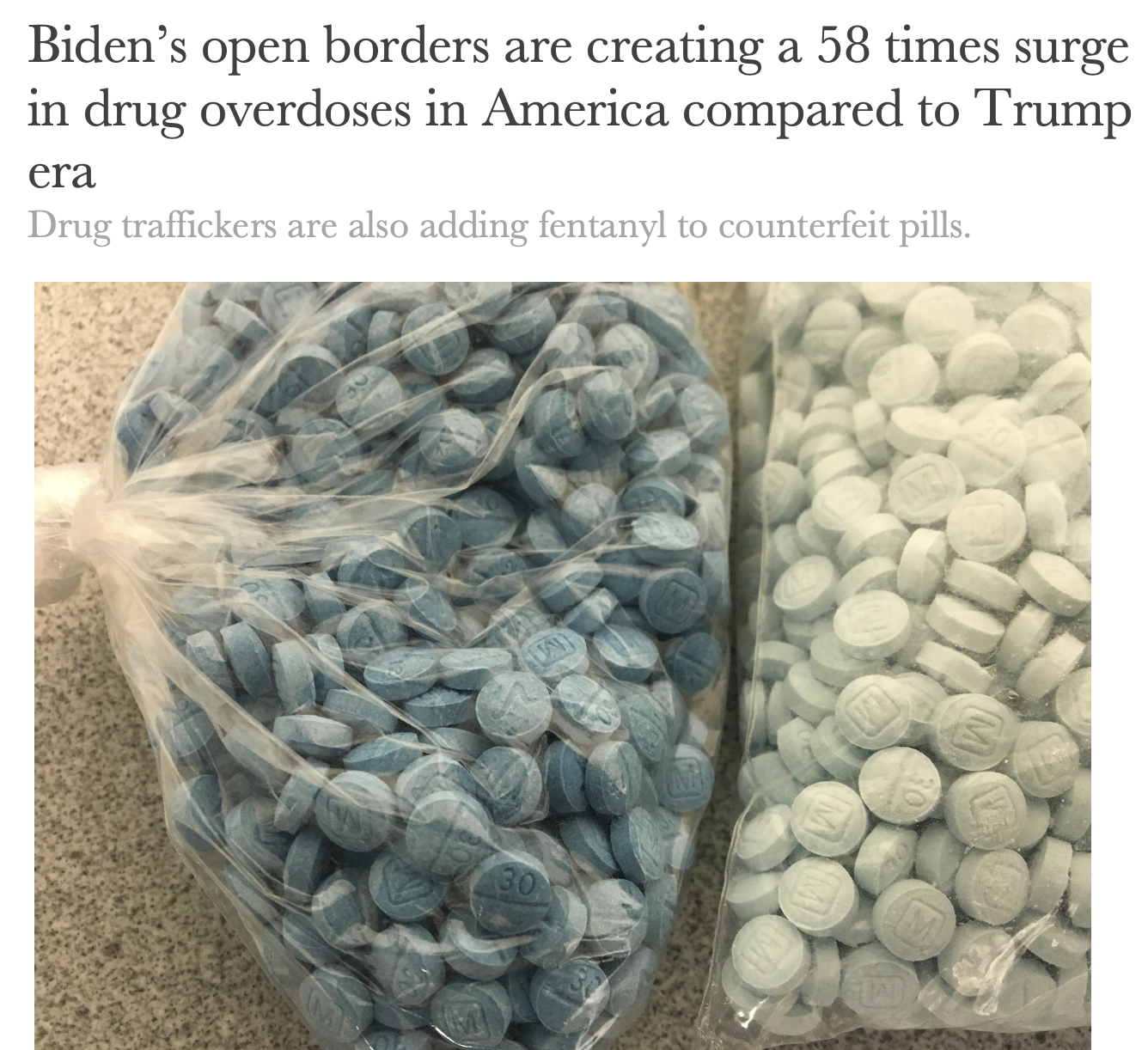


### Raters’ perceptions of fake political news items’ slant

Cf plots in True news of Experiment 10 above

# S. Moral and political attitudes questions

## Attitude on each issue (continuous)

These measures allowed us to determine which news story was congruent and which was incongruent for each participant on each issue. They also allowed us to assess participants’ attitudes on a liberal to conservative axis on each issue.

**Experiments 1-10**

Abor_Attit What is your position on the issue of **abortion?**

|  | Extremely Pro Life | Extremely Pro Choice |
| --- | --- | --- |

| () | 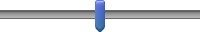 |
| --- | --- |

Gender_Attit What is your position on the issue of **gender equality**?

|  | I don't care at all | Extremely in favor |
| --- | --- | --- |

| () | 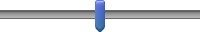 |
| --- | --- |

GunCo_Attit What is your position on the issue of **guns?**

|  | Extremely Pro Gun rights | Extremely Pro Gun control |
| --- | --- | --- |

| () | 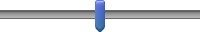 |
| --- | --- |

Race_Attit What is your position on the issue of **racial equality**?

|  | I don't care at all | Extremely in favor |
| --- | --- | --- |

| () | 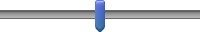 |
| --- | --- |

**Experiment 10**

Immi_Attit What is your position on the issue of immigration?

|  | Extremely opposed | Extremely in favor |
| --- | --- | --- |

|  | 0 | 100 |
| --- | --- | --- |

| () | 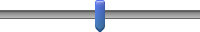 |
| --- | --- |

## Moral importance of issue (dichotomous)

This question was used to assess whether respondents moralized a given issue or not. Order of choices was randomized in Qualtrics.

**Experiments 1-3, 5a, 6a, 7-8**

Is one of the following issues of **absolute importance** to you?

(several choices possible)

Pro-life vs. Pro-choice (1)

Racial equality (2)

Gender equality (3)

Gun control vs. Gun rights (4)

None of the above. Other? (5) ________________________________________________

**Experiments 4, 5b, 6b, 9**

Is one of the following issues of **absolute moral importance** to you? We mean issues that are connected to your core moral beliefs, convictions, and identity. In other words, the issues that are most likely to trigger strong positive or negative emotions in you.

(several choices possible)

Pro-life vs. Pro-choice (1)

Racial equality (2)

Gender equality (3)

Gun control vs. Gun rights (4)

None of the above. Other? (5) ________________________________________________

**Experiment 10**

Is one of the following issues of **absolute moral importance** to you? 
By this, we mean that:
- You're not willing to make compromises with your position on that issue. For instance, you could never vote for a politician if they disagreed with your position on that issue. 
- The issue is connected to your core moral beliefs, convictions, and identity.
- The issue is most likely to trigger strong positive or negative emotions in you.

(several choices possible)

Pro-life vs. Pro-choice (abortion) (1)

Racial equality (2)

Gender equality (3)

Gun control vs. Gun rights (4)

Immigration (5)

None of the above. Other? (6) ________________________________________________

# T. Attention check ‘videogame’ used in all experiments

This attention check was applied to all datasets to delete inattentive participants.

Now imagine you are playing video games with a friend and at some point your friend says:
 “I don’t want to play this game anymore! To make sure that you read the instructions, please write the three following words 'I pay attention' in the box below. I really dislike this game.”
 Do you agree with your friend?

________________________________________________________________

Participants who didn’t write an expression that contained “pay attent” were deleted from the databases.
